# Cascade intramolecular Prins/Friedel–Crafts cyclization for the synthesis of 4-aryltetralin-2-ols and 5-aryltetrahydro-5*H*-benzo[7]annulen-7-ols

**DOI:** 10.3762/bjoc.17.104

**Published:** 2021-06-22

**Authors:** Jie Zheng, Shuyu Meng, Quanrui Wang

**Affiliations:** 1 Department of Chemistry, Fudan University, 2005 Songhu Road, Shanghai 200438, P. R. China

**Keywords:** 4-aryltetralin-2-ol, 5-aryl-benzo[7]annulen-7-ol, cascade reaction, Prins/Friedel–Crafts

## Abstract

The treatment of 2-(2-vinylphenyl)acetaldehydes or 3-(2-vinylphenyl)propanals with BF_3_·Et_2_O results in an intramolecular Prins reaction affording intermediary benzyl carbenium ions, which are then trapped by a variety of electron-rich aromatics via Friedel–Crafts alkylation. This cascade Prins/Friedel–Crafts cyclization protocol paves an expedient path to medicinally useful 4-aryltetralin-2-ol and 5-aryltetrahydro-5*H*-benzo[7]annulen-7-ol derivatives.

## Introduction

**Figure 1:**
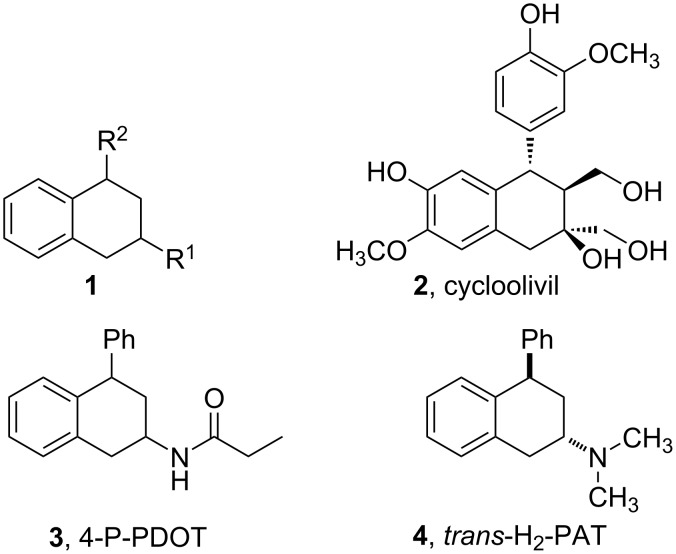
Parent structure of 2,4-disubstituted tetralins (**1**) and selected medicinally useful derivatives **2**–**4**.

**Figure 2:**
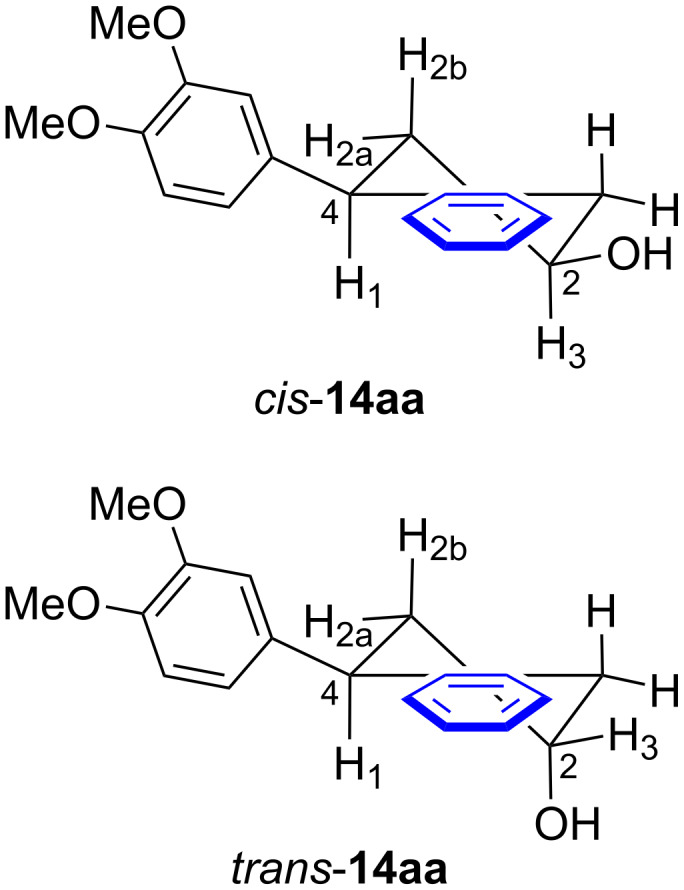
The speculated stereostructures of compound *cis-***14aa** and *trans-***14aa**.

2,4-Disubstituted tetralins (Figure 1, **1**), especially 2-functionalized tetralins are privileged building blocks for medicinal chemistry applications which are known to exhibit a wide spectrum of biological activities [1–3]. Some representative compounds comprising this skeleton are illustrated in Figure 1. Cycloolivil (Figure 1, **2**) [4], which is isolated from the stem bark of *Olea europaea*, has been recognized as inhibitor of cyclic AMP dependent phosphodiesterase, can act as a Ca^2+^ antagonist, and exhibits promising anti-oxidant properties. 4-Phenyl-2-propionamidotetralin (4-P-PDOT, **3**, Figure 1) [5] is a melatonin MT_2_ selective antagonist that can be used to map melatonin receptor subtypes in tissue and serves as a chemical biology tool to identify sub-type selective analogues with therapeutic potential. In addition, *trans*-4-phenyl-*N*,*N*-dimethyl-2-aminotetralin (*trans*-H_2_-PAT, **4**, Figure 1) [6] has been determined to modulate tyrosine hydroxylase activity and dopamine synthesis in rodent forebrain and is also a ligand binding to histamine H_1_ receptors, and thus is a potentially useful therapeutic for psychoses, addiction, and other neuropsychiatric disorders.

**Scheme 1:**
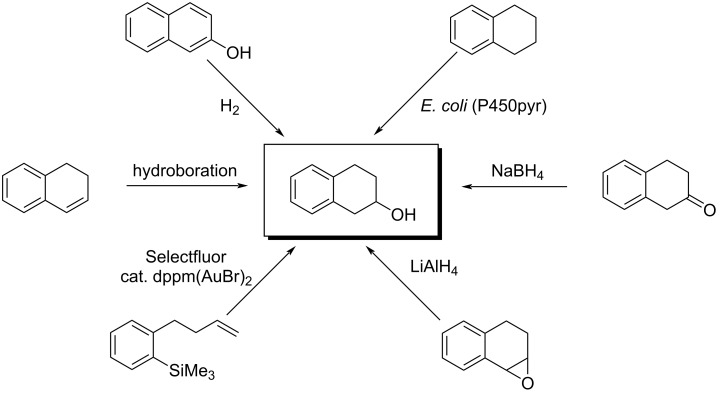
Reported strategies for the synthesis of tetralin-2-ol ring systems.

Although 4-substitituted tetralin-2-ols and derivatives with significant pharmaceutical activities have been identified, only a limited number of synthetic methods is documented in the literature (Scheme 1) [7–9]. Moreover, these methods generally require multiple steps, proceed in low overall yields, and have a limited ability for structural modifications to prepare analogues with new substitution patterns for enhancing activities. Consequently, it is highly desirable to develop new synthetic methods that provide efficient access to 2,4-disubstituted tetralin compounds and thus facilitate their biological investigations.

The cascade Prins/Friedel–Crafts reaction to form multiple chemical bonds in one operation has emerged as an atom-economic and straightforward strategy for the construction of oxygen-containing heterocycles [10–14]. For example, Nagumo and coworkers have developed a Prins/Friedel–Crafts cyclization of homocinnamyl alcohols with aromatic aldehydes under the action of BF_3_·Et_2_O affording 2*H*-indeno[1,2-*b*]furan derivatives [15]. Likewise, Hinkle and coworkers reported in 2017 a three-step domino alkynyl-Prins cyclization, Friedel–Crafts alkenylation, and dehydration/aromatization reaction between 1-aryl-3-hexyne-2,6-diol derivatives and aldehydes, that led to the formation of 1,4-dihydro-2*H*-benzo[*f*]isochromenes [16].

**Scheme 2:**
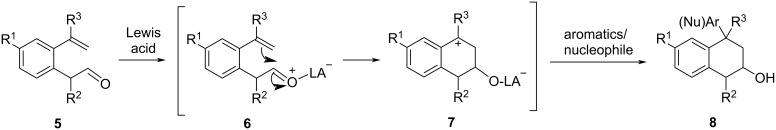
Designed cascade reactions to 4-substituted tetralin-2-ols.

The Prins reaction-induced cyclization, inter alia, became a versatile tool for the assembly of complex molecules from relatively simple and inexpensive materials/reagents in a single operation. The reaction continues to be an interesting and profitable field of research with high impact on synthetic organic chemistry [17–18]. Many of the existing protocols rely on an acid-promoted condensation of a homoallylic alcohol and an aldehyde to give an oxocarbenium ion, which is then reacted with an olefinic/alkynic bond generating a carbocation that undergoes a Friedel–Crafts reaction. Given the potential value of tetralin-2-ol scaffolds to drug research programs, we decided to develop a novel Prins/Friedel–Crafts cyclization strategy for the synthesis of 4-aryl-2-hydroxytetralins starting from 2-(2-vinylphenyl)acetaldehydes (Scheme 2). In this protocol, we envisioned that the aldehyde **5** would give rise to an oxocarbenium ion species **6** upon treatment with a Lewis acid. The intermediate **6** then would undergo a Prins-type intramolecular cyclization with the olefinic bond to produce a stable benzyl carbocation **7**, that may be trapped through a Friedel–Crafts alkylation with an aromatic substrate or through the reaction with an external nucleophile to afford the target product **8**.

## Results and Discussion

**Scheme 3:**
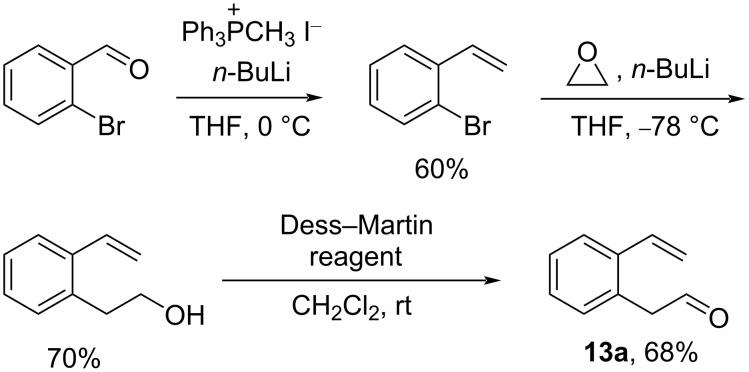
The documented synthesis of 2-(2-vinylphenyl)acetaldehyde (**13a**).

Our research began with the preparation of 2-(2-vinylphenyl)acetaldehydes (**13**) required as substrates for the Prins/Friedel–Crafts cyclization reactions. Commonly, these aromatic alkenyl aldehydes were previously prepared via a three step process as exemplified by **13a** shown in Scheme 3 consisting of the following steps: (i) Wittig reaction of 2-bromobenzaldehyde with methyltriphenylphosphonium iodide ylide, (ii) lithiation of the resultant *o*-bromostyrene with *n*-BuLi and reaction of the aryl lithium species with ethylene oxide, and (iii) oxidation of the resultant primary alcohol using Dess–Martin periodinane [19–20].

**Scheme 4:**
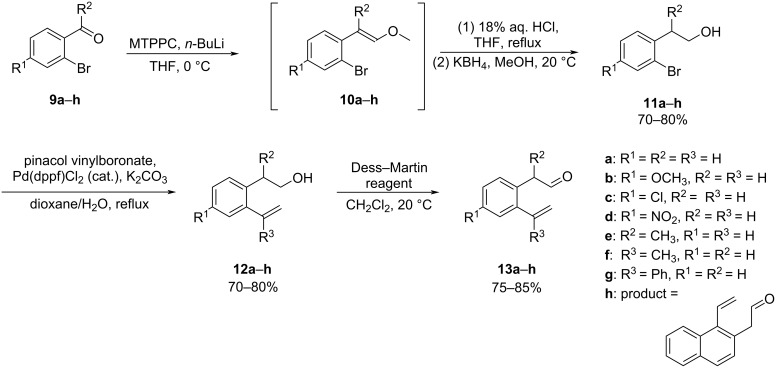
Modified synthesis of 2-(2-vinylphenyl)acetaldehydes **13a**–**g** and 1-vinyl-2-naphthaldehyde (**13h**).

The reported methods involved the use of ethylene oxide, a hazardous and carcinogenic gas. This prompted us to work out a more practical and flexible method to access the aromatic enal compounds **13**. At the offset, we examined the synthesis of 2-(2-vinylphenyl)acetaldehyde (**13a**) using the route as outlined in Scheme 4. The synthesis started with the Wittig reaction of 2-bromobenzaldehyde (**9a**) with (methoxymethyl)triphenylphosphonium chloride (MTPPC) upon action with *n*-butyllithium in THF at 0 °C to give the vinyl ether **10a** that was subjected to acidic hydrolysis using 18% aq HCl furnishing the corresponding aldehyde [21]. Without purification, the resultant aldehyde intermediate was then directly reduced using potassium borohydride to the corresponding primary alcohol **11a** in 74% yield starting from **9a**. Pd-catalyzed cross-coupling of **11a** with pinacol vinylboronate afforded the *o*-hydroxyethyl-styrene **12a** in 78% yield [22–23]. Next, Dess–Martin oxidation of the alcohol **12a** was carried out, and the desired 2-(2-vinylphenyl)acetaldehyde (**13a**) was successfully obtained in 85% yield. Obviously, this modified method has the advantages of mild reaction conditions, operational simplicity, and using cheap and non-toxic reagents.

The modified procedure was then expanded to the synthesis of a set of 2-(2-vinylphenyl)acetaldehydes **13b**–**f** starting from differently substituted 2-bromobenzaldehydes **9** or 1-(2-bromophenyl)ethan-1-one (**9e**) in comparable yields. Likewise, 2-(1-vinylnaphthalen-2-yl)acetaldehyde (**13h**) was prepared from 1-bromo-2-naphthaldehyde in 48% yield over the three steps. It should be noted that the nitro-substituted intermediate **11d** was prepared by nitration of **11a** with nitric acid under the promotion of acetic anhydride.

**Scheme 5:**
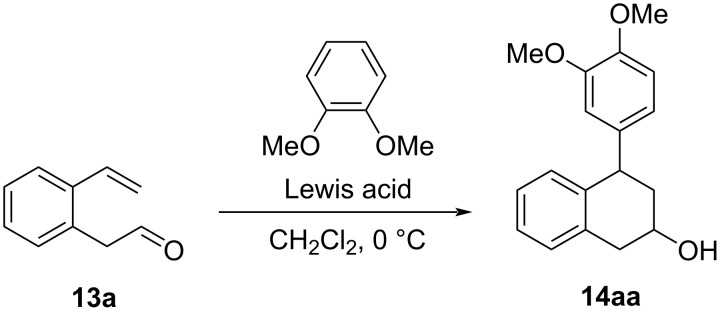
Lewis acid-catalyzed Prins/Friedel–Crafts reaction of **13a** with veratrole.

With the accessibility of the aromatic vinyl aldehydes **13**, next the cascade Prins/Friedel–Crafts reaction was examined. We started our investigations by applying aldehyde **13a** as the model substrate (Scheme 5). A Lewis acid screening was carried out to identify the best catalyst for the tandem intramolecular Prins/Friedel–Crafts reaction (Table 1). Thus, the portion-wise addition of AlCl_3_ (1.1 equiv) to a stirred mixture of **13a** (1.0 equiv) and veratrole (1.05 equiv) in CH_2_Cl_2_ at 0 °C resulted in the intramolecular Prins reaction to generate a benzyl carbenium ion that concurrently underwent Friedel–Crafts reaction with veratrole, leading to the formation of the expected 4-(3,4-dimethoxyphenyl)-1,2,3,4-tetrahydronaphthalen-2-ol (**14aa**), 51:49 mixture of *cis*/*trans*-diastereomers) as a colorless oil in 50% yield (Table 1, entry 1). The use of Et_2_AlCl as the Lewis acid gave tetralin **14aa** in a slightly improved 55% yield (Table 1, entry 2). However, the reaction with AlMe_3_ as the promotor resulted in a competing reduction of **13a** to 2-bromophenylethanol (**12a**) that was obtained as the major product (Table 1, entry 3). Switching to the weaker Lewis acid In(OTf)_3_ failed to induce any intramolecular Prins cyclization (Table 1, entry 4), whilst the use of FeCl_3_ produced **14aa** in a similar 52% yield as observed for AlCl_3_ (Table 1, entry 5). To our delight, 1.1 equivalents of BF_3_·Et_2_O were found to promote the transformation efficiently, and a 70% isolated yield of **14aa** was obtained (Table 1, entry 6). However, experiments with BF_3_·Et_2_O at substoichiometric amounts afforded significantly decreased yields of **14aa** (Table 1, entries 7 and 8).

**Table 1:** Screening of Lewis acid catalysts.^a^

entry	Lewis acid	amount of LA [mol %]	*cis*/*trans* ratio^b^	yield [%]^c^

1	AlCl_3_	110	51:49	50
2	Et_2_AlCl	110	50:50	55
3	AlMe_3_	110	NA	0^d^
4	In(OTf)_3_	110	NA	0
5	FeCl_3_	110	50:50	52
6	BF_3_·Et_2_O	110	49:51	70
7	BF_3_·Et_2_O	80	50:50	50
8	BF_3_·Et_2_O	50	50:50	35

^a^Reaction conditions: a mixture of **13a** (1.40 mmol), veratrole (1.47 mmol) and Lewis acid (1.54 mmol) in CH_2_Cl_2_ (6 mL) was stirred at 0 °C for 2 h; ^b^*cis*/*trans* ratios were determined by ^1^H NMR spectroscopy; ^c^isolated yield after chromatography; ^d^reduction product **12a** instead of the desired **14aa** was identified.

The relative *cis*- and *trans-*configuration of the C-2 hydroxy group and the C-4 aryl substituent (Figure 2) were assigned on the basis of ^1^H-^1^H COSY analysis. Firstly, the HSQC analysis was used to determine H_3_. The ^13^C NMR chemical shift for C_2_ is expected to be in the range of 60 to 70 ppm and the assignment of H_3_ was based on the HSQC correlation between H_3_ and C_2_. Then, H_1_ and H_2_ could be assigned by COSY and HSQC experiments. Following that, NOE analysis was applied to analyze the relative *cis-* and *trans-*configuration. If there is an NOE correlation between H_1_ and H_3_, and meanwhile H_1_ and H_3_ also have a strong NOE correlation with H_2a_, the compound is assigned to be *cis-*configured. Otherwise, it was assigned to be the *trans-*isomer (see Supporting Information File 1 for details).

**Scheme 6:**
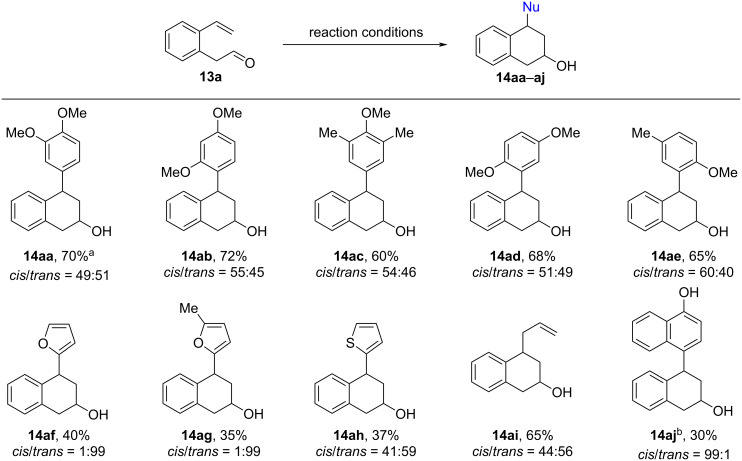
Use of different nucleophiles for the cascade reaction with **13a**. Reaction conditions: a mixture of **13a** (1.40 mmol), nucleophile (1.47 mmol), BF_3_·Et_2_O (1.54 mmol) in anhydr. CH_2_Cl_2_ (6 mL) was stirred at 0 °C for 2 h. ^a^Isolated yield by chromatography; ^b^isolated by preparative HPLC.

Having determined the suitable reaction conditions, we surveyed the scope and limitation of the cascade protocol. Initially, we explored the range of nucleophiles that were used to intercept the benzyl carbenium ion and the results are summarized in Scheme 6. All reactions with electron-rich aromatics containing a *p*- and/or *o*-methoxy substituent as the nucleophile proceeded well to give the desired 2-hydroxy-4-aryltetralins **14aa**–**ae** as 49:51 to 60:40 mixtures of *cis*/*trans* diastereomers in moderate to good yields.

The electron-rich 5-membered heterocycles like furans and thiophene participated also smoothly in the reaction sequence, leading to the clean formation of the respective 2-hydroxy-4-heteroaryltetralins **14af**–**ah**, although the yields were somewhat lower than that with substituted anisole derivatives. As an attempt to enlarge the generality, tetraallysilane was also examined. To our delight, this substrate also participated in the reaction leading to the 4-allyl-substituted tetrahydronaphthalen-2-ol **14ai** in 65% yield.

On comparing the results from the anisole-type nucleophiles or thiophene with that from furans, it was observed that the reactions with furans furnished predominantly *trans*-**14af** and *trans*-**14ag** with a high degree of diastereoselectivity (*cis*/*trans* ratio = 1:99). The preferential formation of the *trans*-configured products for furan nucleophiles may be due to the fact that the addition of furan is reversible leading to equilibration to the more stable trans product. To test this hypothesis, we monitored the reaction by HPLC (Table 2). As expected, we observed that the initially formed *cis-*isomer of **14af** turned gradually to *trans*-**14af** and finally reached 1:99 after 2 hours (for further details, see Supporting Information File 1).

**Table 2:** Dependency of *cis*/*trans* ratio of product **14af** on conditions and time.

entry	conditions/reaction time	*cis*/*trans* ratio^a^

1	addition 20% of BF_3_·Et_2_O	29:71
2	addition 100% of BF_3_·Et_2_O	23:77
3	further stirred for 30 min	21:79
4	further stirred for 60 min	12:88
5	further stirred for 120 min	1:99

^a^*cis*/*trans* ratio was examined by HPLC.

To further expand the substitution pattern, we then tried the reaction of **13a** with allysilane as a carbon-nucleophile. As expected, the 4-allyl-substituted tetrahydronaphthalen-2-ol **14ai** was obtained, again, as a mixture of *cis*/*trans-*isomers in a ratio of 44:56. This example demonstrates the general synthetic utility of this cascade protocol.

**Scheme 7:**
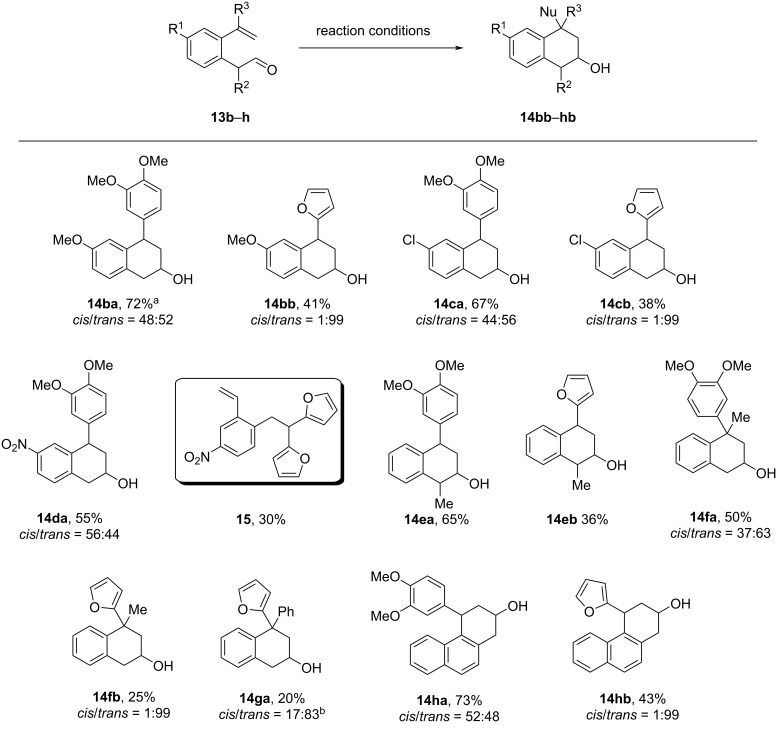
Reaction of aldehydes **13b**–**h** with veratrole or furan. Reaction conditions: a mixture of **13b**–**h** (1.40 mmol), nucleophile (veratrole or furan, 1.47 mmol), BF_3_·Et_2_O (1.54 mmol) in anhydr. CH_2_Cl_2_ (6 mL) was stirred at 0 °C for 2 h. ^a^Isolated yield by chromatography; ^b^*cis*-**14ga** refers to the structure with furyl and hydroxy substituents residing at the same side.

Encouraged by the success of using **13a** as the substrate, the reactions with other 2-(2-vinylphenyl)ethanals **13b**–**g** carrying different substituents on the benzene ring or on the side chain with veratrole and furan as the nucleophiles were investigated. As can be seen from Scheme 7, under comparable conditions, most reactions proceeded smoothly with the attempted alkenyl-aldehydes **13** to furnish the corresponding 2,4-disubstituted tetralins **14ba**–**hb** in acceptable to good isolated yields. For instance, the reaction with aldehydes **13** containing π-donating substituents like methoxy and chloro substituents afforded the 2-hydroxy-4-aryltetralin products **14ba**–**cb** in 38–72% yield. To our gladness, aldehyde **13d**, with an electron-deficient nitro group residing on the benzene ring reacted with veratrole under the standard conditions, delivering tetralin **14da** in 55% yield. However, using furan as the nucleophile component, the reaction sequence with **13d** failed to give the tetralin product. Instead, we only isolated 30% yield of the difuranyl-substituted compound **15** as the major product.

In addition, aldehydes **13e** or **13f** bearing a methyl group at the acetaldehyde side or the benzylic position of the alkene side were also suitable substrates for this cascade strategy: the 1,2,4-trisubsitituted tetralins **14ea** and **14eb** as well as the 2,4,4-trisubsitituted tetralins **14fa** and **14fb** were obtained in moderate to reasonable yields. The aldehyde **13g** bearing a phenyl group at the benzylic position of the alkene side was also tried. Under the standard conditions, the 1,2,4-trisubsitituted tetralin **14ga** was isolated as a 17:83 mixture of *cis*/*trans isomers*, but with 20% yield. The poor yield may be attributed to the enhanced steric hindrance. This cyclization methodology was also applicable to 2-(1-vinylnaphthalen-2-yl)acetaldehyde (**13h**), for which the reaction with veratrole or furan led to the formation of the respective tricyclic 4-aryl-1,2,3,4-tetrahydrophenanthren-2-ols **14ha** and **14hb** in 73% and 43% yields, respectively.

**Scheme 8:**
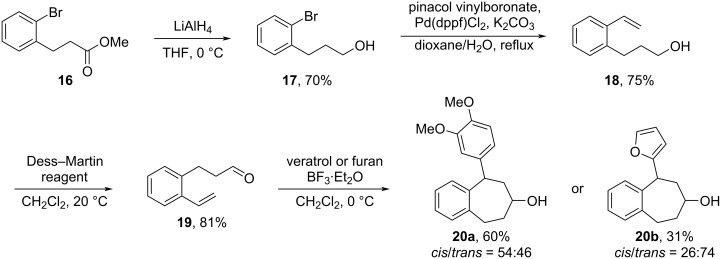
Synthesis of 5-aryltetrahydro-5*H*-benzo[7]annulen-7-ols **20a**, **b**.

In order to further explore the generality of this cascade Prins/Friedel–Crafts cyclization, the established methodology was also applied to the formation of tetrahydro-5*H*-benzocyclohepten-7-ol ring systems. As shown in Scheme 8, the required homo-aldehyde substrate **19** was prepared starting from methyl 3-(2-bromophenyl)propionate (**16**) analogously as for **13**. Reduction of the ester **16** with LiAlH_4_ in THF at 0 °C afforded the alcohol **17** that was subjected to a Suzuki reaction with pinacol vinylboronate using Pd(dppf)Cl_2_ as catalyst to produce 3-(2-vinylphenyl)propan-1-ol (**18**). The oxidation of alcohol **18** with Dess–Martin oxidizing reagent furnished the requisite aldehyde **19** in 43% yield over the three steps.

Under the standard conditions, aldehyde **19** underwent satisfactorily the cascade Prins/Friedel–Crafts cyclization with veratrole or furan as the nucleophile furnishing the tetrahydro-5*H*-benzo[7]annulen-7-ol **20a** (*cis*/*trans* ratio = 54:46) and **20b** (*cis*/*trans* ratio = 26:74) in 60% and 31% yield, respectively. The predominance of the *trans*-product for the reaction with furan further verified the oxophilic character of the employed BF_3_, although the stereoselectivity considerably decreased in comparison with the formation of tetralin ring system as the distance between the reaction sites is increased. It is worth mentioning that the tetrahydro-5*H*-benzo[7]annulen-7-ol skeleton is also of considerable medicinal significance and has attracted much synthetic efforts [24–25].

**Scheme 9:**
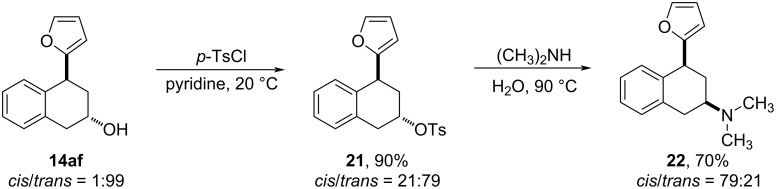
Conversion of 2-hydroxy-4-(2-furyl)tetralin (**14af**) into PAT analogue **22**.

Finally, the ability to structurally diversify the 2-hydroxy-4-substituted tetralin skeletons into medicinally useful derivatives was demonstrated by converting 2-hydroxy-4-furyl-tetralin **14af** into the PAT analogue **22** (see Figure 1) [26]. The reaction of **14af** with *p*-toluenesulfonyl chloride in pyridine afforded the tosylate **21** in 90% yield, which was then treated with 40% aqueous dimethylamine to produce the tertiary amine containing PAT analogue **22** (*cis*/*trans* ratio = 79:21) in 70% yield (Scheme 9). With regard to the partial epimerization of product **21**, it may be due to the action of pyridine. In the preparation of compound **21**, pyridine is used both as solvent and the acid acceptor. Because pyridine itself can show nucleophilic reactivity in addition to basicity, the long reaction time of 20 hours may lead to an ion-pair species with **21** and hence erode the stereochemistry. To prove this idea, we performed the reaction with CH_2_Cl_2_ as the solvent in the presence of 5.0 equivalents of pyridine and 2.0 equivalents of TsCl. Under these conditions, the tosylate **21** was obtained with full retention of the expected stereochemistry (*cis*/*trans* =1:99) (see Supporting Information File 1 for details). The conversion of tosylate **21** to product **22** proceeded in a typical S_N_2 manner resulting in the expected inversion of the configuration.

**Figure 3:**
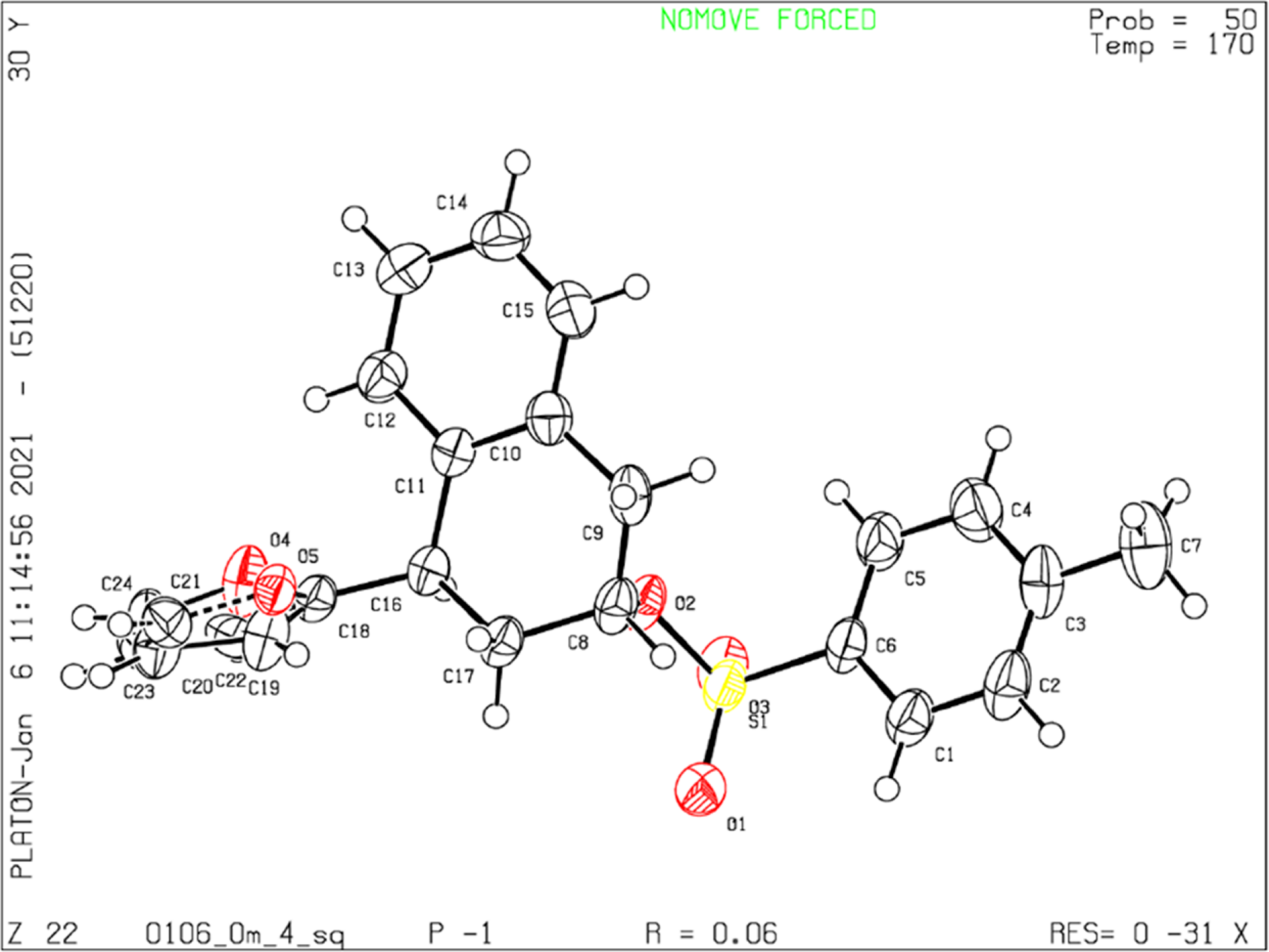
Crystal structure of the tosylate **21**. The displacement ellipsoids are drawn at the 30% probability level.

To unequivocally support the configuration assignment made by NMR analysis, the sulfonate derivative **21** from **14af** was prepared by reaction with tosyl chloride. For compound **21**, we were able to obtain single crystals suitable for X-ray analysis and the X-ray diffraction studies on **21** confirmed undoubtedly its *trans*-configuration. The ORTEP structure is shown in Figure 3 [27].

## Conclusion

In summary, a Prins cyclization and Friedel–Crafts cascade reaction strategy for the synthesis of 4-aryl-tetralin-2-ols and 5-aryl-tetrahydro-5*H*-benzo[7]annulen-7-ols has been established. The sequence involved the Prins cyclization of 2-(2-vinylphenyl)acetaldehydes or 3-(2-vinylphenyl)propanal by action with BF_3_ to generate benzyl carbenium ions that are captured by a Friedel–Crafts alkylation reaction with a range of electron-rich benzenes or heteroaromatics. The method has a relatively broad applicability allowing variation in the benzene ring as well as in the side chain. The further manipulation of the hydroxy group affording the PAT analogue demonstrated the synthetic potential for accessing medicinally useful derivatives.

## Supporting Information

The Supporting Information contains experimental procedures, characterization data of all isolated products as well as copies of NMR spectra and XRPD data for compound **21**.

File 1 Experimental section.

## References

[1] Ichikawa K, Kinoshita T, Nishibe S, Sankawa U (1986). Chem Pharm Bull.

[2] Dubocovich M L, Masana M I, Iacob S, Sauri D M (1997). Naunyn-Schmiedeberg's Arch Pharmacol.

[3] Wyrick S D, Booth R G, Myers A M, Owens C E, Kula N S, Baldessarini R J, McPhail A T, Mailman R B (1993). J Med Chem.

[4] Moritani Y, Ukita T, Ohmizu H, Iwasaki T (1995). J Chem Soc, Chem Commun.

[5] Lucarini S, Bedini A, Spadoni G, Piersanti G (2008). Org Biomol Chem.

[6] Bucholtz E C, Brown R L, Tropsha A, Booth R G, Wyrick S D (1999). J Med Chem.

[7] Makowski P, Cakan R D, Antonietti M, Goettmann F, Titirici M-M (2008). Chem Commun.

[8] Zhang W, Tang W L, Wang Z, Li Z (2010). Adv Synth Catal.

[9] Brenzovich W E, Brazeau J-F, Toste F D (2010). Org Lett.

[10] Indukuri K, Unnava R, Deka M J, Saikia A K (2013). J Org Chem.

[11] Reddy B V S, Sundar C S, Reddy M R, Reddy C S, Sridhar B (2015). Synthesis.

[12] Li R-Q, He Y, Ding Y, Ang C-K, Tian J-S, Loh T-P (2018). Chem Commun.

[13] Barakov R, Shcherban N, Yaremov P, Bezverkhyy I, Čejka J, Opanasenko M (2020). Green Chem.

[14] Kotipalli T, Hou D-R (2018). Org Lett.

[15] Sakata Y, Yasui E, Takatori K, Suzuki Y, Mizukami M, Nagumo S (2018). J Org Chem.

[16] Hinkle R J, Chen Y, Nofi C P, Lewis S E (2017). Org Biomol Chem.

[17] Olier C, Kaafarani M, Gastaldi S, Bertrand M P (2010). Tetrahedron.

[18] Padmaja P, Reddy P N, Reddy B V S (2020). Org Biomol Chem.

[19] Grigg R D, Van Hoveln R, Schomaker J M (2012). J Am Chem Soc.

[20] Fustero S, Rodríguez E, Lázaro R, Herrera L, Catalán S, Barrio P (2013). Adv Synth Catal.

[21] Chua P J, Tan B, Yang L, Zeng X, Zhu D, Zhong G (2010). Chem Commun.

[22] Dai X-J, Engl O D, León T, Buchwald S L (2019). Angew Chem, Int Ed.

[23] Firmansjah L, Fu G C (2007). J Am Chem Soc.

[24] Singh K N, Singh P, Sharma E, Kaur M, Deol Y S (2015). Synthesis.

[25] Wyrick S D, Booth R G, Myers A M, Owens C E, Bucholtz E C, Hooper P C, Kula N S, Baldessarini R J, Mailman R B (1995). J Med Chem.

[26] Booth R G (2010). Therapeutic compounds. Int. Pat. Appl..

[R27] 27CCDC 2060394 contains the supplementary crystallographic data for this paper. These data can be obtained free of charge from the Cambridge Crystallographic Data Centre via http://www.ccdc.cam.ac.uk./data_request/cif.

