# Supplementary material for: Cascade intramolecular Prins/Friedel–Crafts cyclization for the synthesis of 4-aryltetralin-2-ols and 5-aryltetrahydro-5H-benzo[7]annulen-7-ols
Source: Beilstein J Org Chem. 2021 Jun 22;17:1481–9. doi: 10.3762/bjoc.17.104 (PMC8239259; doi:10.3762/bjoc.17.104)
Supplement: File 1 — Experimental section. [file Beilstein_J_Org_Chem-17-1481-s001.pdf]

BEILSTEIN JOURNAL OF ORGANIC CHEMISTRY

Supporting Information

for

Cascade intramolecular Prins/Friedel–Crafts cyclization for the synthesis of 4-aryltetralin-2-ols and 5-aryltetrahydro-5*H*-benzo[7]annulen-7-ols

Jie Zheng, Shuyu Meng and Quanrui Wang

Beilstein J. Org. Chem. **2021,** *17,* 1481–1489. doi:10.3762/bjoc.17.104

Experimental section

License and Terms: This is a supporting information file under the terms of the Creative Commons Attribution License (https://creativecommons.org/licenses/by/4.0). Please note that the reuse, redistribution and reproduction in particular requires that the author(s) and source are credited and that individual graphics may be subject to special legal provisions.
The license is subject to the *Beilstein Journal of Organic Chemistry* terms and conditions: (https://www.beilstein-journals.org/bjoc/terms)

Table of contents

1. General information S2
2. Experimental procedures and characterization data S3
3. *cis*- and *trans*-Configuration analysis method for 4-aryl-tetralin-2-ols S17
4. *trans*-Stereoselectivity study on **14af** and related spectra S19
5. Stereochemical purity erosion study of **21** and related spectra S22
6. Copies of NMR spectra S24
7. X-ray crystal data for compound **21** S99
8. References S102

1. General Information

All reactions were carried out in oven-dried glassware under a nitrogen atmosphere. All solvents and reagents were purchased from Sinopharm Chemical Reagent Co. Ltd. (SCRC). Commercially available reagents were used without further purification. For the sequential Prins/Friedel–Crafts reactions, dehydrated CH_2Cl_2 was prepared by distillation from CaH_2. ^1H NMR (400 MHz) and ^{13}C NMR (101 MHz) spectra were obtained as solutions in chloroform-*d* ($CDCl_3$) using a Varian nmrs 400 MHz spectrometer with tetramethylsilane as an internal standard. High-resolution mass spectra (HRMS) were obtained with an Agilent Micro TOF 11 spectrometer using the positive electrospray ionization (ESI) mode. HPLC was performed on an Agilent 1260 using a DIKMA Diamonsil, C18, 200 × 4.6 mm, 5 μm column, and UV detection at a wavelength of 210 nm. Column chromatography was performed on Biotage using a prepacked silica gel column, with detection at UV wavelengths 220 and 254 nm. Preparative HPLC was performed on Agilent 1260 using a Innoval ODS-2, 30 × 250 mm, 10 μm column, with detection at UV wavelengths 210 and 254 nm. 4-Aryl-2-hydroxytetralins and analogues were generally obtained as a mixture of *cis*- and *trans*- isomers (confirmed by ^1H NMR and NOE, HSQC and COSY). The ratio of the two isomers was inferred from ^1H NMR data. X-ray diffraction analysis of **21** was performed using a Bruker D8 VENTURE diffractometer.

2. Experimental procedures and characterization data

2.1 General procedure for the synthesis of 2-(bromoaryl)ethanols (11).

*Synthesis of 2-(2-bromophenyl)ethan-1-ol (**11a**)* [1]. *n*-Butyllithium (47.00 mmol, 2.90 equiv, 2.5 M in hexanes) was added dropwise into a solution of MTPPC (40.50 mmol, 2.50 equiv) in anhydr. THF (30 mL) at 0 ° C under an inert atmosphere of N_2. The resultant mixture was stirred for 10 min, then 2-bromobenzaldehyde (**9a**, 16.20 mmol, 1.00 equiv) in anhydr. THF (10 mL) was added dropwise to the reaction mixture. After complete addition, the reaction turned from a white suspension to a dark red clear solution. The reaction mixture was stirred further for 1 h at 0 °C, then quenched with saturated aqueous NaCl (30 mL), and extracted with ethyl acetate (2 × 30 mL). The combined organic layers were dried over Na_2SO_4, filtered, and the filtrate was concentrated under reduced pressure to afford the crude vinyl ether product **10a**, which was directly used in the next step.

To compound **10a** (16.20 mmol, 1.0 equiv) in THF (30 mL) was added 18% hydrochloric acid (16.20 mmol, 1.0 equiv) at room temperature. The reaction mixture was stirred under reflux under an inert atmosphere of N_2 for 1 h, concentrated under reduced pressure to remove THF, and methanol (30 mL) was added. After being stirred at room temperature for 10 min, KBH_4 (16.20 mmol, 1.00 equiv) was added slowly at 20 °C and the mixture was stirred for 1 h. Then, saturated aqueous NaCl (30 mL) was added, the mixture extracted with ethyl acetate (2 × 30 mL), and the combined organic layers were dried over Na_2SO_4 and concentrated under reduced pressure. The residue was purified by column chromatography on silica gel eluting with EA/PE (boiling point range 60–90 °C) (*v*/*v* = 1:9) to give the desired 2-(2-bromophenyl)ethan-1-ol (**11a**). Colorless oil. 2.41 g, 74%. IR (KBr): 3383, 3058, 2920, 2880, 1567, 1471, 1440, 1041, 749, 658 cm^{-1}. ^1H NMR ($CDCl_3$, 400 MHz) δ 7.54 (d, J = 7.9 Hz, 1H), 7.27–7.22 (m, 2H), 7.10–7.06 (m, 1H), 3.86 (t, J = 6.7 Hz, 2H), 3.01 (t, J = 6.7 Hz, 2H), 1.57 (s, 1H). ^{13}C NMR ($CDCl_3$, 101 MHz) δ 137.8, 132.9, 131.3, 128.2, 127.4, 124.7, 62.0, 39.3. HRMS (ESI) *m/z* $[M - H_2O + H]^+$ calcd for $C_8H_8Br^+$ 182.9804, found 182.9784.

2-(2-Bromoaryl)ethanols **11b**, **c**, **11e**, and **11h** were synthesized analogously as described for **11a**.

*2-(2-Bromo-4-methoxyphenyl)ethan-1-ol (**11b**)* [2]. Light yellow oil. 2.85 g, 76%. IR (KBr): 3396, 2939, 2880, 1636, 1605, 1567, 1500, 1458, 1283, 1241, 1039, 867, 743 cm^{-1}. ^1H NMR ($CDCl_3$, 400 MHz) δ 7.15 (d, J = 8.5 Hz 1H), 7.09 (d, J = 2.7 Hz, 1H), 6.79 (dd, J = 8.4, 2.7 Hz, 1H), 3.81 (t, J = 6.7 Hz, 2H), 3.75 (s, 3H), 2.93 (t, J = 6.8 Hz, 2H), 1.75 (s, 1H). ^{13}C NMR ($CDCl_3$, 101 MHz) δ 158.7, 131.5, 129.6, 124.7, 118.1, 113.6, 62.2, 55.5, 38.4. HRMS (ESI) *m/z* $[M - H_2O + H]^+$ calcd for $C_9H_{10}BrO^+$ 212.9910, found 212.9890.

*2-(2-Bromo-4-chlorophenyl)ethan-1-ol (**11c**)*. Light yellow oil. 2.79 g, 73%. IR (KBr): 3355, 2935, 2880, 1636, 1586, 1557, 1435, 1381, 1101, 913, 843, 743 cm^{-1}. ^1H NMR ($CDCl_3$, 400 MHz) δ 7.53 (d, J = 2.0 Hz, 1H), 7.24–7.16 (m, 2H), 3.80 (t, J = 6.7 Hz, 2H), 2.94 (t, J = 6.7 Hz, 2H), 2.08 (s, 1H). ^{13}C NMR ($CDCl_3$, 101 MHz) δ 136.4, 132.9, 132.4, 131.9, 127.6, 124.8, 61.7, 38.6. HRMS (ESI) *m/z* $[M - H_2O + H]^+$ calcd for $C_8H_7BrCl^+$ 216.9414, found 216.9387.

*2-(2-Bromophenyl)propan-1-ol (**11e**)* [2]. Colorless oil. 2.44 g, 70%. IR (KBr): 3383, 3063, 2964, 2874, 1654, 1508, 1472, 1438, 1038, 1022, 751 cm^{-1}. ^1H NMR ($CDCl_3$, 400 MHz) δ 7.55 (dd, J = 8.0, 1.2 Hz, 1H), 7.30–7.23 (m, 2H), 7.08–7.04 (m, 1H), 3.73–3.63 (m, 2H), 3.52–3.43 (m, 1H), 1.99 (s, 1H), 1.26 (d, J = 7.0 Hz, 3H). ^{13}C NMR ($CDCl_3$, 101 MHz) δ 142.6, 133.0, 127.9, 127.67, 127.65, 125.2, 67.1, 40.7, 17.1. HRMS (ESI) *m/z* [M − H_2O + H]$^+$ calcd for $C_9H_{10}Br^+$ 196.9960, found 196.9938.

*2-(1-Bromonaphthalen-2-yl)ethan-1-ol (**11h**).* Light yellow oil. 3.09 g, 76%. IR (KBr): 3374, 3052, 2934, 2878, 1647, 1534, 1352, 1255, 1040, 913, 812, 748 cm^{-1}. ^1H NMR ($CDCl_3$, 400 MHz) δ 8.31 (d, J = 8.5 Hz, 1H), 7.78 (d, J = 8.1 Hz, 1H), 7.73 (d, J = 8.3 Hz, 1H), 7.62–7.44 (m, 2H), 7.35 (d, J = 8.3 Hz, 1H), 3.94 (t, J = 6.8 Hz, 2H), 3.25 (t, J = 6.8 Hz, 2H), 1.93 (s, 1H). ^{13}C NMR ($CDCl_3$, 101 MHz) δ 136.1, 133.4, 132.6, 128.6, 128.0, 127.60, 127.4, 127.3, 126.1, 124.3, 62.3, 40.5. HRMS (ESI) *m/z* [M − H_2O + H]$^+$ calcd for $C_{12}H_{10}Br^+$ 232.9960, found 232.9962.

*Synthesis of 2-(2-bromo-4-nitrophenyl)ethan-1-ol (**11d**).* Aqueous HNO_3 (63%, 14.90 mmol, 1.00 equiv) was added dropwise into a solution of **3a** (14.90 mmol, 1.00 equiv) in acetic anhydride (10 mL) and H_2SO_4 (0.15 mmol, 0.01 equiv) at 0 °C under an inert atmosphere of N_2 and the mixture was stirred for 2 h. After the reaction was completed, the solution was poured into ice/water (30 mL) with stirring, and saturated aqueous $NaHCO_3$ was added dropwise with cooling in an ice-bath until free from acetic acid. Then, the mixture was extracted with ethyl acetate (3 × 30 mL), the combined organic layers were dried over Na_2SO_4 and filtered, and the filtrate was concentrated under reduced pressure. The crude product was directly used in the next step. Aqueous NaOH (30%, 14.90 mmol, 1.00 equiv) was added to the crude product in methanol (30 mL) and H_2O (30 mL) at room temperature. The mixture was stirred under reflux under an inert atmosphere of N_2 for 1 h. Then, the reaction mixture was concentrated under reduced pressure. The residue was extracted with ethyl acetate (2 × 30 mL), the combined organic layers were dried over Na_2SO_4 and filtered, and the filtrate was concentrated under reduced pressure. The residue was purified by column chromatography on silica gel eluting with EA/PE (boiling point range 60–90 °C) (*v*/*v* = 1:9) to give the desired 2-(2-bromo-4-nitrophenyl)ethan-1-ol (**11d**). Light yellow oil. 2.60 g, 71%. IR (KBr): 3383, 3058, 2920, 2880, 1567, 1471, 1440, 1041, 749, 658 cm^{-1}. ^1H NMR ($CDCl_3$, 400 MHz) δ 8.15 (d, J = 2.7 Hz, 1H), 7.92 (dd, J = 8.7, 2.8 Hz, 1H), 7.71 (d, J = 8.7 Hz, 1H), 3.93 (t, J = 6.5 Hz, 2H), 3.09 (t, J = 6.5 Hz, 2H), 1.75 (s, 1H). ^{13}C NMR ($CDCl_3$, 101 MHz) δ 147.1, 140.2, 133.7, 132.0, 125.7, 122.7, 61.2, 39.0. HRMS (ESI) *m/z* [M − H_2O + H]$^+$ calcd for $C_8H_7BrNO_2^+$ 227.9655, found 227.9627.

2.2 General procedure for the synthesis of 2-(vinylaryl)ethanols (12).

*Synthesis of 2-(2-vinylphenyl)ethan-1-ol (**12a**)* [3]. Compound **11a** (10.00 mmol, 1.00 equiv), pinacol vinylboronate (12.00 mmol, 1.20 equiv) and Pd(dppf)Cl_2 (1.00 mmol, 0.10 equiv) were added to dioxane (16 mL) in an inert atmosphere of N_2 at 20 °C. Then, K_2CO_3 (13.00 mmol, 1.30 equiv) in H_2O (4 mL) was added to above mixture and the mixture was heated at reflux for 2 h. Afterwards saturated aqueous NaCl (30 mL) was added, and the

mixture extracted with ethyl acetate (3 × 30 mL). The combined organic layers were dried over Na_2SO_4 and filtered, and the filtrate was concentrated under reduced pressure. The residue was purified by column chromatography on silica gel eluting with EA/PE (boiling point range 60–90 °C) (*v*/*v* = 1:9) to give the desired 2-(2-vinylphenyl)ethan-1-ol (**12a**). Light yellow oil. 1.16 g, 78%. IR (KBr): 3374, 2947, 2878, 1831, 1654, 1626, 1602, 1484, 1450, 1417, 1044, 914, 774 cm^{-1}. ^1H NMR ($CDCl_3$, 400 MHz) δ 7.52–7.49 (m, 1H), 7.24–7.16 (m, 3H), 7.01 (dd, J = 17.3, 11.0 Hz, 1H), 5.65 (dd, J = 17.4, 1.3 Hz, 1H), 5.31 (dd, J = 11.0, 1.3 Hz, 1H), 3.80 (t, J = 6.9 Hz, 2H), 2.95 (t, J = 6.9 Hz, 2H), 1.64 (s, 1H). ^{13}C NMR ($CDCl_3$, 101 MHz) δ 137.1, 135.6, 134.4, 130.3, 127.9, 126.9, 126.0, 116.0, 63.1, 36.4. HRMS (ESI) *m/z* [M − H_2O + H]$^+$ calcd for $C_{10}H_{11}^+$ 131.0855, found 131.0873.

2-(Vinylaryl)alkanols **12b**–**h** were synthesized analogously as described for **12a**.

*2-(4-Methoxy-2-vinylphenyl)ethan-1-ol (**12b**).* Light yellow oil. 1.35 g, 76%. IR (KBr): 3374, 2943, 2876, 1624, 1605, 1570, 1500, 1465, 1424, 1288, 1243, 1041, 913, 743 cm^{-1}. ^1H NMR ($CDCl_3$, 400 MHz) δ 7.08 (d, J = 8.4 Hz, 1H), 7.03 (d, J = 2.7 Hz, 1H), 6.96 (dd, J = 17.3, 10.9 Hz, 1H), 6.77 (dd, J = 8.4, 2.7 Hz, 1H), 5.63 (dd, J = 17.3, 1.3 Hz, 1H), 5.30 (dd, J = 10.9, 1.3 Hz, 1H), 3.79 (s, 3H), 3.74 (t, J = 6.9 Hz, 2H), 2.88 (t, J = 6.9 Hz, 2H), 1.74 (s, 1H). ^{13}C NMR ($CDCl_3$, 101 MHz) δ 158.4, 138.0, 134.4, 131.4, 128.0, 116.0, 113.6, 111.1, 63.3, 55.3, 35.6. HRMS (ESI) m/z [M − H_2O + H]$^+$ calcd for $C_{11}H_{13}O^+$ 161.0961, found 161.0931.

*2-(4-Chloro-2-vinylphenyl)ethan-1-ol (**12c**).* Light yellow oil. 1.30 g, 71%. IR (KBr): 3384, 2947, 2879, 1653, 1625, 1521, 1507, 1479, 1458, 1418, 1041, 914, 743 cm^{-1}. ^1H NMR ($CDCl_3$, 400 MHz) δ 7.46 (d, J = 2.3 Hz, 1H), 7.20–7.07 (m, 2H), 6.91 (dd, J = 17.3, 11.0 Hz, 1H), 5.64 (dd, J = 17.3, 1.3 Hz, 1H), 5.34 (dd, J = 10.9, 1.3 Hz, 1H), 3.77 (t, J = 6.8 Hz, 2H), 2.90 (t, J = 6.8 Hz, 2H), 1.58 (s, 1H). ^{13}C NMR ($CDCl_3$, 101 MHz) δ 138.7, 134.1, 133.3, 132.7, 131.6, 127.7, 125.9, 117.2, 62.9, 35.8. HRMS (ESI) *m/z* [M − H_2O + H]$^+$ calcd for $C_{10}H_{10}Cl^+$ 165.0466, found 165.0496.

*2-(4-Nitro-2-vinylphenyl)ethan-1-ol (**12d**).* Red oil. 1.35 g, 70%. IR (KBr): 3421, 2922, 1608, 1569, 1463, 14209, 1037, 912, 741 cm^{-1}. ^1H NMR ($CDCl_3$, 400 MHz) δ 8.11–7.98 (m, 2H), 7.60 (d, J = 8.5 Hz, 1H), 7.01 (dd, J = 17.4, 11.1 Hz, 1H), 5.78 (dd, J = 17.3, 1.3 Hz, 1H), 5.51 (d, J = 11.0, 1.3 Hz, 1H), 3.86 (t, J = 6.7 Hz, 2H), 3.01 (t, J = 6.7 Hz, 2H), 1.84 (s, 1H). ^{13}C NMR ($CDCl_3$, 101 MHz) δ 147.0, 143.6, 137.5, 132.9, 126.8, 125.1, 121.9, 119.9, 62.4, 36.0. HRMS (ESI) *m/z* [M − H_2O + H]$^+$ calcd for $C_{10}H_{10}NO_2^+$ 176.0706, found 176.0688.

*2-(2-Vinylphenyl)propan-1-ol (**12e**).* Yellow oil. 1.18 g, 73%. IR (KBr): 3386, 3062, 2926, 1625, 1600, 1507, 1450, 1384, 1036, 1011, 913, 773 cm^{-1}. ^1H NMR ($CDCl_3$, 400 MHz) δ 7.47 (d, J = 7.5 Hz, 1H), 7.31–7.18 (m, 3H), 7.09 (dd, J = 17.3, 10.9 Hz, 1H), 5.60 (dd, J = 17.1, 1.3 Hz, 1H), 5.32 (dd, J = 10.8, 1.3 Hz, 1H), 3.79–3.62 (m, 2H), 3.39–3.30 (m, 1H), 1.69 (s, 1H), 1.26 (d, J = 6.9 Hz, 3H). ^{13}C NMR ($CDCl_3$, 101 MHz) δ 140.8, 137.5, 134.8, 128.1, 126.62, 126.56, 125.7, 116.5, 68.1, 37.0, 17.5. HRMS (ESI) *m/z* [M − H_2O + H]$^+$ calcd for $C_{11}H_{13}^+$ 145.1012, found 145.0990.

*2-(2-(Prop-1-en-2-yl)phenyl)ethan-1-ol (**12f**)* [4]. Red oil. 1.15 g, 71%. IR (KBr): 3363, 3075, 2964, 1638, 1600, 1522, 1446, 1371, 1045, 901, 766 cm^{-1}. ^1H NMR ($CDCl_3$, 400 MHz)

δ 7.25–7.15 (m, 3H), 7.15–7.10 (m, 1H), 5.20–5.19 (m, 1H), 4.85–4.84 (m, 1H), 3.81 (t, *J* = 7.0 Hz, 2H), 2.91 (t, *J* = 7.0 Hz, 2H), 2.05–2.04 (m, 3H), 1.60 (s, 1H). ^{13}C NMR ($CDCl_3$, 101 MHz) δ 145.4, 144.3, 134.7, 129.7, 128.4, 127.0, 126.3, 115.2, 63.8, 36.2, 25.3. HRMS (ESI) *m/z* [M + Na]$^+$ calcd for $C_{11}H_{14}NaO^+$ 185.0937, found 185.0962.

*2-(2-(1-Phenylvinyl)phenyl)ethan-1-ol (**12g**).* Yellow oil. 1.64 g, 73%. IR (KBr): 3446, 3050, 1630, 1590, 1420, 1375, 1040, 960, 817 cm^{-1}. ^{1}H NMR ($CDCl_3$, 400 MHz) δ 7.33–7.26 (m, 9H), 5.82 (d, *J* = 1.1 Hz, 1H), 5.24 (d, *J* = 1.1 Hz, 1H), 3.65 (t, *J* = 6.9 Hz, 1H), 2.68 (t, *J* = 6.9 Hz, 1H). ^{13}C NMR ($CDCl_3$, 101 MHz) δ 149.0, 136.3, 130.7, 130.0, 128.4, 127.82, 127.76, 126.49, 126.45, 115.4, 63.2, 36.7. HRMS (ESI) *m/z* [M − H_2O + H]$^+$ calcd for $C_{16}H_{15}^+$ 207.1168, found 207.1160.

*2-(1-Vinylnaphthalen-2-yl)ethan-1-ol (**12h**).* Red oil. 1.51 g, 76%. IR (KBr): 3447, 3052, 2936, 1629, 1594, 1507, 1416, 1376, 1040, 960, 817, 750 cm^{-1}. ^{1}H NMR ($CDCl_3$, 400 MHz) δ 8.14–8.12 (m, 1H), 7.86–7.69 (m, 2H), 7.52–7.39 (m, 2H), 7.36 (d, *J* = 8.4 Hz, 1H), 7.07 (dd, *J* = 17.9, 11.4 Hz, 1H), 5.79 (dd, *J* = 11.5, 2.1 Hz, 1H), 5.45 (dd, *J* = 17.9, 2.1 Hz, 1H), 3.87 (t, *J* = 7.0 Hz, 2H), 3.10 (t, *J* = 6.9 Hz, 2H), 1.62 (s, 1H). ^{13}C NMR ($CDCl_3$, 101 MHz) δ 135.6, 133.9, 132.8, 132.4, 131.9, 128.02, 127.95, 127.3, 126.0, 125.7, 125.3, 121.7, 63.3, 37.0. HRMS (ESI) *m/z* [M + Na]$^+$ calcd for $C_{14}H_{14}NaO^+$ 221.0937, found 221.0948.

2.3 General procedure for the synthesis of 2-(2-vinylaryl)acetaldehydes (13).

*Synthesis of 2-(2-vinylphenyl)acetaldehyde (**13a**)* [5]. Dess–Martin periodinane (8.10 mmol, 1.20 equiv) was added in portions into a solution of 2-(2-vinylphenyl)ethan-1-ol (**12a**, 6.80 mmol, 1.00 equiv) in CH_2Cl_2 (20 mL) at 0 °C under an inert atmosphere of N_2. The resultant reaction mixture was stirred for further 2 h at 20 °C, then quenched with 8% aqueous $NaHCO_3$ (20 mL). The mixture was filtered and the cake was washed with CH_2Cl_2 (10 mL). The filtrate was separated and the aqueous layer was extracted with CH_2Cl_2 (2 × 10 mL). The combined organic layers were dried over Na_2SO_4 and filtered, and the filtrate was concentrated under reduced pressure. The residue was purified by column chromatography on silica gel eluting with EA/PE (boiling point range 60–90 °C) (*v*/*v* = 1:19) to give the desired 2-(2-vinylphenyl)acetaldehyde (**13a**). Colorless oil. 0.84 g, 85%. IR (KBr): 3427, 3064, 2824, 2726, 1728, 1626, 1485, 1450, 1416, 1318, 1037, 990, 919, 773 cm^{-1}. ^{1}H NMR ($CDCl_3$, 400 MHz) δ 9.68 (t, *J* = 2.3 Hz, 1H), 7.58–7.50 (m, 1H), 7.35–7.22 (m, 2H), 7.20–7.13 (m, 1H), 6.83 (dd, *J* = 17.3, 11.0 Hz, 1H), 5.66 (dd, *J* = 17.3, 1.2 Hz, 1H), 5.34 (dd, *J* = 10.9, 0.9 Hz, 1H), 3.76 (d, *J* = 2.3 Hz, 2H). ^{13}C NMR ($CDCl_3$, 101 MHz) δ 199.2, 137.8, 133.9, 130.9, 129.5, 128.2, 128.0, 126.4, 117.2, 48.4. HRMS (ESI) *m/z* [M − H_2O + H]$^+$ calcd for $C_{10}H_9^+$ 129.0699, found 129.0711.

2-(Vinylaryl)aldehydes 13b–h were synthesized analogously as described for 13a.

*2-(4-Methoxy-2-vinylphenyl)acetaldehyde (**13b**).* Colorless oil. 1.02 g, 85%. IR (KBr): 3446, 2836, 1720, 1624, 1493, 1420, 1290, 1199, 1029, 913, 743 cm^{-1}. ^{1}H NMR ($CDCl_3$, 400 MHz) δ 9.64 (s, 1H), 7.11–7.03 (m, 2H), 6.85–6.72 (m, 2H), 5.64 (d, *J* = 17.2 Hz, 1H), 5.33 (d, *J* = 10.9 Hz, 1H), 3.81 (s, 3H), 3.68 (s, 2H). ^{13}C NMR ($CDCl_3$, 101 MHz) δ 199.5, 159.2, 138.9,

134.0, 132.0, 121.7, 117.2, 113.9, 111.6, 55.3, 47.6. HRMS (ESI) *m/z* $[M - H_2O + H]^+$ calcd for $C_{11}H_{11}O^+$ 159.0804, found 159.0774.

*2-(4-Chloro-2-vinylphenyl)acetaldehyde (**13c**)*. Yellow oil. 1.01 g, 82%. IR (KBr): 3445, 2828, 1723, 1627, 1479, 1417, 1215, 1116, 1040, 913, 743 cm^{-1}. ^1H NMR ($CDCl_3$, 400 MHz) δ 9.65 (t, J = 2.1 Hz, 1H), 7.50 (d, J = 2.2 Hz, 1H), 7.26–7.19 (m, 1H), 7.08 (d, J = 8.2 Hz, 1H), 6.72 (dd, J = 17.2, 10.9 Hz, 1H), 5.66 (d, J = 17.2 Hz, 1H), 5.38 (d, J = 10.9 Hz, 1H), 3.72 (d, J = 2.0 Hz, 2H). ^{13}C NMR ($CDCl_3$, 101 MHz) δ 198.5, 139.4, 133.9, 132.9, 132.1, 128.1, 127.9, 126.4, 118.5, 47.7. HRMS (ESI) *m/z* $[M - H_2O + H]^+$ calcd for $C_{10}H_8Cl^+$ 163.0309, found 163.0280.

*2-(4-Nitro-2-vinylphenyl)acetaldehyde (**13d**).* Yellow oil. 0.98 g, 75%. IR (KBr): 3448, 2919, 1718, 1653, 1499, 1465, 1383, 1241, 1049, 913, 743 cm^{-1}. ^1H NMR ($CDCl_3$, 400 MHz) δ 9.74 (t, J = 1.6 Hz, 1H), 8.12 (dd, J = 8.6, 2.4 Hz, 1H), 8.04 (d, J = 2.4 Hz, 1H), 7.66 (d, J = 8.6 Hz, 1H), 6.78 (dd, J = 17.2, 11.0 Hz, 1H), 5.80 (dd, J = 17.3, 0.6 Hz, 1H), 5.54 (dd, J = 11.0, 0.6 Hz, 1H), 3.90 (d, J = 1.6 Hz, 2H). ^{13}C NMR ($CDCl_3$, 101 MHz) δ 197.2, 147.1, 144.2, 132.4, 131.2, 127.2, 125.9, 123.0, 121.1, 48.0. HRMS (ESI) *m/z* $[M - H_2O + H]^+$ calcd for $C_{10}H_8NO_2^+$ 174.0550, found 174.0521.

*2-(2-Vinylphenyl)propanal (**13e**).* Yellow oil. 0.85 g, 78%. IR (KBr): 3405, 2948, 2836, 1654, 1452, 1413, 1383, 1021, 669 cm^{-1}. ^1H NMR ($CDCl_3$, 400 MHz) δ 9.67 (s, 1H), 7.57–7.50 (m, 1H), 7.33–7.26 (m, 2H), 7.10–7.03 (m, 1H), 6.95 (dd, J = 17.2, 10.9 Hz, 1H), 5.64 (dd, J = 17.2, 1.4 Hz, 1H), 5.37 (dd, J = 10.9, 1.4 Hz, 1H), 3.92 (q, J = 7.0 Hz, 1H), 1.41 (d, J = 7.0 Hz, 3H). ^{13}C NMR ($CDCl_3$, 101 MHz) δ 201.1, 137.7, 135.3, 134.1, 128.4, 127.9, 127.7, 127.0, 117.7, 49.2, 14.6. HRMS (ESI) *m/z* $[M - H_2O + H]^+$ calcd for $C_{11}H_{11}^+$ 143.0855, found 143.0829.

*2-(2-(Prop-1-en-2-yl)phenyl)acetaldehyde (**13f**)* [6]. Yellow oil. 0.77 g, 70%. IR (KBr): 3447, 2970, 1724, 1654, 1541, 1436, 1384, 1047, 905, 766 cm^{-1}. ^1H NMR ($CDCl_3$, 400 MHz) δ 9.70 (t, J = 2.1 Hz, 1H), 7.30–7.24 (m, 2H), 7.22–7.17 (m, 2H), 5.23–5.21 (m, 1H), 4.81–4.80 (m, 1H), 3.73 (d, J = 2.2 Hz, 2H), 2.01–2.00 (m, 3H). ^{13}C NMR ($CDCl_3$, 101 MHz) δ 200.0, 144.7, 140.1, 134.2, 129.8, 128.5, 125.7, 124.1, 116.1, 48.2, 25.0. HRMS (ESI) *m/z* $[M - H_2O + H]^+$ calcd for $C_{11}H_{11}^+$ 143.0855, found 143.0825.

*2-(2-(1-Phenylvinyl)phenyl)acetaldehyde (**13g**).* Colorless oil. 1.21 g, 80%. IR (KBr): 3425, 3050, 1918, 1770, 1628, 1508, 1415, 1261, 1180, 1035, 994 cm^{-1}. ^1H NMR ($CDCl_3$, 400 MHz) δ 9.46 (t, J = 2.2 Hz, 1H), 7.93–6.96 (m, 9H), 5.82 (d, J = 1.1 Hz, 1H), 5.24 (d, J = 1.1 Hz, 1H), 3.48 (d, J = 2.1 Hz, 2H). ^{13}C NMR ($CDCl_3$, 101 MHz) δ 199.4, 148.4, 142.3, 140.1, 130.77, 130.72, 130.6, 128.6, 128.2, 128.11, 128.09, 127.6, 126.56, 126.55, 116.2, 48.28, 48.27. HRMS (ESI) *m/z* $[M - H_2O + H]^+$ calcd for $C_{16}H_{13}^+$ 205.1012, found 205.1010.

*2-(1-Vinylnaphthalen-2-yl)acetaldehyde (**13h**).* Colorless oil. 1.04 g, 78%. IR (KBr): 3425, 3053, 2823, 2724, 1918, 1773, 1628, 1594, 1508, 1416, 1384, 1261, 1180, 1035, 994, 817, 748 cm^{-1}. ^1H NMR ($CDCl_3$, 400 MHz) δ 9.76 (t, J = 2.2 Hz, 1H), 8.12–8.07 (m, 1H), 7.87–7.75 (m, 2H), 7.56–7.44 (m, 2H), 7.30 (d, J = 8.4 Hz, 1H), 7.03 (dd, J = 18.0, 11.4 Hz, 1H), 5.80 (dd, J = 11.5, 1.9 Hz, 1H), 5.40 (dd, J = 17.8, 1.8 Hz, 1H), 3.94 (d, J = 2.0 Hz, 2H). ^{13}C NMR ($CDCl_3$, 101 MHz) δ 199.7, 136.6, 133.8, 132.76, 132.0, 128.2, 128.1, 127.8, 126.8, 126.3,

125.9, 125.6, 122.5, 49.1. HRMS (ESI) *m/z* [M – H_2O + H]$^+$ calcd for $C_{14}H_{11}^+$ 179.0855, found 179.0839.

2.4 General procedure for the synthesis of 4-aryl-2-hydroxytetralins (14).

*Synthesis of 4-(3,4-dimethoxyphenyl)-1,2,3,4-tetrahydronaphthalen-2-ol (**14aa**)*. Boron trifluoride etherate (48%, 1.54 mmol, 1.10 equiv) was added dropwise into the mixture of aldehyde **13a** (1.40 mmol, 1.00 equiv) and veratrole (1.47 mmol, 1.05 equiv) dissolved in anhydr. CH_2Cl_2 (6 mL) at 0 °C in an inert atmosphere of N_2. The resultant solution was stirred for further 2 h at 0 °C, then quenched with saturated aqueous NaCl (10 mL), and extracted with CH_2Cl_2 (2 × 10 mL). The combined organic layers were dried over Na_2SO_4 and filtered, and the filtrate was concentrated under reduced pressure. The residue was purified by column chromatography on silica gel eluting with EA/PE (boiling point range 60–90 °C) (*v/v* = 1:4) to give **14aa**. Colorless oil. 0.28 g, 70%, *cis*/*trans* = 49:51. IR (KBr): 3448, 3058, 2933, 2836, 1605, 1591, 1515, 1464, 1450, 1418, 1262, 1140, 1115, 1028, 811, 740 cm^{-1}. ^{1}H NMR ($CDCl_3$, 400 MHz) *δ* 7.16–7.11 (m, 4H), 7.09–7.00 (m, 2H), 6.92–6.90 (m, 1H), 6.82–6.73 (m, 4H), 6.65 (d, *J* = 2.0 Hz, 1H), 6.63 (d, *J* = 2.0 Hz, 1H), 6.55 (dd, *J* = 8.2, 2.1 Hz, 1H), 4.32–4.29 (m, 1H, *trans*-), 4.28–4.23 (m, 1H, *trans*-), 4.20–4.12 (m, 1H, *cis*-), 4.08–4.04 (m, 1H, *cis*-), 3.86 (s, 3H), 3.83 (s, 3H), 3.79 (s, 3H), 3.78 (s, 3H), 3.24–3.14 (m, 2H), 2.92–2.80 (m, 2H), 2.39–2.34 (m, 1H), 2.23–2.16 (m, 1H), 2.09–2.04 (m, 1H), 1.89 (q, *J* = 12.2 Hz, 1H). ^{13}C NMR ($CDCl_3$, 101 MHz) *δ* 149.0, 148.8, 147.6, 147.4, 139.4, 139.1, 138.4, 138.3, 134.9, 134.5, 129.7, 129.4, 129.2, 129.1, 126.34, 126.25, 126.1, 120.8, 112.0, 111.7, 111.2, 111.0, 67.7, 64.6, 55.9, 55.8, 45.9, 43.1, 42.3, 40.3, 39.5, 38.3. HRMS (ESI) *m/z* [M + Na]$^+$ calcd for $C_{18}H_{20}NaO_3^+$ 307.1305, found 307.1319.

4-Aryl-2-hydroxy tetralins **14ab-hb** and **15** were synthesized analogously as described for **14aa**.

*4-(2,4-Dimethoxyphenyl)-1,2,3,4-tetrahydronaphthalen-2-ol (**14ab**).* Colorless oil. 0.29 g, 72%, *cis*/*trans* = 55:45. IR (KBr): 3446, 3058, 2933, 2361, 1611, 1507, 1458, 1291, 1207, 1156, 1037 cm^{-1}. ^{1}H NMR ($CDCl_3$, 400 MHz) *δ* 7.14–7.09 (m, 4H), 7.08–6.99 (m, 3H), 6.92–6.89 (m, 2H), 6.78–6.76 (m, 1H), 6.50–6.47 (m, 2H), 6.43 (dd, *J* = 8.4, 2.5 Hz, 1H), 6.30 (dd, *J* = 8.2, 2.4 Hz, 1H), 4.67 (t, *J* = 5.7 Hz, 1H, *trans*-), 4.53 (dd, *J* = 11.6, 5.8 Hz, 1H, *cis*-), 4.21–4.13 (m, 2H), 3.83 (s, 3H), 3.80 (s, 3H), 3.76 (s, 6H), 3.23–3.12 (m, 2H), 2.92–2.77 (m, 2H), 2.35–2.29 (m, 1H), 2.15–2.03 (m, 2H), 1.89 (q, *J* = 11.6 Hz, 1H). ^{13}C NMR ($CDCl_3$, 101 MHz) *δ* 159.3, 159.1, 158.2, 157.7, 139.4, 138.4, 135.2, 135.0, 130.4, 129.9, 129.7, 129.2, 129.1, 128.6, 127.5, 126.7, 126.1, 126.0, 125.8, 104.6, 103.6, 98.6, 98.4, 67.9, 64.9, 55.5, 55.4, 55.31, 55.28, 41.1, 39.6, 38.6, 38.2, 38.1, 36.5. HRMS (ESI) *m/z* [M + H]$^+$ calcd for $C_{18}H_{21}O_3^+$ 285.1485, found 285.1488.

*4-(4-Methoxy-3,5-dimethylphenyl)-1,2,3,4-tetrahydronaphthalen-2-ol (**14ac**).* Colorless oil. 0.24 g, 60%, *cis*/*trans* = 54:46. IR (KBr): 3424, 3059, 2925, 1654, 1601, 1487, 1450, 1265, 1220, 1147, 1054, 739 cm^{-1}. ^{1}H NMR ($CDCl_3$, 400 MHz) *δ* 7.15–7.09 (m, 4H), 7.08–7.01 (m, 3H), 6.91 (d, *J* = 7.6 Hz, 1H), 6.80 (s, 2H), 6.68 (s, 2H), 4.29–4.26 (m, 2H, *trans*-), 4.19–4.12

(m, 1H, *cis*-), 4.01 (dd, *J* = 12.1, 5.6 Hz, 1H, *cis*-), 3.72 (s, 3H), 3.70 (s, 3H), 3.24–3.14 (m, 2H), 2.92–2.80 (m, 2H), 2.37–2.31 (m, 1H), 2.24 (s, 6H), 2.19 (s, 6H), 2.18–2.14 (m, 1H), 2.08–2.02 (m, 1H), 1.87 (q, *J* = 12.0 Hz, 1H). ^{13}C NMR ($CDCl_3$, 101 MHz) *δ* 155.4, 155.2, 141.9, 141.1, 139.2, 138.3, 134.9, 134.4, 130.8, 130.5, 129.9, 129.4, 129.3, 129.1, 129.0, 128.9, 126.3, 126.14, 126.12, 67.8, 64.6, 59.68, 59.65, 45.7, 43.2, 42.2, 40.3, 39.6, 38.3, 16.1. HRMS (ESI) *m/z* [M – H_2O + H]$^+$ calcd for $C_{19}H_{21}O^+$ 265.1587, found 265.1579.

*4-(2,5-Dimethoxyphenyl)-1,2,3,4-tetrahydronaphthalen-2-ol (**14ad**).* Colorless oil. 0.27 g, 68%, *cis*/*trans* = 51:49. IR (KBr): 3419, 2927, 2360, 1652, 1495, 1463, 1278, 1214, 1048, 745 cm^{-1}. ^1H NMR ($CDCl_3$, 400 MHz) *δ* 7.14–7.00 (m, 6H), 6.91–6.67 (m, 6H), 6.59 (d, *J* = 3.1 Hz, 1H), 6.21 (d, *J* = 3.2 Hz, 1H), 4.73 (t, *J* = 5.9 Hz, 1H, *trans*-), 4.59 (dd, *J* = 11.7, 5.9 Hz, 1H, *cis*-), 4.21–4.13 (m, 2H), 3.80 (s, 3H), 3.73 (s, 3H), 3.69 (s, 3H), 3.63 (s, 3H), 3.22–3.11 (m, 2H), 2.92–2.77 (m, 2H), 2.38–2.32 (m, 1H), 2.16–2.04 (m, 2H), 1.90 (q, *J* = 11.6 Hz, 1H). ^{13}C NMR ($CDCl_3$, 101 MHz) δ 153.8, 153.2, 151.7, 151.3, 138.82, 137.84, 136.4, 135.6, 135.2, 135.1, 129.7, 129.33, 129.1, 128.7, 126.2, 126.1, 125.9, 117.1, 115.7, 112.1, 111.7, 111.2, 110.6, 67.8, 64.8, 56.3, 55.98, 55.6, 55.5, 40.9, 39.51, 39.1, 38.5, 37.8, 37.1. HRMS (ESI) *m/z* [M + H]$^+$ calcd for $C_{18}H_{21}O_3^+$ 285.1485, found 285.1485.

*4-(2-Methoxy-5-methylphenyl)-1,2,3,4-tetrahydronaphthalen-2-ol (**14ae**).* Yellow oil. 0.24 g, 65%, *cis*/*trans* = 60:40. IR (KBr): 3422, 2920, 2362, 1735, 1653, 1499, 1458, 1243, 1037, 746 cm^{-1}. ^1H NMR ($CDCl_3$, 400 MHz) *δ* 7.17–7.17 (m, 4H), 7.05–7.01 (m, 3H), 6.98 (dd, *J* = 8.3, 2.3 Hz, 1H), 6.91 (d, *J* = 7.6 Hz, 1H), 6.85–6.77 (m, 4H), 6.45 (d, *J* = 2.2 Hz, 1H), 4.75 (t, *J* = 6.1 Hz, 1H, *trans*-), 4.61 (dd, *J* = 11.8, 5.8 Hz, 1H, *cis*-), 4.21–4.18 (m, 2H), 3.82 (s, 3H), 3.76 (s, 3H), 3.25–3.13 (m, 2H), 2.95–2.80 (m, 2H), 2.37–2.31 (m, 1H), 2.28 (s, 3H), 2.16 (s, 3H), 2.16–2.10 (m, 2H), 1.92 (q, *J* = 11.7 Hz, 1H). ^{13}C NMR ($CDCl_3$, 101 MHz) *δ* 155.2, 154.9, 139.3, 138.3, 135.1, 135.1, 134.8, 134.0, 130.6, 130.2, 130.1, 129.7, 129.4, 129.3, 129.1, 128.7, 111.0, 110.4, 67.9, 64.9, 55.7, 55.6, 41.0, 39.6, 38.7, 38.0, 36.6, 20.59, 20.56. HRMS (ESI) *m/z* [M – H_2O + H]$^+$ calcd for $C_{18}H_{19}O^+$ 251.1430, found 251.1429.

*4-(Furan-2-yl)-1,2,3,4-tetrahydronaphthalen-2-ol (**14af**).* Yellow oil. 0.12 g, 40%, *cis*/*trans* = 1:99. IR (KBr): 3422, 2924, 2360, 1700, 1653, 1490, 1450, 1384, 1053, 739 cm^{-1}. ^1H NMR ($CDCl_3$, 400 MHz) *δ* 7.32 (dd, *J* = 1.8, 0.8 Hz, 1H), 7.19–7.08 (m, 4H), 6.25 (dd, *J* = 3.2, 1.9 Hz, 1H), 5.81 (dt, *J* = 3.2, 0.8 Hz, 1H), 4.40 (t, *J* = 5.7 Hz, 1H), 4.28–4.21 (m, 1H), 3.17 (dd, *J* = 16.4, 5.0 Hz, 1H), 2.79 (dd, *J* = 16.4, 5.0 Hz, 1H), 2.39–2.33 (m, 1H), 2.11–2.04 (m, 1H). ^{13}C NMR ($CDCl_3$, 101 MHz) *δ* 158.4, 141.4, 135.3, 134.4, 129.6, 129.3, 126.8, 126.1, 110.0, 106.6, 64.7, 38.3, 37.0, 36.5. HRMS (ESI) *m/z* [M + H]$^+$ calcd for $C_{14}H_{15}O_2^+$ 215.1067, found 215.1066.

*4-(5-Methylfuran-2-yl)-1,2,3,4-tetrahydronaphthalen-2-ol (**14ag**).* Yellow oil. 0.11 g, 35%, *cis*/*trans* = 1:99. IR (KBr): 3447, 2923, 1653, 1457, 1384, 1055, 743 cm^{-1}. ^1H NMR ($CDCl_3$, 400 MHz) *δ* 7.16–7.10 (m, 4H), 5.80 (dd, *J* = 2.9, 1.1 Hz, 1H), 5.62 (d, *J* = 3.0 Hz, 1H), 4.32 (t, *J* = 5.6 Hz, 1H), 4.28–4.22 (m, 1H), 3.17 (dd, *J* = 16.3, 5.2 Hz, 1H), 2.78 (dd, *J* = 16.4, 7.8 Hz, 1H), 2.38–2.33 (m, 1H), 2.24 (s, 3H), 2.08–2.01 (m, 1H). ^{13}C NMR ($CDCl_3$, 101 MHz) *δ* 156.5, 150.9, 135.5, 134.4, 129.5, 129.4, 126.6, 126.0, 107.3, 105.8, 64.8, 38.4, 37.3, 36.6, 13.6. HRMS (ESI) *m/z* [M – H_2O + H]$^+$ calcd for $C_{15}H_{15}O^+$ 211.1117, found 211.1116.

*4-(Thiophen-2-yl)-1,2,3,4-tetrahydronaphthalen-2-ol (**14ah**).* Yellow oil. 0.12 g, 37%, *cis*/*trans* = 41:59. IR (KBr): 3421, 2917, 2849, 1653, 1449, 1384, 1039, 743, 698 cm^{-1}. ^{1}H NMR ($CDCl_3$, 400 MHz) δ 7.20–7.05 (m, 10H), 6.98–6.95 (m, 1H), 6.92–6.88 (m, 2H), 6.67–6.66 (m, 1H), 4.63 (t, J = 5.8 Hz, 1H, *trans*-), 4.48 (dd, J = 11.8, 5.4 Hz, 1H, *cis*-), 4.32–4.26 (m, 1H), 4.20–4.13 (m, 1H), 3.23–3.14 (m, 2H), 2.92–2.78 (m, 2H), 2.53–2.47 (m, 1H), 2.23 (t, J = 6.0 Hz, 2H), 2.05–1.96 (m, 1H). ^{13}C NMR ($CDCl_3$, 101 MHz) δ 150.4, 149.1, 138.2, 137.5, 134.2, 134.1, 129.6, 129.5, 129.2, 128.7, 126.8, 126.7, 126.5, 126.3, 126.2, 125.4, 125.3, 124.0, 123.8, 67.5, 64.5, 43.5, 40.9, 40.7, 39.3, 38.6, 38.3. HRMS (ESI) *m/z* [M – H_2O + H]$^+$ calcd for $C_{14}H_{13}S^+$ 213.0732, found 213.0734.

*4-Allyl-1,2,3,4-tetrahydronaphthalen-2-ol (**14ai**).* Tetraallylsilane was used as the nucleophile to give **14ai** as a yellow oil. 0.17 g, 65%, *cis*/*trans* = 44:56. IR (KBr): 3373, 2924, 2360, 1653, 1507, 1489, 1451, 1364, 1118, 1039, 914, 746 cm^{-1}. ^{1}H NMR ($CDCl_3$, 400 MHz) δ 7.31–7.07 (m, 8H), 5.88–5.71 (m, 2H), 5.12–5.04 (m, 4H), 4.27–4.21 (m, 1H, *trans*-), 4.08–4.00 (m, 1H, *cis*-), 3.14–3.00 (m, 3H), 2.77–2.65 (m, 3H), 2.52–2.27 (m, 4H), 2.23–2.17 (m, 1H), 2.02–1.97 (m, 1H), 1.87–1.80 (m, 1H), 1.54–1.45 (m, 1H). ^{13}C NMR ($CDCl_3$, 101 MHz) δ 139.2, 138.6, 136.9, 136.2, 135.1, 134.1, 129.5, 129.4, 128.2, 126.8, 126.3, 126.12, 126.06, 126.0, 117.0, 116.7, 67.7, 64.5, 41.8, 40.3, 39.7, 38.8, 38.6, 37.2, 36.6, 35.4. HRMS (ESI) *m/z* [M – H_2O + H]$^+$ calcd for $C_{13}H_{15}^+$ 171.1168, found 171.1175.

*1,2,3,4-Tetrahydro-[1,1'-binaphthalene]-3,4'-diol (**14aj**).* The product **14aj** was synthesized analogously as described for **14aa**. After purification by column chromatography on silica gel, the obtained **14aj** was further purified by preparative HPLC. The pre-HPLC method is summarized in Table S1. Yellow oil. 0.12 g, 30%, *cis*/*trans* = 99:1. IR (KBr): 3380, 2924, 2360, 1650, 1500, 1450, 1364, 1118 cm^{-1}. ^{1}H NMR ($CDCl_3$, 400 MHz) δ 8.28–8.26 (m, 1H), 7.91–7.83 (m, 1H), 7.49–7.43 (m, 2H), 7.20–7.09 (m, 3H), 6.98 (t, J = 7.4 Hz, 1H), 6.79–6.75 (m, 2H), 4.85–4.81 (m, 1H), 4.35–4.28 (m, 1H), 3.29–3.24 (m, 1H), 3.05–2.99 (m, 1H), 2.50–2.45 (m, 1H), 2.20–2.11 (m, 1H). ^{13}C NMR ($CDCl_3$, 101 MHz) δ 150.5, 139.3, 134.7, 129.3, 128.9, 126.7, 126.40, 126.37, 126.2, 124.8, 122.5, 108.4, 68.1, 41.7, 39.6, 29.7. HRMS (ESI) *m/z* [M – H_2O + H]$^+$ calcd for $C_{20}H_{17}O^+$ 273.1274, found 273.1284.

Table S1: Preparative HPLC method for **14aj.**

Instrument	Agilent 1260		
column	Innoval ODS-2, 30 × 250 mm, 10 μm		
mobile phase gradient program：	time (min)	A%: 0.1%TFA aq.	B%: MeCN
	0.0	55	45
	3.0	45	55
	33.0	25	75
	35.0	5	95
	38.0	5	95
	40.0	55	45
	44.0	55	45

stop time	44 min
post time	OFF
flow rate	10 mL/min
UV detector	Detection: 210 nm<br>Monitor: 254 nm
conc.	50 mg/mL

*4-(3,4-Dimethoxyphenyl)-6-methoxy-1,2,3,4-tetrahydronaphthalen-2-ol (**14ba**).* Yellow oil. 0.32 g, 72%, *cis*/*trans* = 48:52. IR (KBr): 3479, 3002, 2933, 2836, 2253, 1651, 1614, 1514, 1455, 1258, 1187, 1028, 912, 739 cm^{-1}. ^{1}H NMR ($CDCl_3$, 400 MHz) δ 7.05–7.01 (m, 2H), 6.81–6.65 (m, 5H), 6.64 (dd, J = 7.3, 1.9 Hz, 1H), 6.56 (dd, J = 8.2, 2.1 Hz, 1H), 6.43 (d, J = 2.7 Hz, 1H), 6.31 (d, J = 2.7 Hz, 1H), 4.27–4.24 (m, 2H, *trans*-), 4.17–4.09 (m, 1H, *cis*-), 4.02 (dd, J = 12.2, 5.5 Hz, 1H, *cis*-), 3.86 (s, 3H), 3.83 (s, 3H), 3.80 (s, 3H), 3.79 (s, 3H), 3.64 (s, 3H), 3.60 (s, 3H), 3.17–3.08 (m, 2H), 2.83–2.73 (m, 2H), 2.37–2.31 (m, 1H), 2.20–2.14 (m, 1H), 2.06–2.00 (m, 1H), 1.86 (q, J = 11.9 Hz, 1H). ^{13}C NMR ($CDCl_3$, 101 MHz) δ 157.9, 149.0, 148.8, 147.6, 147.4, 140.3, 139.4, 139.0, 138.1, 130.3, 130.0, 127.0, 126.5, 120.9, 114.3, 114.1, 112.9, 112.4, 111.9, 111.6, 111.1, 111.0, 67.9, 64.7, 55.9, 55.20, 55.17, 55.15, 46.1, 43.0, 42.6, 40.2, 38.8, 37.5. HRMS (ESI) *m/z* $[M - H_2O + H]^+$ calcd for $C_{19}H_{21}O_3^+$ 297.1485, found 297.1483.

*4-(Furan-2-yl)-6-methoxy-1,2,3,4-tetrahydronaphthalen-2-ol (**14bb**).* Yellow oil. 0.14 g, 41%, *cis*/*trans* = 1:99. IR (KBr): 3445, 2931, 2360, 1779, 1615, 1506, 1464, 1267, 1041, 942, 767 cm^{-1}. ^{1}H NMR ($CDCl_3$, 400 MHz) δ 7.31 (dd, J = 1.8, 0.8 Hz, 1H), 7.03 (d, J = 8.4 Hz, 1H), 6.75 (dd, J = 8.4, 2.7 Hz, 1H), 6.62 (d, J = 2.7 Hz, 1H), 6.25 (dd, J = 3.2, 1.9 Hz, 1H), 5.88–5.82 (m, 1H), 4.36 (t, J = 5.9 Hz, 1H), 4.29–4.18 (m, 1H), 3.71 (s, 3H), 3.11 (dd, J = 16.1, 5.0 Hz, 1H), 2.71 (dd, J = 16.1, 7.5 Hz, 1H), 2.35–2.29 (m, 1H), 2.09–2.02 (m, 1H). ^{13}C NMR ($CDCl_3$, 101 MHz) δ 158.2, 157.8, 141.4, 136.4, 130.4, 126.4, 113.9, 113.3, 110.0, 106.6, 64.9, 55.2, 37.5, 37.3, 36.4. HRMS (ESI) *m/z* $[M - H_2O + H]^+$ calcd for $C_{15}H_{15}O_2^+$ 227.1067, found 227.1077.

*6-Chloro-4-(3,4-dimethoxyphenyl)-1,2,3,4-tetrahydronaphthalen-2-ol (**14ca**).* Yellow oil. 0.30 g, 67%, *cis*/*trans* = 44:56. IR (KBr): 3444, 2932, 2253, 1659, 1557, 1483, 1464, 1261, 1140, 1026, 912, 742 cm^{-1}. ^{1}H NMR ($CDCl_3$, 400 MHz) δ 7.12–7.03 (m, 4H), 6.87 (d, J = 2.1 Hz, 1H), 6.83–6.70 (m, 4H), 6.62–6.61 (m, 2H), 6.55 (dd, J = 8.2, 2.1 Hz, 1H), 4.31–4.23 (m, 2H, *trans*-), 4.18–4.10 (m, 1H, *cis*-), 4.00 (dd, J = 12.3, 5.6 Hz, 1H, *cis*-), 3.87 (s, 3H), 3.85 (s, 3H), 3.81 (s, 3H), 3.80 (s, 3H), 3.19–3.10 (m, 2H), 2.85–2.76 (m, 2H), 2.38–2.32 (m, 1H), 2.21–2.14 (m, 1H), 2.05–2.00 (m, 1H), 1.87 (q, J = 12.0 Hz, 1H). ^{13}C NMR ($CDCl_3$, 101 MHz) δ 149.1, 148.9, 147.8, 147.6, 141.0, 140.3, 138.2, 137.4, 133.3, 132.8, 131.8, 131.7, 130.7, 130.4, 129.4, 128.8, 126.6, 126.5, 120.84, 120.81, 111.8, 111.4, 111.3, 111.1, 67.5, 64.4, 55.9, 55.9, 45.7, 42.7, 42.0, 39.8, 38.9, 37.6. HRMS (ESI) *m/z* $[M - H_2O + H]^+$ calcd for $C_{18}H_{18}ClO_2^+$ 301.0990, found 301.0978.

*6-Chloro-4-(furan-2-yl)-1,2,3,4-tetrahydronaphthalen-2-ol (**14cb**).* Yellow oil. 0.13 g, 38%, *cis*/*trans* = 99:1. IR (KBr): 3418, 2930, 2250, 1779, 1715, 1596, 1486, 1053, 911, 735 cm^{-1}. ^{1}H NMR ($CDCl_3$, 400 MHz) δ 7.32 (dd, J = 1.7, 0.7 Hz, 1H), 7.14–7.12 (m, 1H), 7.06–7.04 (m, 2H), 6.27 (dd, J = 3.2, 2.0 Hz, 1H), 5.88 (d, J = 3.2 Hz, 1H), 4.36 (t, J = 6.1 Hz, 1H), 4.29–4.23 (m, 1H), 3.12 (dd, J = 16.7, 4.9 Hz, 1H), 2.74 (dd, J = 16.6, 7.2 Hz, 1H), 2.34–2.29 (m, 1H), 2.10–2.03 (m, 1H). ^{13}C NMR ($CDCl_3$, 101 MHz) δ 157.4, 141.7, 137.3, 132.7, 131.7, 130.8, 128.9, 127.0, 110.1, 106.87, 64.5, 37.6, 36.7, 36.1. HRMS (ESI) *m/z* [M − H_2O + H]$^+$ calcd for $C_{14}H_{12}ClO^+$ 231.0571, found 231.0542.

*4-(3,4-Dimethoxyphenyl)-6-nitro-1,2,3,4-tetrahydronaphthalen-2-ol (**14da**).* Yellow oil. 0.25 g, 55%, *cis*/*trans* = 56:44. IR (KBr): 3444, 2932, 2253, 1659, 1557, 1483, 1464, 1261, 1140, 1026, 912, 742 cm^{-1}. ^{1}H NMR ($CDCl_3$, 400 MHz) δ 8.00–7.95 (m, 2H), 7.87–7.81 (m, 2H), 7.04 (d, J = 8.6 Hz, 1H), 6.94 (d, J = 8.5 Hz, 1H), 6.83–6.77 (m, 2H), 6.70 (d, J = 8.0 Hz, 1H), 6.59–6.55 (m, 3H), 4.39–4.32 (m, 2H, *trans*-), 4.24–4.17 (m, 1H, *cis*-), 4.11–4.05 (m, 1H, *cis*-), 3.86 (s, 3H), 3.84 (s, 3H), 3.79 (s, 6H), 3.30–3.25 (m, 2H), 2.99–2.92 (m, 2H), 2.46–2.40 (m, 1H), 2.30–2.22 (m, 1H), 2.11–2.03 (m, 1H), 1.93 (q, J = 11.9 Hz, 1H). ^{13}C NMR ($CDCl_3$, 101 MHz) δ 149.3, 149.2, 148.1, 147.9, 146.8, 146.6, 146.4, 137.5, 136.8, 136.7, 136.0, 130.6, 130.1, 124.39, 124.36, 124.14, 124.12, 121.03, 121.01, 120.93, 120.90, 120.9, 120.8, 111.82, 111.76, 111.5, 111.4, 111.34, 111.28, 66.9, 64.1, 55.9, 55.9, 45.9, 42.3, 41.9, 39.4, 39.2, 38.0. HRMS (ESI) *m/z* [M + Na]$^+$ calcd for $C_{18}H_{19}NNaO_5^+$ 352.1155, found 352.1158.

*4-(3,4-Dimethoxyphenyl)-1-methyl-1,2,3,4-tetrahydronaphthalen-2-ol (**14ea**).* Yellow oil. 0.27 g, 65%. IR (KBr): 3420, 2934, 1591, 1487, 1463, 1262, 1155, 1028, 738 cm^{-1}. ^{1}H NMR ($CDCl_3$, 400 MHz) δ 7.33–7.16 (m, 3H), 7.05–7.01 (m, 2H), 6.89–6.71 (m, 5H), 6.65–6.53 (m, 4H), 4.31 (t, J = 6.9 Hz, 1H), 4.25–4.17 (m, 1H), 4.09–4.04 (m, 1H), 3.92–3.89 (m, 1H), 3.85 (s, 6H), 3.78 (s, 6H), 3.17–3.13 (m, 1H), 2.91–2.85 (m, 1H), 2.39–2.29 (m, 1H), 2.17–2.09 (m, 2H), 2.02–1.94 (m, 1H), 1.47 (d, J = 6.8 Hz, 3H), 1.37 (d, J = 6.8 Hz, 3H). ^{13}C NMR ($CDCl_3$, 101 MHz) δ 149.0, 148.9, 148.8, 147.6, 147.4, 147.3, 140.1, 140.01, 139.97, 139.8, 139.2, 139.0, 138.64, 137.95, 137.8, 129.9, 129.6, 129.0, 128.9, 128.2, 127.3, 126.5, 126.4, 126.1, 126.0, 125.8, 120.8, 112.07, 112.02, 111.7, 111.2, 111.0, 73.6, 70.6, 69.5, 68.2, 55.9, 45.5, 42.6, 42.1, 41.9, 41.7, 41.4, 38.3, 37.7, 36.9, 21.7, 18.1, 16.8. HRMS (ESI) *m/z* [M − H_2O + H]$^+$ calcd for $C_{19}H_{21}O_2^+$ 281.1536, found 281.1518.

*4-(Furan-2-yl)-1-methyl-1,2,3,4-tetrahydronaphthalen-2-ol (**14eb**).* Yellow oil. 0.12 g, 36%. IR (KBr): 3421, 2928, 1717, 1617, 1504, 1457, 1009, 745 cm^{-1}. ^{1}H NMR ($CDCl_3$, 400 MHz) δ 7.35–7.28 (m, 2H), 7.25–7.16 (m, 3H), 7.13– 7.02 (m, 4H), 6.28–6.26 (m, 2H), 6.25–6.23 (m, 1H), 5.90 (d, J = 3.2 Hz, 1H), 5.81 (d, J = 2.7 Hz, 1H), 4.41–4.34 (m, 2H), 4.24–4.21 (m, 1H), 3.91–3.87 (m, 1H), 3.14–3.12 (m, 1H), 2.88–2.81 (m, 1H), 2.39–2.33 (m, 1H), 2.27–2.16 (m, 2H), 2.11–2.05 (m, 1H), 1.38 (d, J = 7.0 Hz, 3H), 1.31 (d, J = 7.1 Hz, 3H). ^{13}C NMR ($CDCl_3$, 101 MHz) δ 158.8, 158.2, 141.4, 141.3, 140.3, 139.5, 135.1, 134.7, 129.4, 128.9, 128.8, 126.89, 126.87, 126.1, 109.0, 110.0, 106.7, 106.5, 70.7, 67.9, 41.3, 38.2, 37.2, 36.28, 33.6, 32.8, 20.6, 16.6. HRMS (ESI) *m/z* [M − H_2O + H]$^+$ calcd for $C_{15}H_{15}O^+$ 211.1117, found 211.1110.

*4-(3,4-Dimethoxyphenyl)-4-methyl-1,2,3,4-tetrahydronaphthalen-2-ol (**14fa**).* Yellow oil. 0.21 g, 50%, *cis*/*trans* = 37:63. IR (KBr): 3440, 2933, 1590, 1490, 1238, 1047, 727 cm^{-1}. ^1H NMR ($CDCl_3$, 400 MHz) *δ* 7.24–7.20 (m, 1H), 7.14–7.08 (m, 4H), 6.82–6.78 (m, 3H), 6.71–6.65 (m, 4H), 6.59–6.56 (m, 1H), 6.37 (dd, *J* = 8.5, 2.3 Hz, 1H), 4.31–4.23 (m, 1H, *trans*-), 4.19–4.15 (m, 1H, *cis*-), 3.87 (s, 6H), 3.79 (s, 6H), 2.99–2.92 (m, 2H), 2.88–2.73 (m, 2H), 2.31–2.27 (m, 1H), 2.11–2.07 (m, 1H), 1.96–1.87 (m, 2H), 1.75 (s, 3H), 1.67 (s, 3H). ^{13}C NMR ($CDCl_3$, 101 MHz) *δ* 149.0, 147.6, 139.5, 137.5, 134.6, 129.5, 129.3, 129.20, 129.15, 128.7, 128.2, 126.5, 126.3, 126.1, 126.04, 126.01, 121.9, 119.6, 119.0, 112.0, 111.2, 110.9, 110.6, 110.5, 110.4, 72.7, 65.6, 64.5, 55.9, 55.8, 55.8, 53.6, 50.6, 49.6, 44.7, 44.1, 40.0, 39.7, 39.1, 31.6, 31.1, 30.5, 22.6, 15.8, 14.1. HRMS (ESI) *m/z* $[M + Na]^+$ calcd for $C_{19}H_{22}NaO_3^+$ 321.1461, found 321.1474.

*4-(Furan-2-yl)-4-methyl-1,2,3,4-tetrahydronaphthalen-2-ol (**14fb**).* Red oil. 0.08 g, 25%, *cis*/*trans* = 1:99. IR (KBr): 3431, 2957, 2853, 1750, 1654, 1465, 1379, 1238, 1047, 750 cm^{-1}. ^1H NMR ($CDCl_3$, 400 MHz) *δ* 7.31–7.27 (m, 2H), 7.19–7.07 (m, 3H), 6.16 (dd, *J* = 3.1, 2.0 Hz, 1H), 5.55 (d, *J* = 3.1 Hz, 1H), 4.08–4.00 (m, 1H), 3.16–3.11 (m, 1H), 2.77 (dd, *J* = 15.7, 10.1 Hz, 1H), 2.74 (dt, *J* = 12.5, 2.9 Hz, 1H), 1.82–1.72 (m, 1H), 1.71 (s, 3H). ^{13}C NMR ($CDCl_3$, 101 MHz) *δ* 162.0, 141.2, 139.8, 134.7, 129.4, 127.8, 126.8, 126.3, 109.7, 105.8, 65.0, 46.0, 41.4, 39.6, 28.9. HRMS (ESI) *m/z* $[M - H_2O + H]^+$ calcd for calcd for $C_{15}H_{15}O^+$ 211.1117, found 211.1115.

*4-(furan-2-yl)-4-phenyl-1,2,3,4-tetrahydronaphthalen-2-ol (**14ga**).* Yellow oil. 0.08 g, 20%, *cis*/*trans* = 13:87. IR (KBr): 3420, 3050, 1922, 1716, 1600, 1504, 1050 cm^{-1}. ^1H NMR ($CDCl_3$, 400 MHz) *δ* 7.45 (dd, *J* = 1.8, 0.9 Hz, 1H), 7.27–7.17 (m, 6H), 7.09–7.05 (m, 1H), 6.98–6.93 (m, 2H), 6.86 (d, *J* = 7.8 Hz, 1H), 6.29 (dd, *J* = 3.2, 1.9 Hz, 1H), 5.70 (dd, *J* = 3.2, 0.8 Hz, 1H), 4.03–3.98 (m, 1H), 3.21–3.19 (m, 1H), 3.00–2.90 (m, 2H), 2.34 (dd, *J* = 12.6, 11.4 Hz, 1H), 2.61 (dd, *J* = 12.1, 2.7 Hz, 1H), 2.08–2.01 (m, 1H). ^{13}C NMR ($CDCl_3$, 101 MHz) *δ* 148.2, 141.8, 139.8, 135.2, 130.9, 129.3, 128.4, 128.1, 127.9, 126.9, 126.4, 125.9, 110.09, 110.06, 65.5, 51.9, 46.0, 39.4. HRMS (ESI) *m/z* $[M - H_2O + H]^+$ calcd for $C_{20}H_{17}O^+$ 273.1274, found 273.1254.

*4-(3,4-Dimethoxyphenyl)-1,2,3,4-tetrahydrophenanthren-2-ol (**14ha**).* Yellow oil. 0.34 g, 73%, *cis*/*trans* = 52:48. IR (KBr): 3417, 3051, 2933, 2836, 2252, 1732, 1603, 1505, 1417, 1259, 1139, 912, 743 cm^{-1}. ^1H NMR ($CDCl_3$, 400 MHz) *δ* 7.77–7.70 (m, 4H), 7.62–7.57 (m, 2H), 7.35–7.20 (m, 6H), 6.68–6.58 (m, 4H), 6.44 (dd, *J* = 8.2, 2.1 Hz, 1H), 6.32 (dd, *J* = 8.3, 2.1 Hz, 1H), 4.89–4.87 (m, 1H, *trans*-), 4.77 (t, *J* = 7.1 Hz, 1H, *cis*-), 4.22–4.14 (m, 2H), 3.76 (s, 3H), 3.75 (s, 3H), 3.74 (s, 3H), 3.71 (s, 3H), 3.38–3.31 (m, 1H), 3.21–3.05 (m, 2H), 2.92 (dd, *J* = 16.5, 10.0 Hz, 1H), 2.59–2.54 (m, 1H), 2.35–2.31 (m, 1H), 2.23–2.07 (m, 2H). ^{13}C NMR ($CDCl_3$, 101 MHz) *δ* 149.1, 147.1, 140.1, 138.8, 133.4, 132.9, 132.7, 131.9, 128.4, 128.1, 127.8, 127.5, 126.1, 125.7, 125.1, 124.9, 124.7, 124.5, 120.4, 119.3, 111.5, 111.4, 110.8, 66.9, 63.5, 55.8, 55.8, 55.7, 42.4, 41.9, 41.3, 41.2, 40.0, 39.7. HRMS (ESI) *m/z* $[M - H_2O + H]^+$ calcd for $C_{22}H_{21}O_2^+$ 317.1536, found 317.1534.

*4-(Furan-2-yl)-1,2,3,4-tetrahydrophenanthren-2-ol (**14hb**).* Yellow oil. 0.16 g, 43%, *cis*/*trans* = 1:99. IR (KBr): 3420, 3051, 2926, 2247, 1922, 1716, 1602, 1504, 1050, 923, 744

cm^{-1}. ^{1}H NMR ($CDCl_3$, 400 MHz) δ 7.79–7.76 (m, 1H), 7.72–7.70 (m, 2H), 7.39–7.32 (m, 3H), 7.25–7.23 (m, 1H), 6.13–6.12 (m, 1H), 5.42–5.41 (m, 1H), 4.97 (d, J = 5.2 Hz, 1H), 4.21–4.13 (m, 1H), 3.33 (dd, J = 16.3, 5.9 Hz, 1H), 2.91 (dd, J = 16.5, 10.1 Hz, 1H), 2.61 (dd, J = 12.1, 2.7 Hz, 1H), 2.08–2.01 (m, 1H). ^{13}C NMR ($CDCl_3$, 101 MHz) δ 157.8, 141.0, 133.3, 132.5, 131.9, 129.6, 128.4, 127.9, 127.8, 126.2, 125.0, 123.8, 110.2, 107.4, 64.4, 39.5, 37.5, 36.2. HRMS (ESI) *m/z* [M – H_2O + H]$^+$ calcd for $C_{18}H_{15}O^+$ 247.1117, found 247.1114.

*2,2'-(2-(4-Nitro-2-vinylphenyl)ethane-1,1-diyl)difuran (**15**).* Red oil. 0.13 g, 30%. IR (KBr): 3462, 2923, 2361, 1583, 1518, 1353, 1147, 1012, 915, 734 cm^{-1}. ^{1}H NMR ($CDCl_3$, 400 MHz) δ 7.99 (dd, J = 8.6, 2.4 Hz, 1H), 7.75 (d, J = 2.4 Hz, 1H), 7.56 (d, J = 8.6 Hz, 1H), 7.34 (d, J = 1.9 Hz, 2H), 6.93 (dd, J = 17.3, 11.0 Hz, 1H), 6.25 (dd, J = 3.3, 1.9 Hz, 2H), 6.00 (d, J = 3.2 Hz, 2H), 5.76 (dd, J = 17.3, 0.9 Hz, 1H), 5.49 (dd, J = 11.0, 0.9 Hz, 1H), 4.30 (t, J = 7.7 Hz, 1H), 3.44 (d, J = 7.8 Hz, 2H). ^{13}C NMR ($CDCl_3$, 101 MHz) δ 153.2, 146.8, 143.3, 141.8, 137.8, 132.5, 126.6, 125.23, 121.8, 119.9, 110.3, 106.8, 39.9, 36.5. HRMS (ESI) *m/z* [M + H]$^+$ calcd for $C_{18}H_{16}NO_4^+$ 310.1074, found 310.1053.

2.5 General procedure for the synthesis of 3-(2-bromophenyl)propan-1-ols 17.

*Synthesis of 3-(2-bromophenyl)propan-1-ol (**17**)* [7]. $LiAlH_4$ (12.34 mmol, 1.00 equiv, 2.5 M in THF) was added dropwise into a solution of methyl 3-(2-bromophenyl)propanoate (**16**, 12.34 mmol, 1.00 equiv) in anhydr. THF (30 mL) at 0 °C under an inert atmosphere of N_2. After the addition was complete, the reaction mixture was stirred for further 2 h at 0 °C. Then, the reaction was quenched by sequential addition of water (0.5 g), 15% aqueous NaOH (0.5 g) and water (1.5 g), and stirred for further 10 min, then filtered. The cake was washed with THF (10 mL). The combined filtrates were dried over Na_2SO_4 and filtered, and the filtrate was concentrated under reduced pressure. The residue was purified by column chromatography on silica gel eluting with EA/PE (boiling point range 60–90 °C) (*v*/*v* = 1:9) to give 3-(2-bromophenyl)propan-1-ol (**17**). Colorless oil. 1.86 g, 70%. IR (KBr): 3383, 2937, 2867, 1567, 1471, 1438, 1160, 1057, 1019, 748 cm^{-1}. ^{1}H NMR ($CDCl_3$, 400 MHz) δ 7.54–7.47 (m, 1H), 7.24–7.19 (m, 2H), 7.09–6.99 (m, 1H), 3.68 (t, J = 6.4 Hz, 2H), 2.90–2.76 (m, 2H), 1.96–1.83 (m, 2H). ^{13}C NMR ($CDCl_3$, 101 MHz) δ 141.1, 132.8, 130.4, 127.6, 127.5, 124.4, 62.1, 32.7, 32.4. HRMS (ESI) *m/z* [M – H_2O + H]$^+$ calcd for $C_9H_{10}Br^+$ 196.9960, found 196.9933.

2.6 General procedure for the synthesis of 3-(2-vinylphenyl)propan-1-ols 18.

*Synthesis of 3-(2-vinylphenyl)propan-1-ol (**10**)* [8]. Alcohol **17** (8.00 mmol, 1.00 equiv), pinacol vinylboronate (9.60 mmol, 1.20 equiv) and Pd(dppf)Cl_2 (0.80 mmol, 0.10 equiv) were added to dioxane (16 mL) under an inert atmosphere of N_2 at 20 °C. Then, K_2CO_3 (10.40 mmol, 1.30 equiv) in H_2O (4 mL) was added to the above mixture and the reaction mixture was heated at reflux for 2 h. Then, saturated aqueous NaCl (30 mL) was added, and the mixture was extracted with ethyl acetate (3 × 30 mL). The combined organic layers were dried over Na_2SO_4 and filtered, and the filtrate was concentrated under reduced pressure. The residue was purified by column chromatography on silica gel eluting with EA/PE (boiling point

range 60–90 °C) (*v*/*v* = 1:9) to give product **18**. Light yellow oil. 0.97 g, 75%. IR (KBr): 3374, 2940, 2872, 1625, 1484, 1449, 1382, 1059, 913, 773 cm^{-1}. ^{1}H NMR ($CDCl_3$, 400 MHz) δ 7.51–7.47 (m, 1H), 7.22–7.13 (m, 3H), 7.01 (dd, *J* = 17.4, 10.9 Hz, 1H), 5.65 (dd, *J* = 17.4, 1.4 Hz, 1H), 5.29 (dd, *J* = 11.0, 1.4 Hz, 1H), 3.66 (t, *J* = 6.3 Hz, 2H), 2.80–2.74 (m, 2H), 1.88–1.79 (m, 2H). ^{13}C NMR ($CDCl_3$, 101 MHz) δ 139.2, 136.5, 134.5, 129.5, 127.8, 126.3, 125.8, 115.6, 62.2, 33.8, 29.4. HRMS (ESI) *m/z* [M – H_2O + H]$^+$ calcd for $C_{11}H_{13}^+$ 145.1012, found 145.1005.

2.7 General procedure for the synthesis of 3-(2-vinylphenyl)propanals 19.

*Synthesis of 3-(2-vinylphenyl)propanal (**19**).* Dess–Martin periodinane (6.00 mmol, 1.20 equiv) was added in portions to a solution of compound **18** (5.00 mmol, 1.00 equiv) in CH_2Cl_2 (8 mL) at 0 °C under an inert atmosphere of N_2. The resultant mixture was stirred for further 2 h at 20 °C, then quenched with 8% aqueous $NaHCO_3$ (20 mL). The mixture was filtered and the cake was washed with CH_2Cl_2 (10 mL). The filtrate was separated and the aqueous layer was extracted with CH_2Cl_2 (2 × 10 mL). The combined organic layers were dried over Na_2SO_4 and filtered, and the filtrate was concentrated under reduced pressure. The residue was purified by column chromatography on silica gel eluting with EA/PE (boiling point range 60–90 °C) (*v*/*v* = 1:19) to give product **19**. Yellow oil. 0.65 g, 81%. IR (KBr): 3431, 2941, 2824, 2724, 1728, 1625, 1484, 1451, 1411, 1387, 1055, 913, 774 cm^{-1}. ^{1}H NMR ($CDCl_3$, 400 MHz) δ 9.80 (t, *J* = 1.3 Hz, 1H), 7.51–7.46 (m, 1H), 7.24–7.14 (m, 3H), 6.94 (dd, *J* = 17.3, 11.0 Hz, 1H), 5.66 (dd, *J* = 17.5, 1.4 Hz, 1H), 5.33 (dd, *J* = 10.9, 1.4 Hz, 1H), 3.05–2.98 (m, 2H), 2.75–2.68 (m, 2H). ^{13}C NMR ($CDCl_3$, 101 MHz) δ 201.5, 137.6, 136.5, 134.1, 129.3, 128.0, 126.8, 126.1, 116.3, 44.7, 25.5. HRMS (ESI) *m/z* [M – H_2O + H]$^+$ calcd for $C_{11}H_{11}^+$ 143.0855, found 143.0841.

5-Aryl-7-hydroxy-benzo[7]annulens **20a** and **20b** were synthesized analogously as described for **6a**.

*5-(3,4-Dimethoxyphenyl)-6,7,8,9-tetrahydro-5H-benzo[7]annulen-7-ol (**20a**).* Yellow oil. 0.25 g, 60%, *cis*/*trans* = 54:46. IR (KBr): 3445, 3063, 2933, 2253, 1738, 1634, 1487, 1262, 1143, 1029, 913, 742 cm^{-1}. ^{1}H NMR ($CDCl_3$, 400 MHz) δ 7.17–6.98 (m, 7H), 6.89 (d, *J* = 8.1 Hz, 1H), 6.91–6.68 (m, 5H), 6.49 (d, *J* = 7.6 Hz, 1H), 4.63–4.58 (m, 1H, *trans*-), 4.20–4.14 (m, 1H, *cis*-), 4.07–3.98 (m, 2H), 3.90 (s, 3H), 3.86 (s, 3H), 3.84 (s, 3H), 3.79 (s, 3H), 2.97–2.76 (m, 2H), 2.59–2.49 (m, 2H), 2.47–2.37 (m, 1H), 2.35–2.24 (m, 3H), 1.93–1.81 (m, 3H), 1.40 (q, *J* = 11.9 Hz, 1H). ^{13}C NMR ($CDCl_3$, 101 MHz) δ 148.9, 147.5, 145.3, 142.2, 141.0, 137.2, 129.5, 129.2, 127.1, 126.4, 126.3, 126.24, 126.21, 120.5, 120.2, 112.1, 111.8, 111.2, 111.1, 74.3, 68.9, 55.9, 55.9, 55.8, 43.6, 41.3, 36.4, 35.4, 31.0, 29.6. HRMS (ESI) *m/z* [M – H_2O + H]$^+$ calcd for $C_{19}H_{21}O_2^+$ 281.1536, found 281.1506.

*5-(Furan-2-yl)-6,7,8,9-tetrahydro-5H-benzo[7]annulen-7-ol (**20b**).* Yellow oil. 0.10 g, 31%, *cis*/*trans* = 26:74. IR (KBr): 3447, 2927, 1718, 1636, 1489, 1452, 1383, 1034, 911, 732 cm^{-1}. ^{1}H NMR ($CDCl_3$, 400 MHz) δ 7.39–7.36 (m, 1H), 7.17–7.09 (m, 4H), 6.33–6.26 (m, 1H), 5.85 (s, 1H), 4.55 (d, *J* = 8.3 Hz, 1H), 4.10–3.99 (m, 2H), 2.85–2.46 (m, 2H), 2.45–2.26 (m, 2H), 2.05–1.80 (m, 2H). ^{13}C NMR ($CDCl_3$, 101 MHz) δ 157.3, 141.9, 141.3, 129.8, 129.2, 126.9,

126.7, 126.6, 126.4, 110.1, 106.4, 69.6, 39.5, 35.7, 29.8. HRMS (ESI) *m/z* [M − H_2O + H]$^+$ calcd for $C_{15}H_{15}O^+$ 211.1117, found 211.1078.

2.8 Synthesis of 4-(furan-2-yl)-1,2,3,4-tetrahydronaphthalen-2-yl 4-methylbenzene-sulfonate 21.

p-Toluenesulfonyl chloride (2.40 mmol, 1.20 equiv) was added dropwise into a solution of 4-(furan-2-yl)-1,2,3,4-tetrahydronaphthalen-2-ol (**14af**, 2.00 mmol, 1.00 equiv) in pyridine (4 mL) at 0 °C under an inert atmosphere of N_2. The resultant mixture was stirred for 20 h at 20 °C. Then, saturated aqueous NaCl solution (10 mL) and CH_2Cl_2 (20 mL) were added into the reaction mixture, and stirred for another 10 min. The aqueous layer was extracted with CH_2Cl_2 (2 × 10 mL). The combined organic layers were dried over with Na_2SO_4 and filtered, and the filtrate was concentrated under reduced pressure. The residue was purified by column chromatography on silica gel eluting with EA/PE (boiling point range 60–90 °C) (*v/v* = 1:10) to give the tosylate **21**. Red oil. 0.66 g, 90%, *cis*/*trans* = 21:79. IR (KBr): 3420, 2954, 1598, 1494, 1452, 1361, 1177, 1097, 1041, 911, 736 cm^{-1}. ^{1}H NMR ($CDCl_3$, 400 MHz) δ 7.79–7.68 (m, 2H), 7.34–7.23 (m, 3H), 7.18–7.06 (m, 2H), 7.06–7.01 (m, 2H), 6.24 (dd, *J* = 3.1, 1.9 Hz, 1H), 5.82 (d, *J* = 3.2 Hz, 1H), 5.02–4.91 (m, 1H), 4.32 (t, *J* = 6.0 Hz, 1H), 3.21–3.07 (m, 1H), 3.03–2.95 (m, 1H), 2.44 (s, 3H), 2.29–2.14 (m, 2H). ^{13}C NMR ($CDCl_3$, 101 MHz) δ 156.9, 144.6, 141.7, 141.6, 134.7, 134.0, 132.6, 129.9, 129.8, 129.3, 129.1, 127.7, 127.0, 126.5, 110.0, 106.9, 76.4, 36.7, 35.3, 33.6, 21.7. HRMS (ESI) *m/z* [M − *p*TSA + H]$^+$ calcd for $C_{14}H_{13}O^+$ 197.0961, found 197.0938.

2.9 Synthesis of 4-(furan-2-yl)-*N*,*N*-dimethyl-1,2,3,4-tetrahydronaphthalen-2-amine hydrochloride (22)

Compound **21** (1.00 mmol, 1.00 equiv) was added to 40% dimethylamine in water (20.00 mmol, 20.00 equiv) at 20 °C under an inert atmosphere of N_2, then heated in a sealed tube at 90 °C for 20 h. Afterwards, saturated aqueous NaCl (10 mL) and CH_2Cl_2 (20 mL) were added into the reaction mixture, and stirred for another 10 min. The aqueous layer was extracted with CH_2Cl_2 (2 × 10 mL). The combined organic layers were dried over with Na_2SO_4 and filtered, and the filtrate was concentrated under reduced pressure. The residue was purified by column chromatography on silica gel eluting with EA/PE (boiling point range 60–90 °C) (*v/v* = 1:10) and acidified with 1 M HCl in methanol (1.00 mmol, 1.00 equiv) to give the hydrochloride salt **22**. Yellow oil. 0.19 g, 70%, *cis*/*trans* = 79:21. IR (KBr): 3424, 2957, 2920, 2850, 2668, 1653, 1467, 1234, 1081, 741 cm^{-1}. ^{1}H NMR ($CDCl_3$, 400 MHz) δ 12.71 (s, 1H), 7.33–7.29 (m, 1H), 7.20–7.09 (m, 3H), 6.94–6.88 (m, 1H), 6.33–6.30 (m, 1H), 6.22–6.18 (m, 1H), 4.38–4.27 (m, 1H), 3.67–3.60 (m, 1H), 3.37–3.33 (m, 1H), 3.24–3.13 (m, 1H), 2.82 (s, 6H), 2.67–2.65 (m, 1H), 2.27–2.16 (m, 1H). ^{13}C NMR ($CDCl_3$, 101 MHz) δ 155.0, 142.0, 135.0, 131.4, 129.5, 128.1, 127.33, 127.28, 110.2, 107.5, 61.4, 39.5, 39.3, 38.9, 30.1, 29.6. HRMS (ESI) *m/z* [M + H]$^+$ calcd for $C_{16}H_{20}NO^+$ 242.1539, found 242.1510.

3. *cis*- and *trans*-Configuration analysis method for 4-aryltetralin-2-ols

The relative *cis*- and *trans*-configuration of the C-2 hydroxy group and the C-4 aryl substituent of the 4-aryltetralin-2-ols were determined by 2D NMR analysis. As exemplified by 4-(3,4-dimethoxyphenyl)-1,2,3,4-tetrahydronaphthalen-2-ol (**14aa**), first HSQC analysis was used to determine H_3. The C_2 chemical shift in the ^{13}C NMR spectrum is expected to be in the range of 60 to 70 ppm. The assignment of H_3 by HSQC results through the correlation signal between H_3 and C_2. Then, H_1 and H_2 could be assigned by COSY and HSQC. After that, NOE spectroscopy was applied to analyze the relative *cis*- and *trans*-configuration. If there is a NOE correlation between H_1 and H_3, and meanwhile H_1 and H_3 also show a strong NOE correlation with H_{2a}, the compound is assigned to be *cis*-configured. Otherwise, it is the *trans*-isomer.

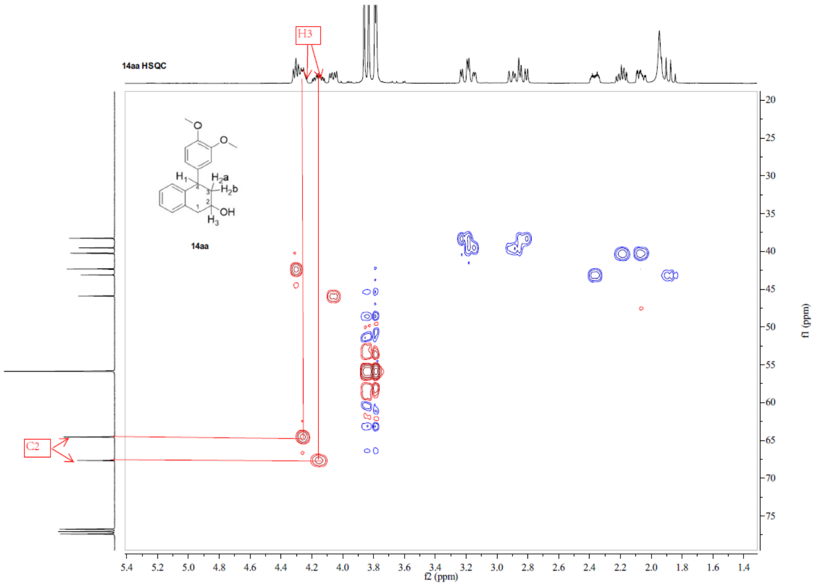

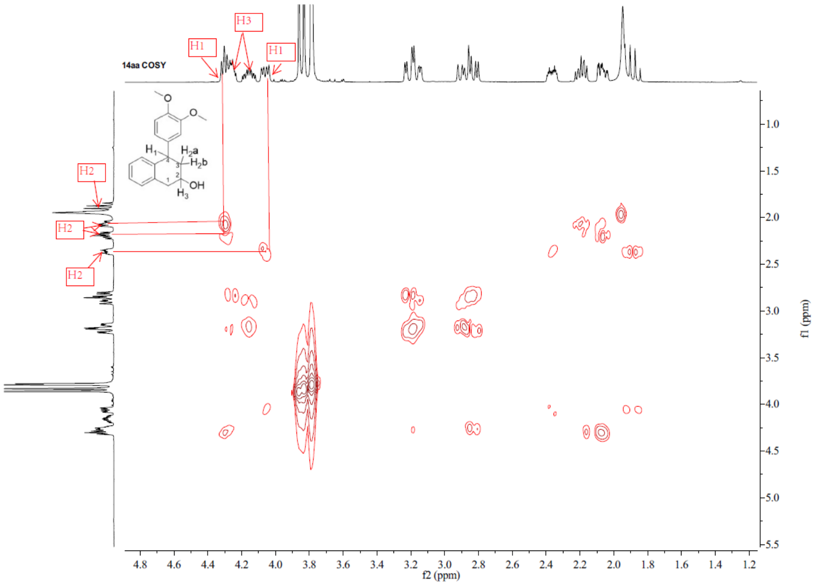

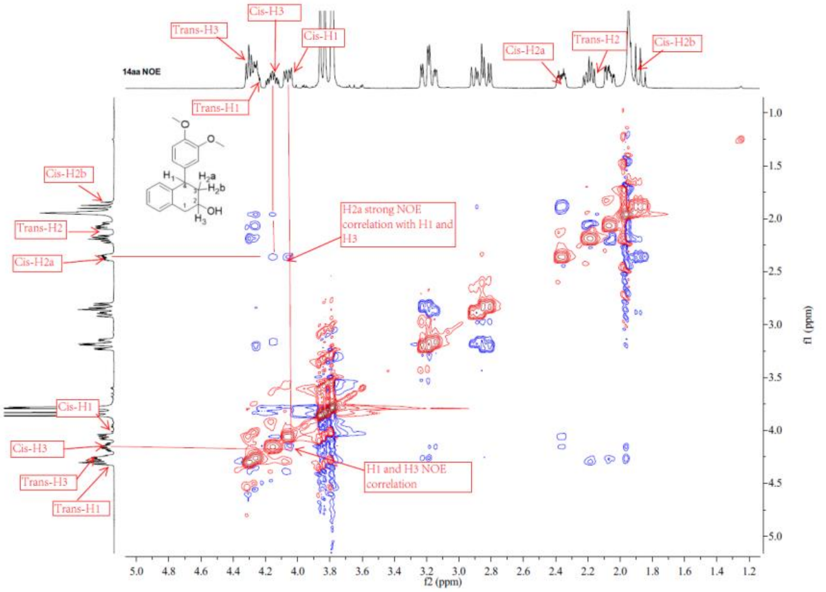
14aa NOE
Trans-H3
Cis-H3
Cis-H1
Trans-H1
Cis-H2a
Trans-H2
Cis-H2b
H2a strong NOE correlation with H1 and H3
H1 and H3 NOE correlation
f2 (ppm)
f1 (ppm)

4. *trans*-Stereoselectivity study of 14af and related spectra

Procedure for the preparation of the *cis*- and *trans*-mixture of **14af**: Aluminum chloride (1.54 mmol, 1.10 equiv) was added portionwise into the mixture of aldehyde **13a** (1.40 mmol, 1.00 equiv) and furan (1.47 mmol, 1.05 equiv) dissolved in anhydr CH_2Cl_2 (6 mL) at 0 °C under an inert atmosphere of N_2. The resultant solution was stirred for further 2 h at 0 °C, then the reaction was quenched with saturated aqueous NaCl (10 mL), and extracted with CH_2Cl_2 (2 × 10 mL). The combined organic layers were dried over Na_2SO_4 and filtered, and the filtrate was concentrated under reduced pressure. The residue was purified by column chromatography on silica gel eluting with EA/PE (boiling point range 60–90 °C) (*v*/*v* = 1:4) to give **14af** as a *cis*- and *trans*-mixture . Yellow oil. 0.06 g, 20%, *cis*/*trans* = 21:79.

An HPLC method has been established (Table S2). With the analysis method at hand, the predominating formation of the *trans*-stereoisomer of **14af** was studied. Several samples were taken to monitor the reaction catalyzed by $BF_3{\cdot}Et_2O$. It was found that the *cis*-isomer gradually transforms into *trans*-**14af**. The ratio of *cis*/*trans* that was finally reached was 1:99 after 2 hours. This may be an evidence of the reversible addition of furan leading to equilibration to the more stable *trans*-product. For furan and its derivatives as nucleophiles, this equilibration led to the formation of the more stable *trans*-products under the action of $BF_3{\cdot}Et_2O$ over time.

Table S2: HPLC method for the *trans*-stereoselectivity study of **14af**

instrument	Agilent 1260		
column	DIKMA Diamonsil, C18, 200 × 4.6mm, 5 μm		
oven	30 °C		
mobile phase gradient program:	time (min)	A%: 0.1%TFA aq.	B%: MeCN
	0.0	52	48
	15.0	52	48
	18.0	5	95
	20.0	5	95
	21.0	52	48
	26.0	52	48
stop time	26 min		
post time	OFF		
flow rate	1.0 mL/min		
UV detector	210 nm		
injection volume	5 μL		

Mixture of 14af (*cis/trans* = 21:79) ^{1}H *NMR (CDCl$_3$, 400 MHz)*

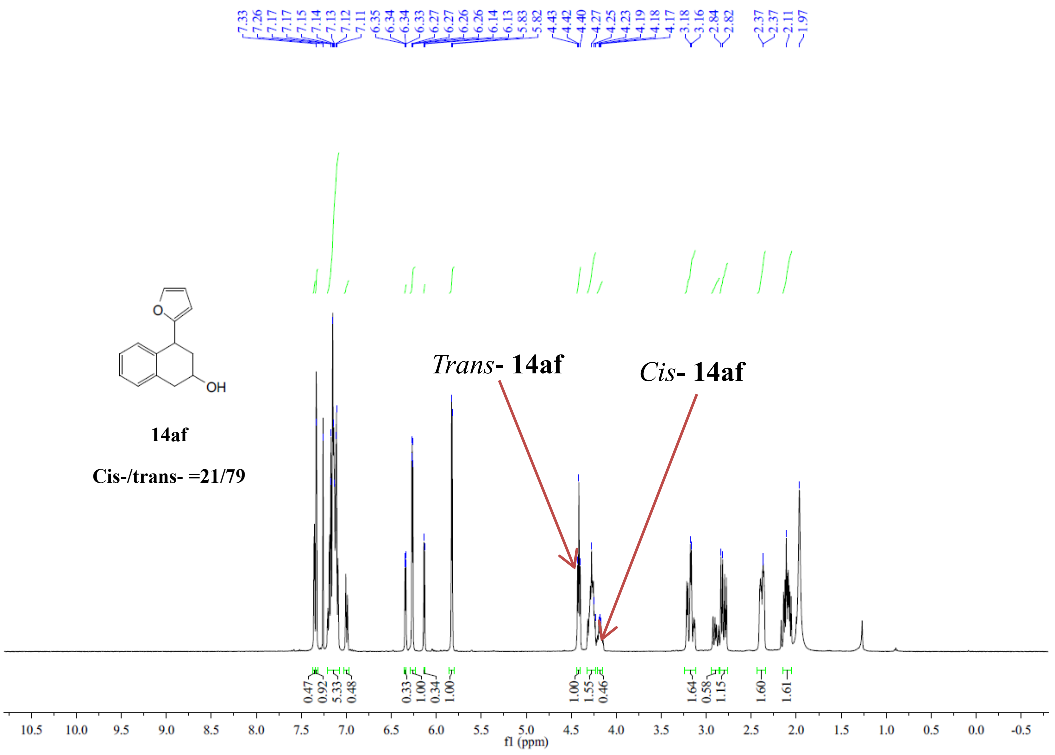

Mixture of 14af (*cis/trans* = 21:79) ^{13}C *NMR (CDCl$_3$, 101 MHz)*

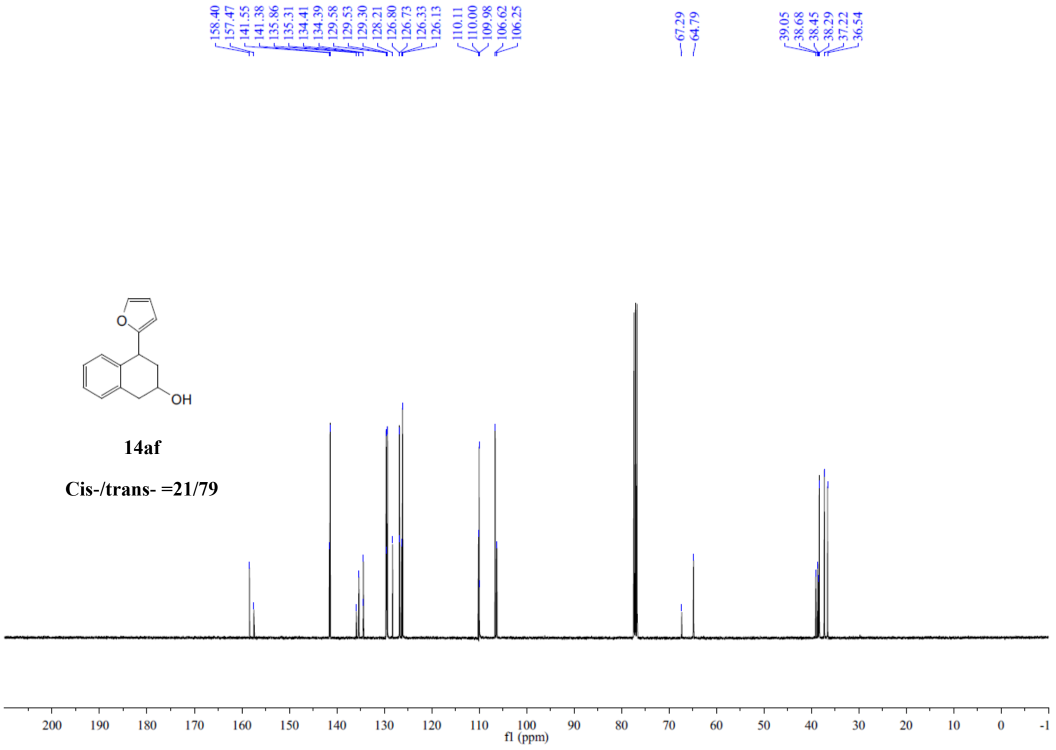

Mixture of 14af (*cis*/*trans* = 21:79) HPLC spectrum

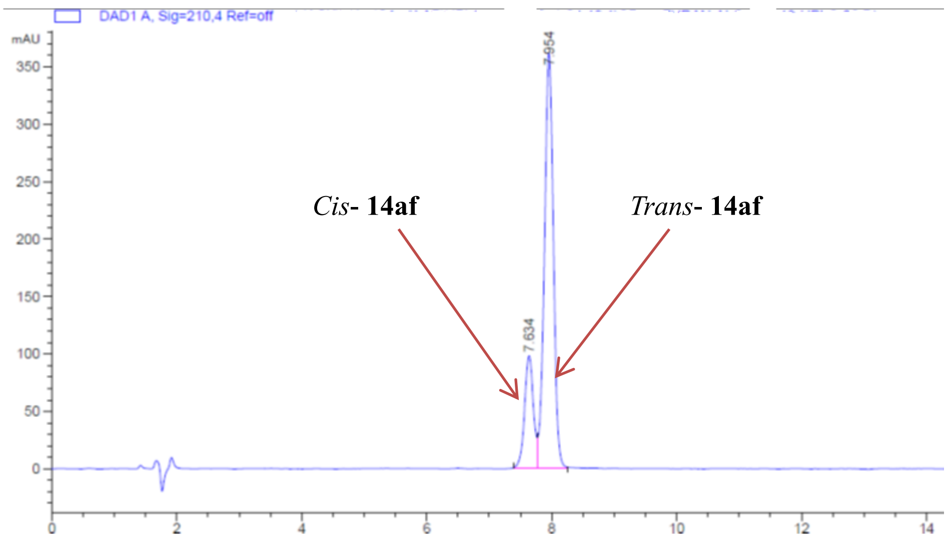

#	[min]		[min]	[mAU*s]	[mAU]	%
1	7.634	MF	0.1631	955.51837	97.66332	20.9588
2	7.954	FM	0.1661	3603.52393	361.48886	79.0412

HPLC 3D overlap spectrum of 14af in process analysis

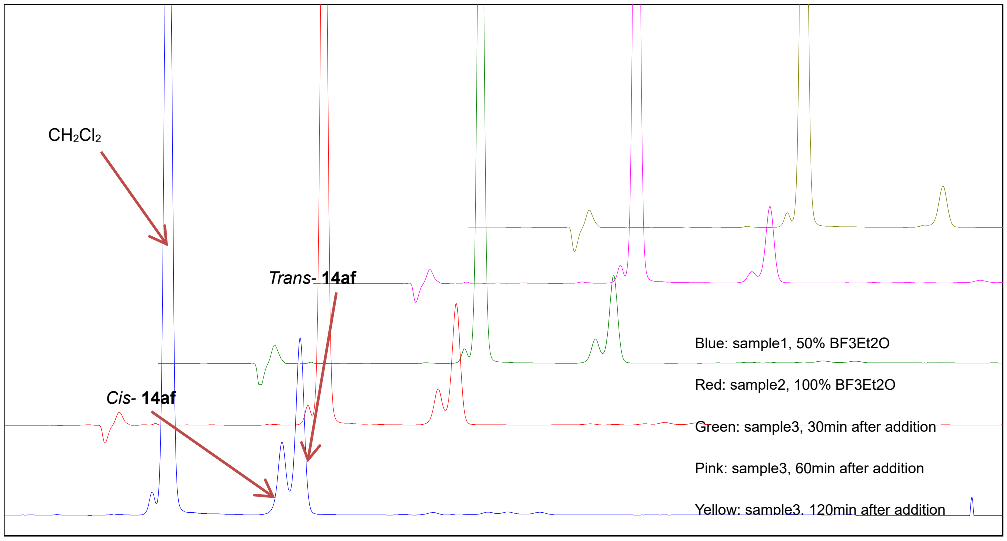

5. Stereochemical purity erosion study of 21 and related spectra

The mixture of **14af** (*cis*/*trans* = 21:79) was used to prepare the mixture of **21** (*cis*/*trans* = 26:74). Then, the established HPLC method was used to determine the alternation (Table S3).

Table S3: HPLC method for the study of the stereochemical purity erosion of **21**

instrument	Agilent 1260		
column	DIKMA Diamonsil, C18, 200 × 4.6 mm, 5 μm		
oven	30 °C		
mobile phase gradient program:	Time (min)	A%: 0.1%H_3PO_4 *aq.*	B%: MeCN
	0.0	52	48
	10.0	52	48
	20.0	5	95
	25.0	5	95
stop time	25 min		
post time	4 min		
flow rate	1.0 mL/min		
UV detector	210 nm		
injection volume	5 μL		

Mixture of 21 (*cis*/*trans* = 26:74) HPLC spectrum

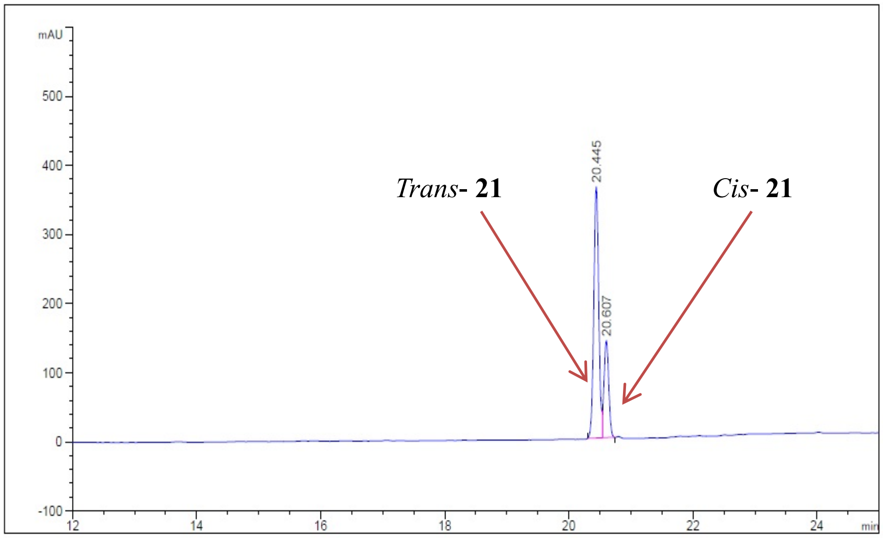

#	|min|		|min|	|mAU*s|	|mAU|	%
1	20.445	MF	0.0920	2012.69348	364.54968	73.5220
2	20.607	FM	0.0859	724.84619	140.70404	26.4780

HPLC spectrum: with CH_2Cl_2 as solvent under the comparable conditions

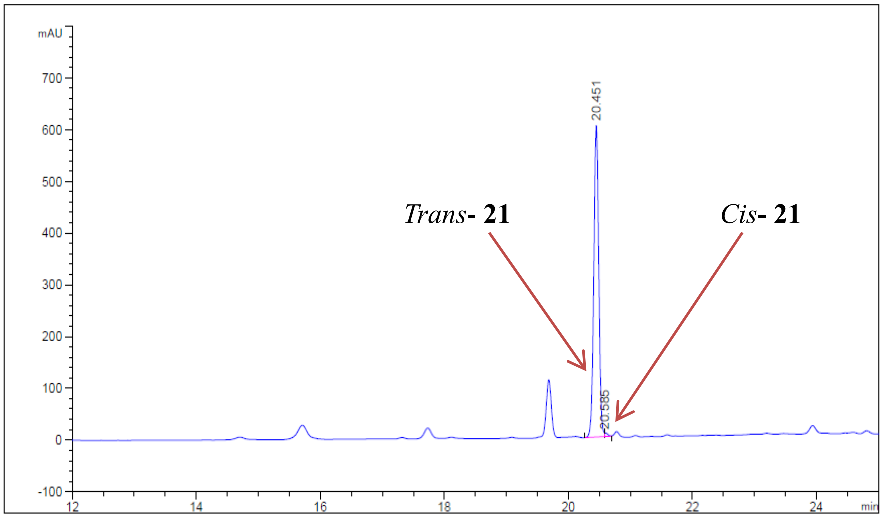

#	[min]		[min]	[mAU*s]	[mAU]	%
1	20.451	MF	0.0919	3337.82544	605.18506	99.3092
2	20.585	FM	0.0545	23.21745	7.09984	0.6908

6. Copies of NMR spectra

*^{1}H NMR ($CDCl_3$, 400 MHz) of 2-(2-Bromophenyl)ethan-1-ol **(11a)***

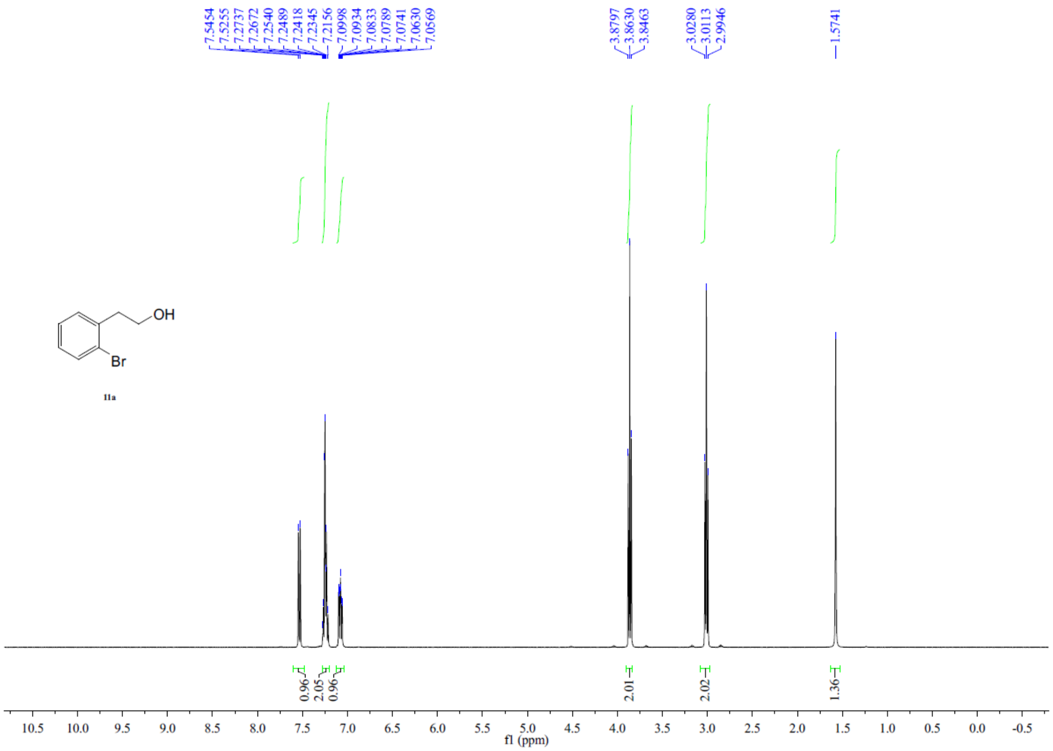

*^{13}C NMR ($CDCl_3$, 101 MHz) of 2-(2-Bromophenyl)ethan-1-ol **(11a)***

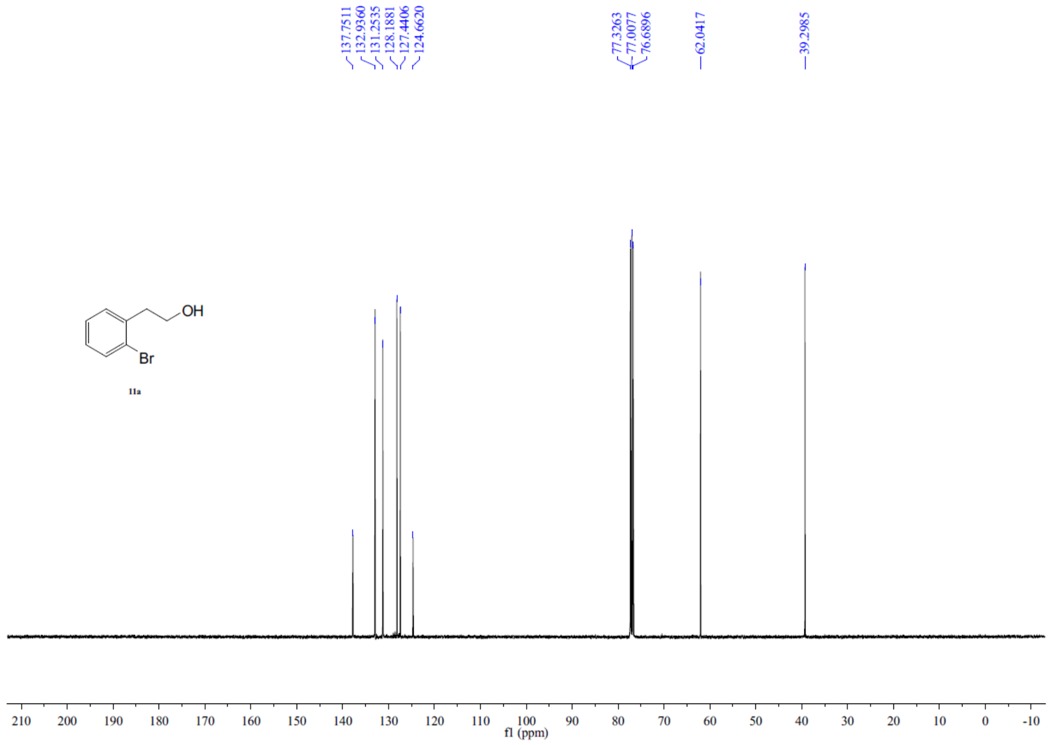

^{1}H NMR ($CDCl_3$, 400 MHz) of 2-(2-Bromo-4-methoxyphenyl)ethan-1-ol ***(11b)***

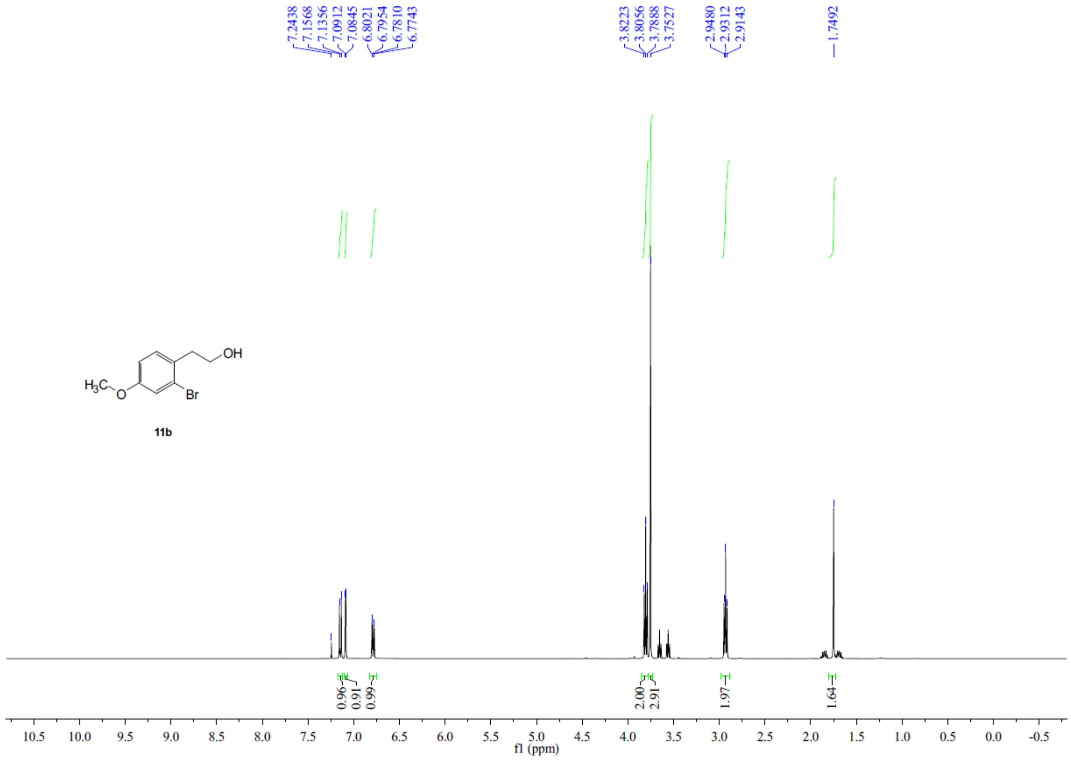

*^{13}C NMR ($CDCl_3$, 101 MHz) of 2-(2-Bromo-4-methoxyphenyl)ethan-1-ol (**11b**)*

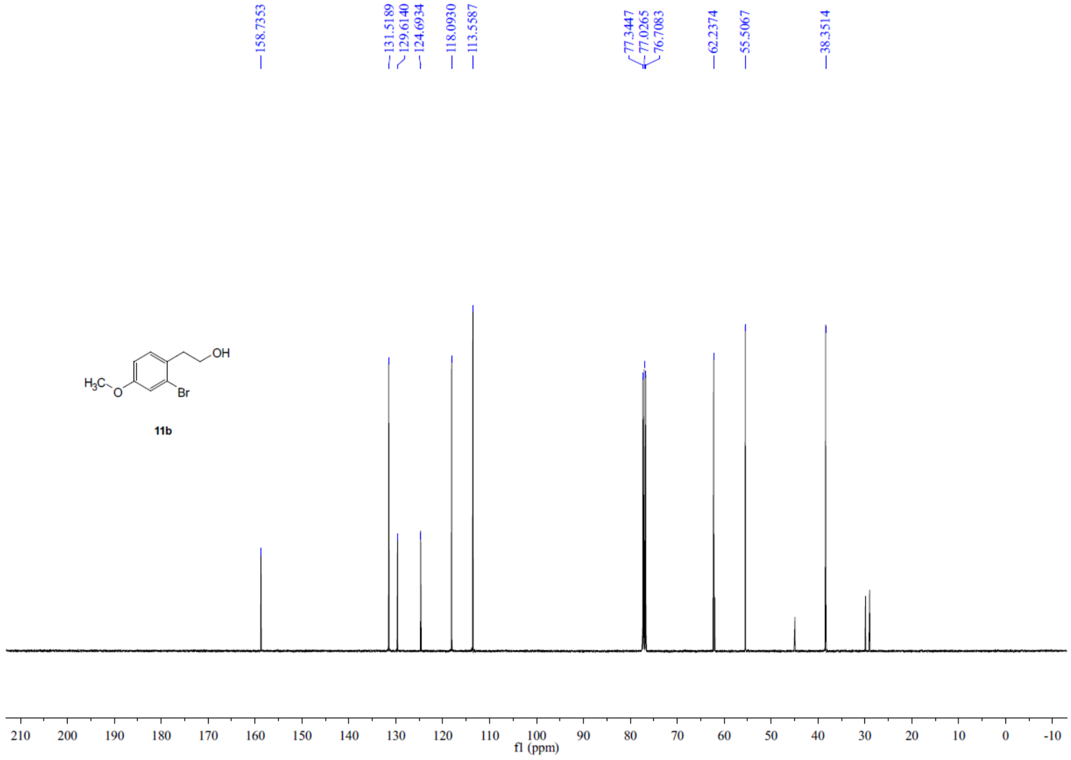

^{1}H NMR (CDCl$_3$, 400 MHz) of 2-(2-Bromo-4-chlorophenyl)ethan-1-ol ***(11c)***

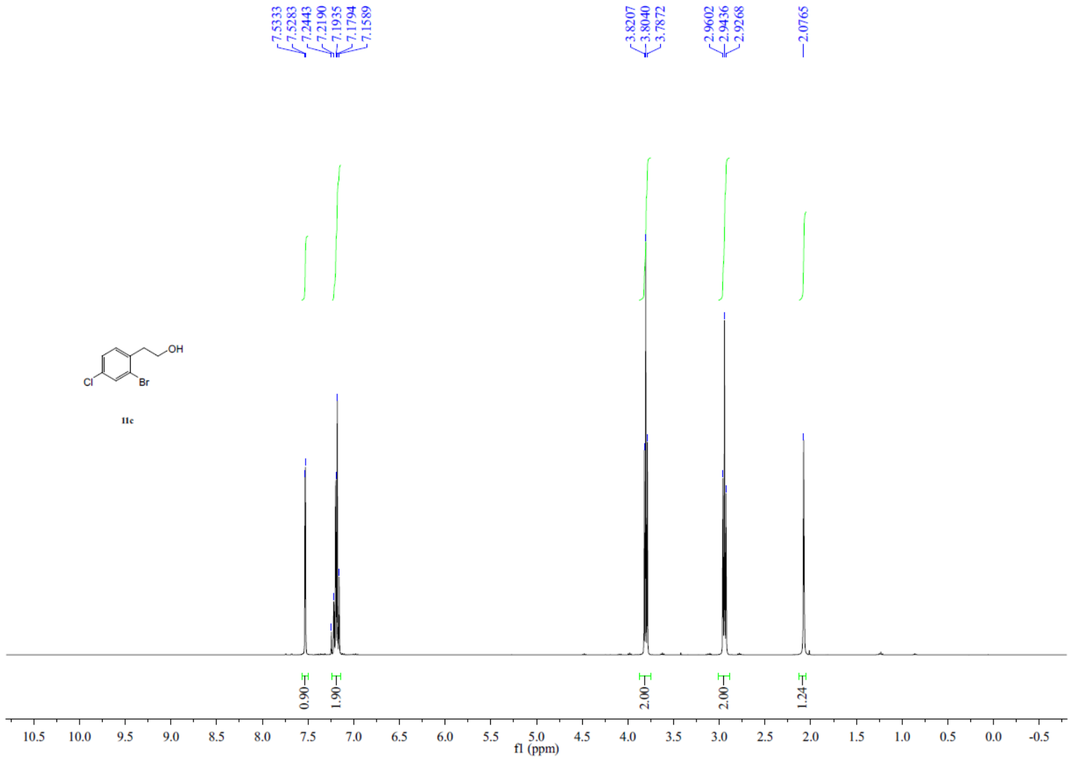

*^{13}C NMR (CDCl$_3$, 101 MHz)of 2-(2-Bromo-4-chlorophenyl)ethan-1-ol (**11c**)*

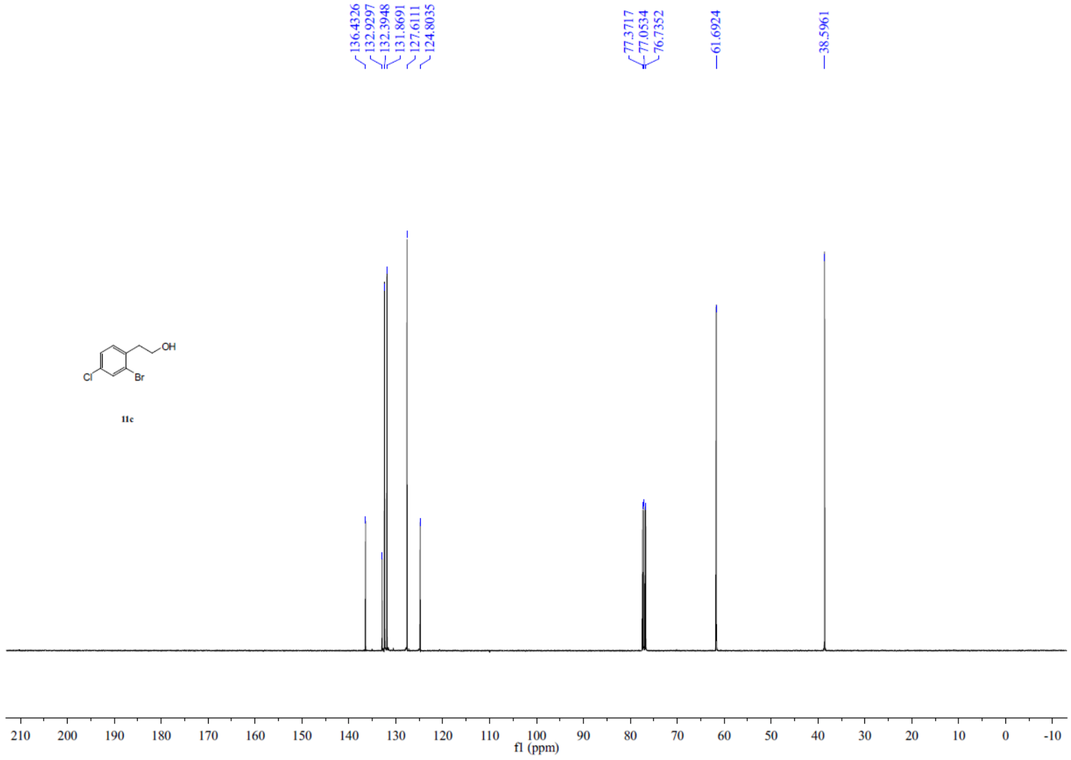

*(**11e**) ^{1}H NMR ($CDCl_3$, 400 MHz) of 2-(2-Bromophenyl)propan-1-ol*

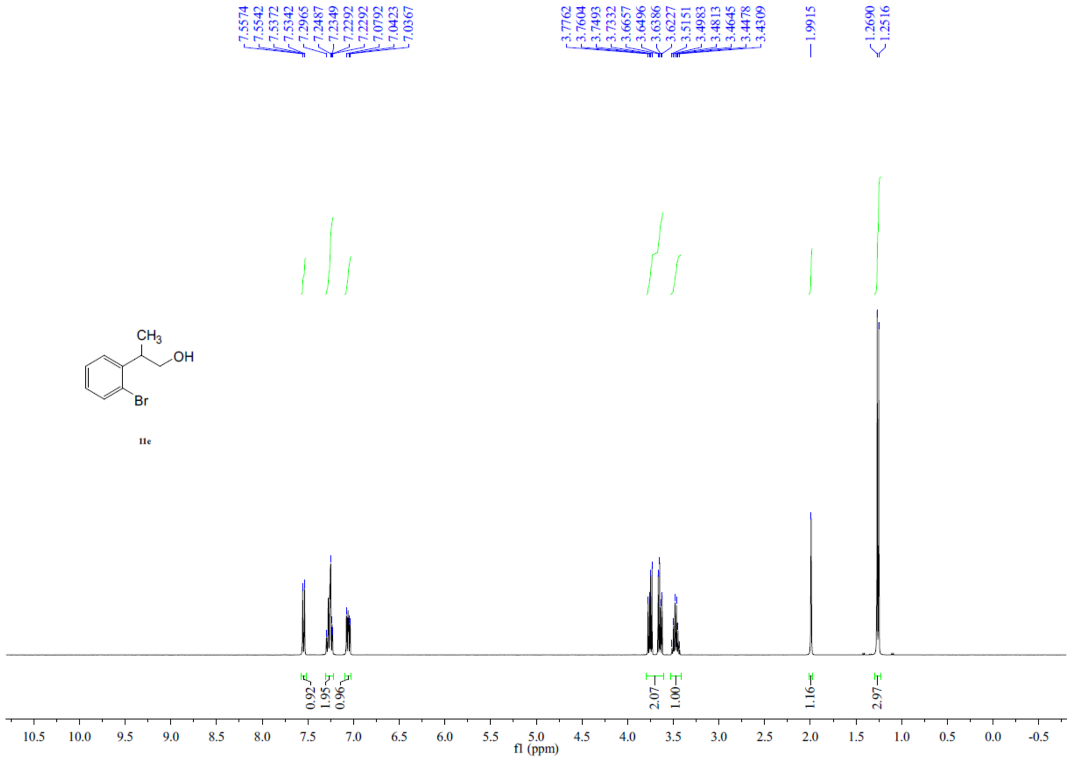

*^{13}C NMR ($CDCl_3$, 101 MHz) of 2-(2-Bromophenyl)propan-1-ol (**11e**)*

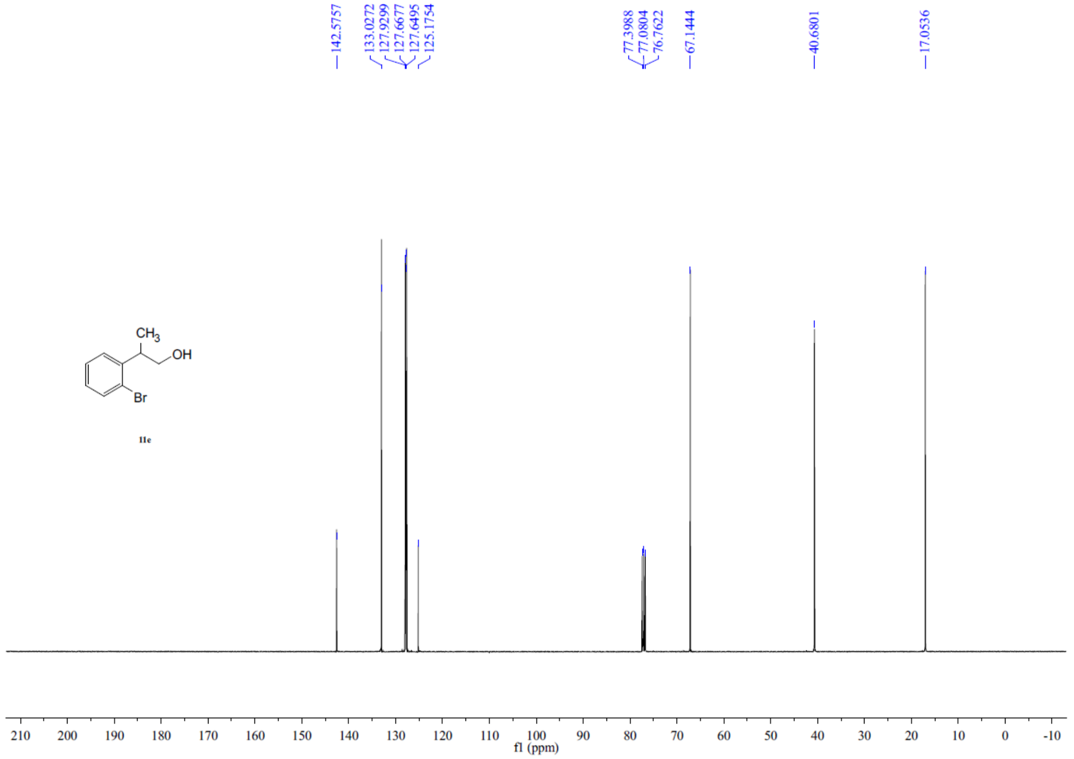

^{1}H NMR ($CDCl_3$, 400 MHz) of 2-(1-Bromonaphthalen-2-yl)ethan-1-ol (**11h**)

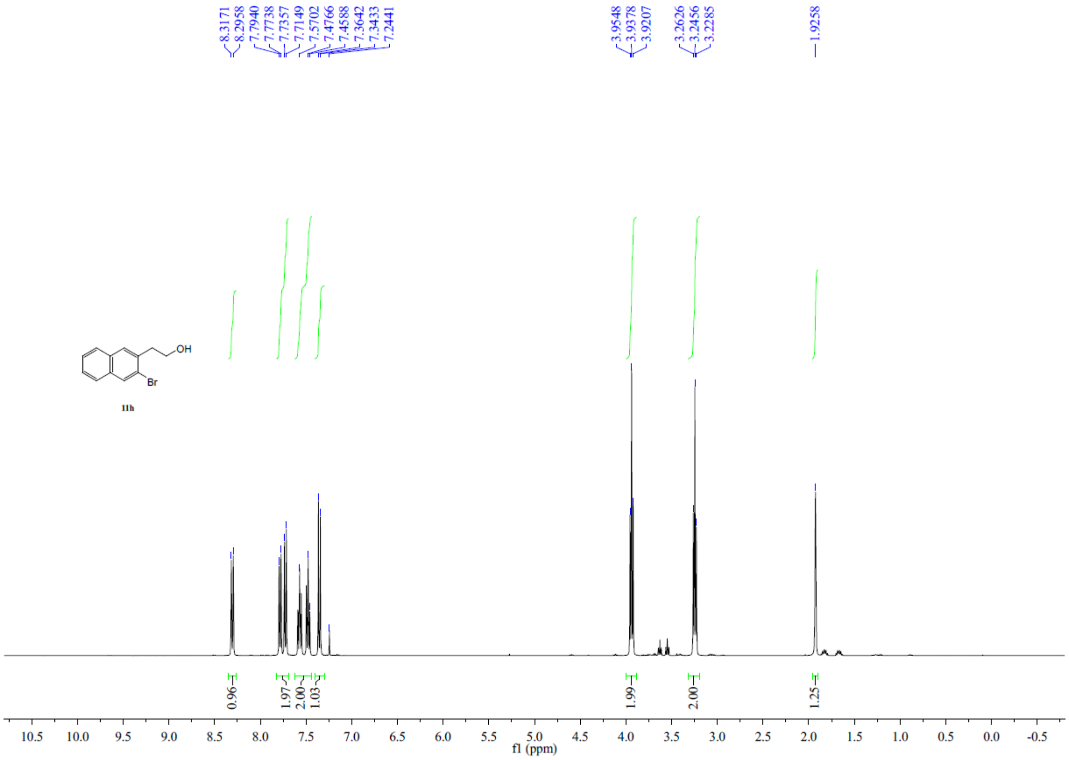

^{13}C NMR ($CDCl_3$, 101 MHz) of 2-(1-Bromonaphthalen-2-yl)ethan-1-ol (**11h**)

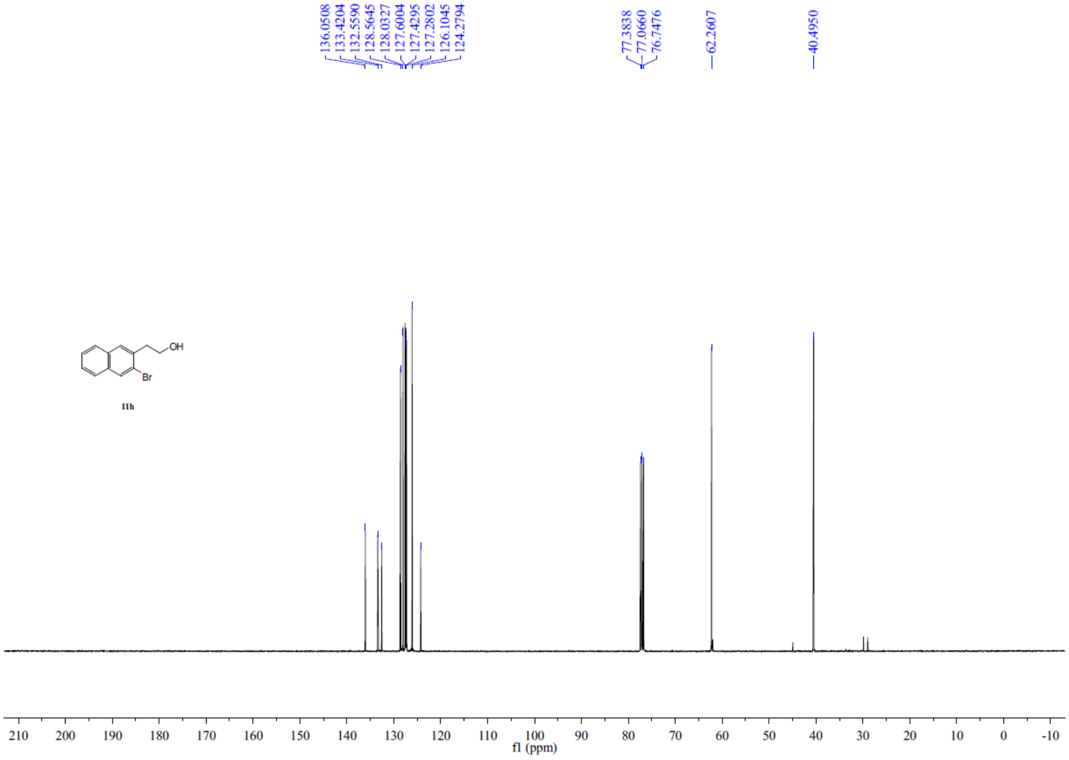

^1H *NMR ($CDCl_3$, 400 MHz) of 2-(2-Bromo-4-nitrophenyl)ethan-1-ol (**11d**)*

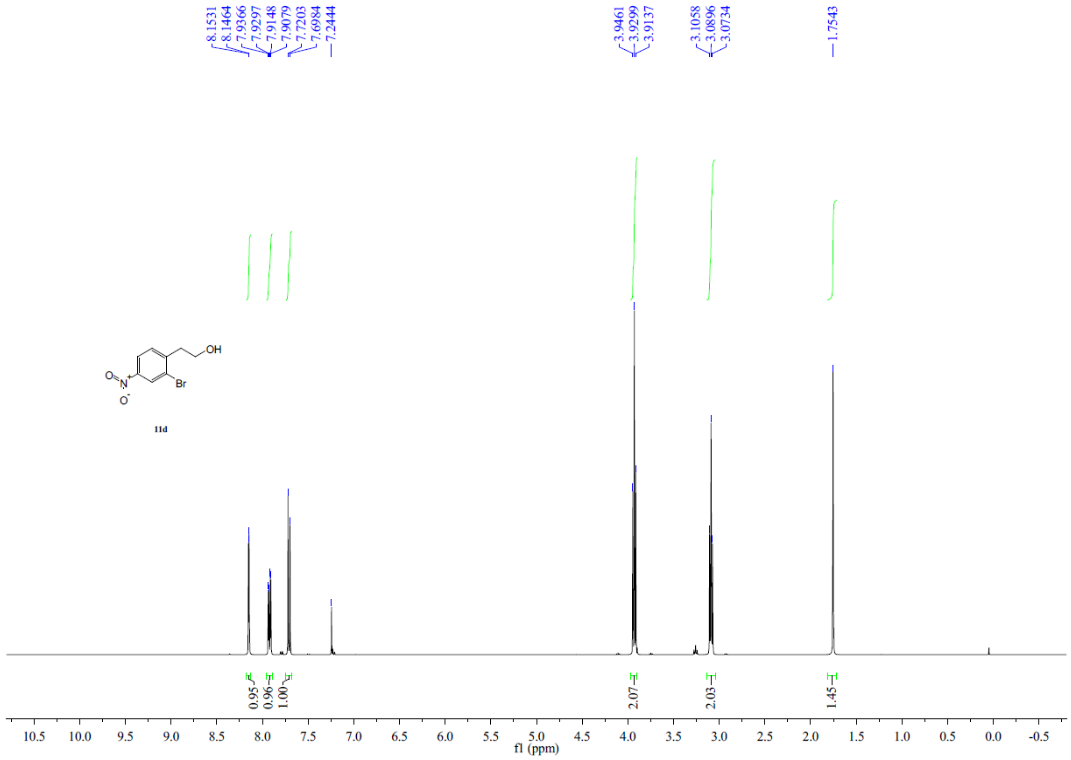

^{13}C *NMR ($CDCl_3$, 101 MHz) of 2-(2-Bromo-4-nitrophenyl)ethan-1-ol (**11d**)*

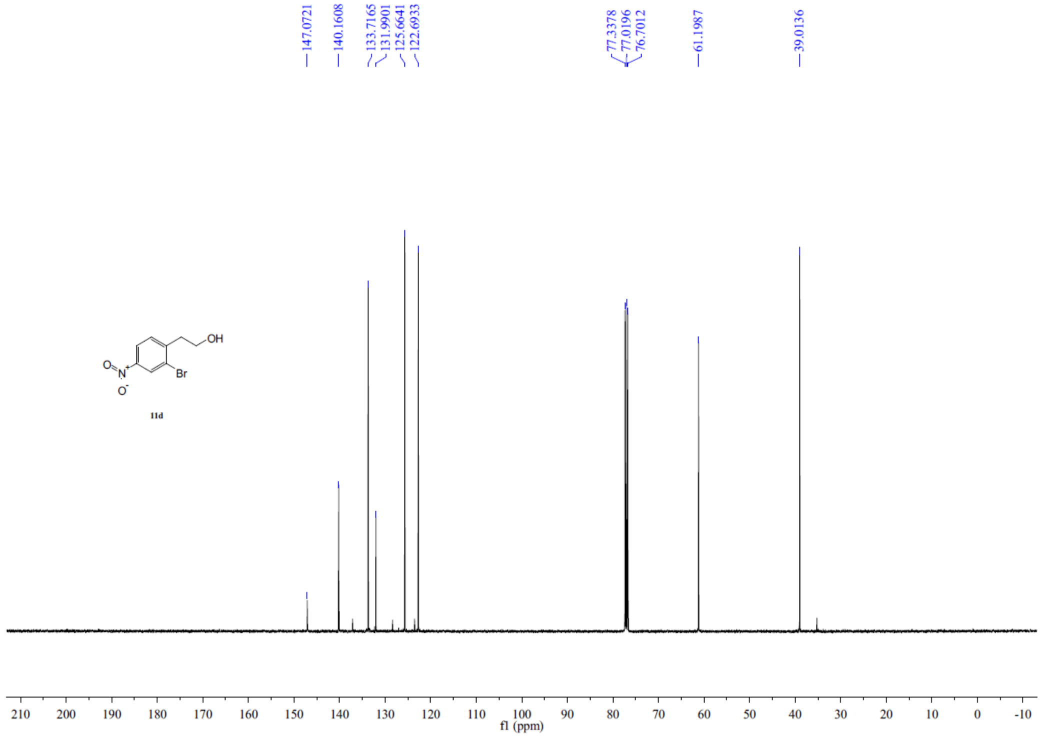

*^{1}H NMR (CDCl$_3$, 400 MHz) of 2-(2-Vinylphenyl)ethan-1-ol (**12a**)*

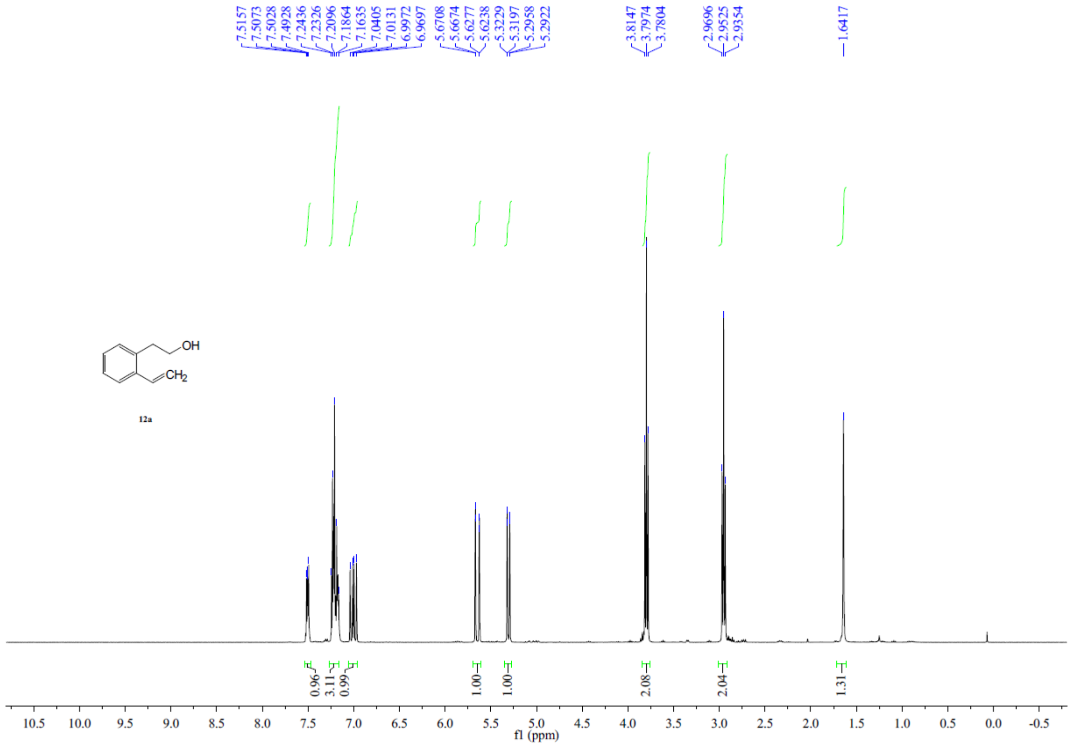

*^{13}C NMR (CDCl$_3$, 101 MHz) of 2-(2-Vinylphenyl)ethan-1-ol (**12a**)*

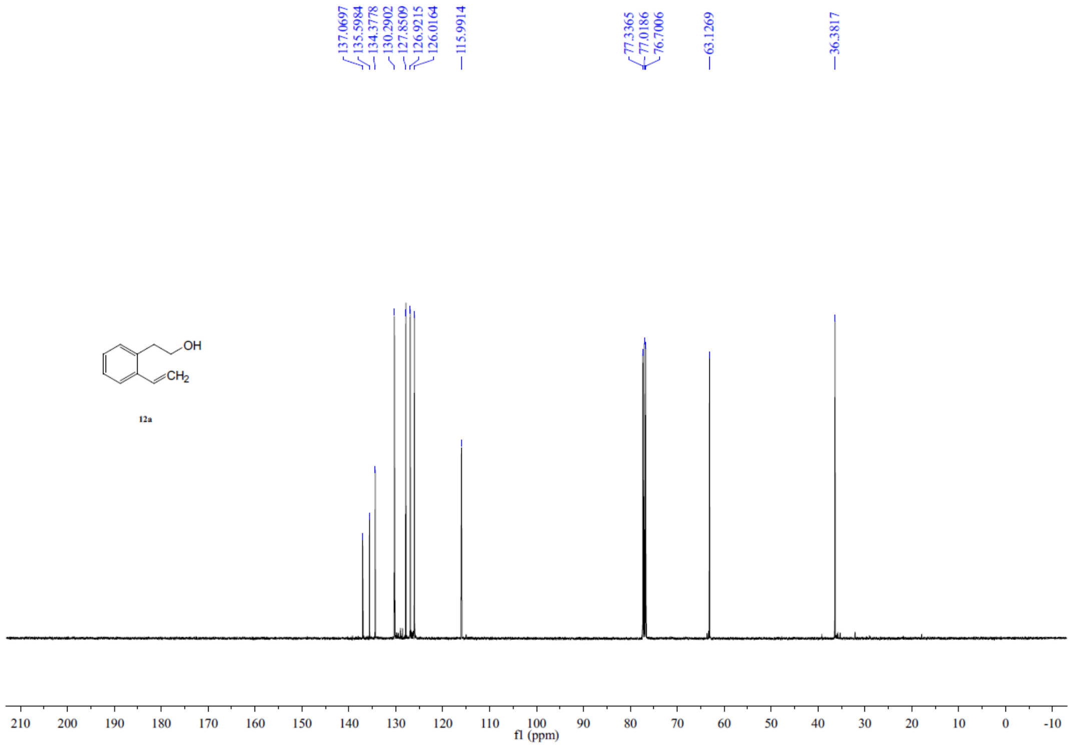

^1H NMR ($CDCl_3$, 400 MHz) of 2-(4-Methoxy-2-vinylphenyl)ethan-1-ol (**12b**)

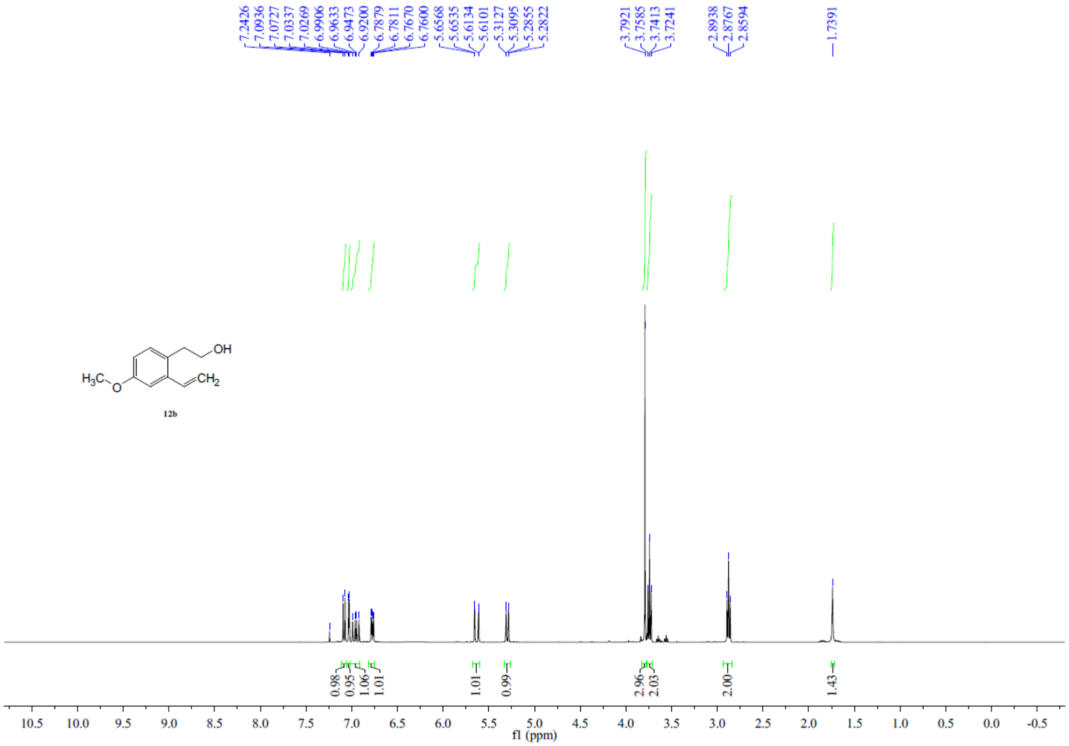

^{13}C NMR ($CDCl_3$, 101 MHz) of 2-(4-Methoxy-2-vinylphenyl)ethan-1-ol (**12b**)

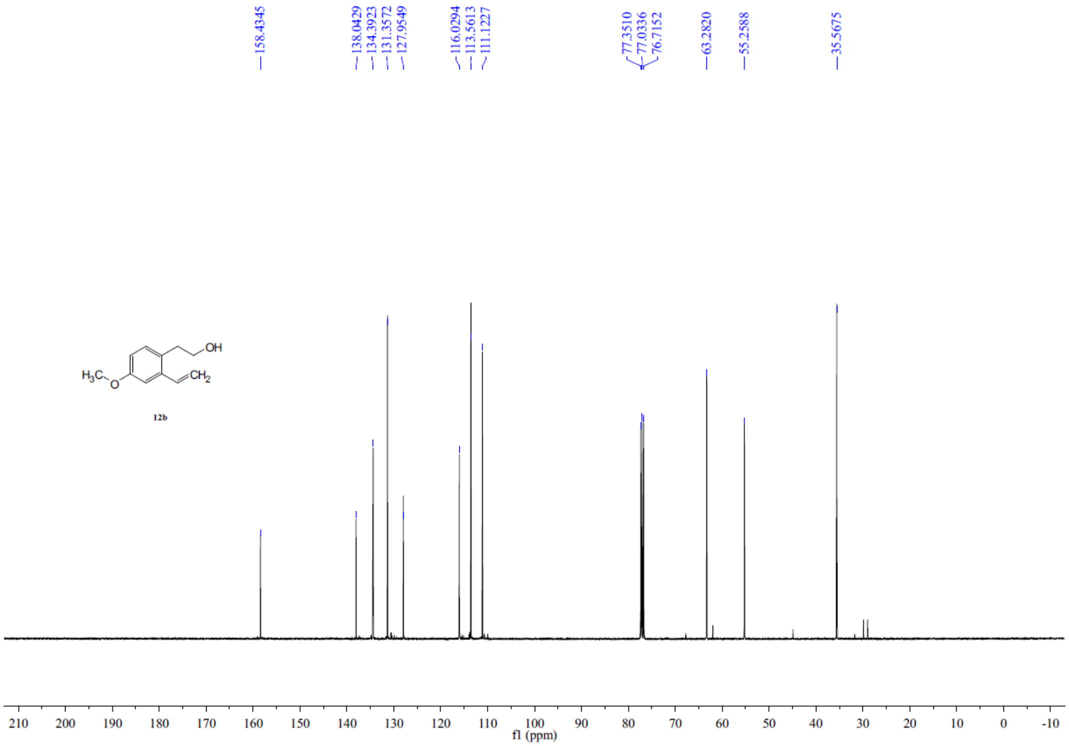

^{1}H NMR ($CDCl_3$, 400 MHz) of 2-(4-Chloro-2-vinylphenyl)ethan-1-ol (**12c**)

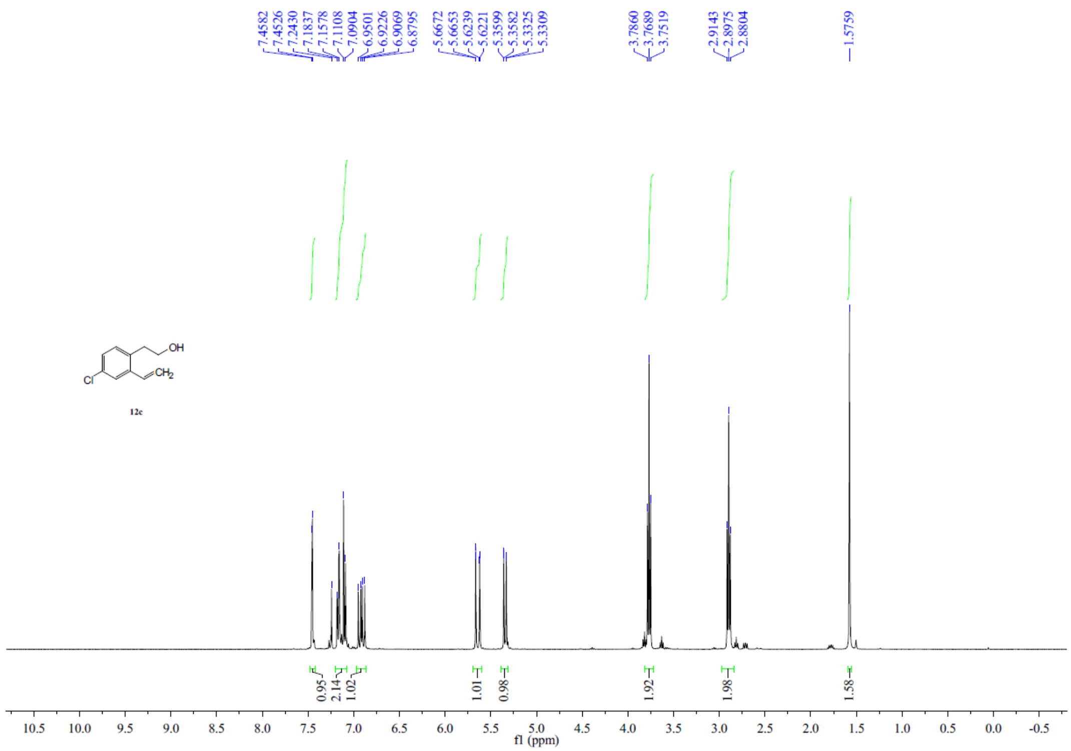

^{13}C NMR ($CDCl_3$, 101 MHz) of 2-(4-Chloro-2-vinylphenyl)ethan-1-ol (**12c**)

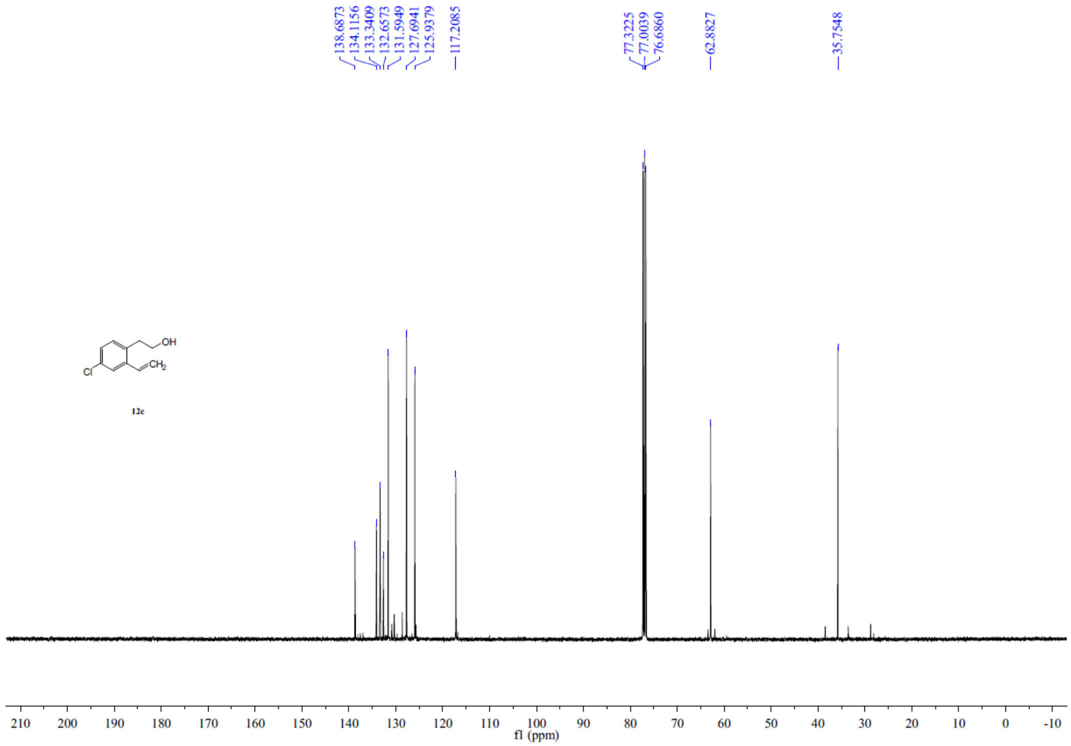

*^{1}H NMR ($CDCl_3$, 400 MHz) of 2-(4-Nitro-2-vinylphenyl)ethan-1-ol (**12d**)*

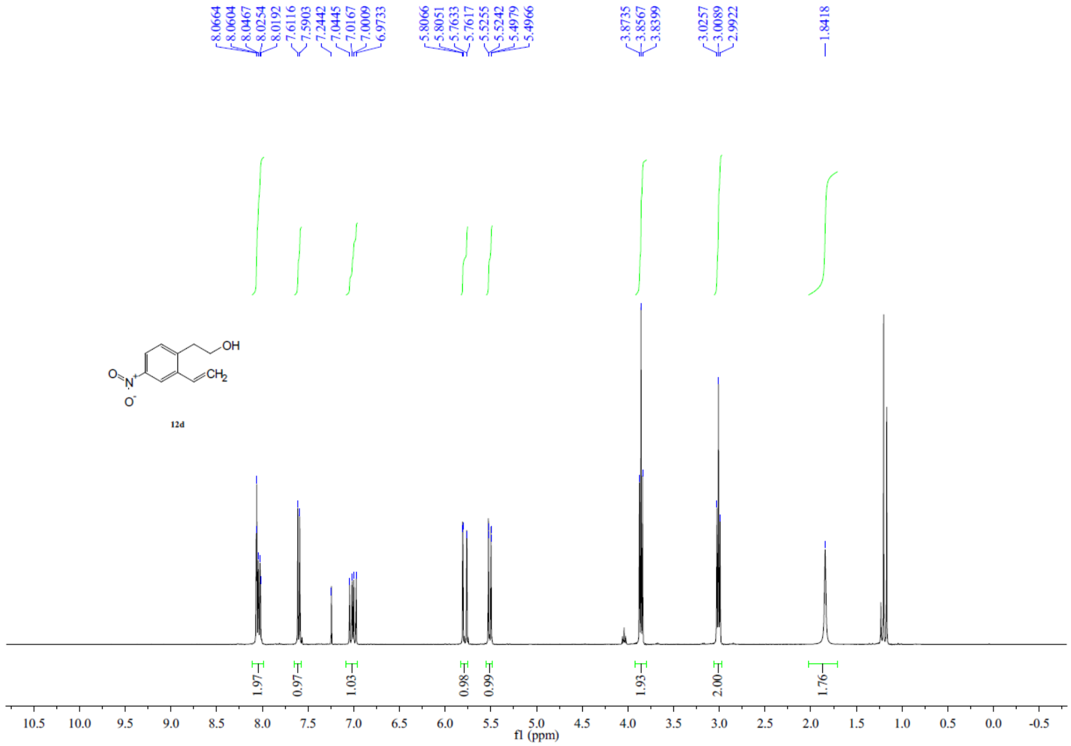

*^{13}C NMR ($CDCl_3$, 101 MHz) of 2-(4-Nitro-2-vinylphenyl)ethan-1-ol (**12d**)*

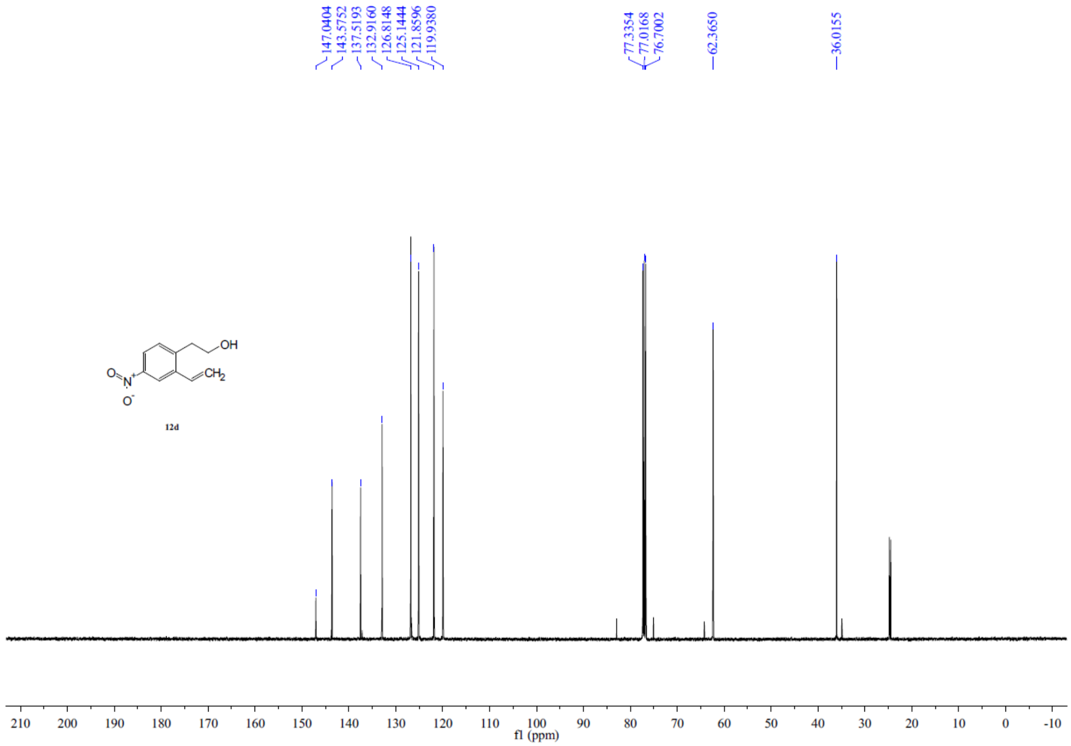

^1H NMR ($CDCl_3$, 400 MHz) of 2-(2-Vinylphenyl)propan-1-ol (***12e***)

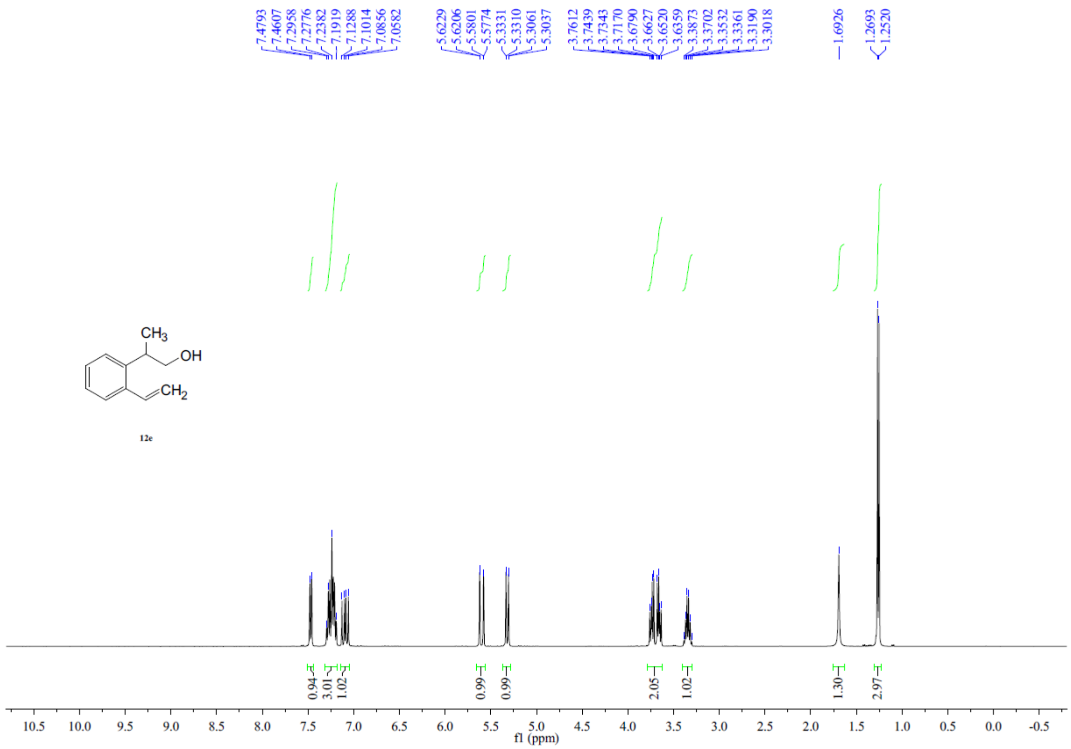

^{13}C NMR ($CDCl_3$, 101 MHz) of 2-(2-Vinylphenyl)propan-1-ol (***12e***)

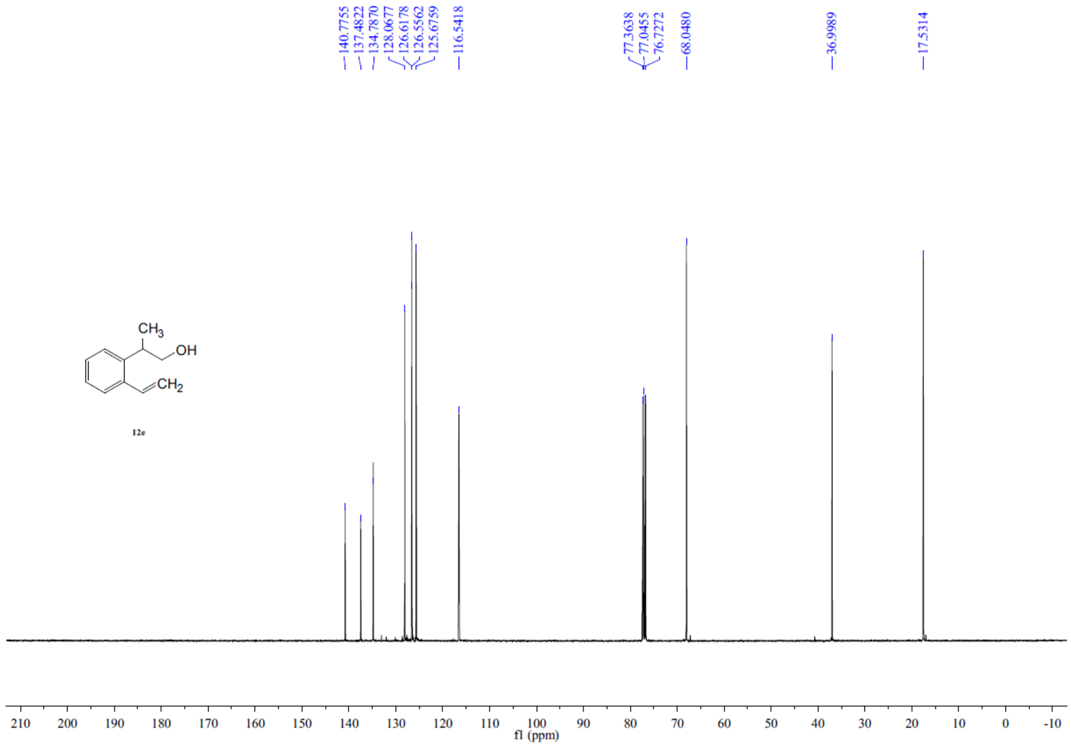

^{1}H NMR ($CDCl_3$, 400 MHz) of 2-(2-(Prop-1-en-2-yl)phenyl)ethan-1-ol (**12f**)

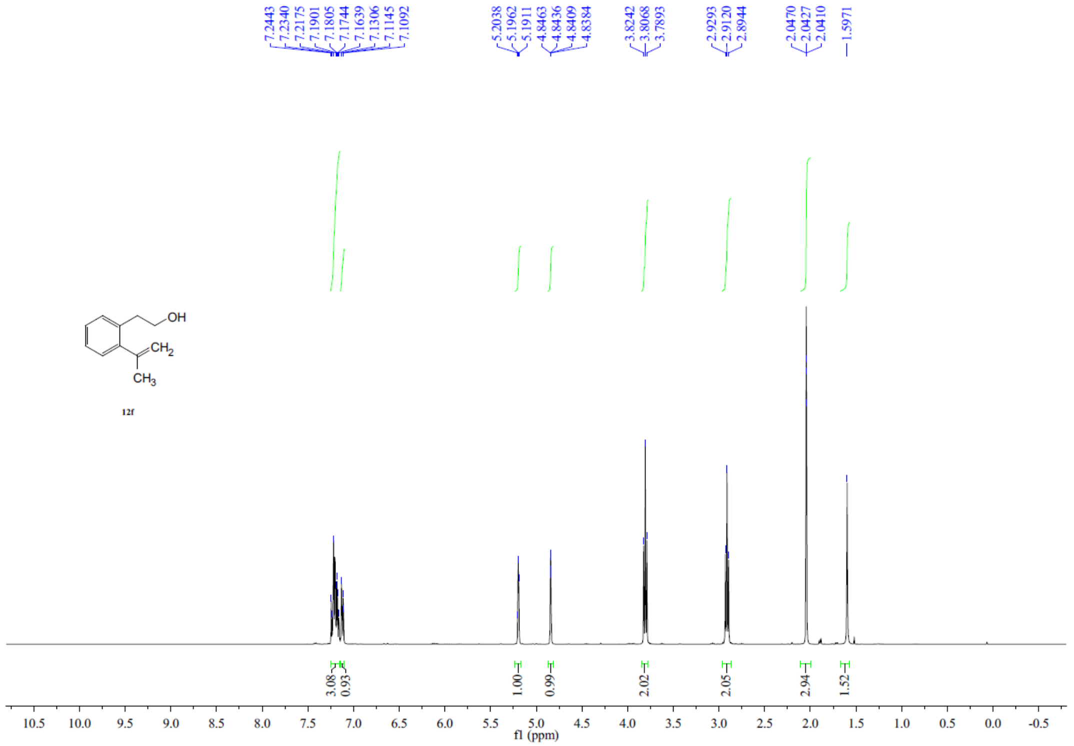

^{13}C NMR ($CDCl_3$, 101 MHz) of 2-(2-(Prop-1-en-2-yl)phenyl)ethan-1-ol (**12f**)

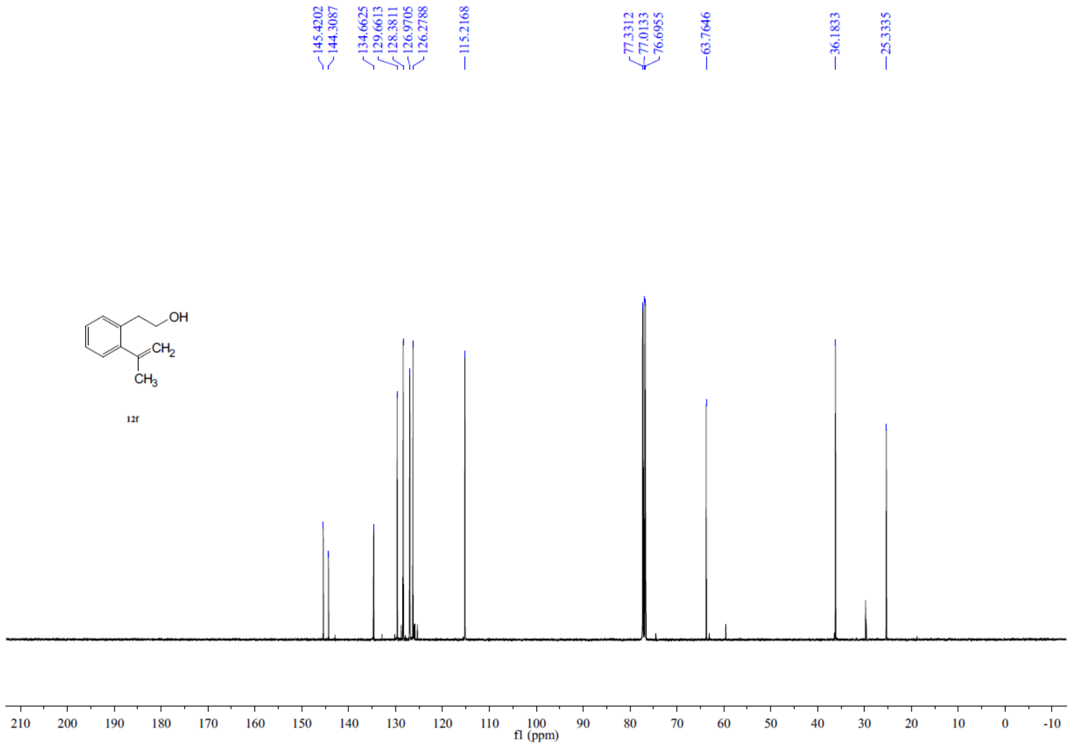

^{1}H NMR ($CDCl_3$, 400 MHz) of 2-(2-(1-phenylvinyl)phenyl)ethan-1-ol (**12g**)

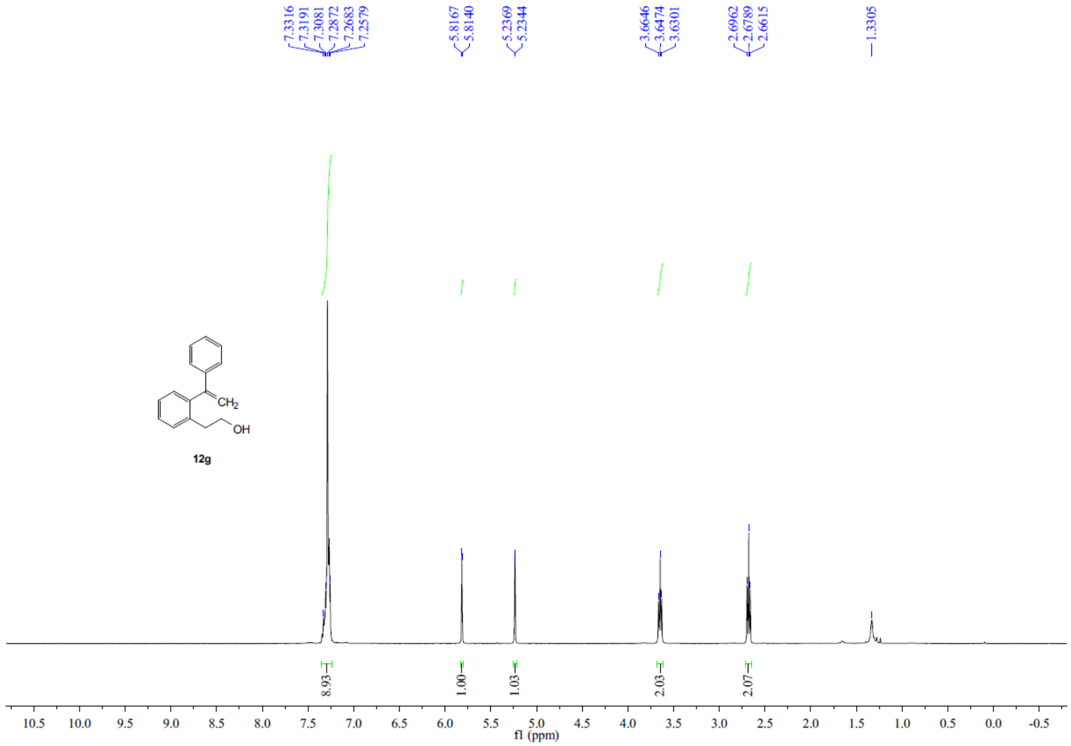

^{13}C NMR ($CDCl_3$, 101 MHz) of 2-(2-(1-Phenylvinyl)phenyl)ethan-1-ol (**12g**)

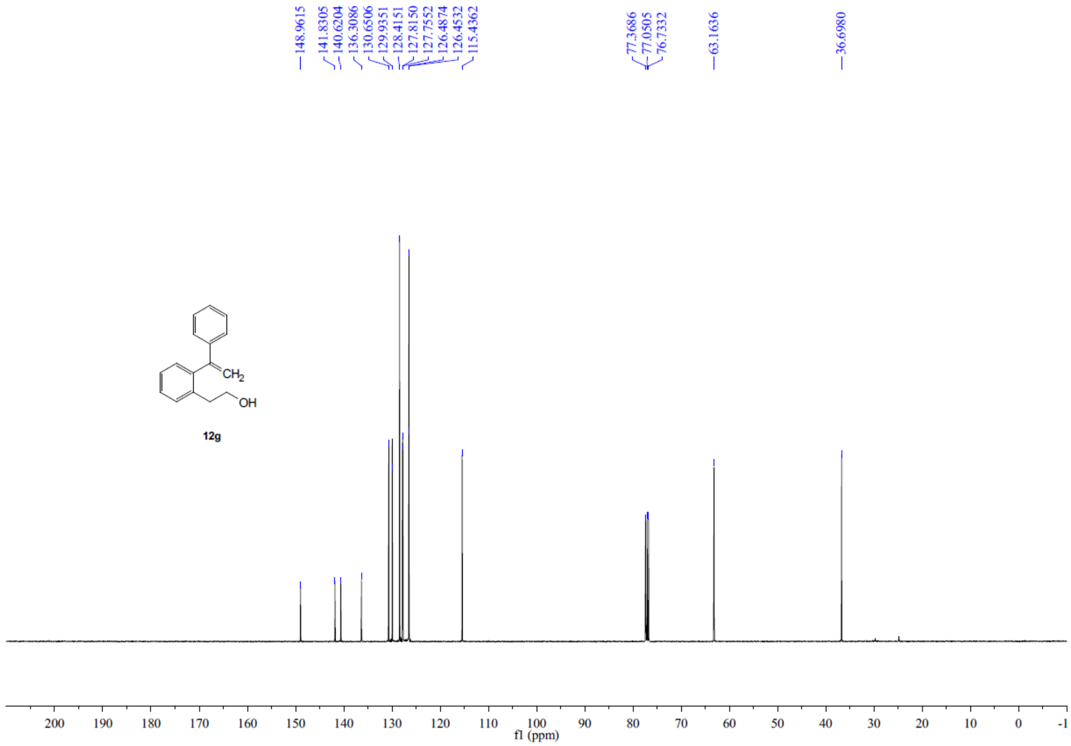

^{1}H NMR ($CDCl_3$, 400 MHz) of 2-(1-Vinylnaphthalen-2-ylethan-1-ol (**12h**)

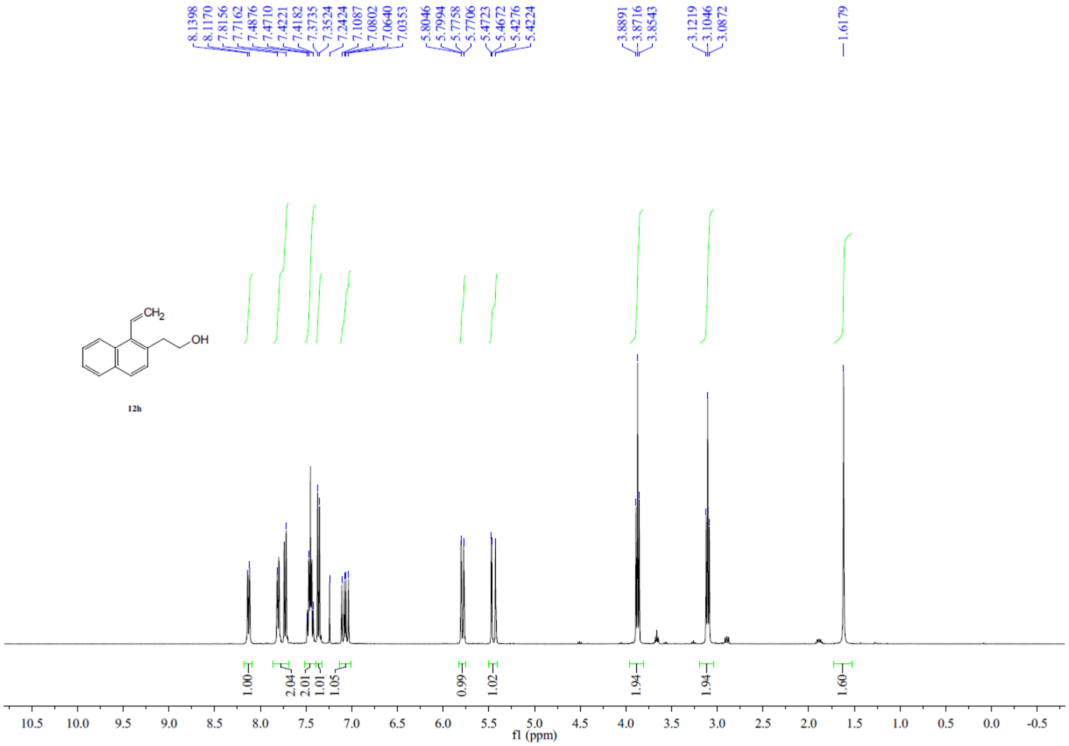

^{13}C NMR ($CDCl_3$, 101 MHz) of 2-(1-Vinylnaphthalen-2-yl)ethan-1-ol (**12h**)

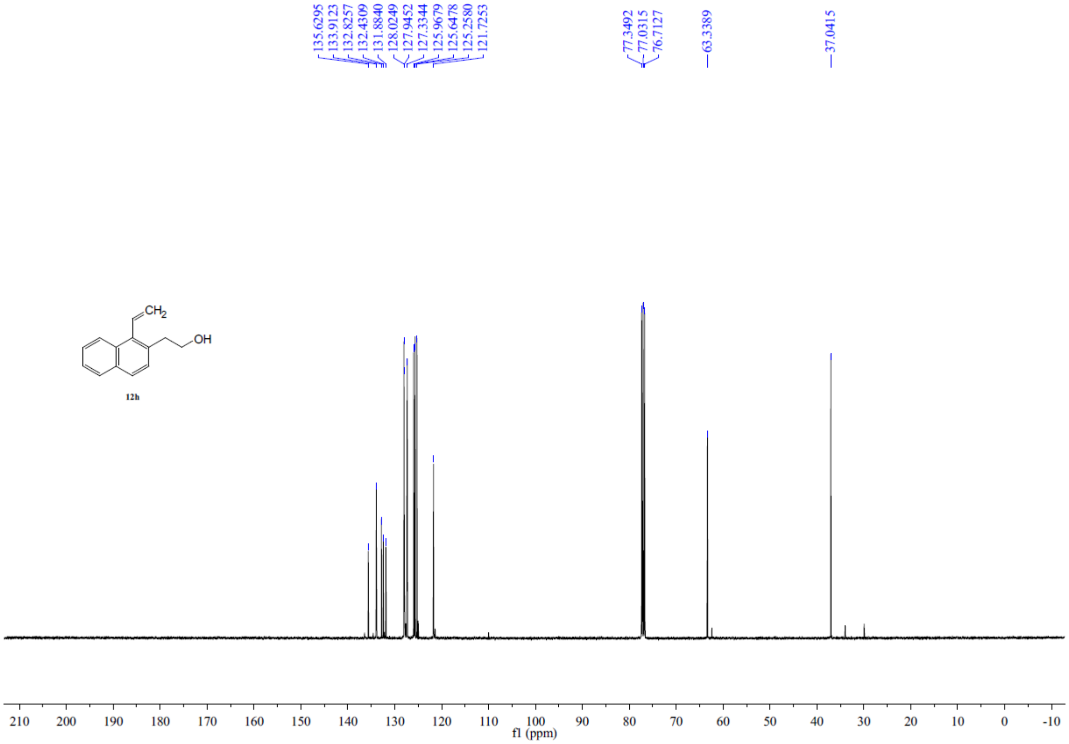

^1H NMR ($CDCl_3$, 400 MHz) of 2-(2-Vinylphenyl)acetaldehyde (***13a***)

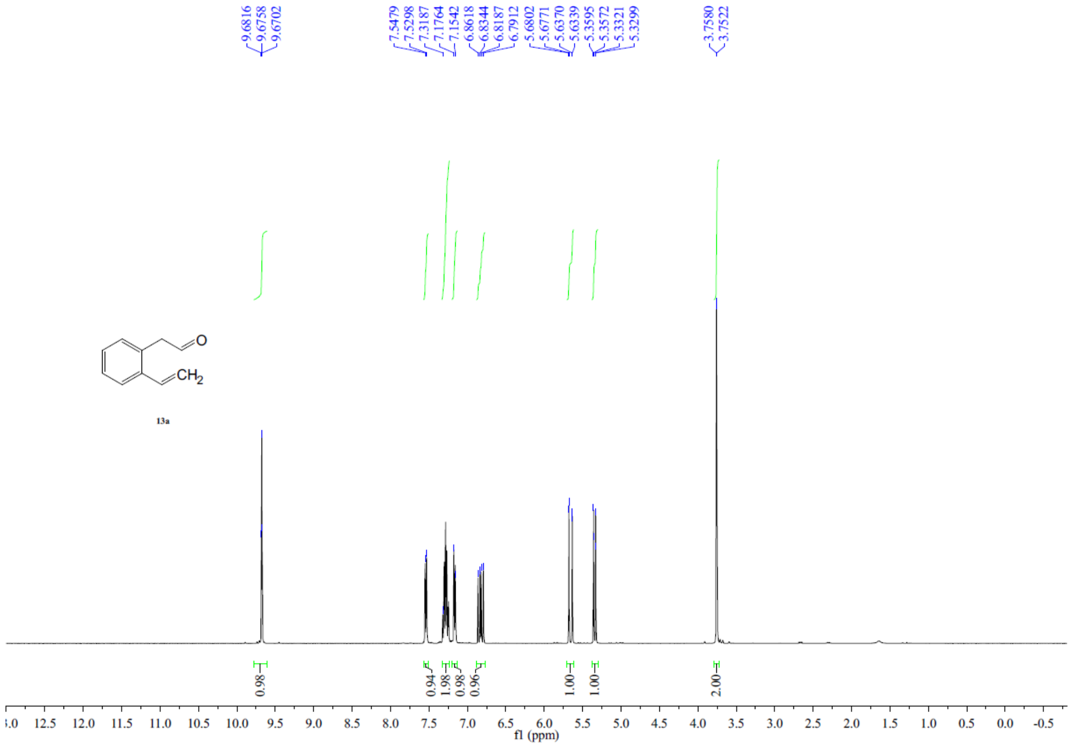

^{13}C NMR ($CDCl_3$, 101 MHz) of 2-(2-Vinylphenyl)acetaldehyde (***13a***)

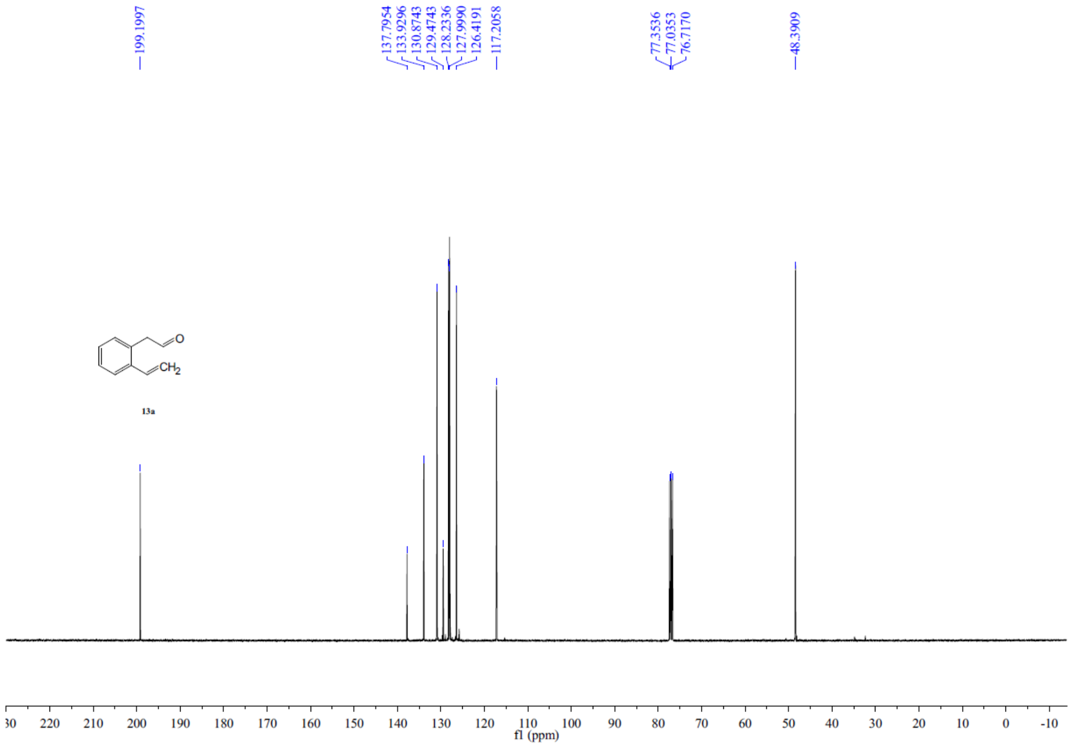

^{1}H NMR ($CDCl_3$, 400 MHz) of 2-(4-Methoxy-2-vinylphenyl)acetaldehyde (**13b**)

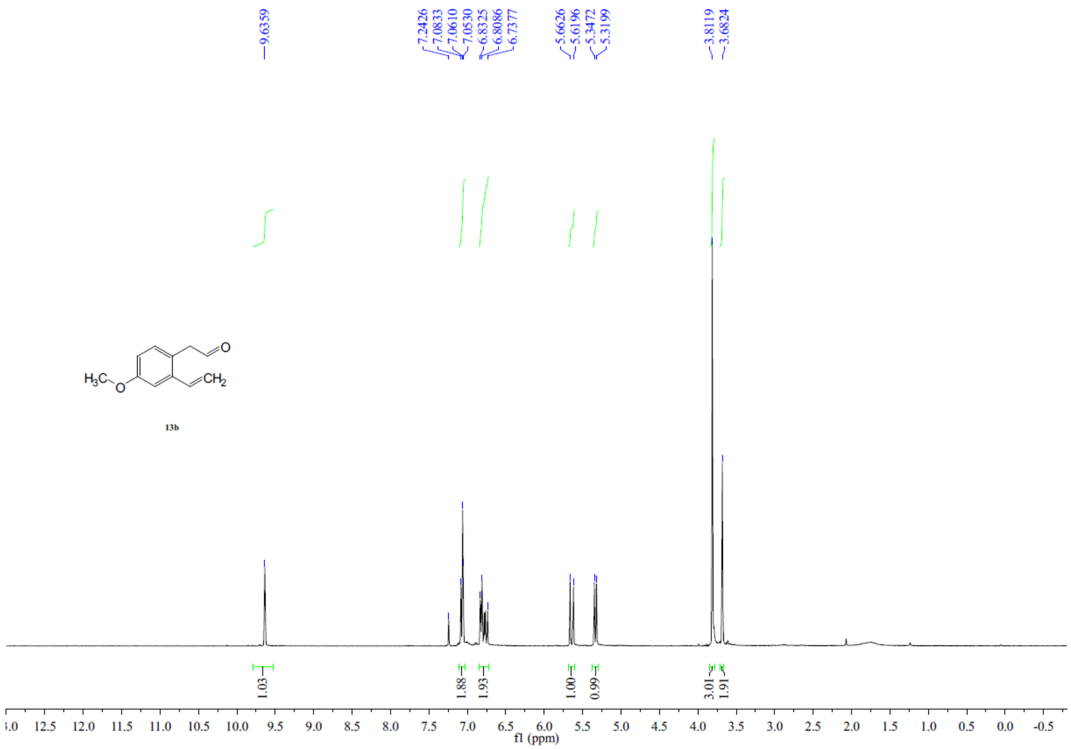

^{13}C NMR ($CDCl_3$, 101 MHz) of 2-(4-Methoxy-2-vinylphenyl)acetaldehyde (**13b**)

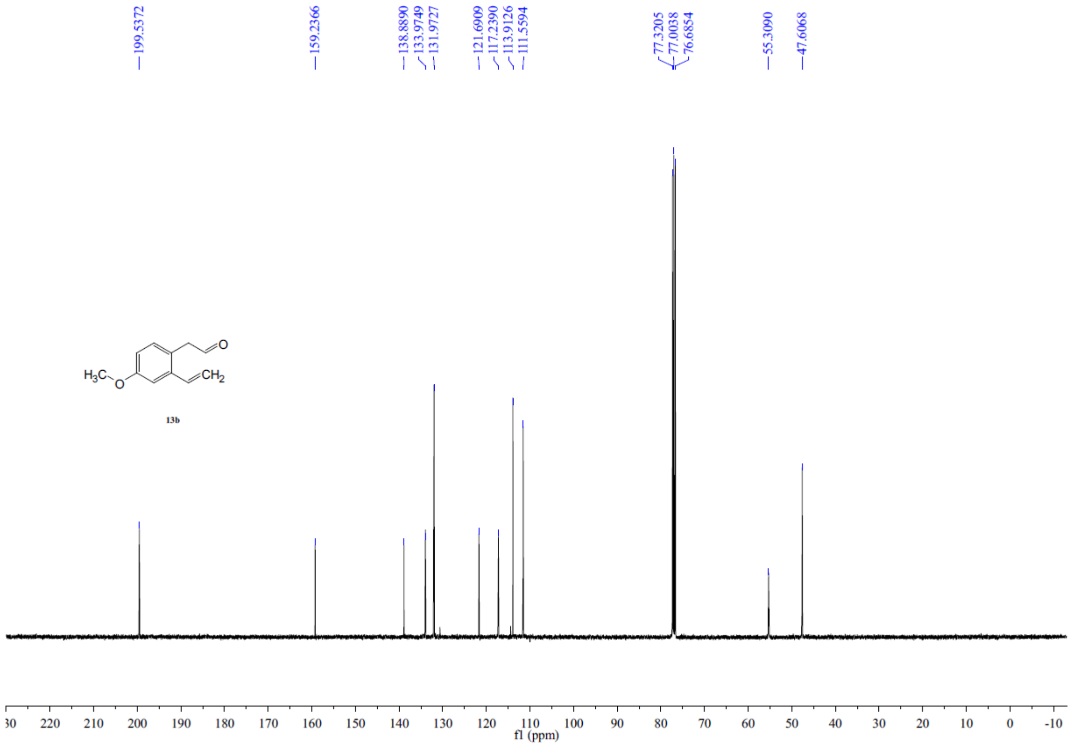

^{1}H NMR ($CDCl_3$, 400 MHz) of 2-(4-Chloro-2-vinylphenyl)acetaldehyde ***(13c)***

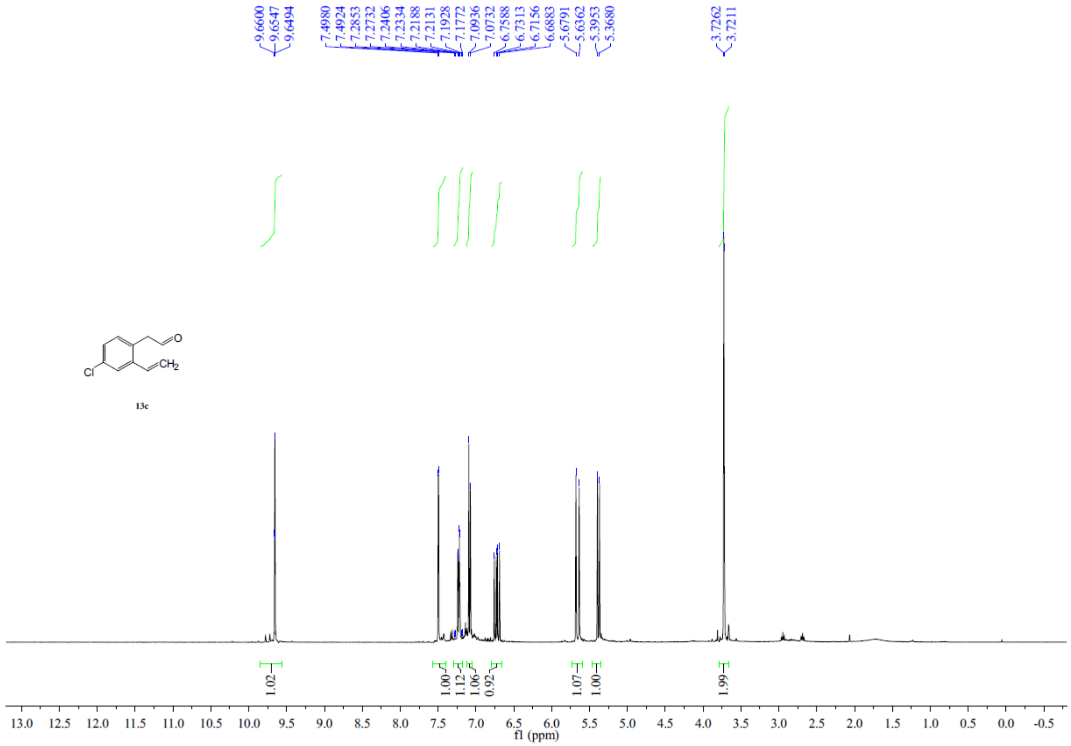

^{13}C NMR ($CDCl_3$, 101 MHz) of 2-(4-Chloro-2-vinylphenylacetaldehyde ***(13c)***

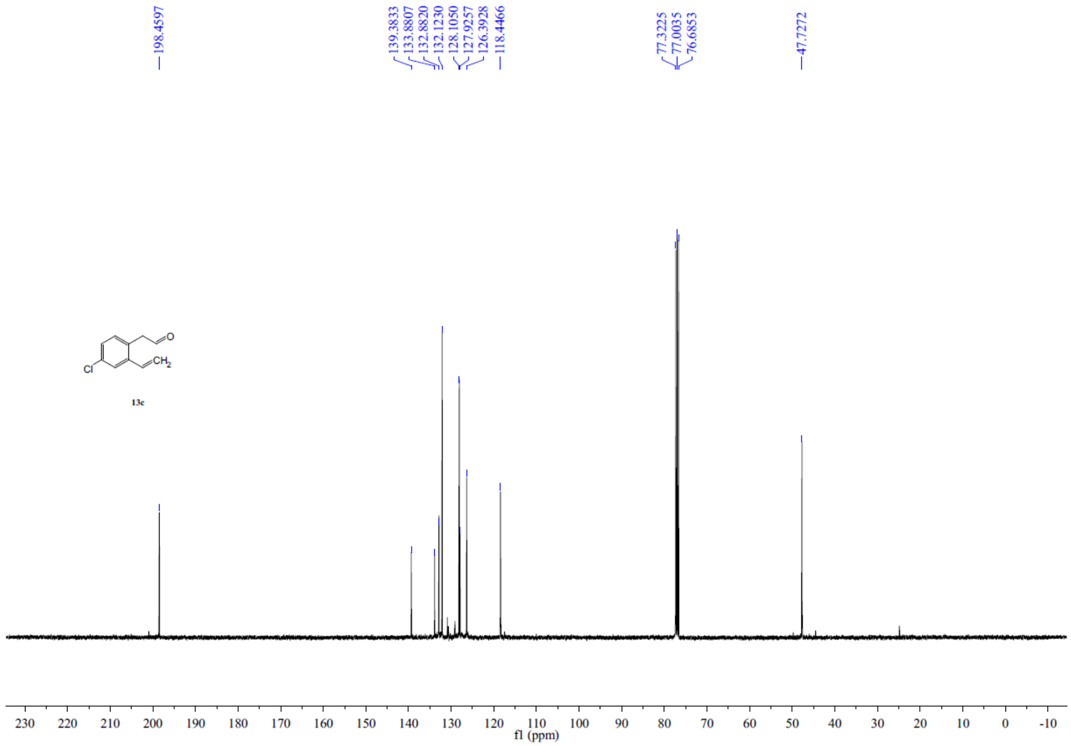

^{1}H NMR ($CDCl_3$, 400 MHz) of 2-(4-Nitro-2-vinylphenyl)acetaldehyde (**13d**)

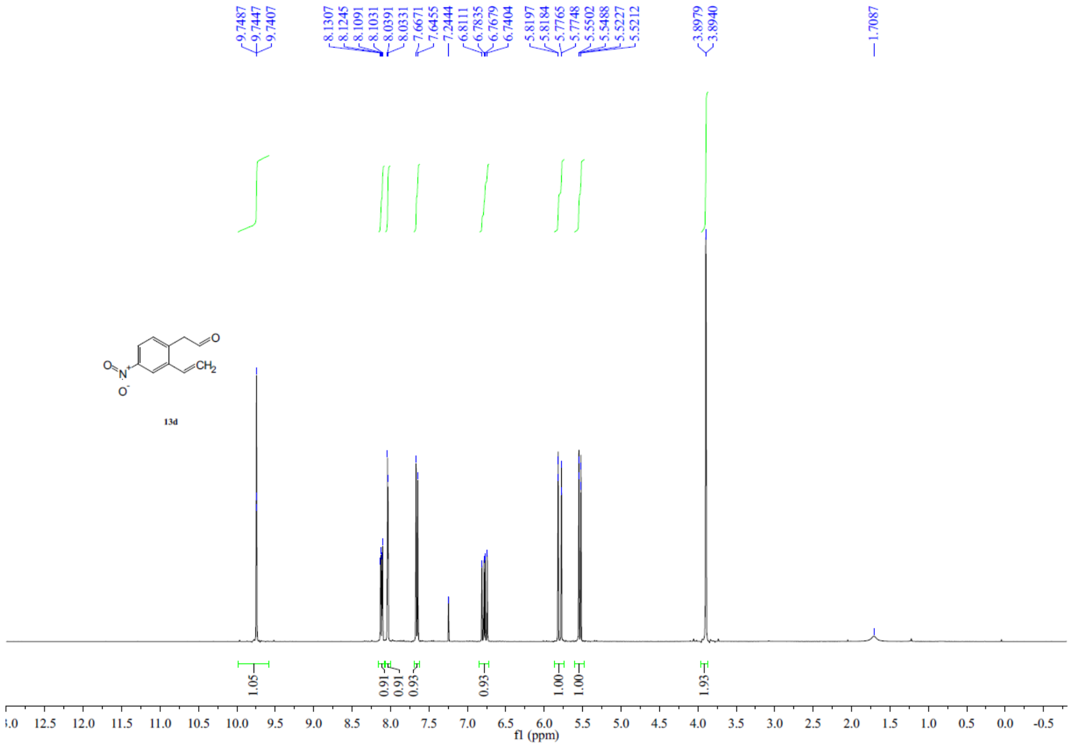

^{13}C NMR ($CDCl_3$, 101 MHz) of 2-(4-Nitro-2-vinylphenyl)acetaldehyde (**13d**)

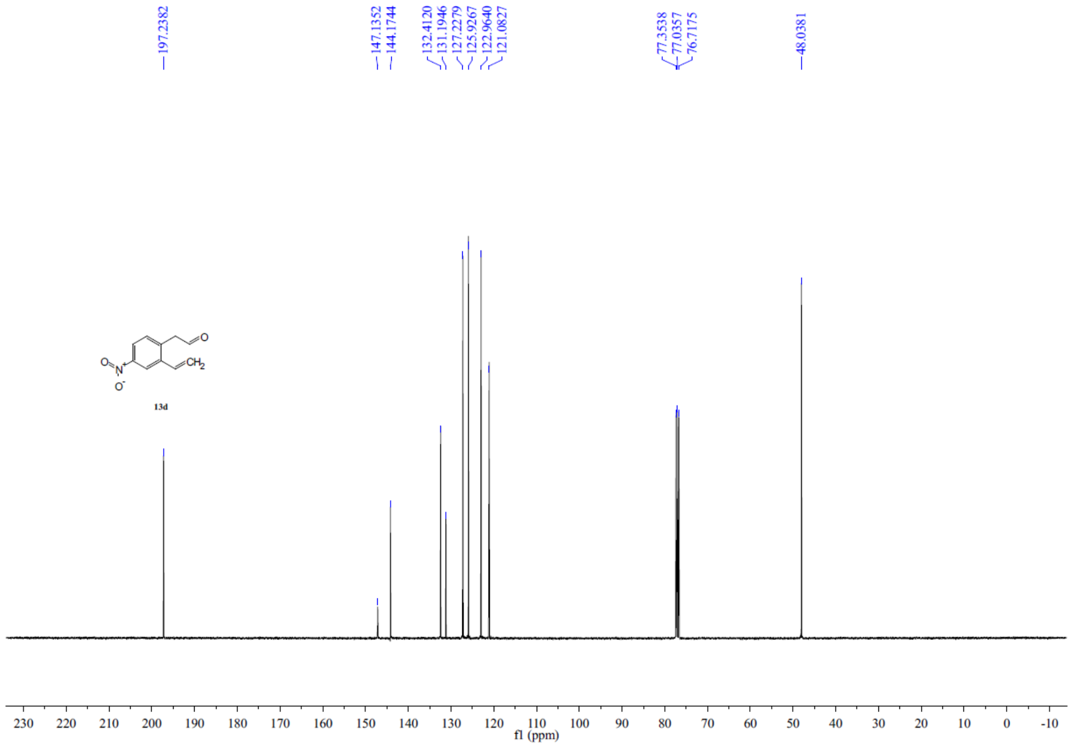

^1H NMR ($CDCl_3$, 400 MHz) of 2-(2-Vinylphenyl)propanal (**13e**)

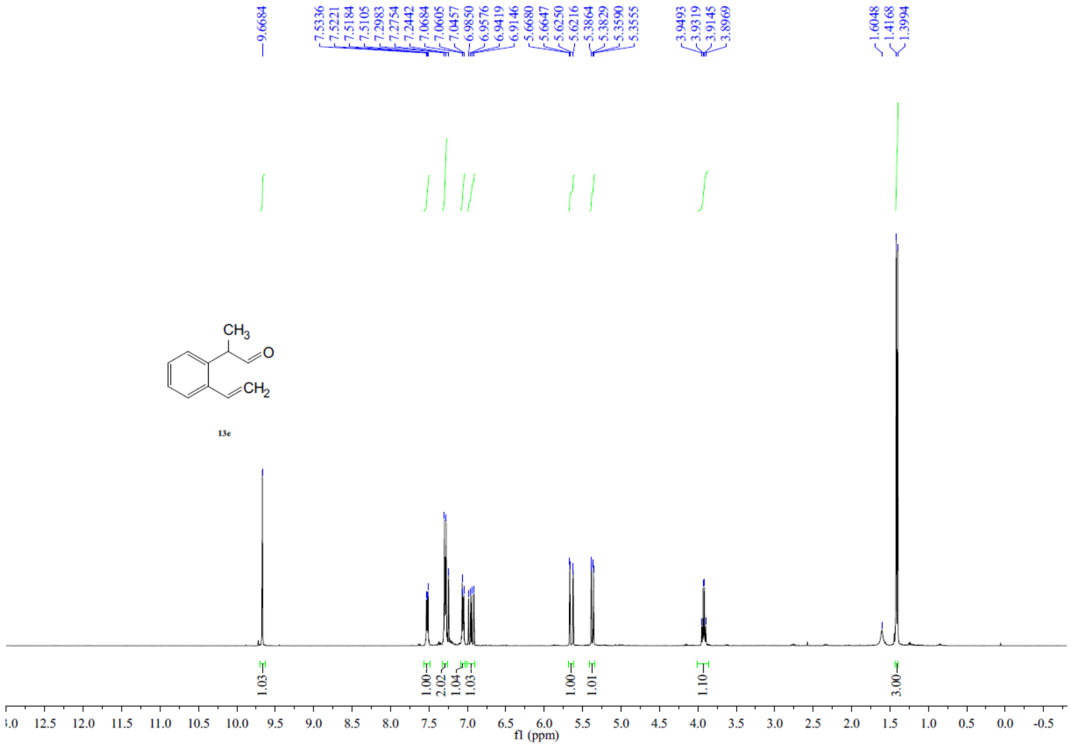

^{13}C NMR ($CDCl_3$, 101 MHz) of 2-(2-Vinylphenyl)propanal (**13e**)

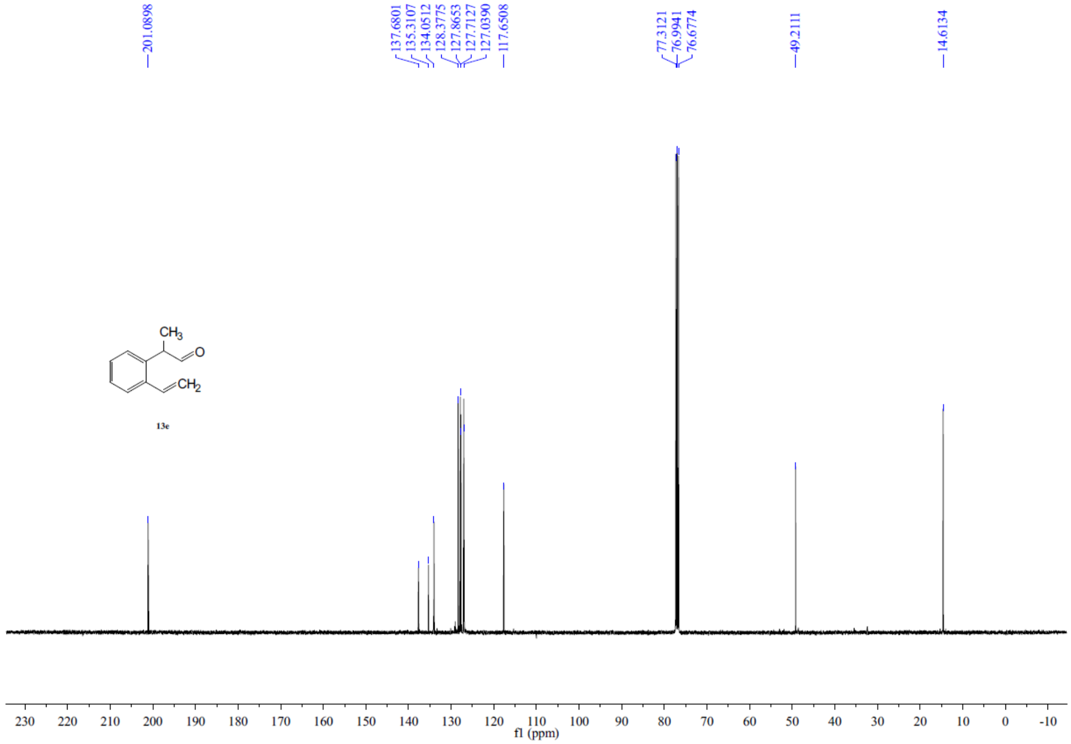

*^{1}H NMR ($CDCl_3$, 400 MHz) of 2-(2-(Prop-1-en-2-yl)phenyl)acetaldehyde (**13f**)*

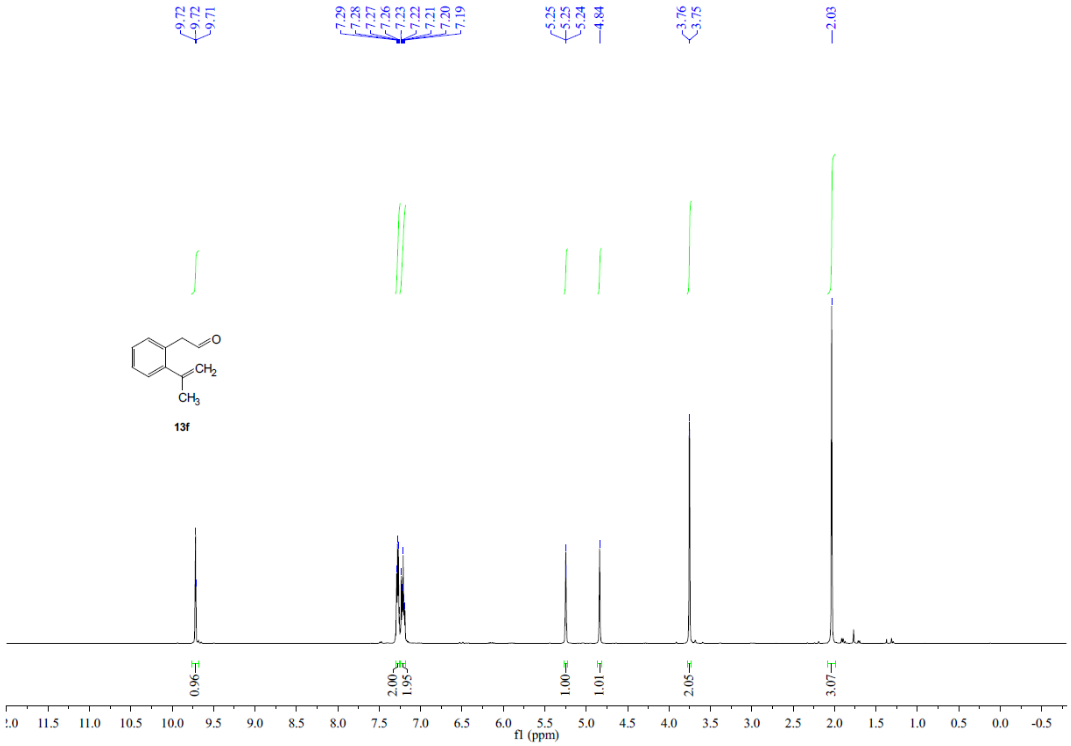

*^{13}C NMR ($CDCl_3$, 101 MHz) of 2-(2-(Prop-1-en-2-yl phenyl)acetaldehyde (**13f**)*

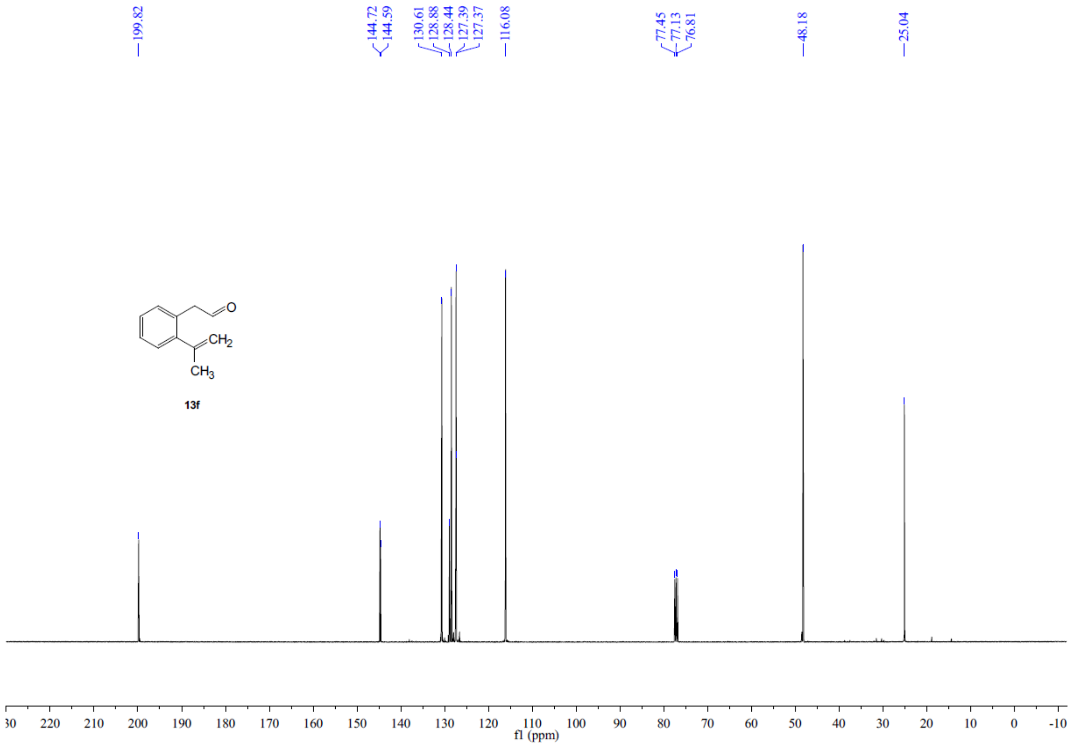

^{1}H NMR ($CDCl_3$, 400 MHz) of 2-(2-(1-Phenylvinyl)phenyl)acetaldehyde (**13g**)

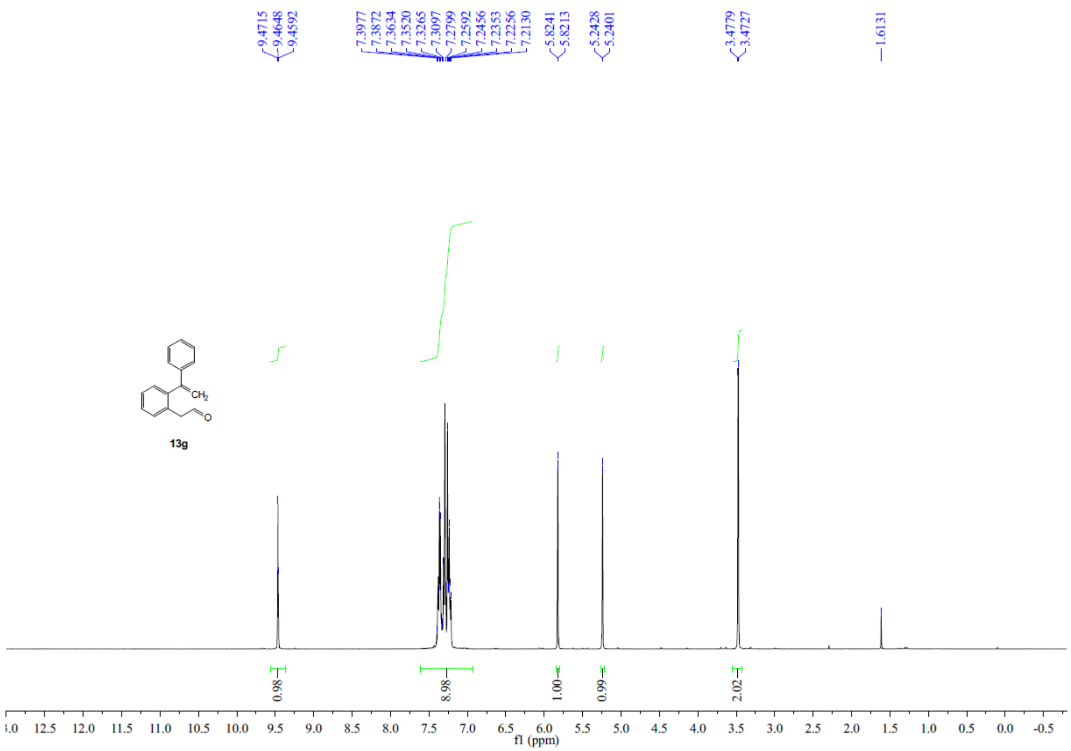

^{13}C NMR ($CDCl_3$, 101 MHz) of 2-(2-(1-Phenylvinyl)phenyl)acetaldehyde (**13g**)

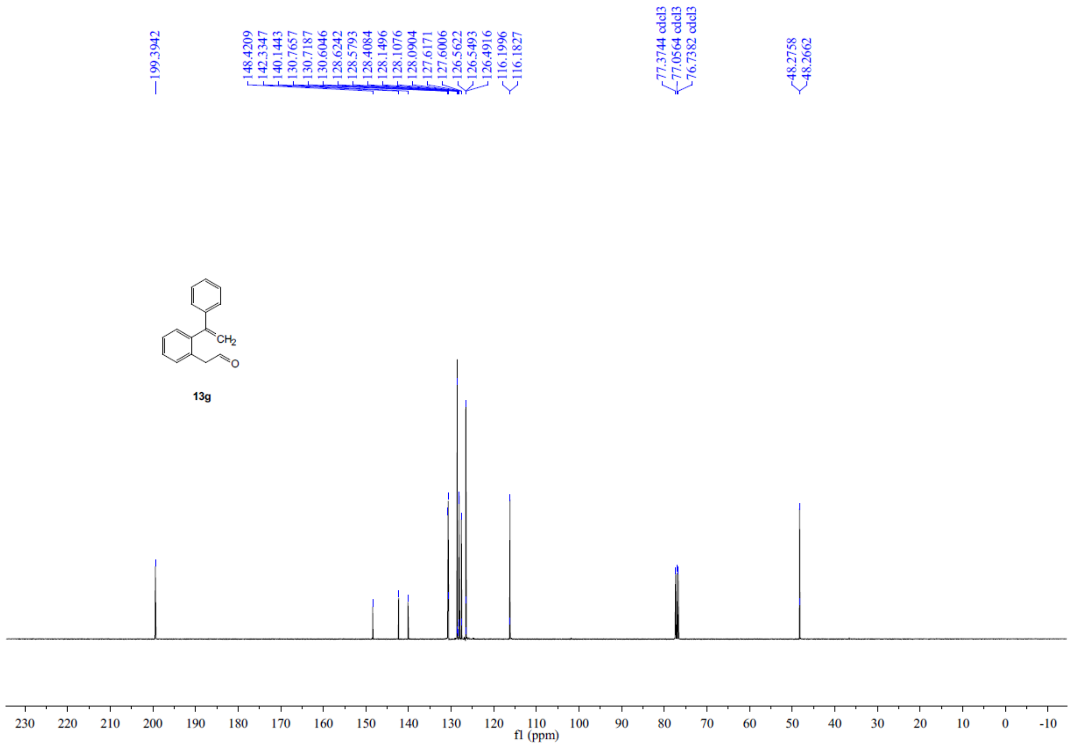

*^{1}H NMR (CDCl$_3$, 400 MHz) of 2-(1-Vinylnaphthalen-2-yl)acetaldehyde (**13h**)*

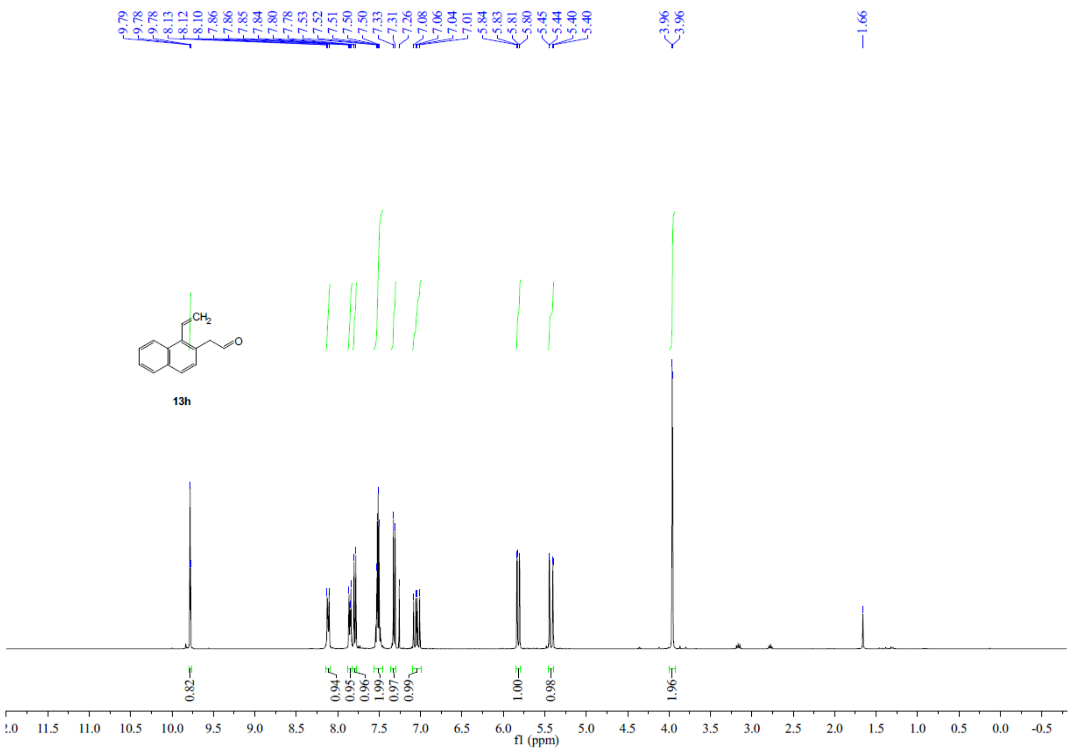

*^{13}C NMR (CDCl$_3$, 101 MHz) of 2-(1-Vinylnaphthalen-2-yl)acetaldehyde (**13h**)*

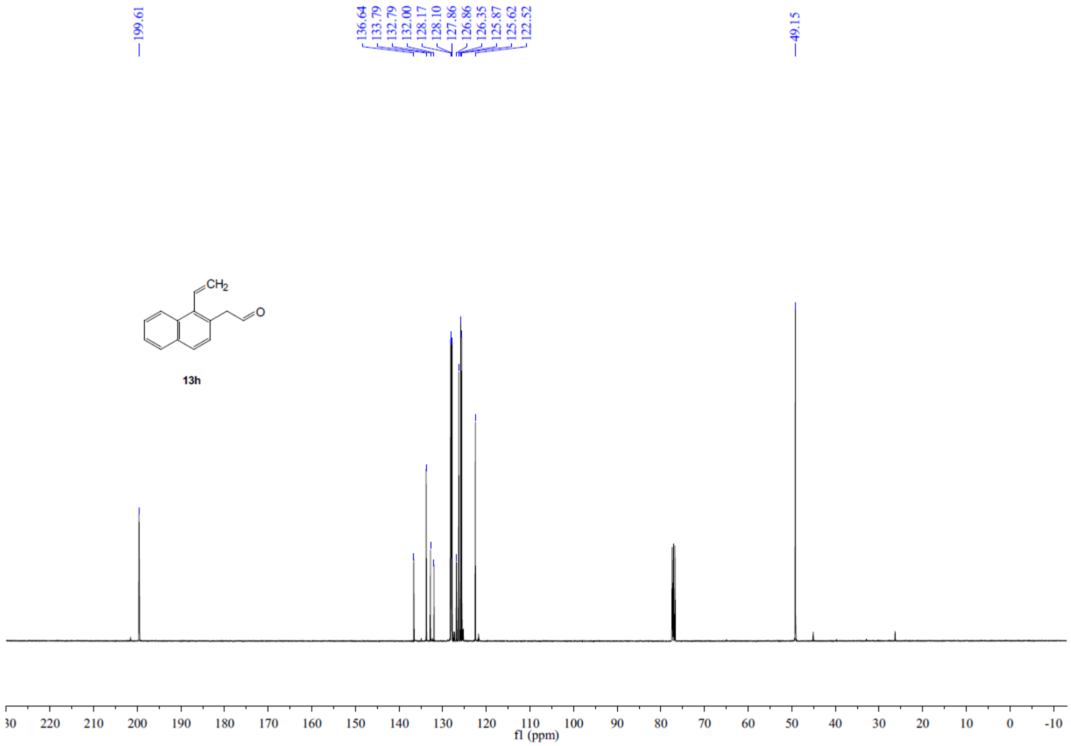

*^{1}H NMR ($CDCl_3$, 400 MHz) of 4-(3,4-Dimethoxyphenyl)-1,2,3,4-tetrahydronaphthalen-2-ol (**14aa**)*

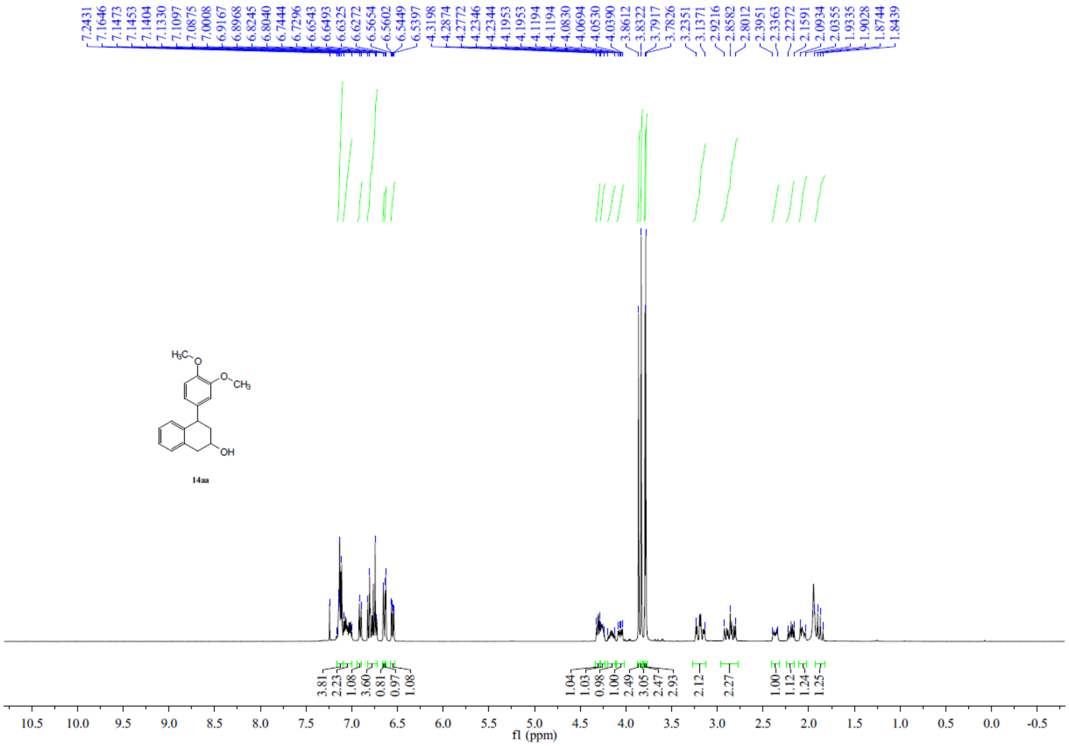

*^{13}C NMR ($CDCl_3$, 101 MHz) of 4-(3,4-Dimethoxyphenyl)-1,2,3,4-tetrahydronaphthalen-2-ol (**14aa**)*

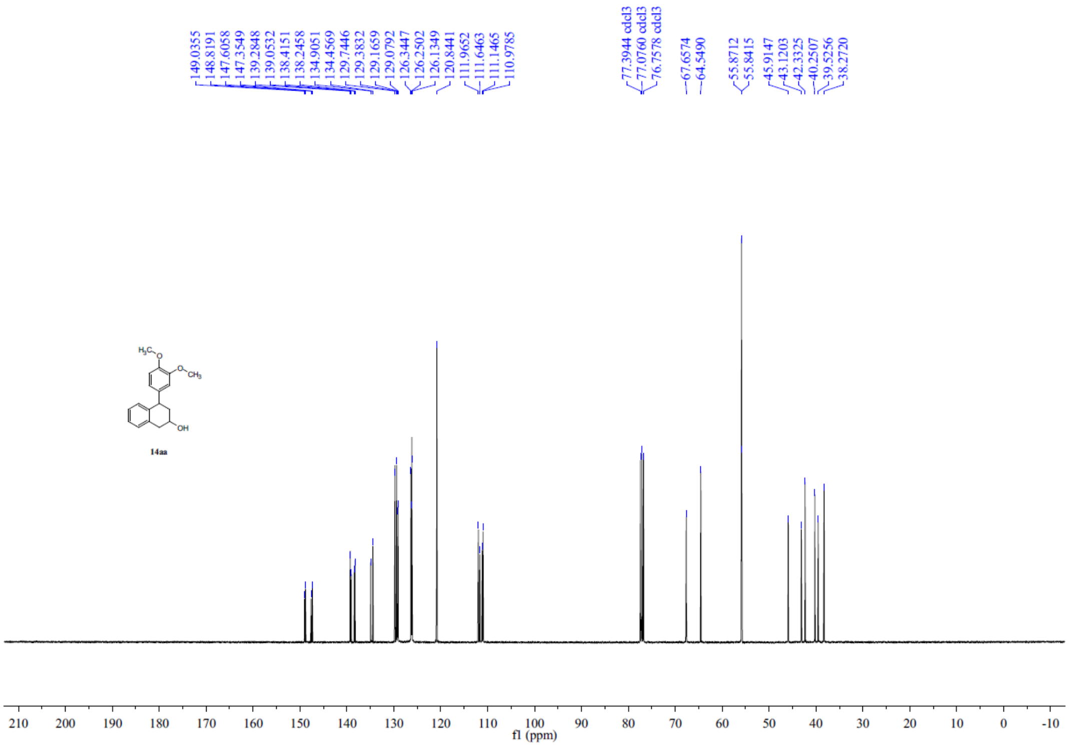

*HSQC of 4-(3,4-Dimethoxyphenyl)-1,2,3,4-tetrahydronaphthalen-2-ol (**14aa**)*

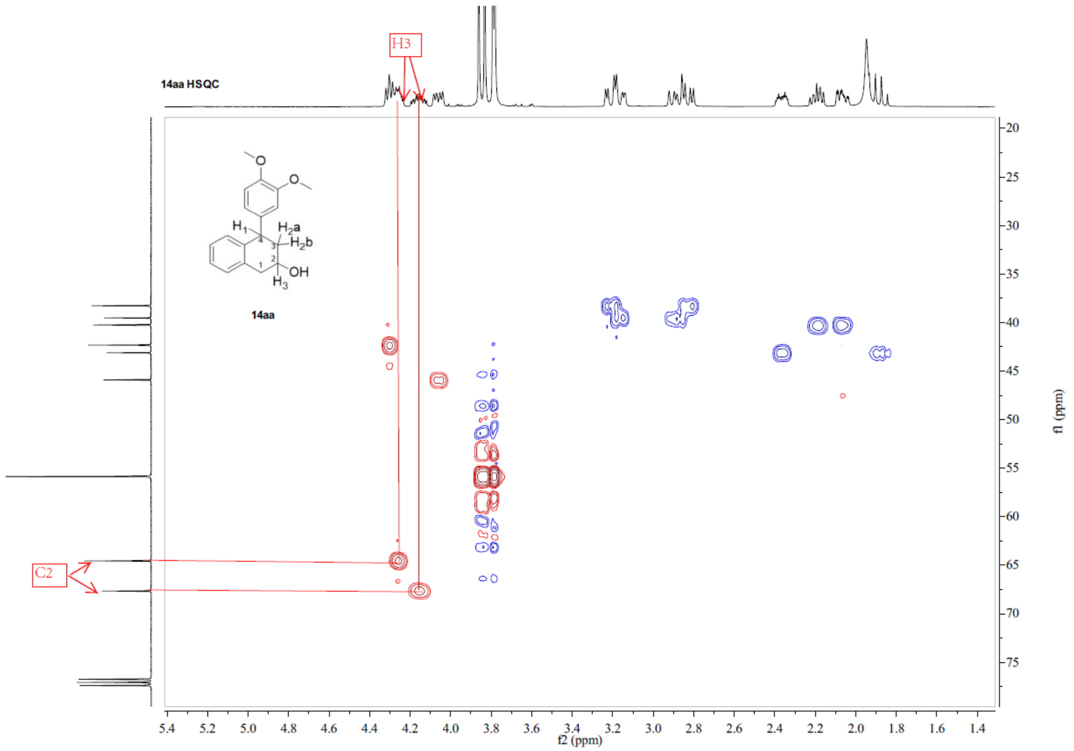

*NOE of 4-(3,4-Dimethoxyphenyl)-1,2,3,4-tetrahydronaphthalen-2-ol (**14aa**)*

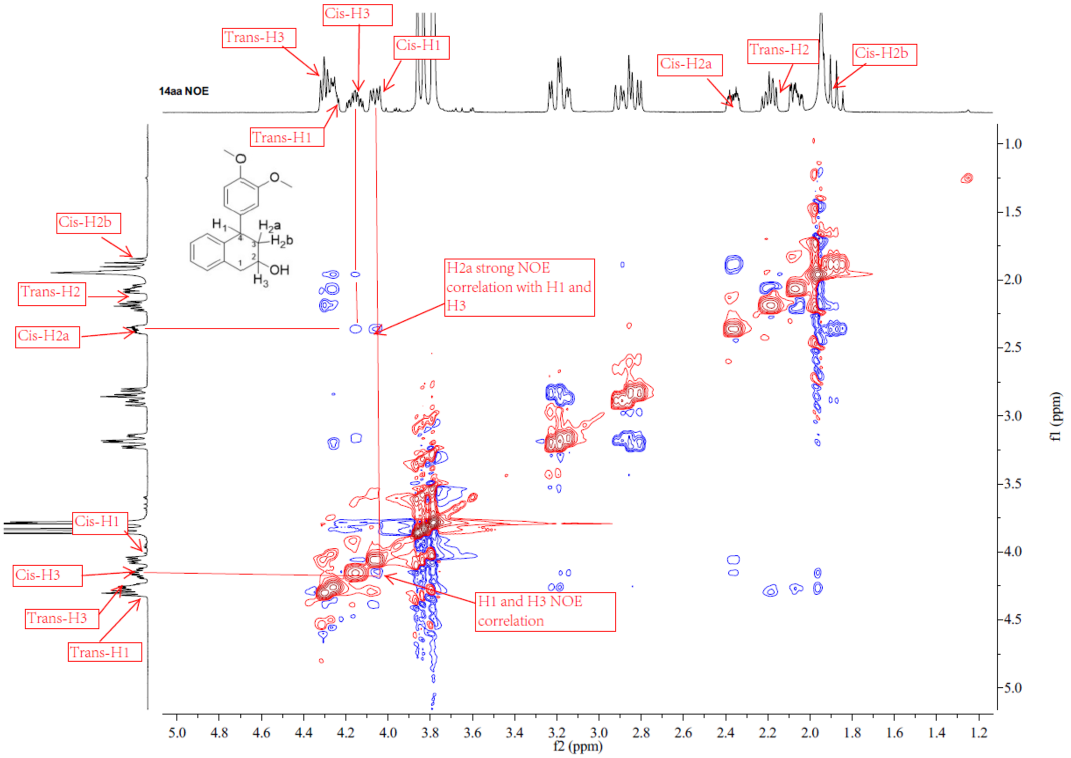

HMBC of 4-(3,4-Dimethoxyphenyl)-1,2,3,4-tetrahydronaphthalen-2-ol ***(14aa)***

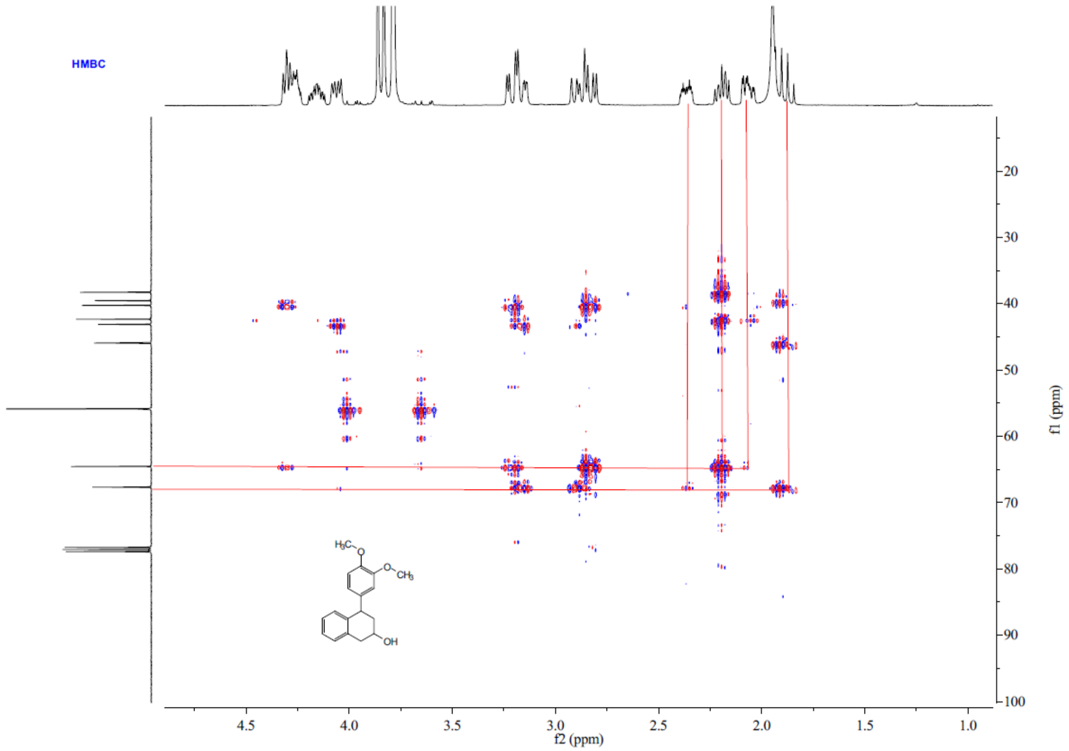

COSY of 4-(3,4-Dimethoxyphenyl)-1,2,3,4-tetrahydronaphthalen-2-ol ***(14aa)***

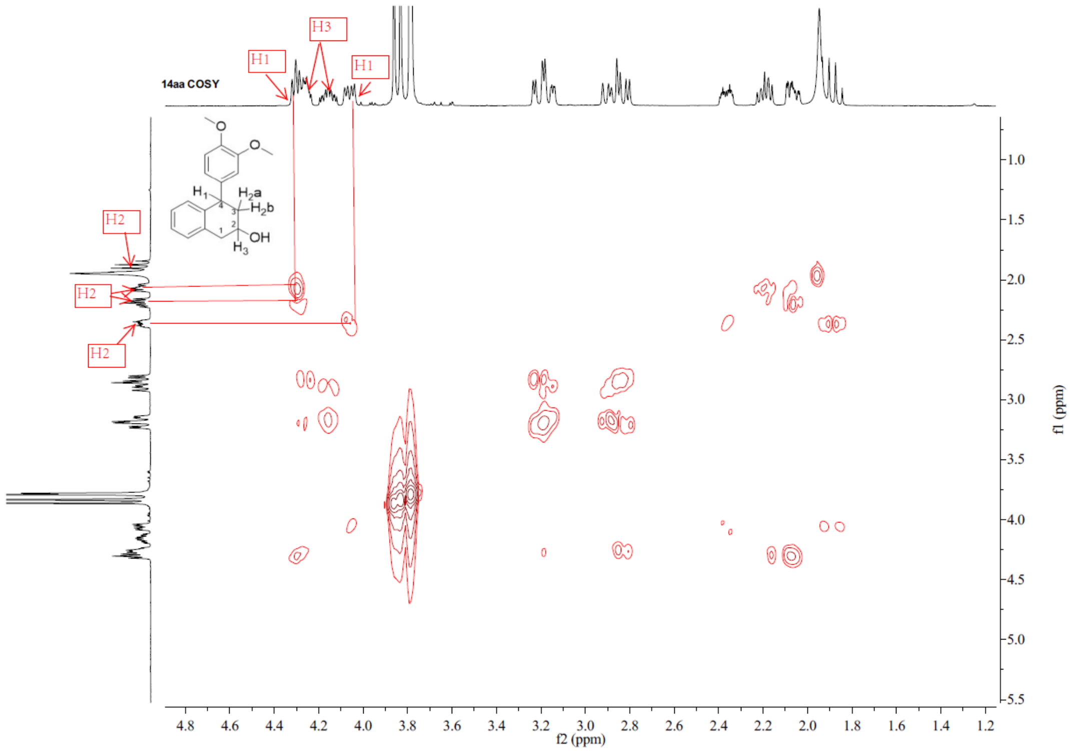

^1H NMR ($CDCl_3$, 400 MHz) of 4-(2,4-Dimethoxyphenyl)-1,2,3,4-tetrahydronaphthalen-2-ol ***(14ab)***

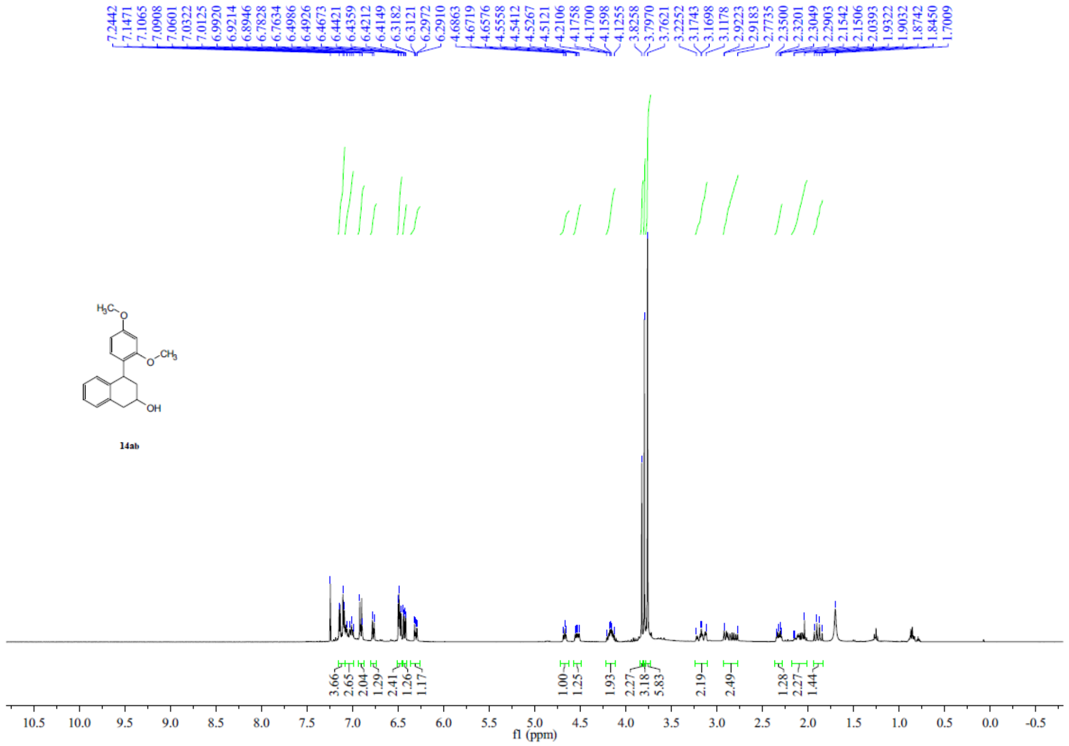

^{13}C NMR ($CDCl_3$, 101 MHz) of 4-(2,4-Dimethoxyphenyl)-1,2,3,4-tetrahydronaphthalen-2-ol ***(14ab)***

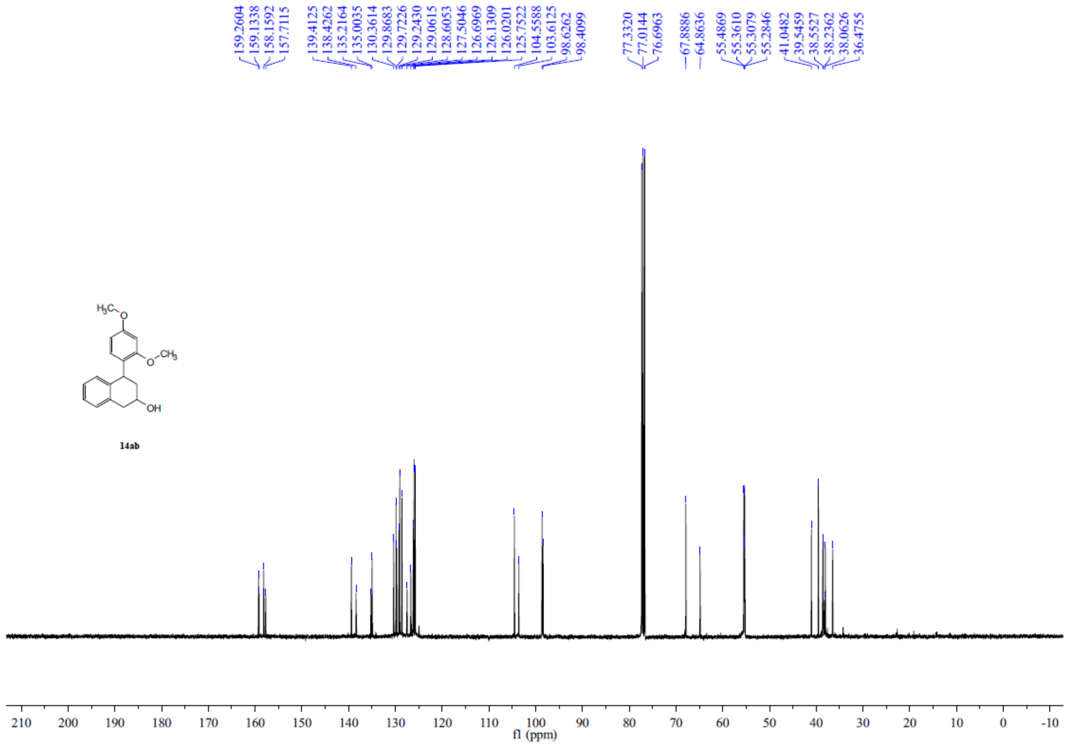

*HSQC of 4-(2,4-Dimethoxyphenyl)-1,2,3,4-tetrahydronaphthalen-2-ol **(14ab))***

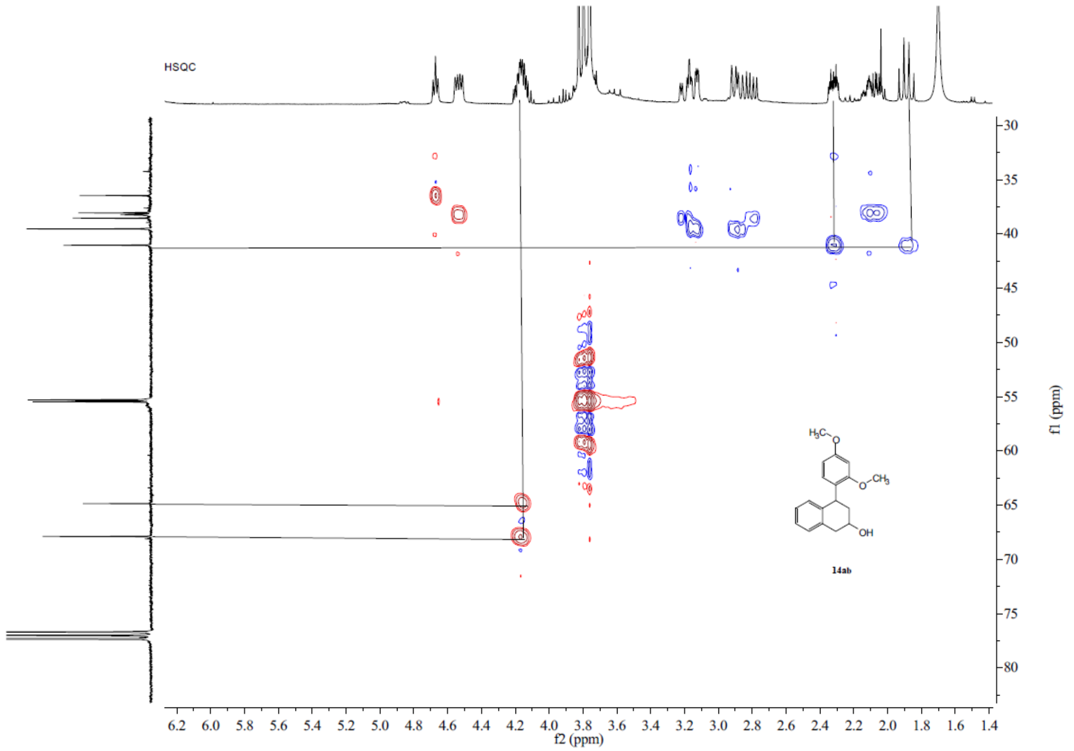

*NOE of 4-(2,4-Dimethoxyphenyl)-1,2,3,4-tetrahydronaphthalen-2-ol **(14ab)***

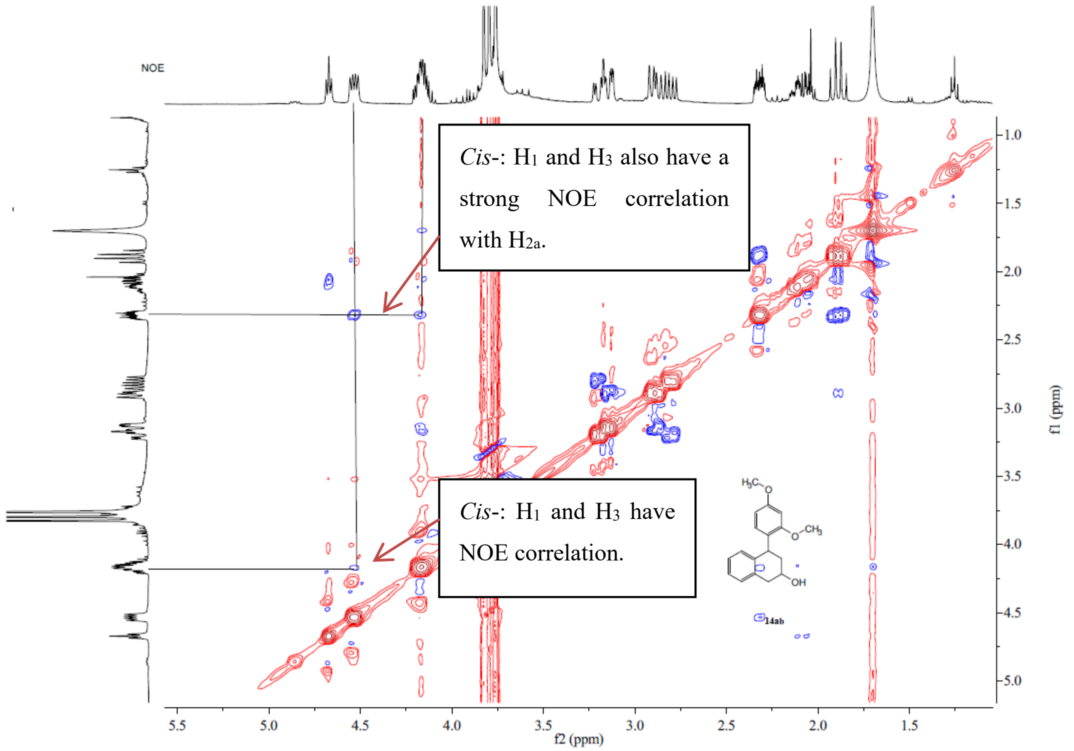

^{1}H NMR ($CDCl_3$, 400 MHz) of 4-(4-Methoxy-3,5-dimethylphenyl)-1,2,3,4-tetrahydronaphthalen-2-ol ***(14ac)***

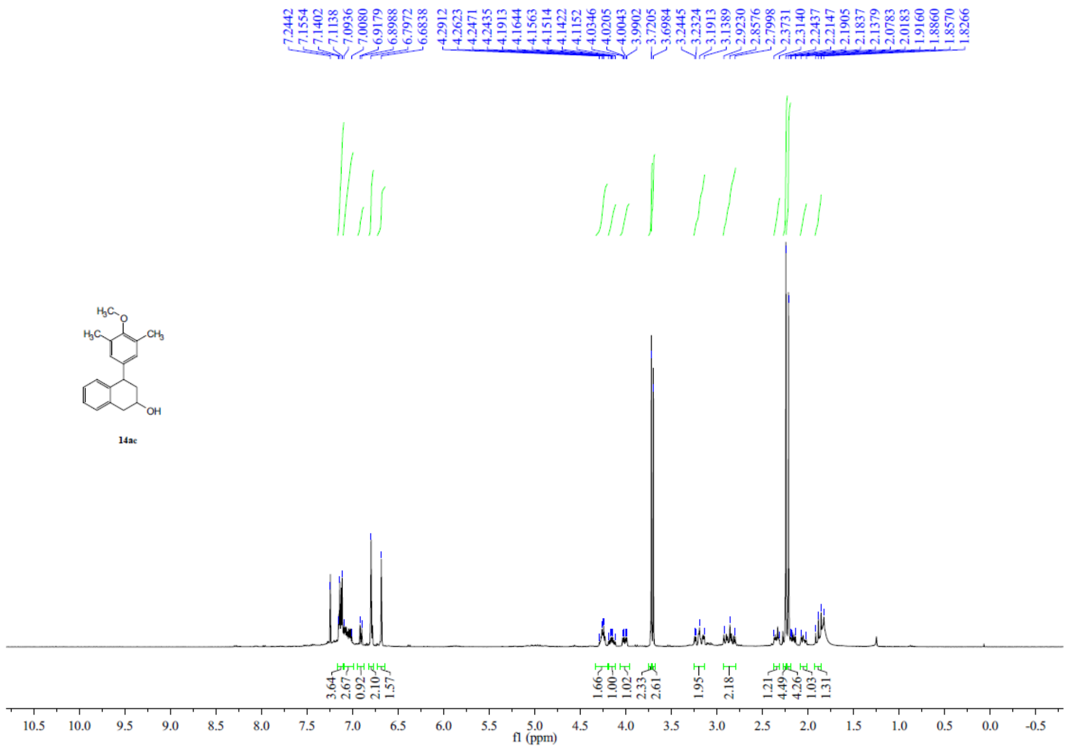

^{13}C NMR ($CDCl_3$, 101 MHz) of 4-(4-Methoxy-3,5-dimethylphenyl)-1,2,3,4-tetrahydronaphthalen-2-ol ***(14ac)***

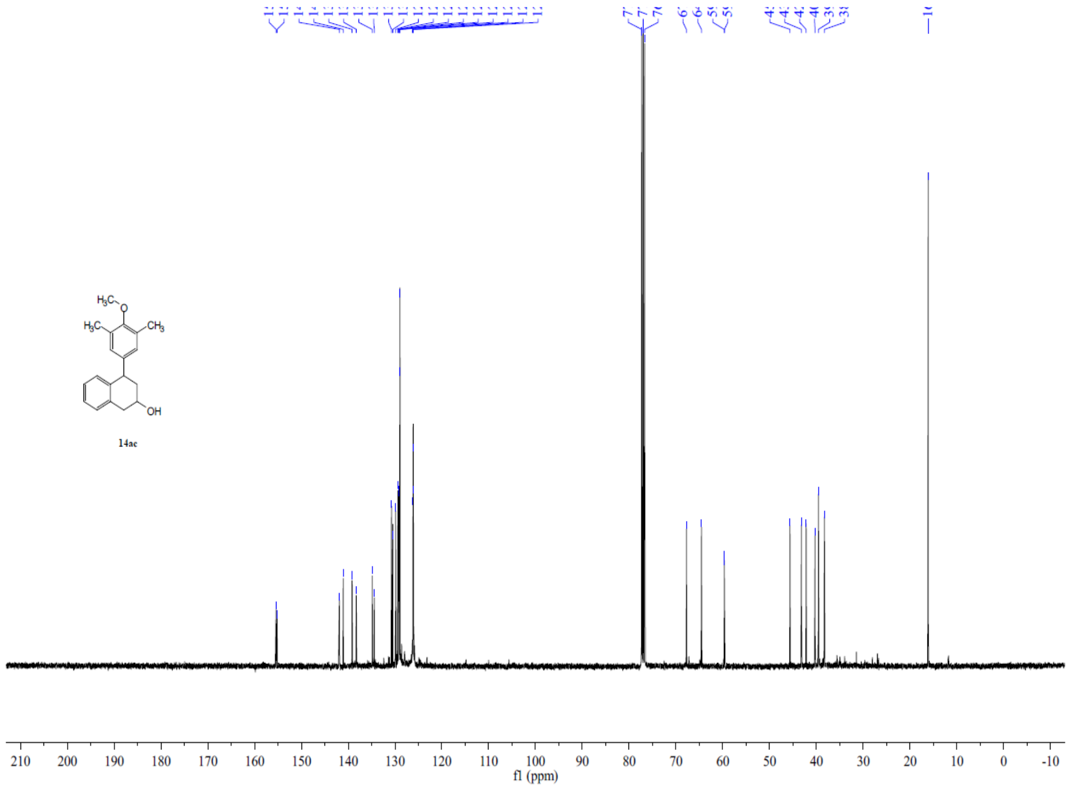

*HSQC of 4-(4-Methoxy-3,5-dimethylphenyl)-1,2,3,4-tetrahydronaphthalen-2-ol **(14ac)***

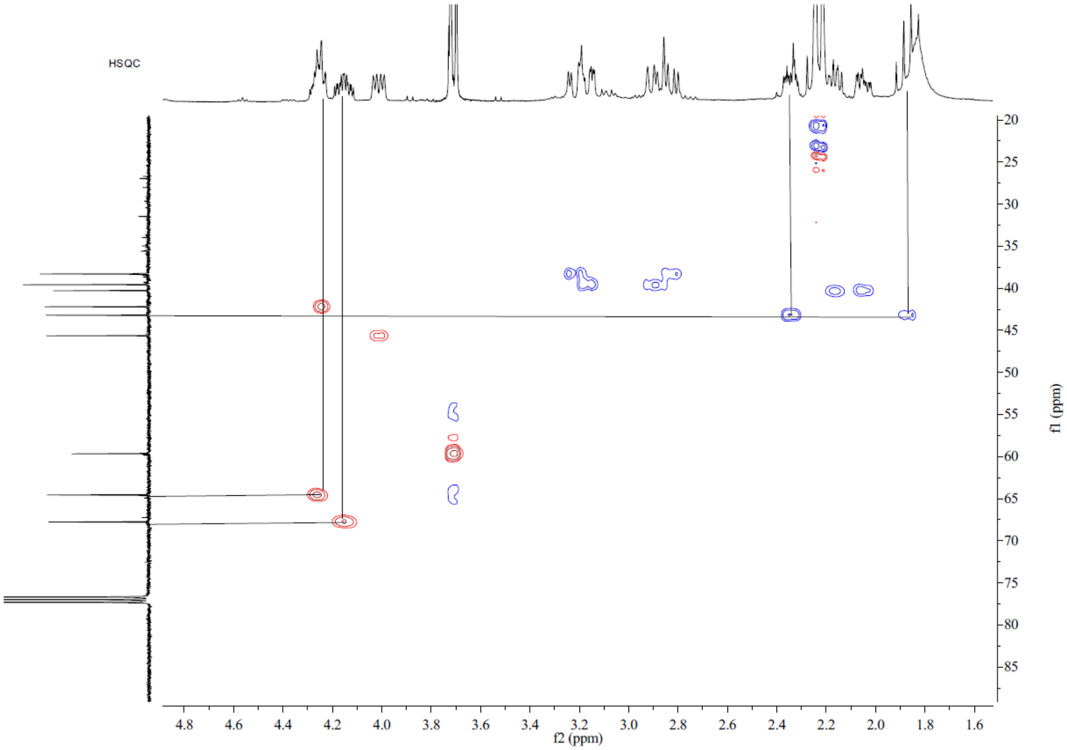

*NOE of 4-(4-Methoxy-3,5-dimethylphenyl)-1,2,3,4-tetrahydronaphthalen-2-ol **(14ac)***

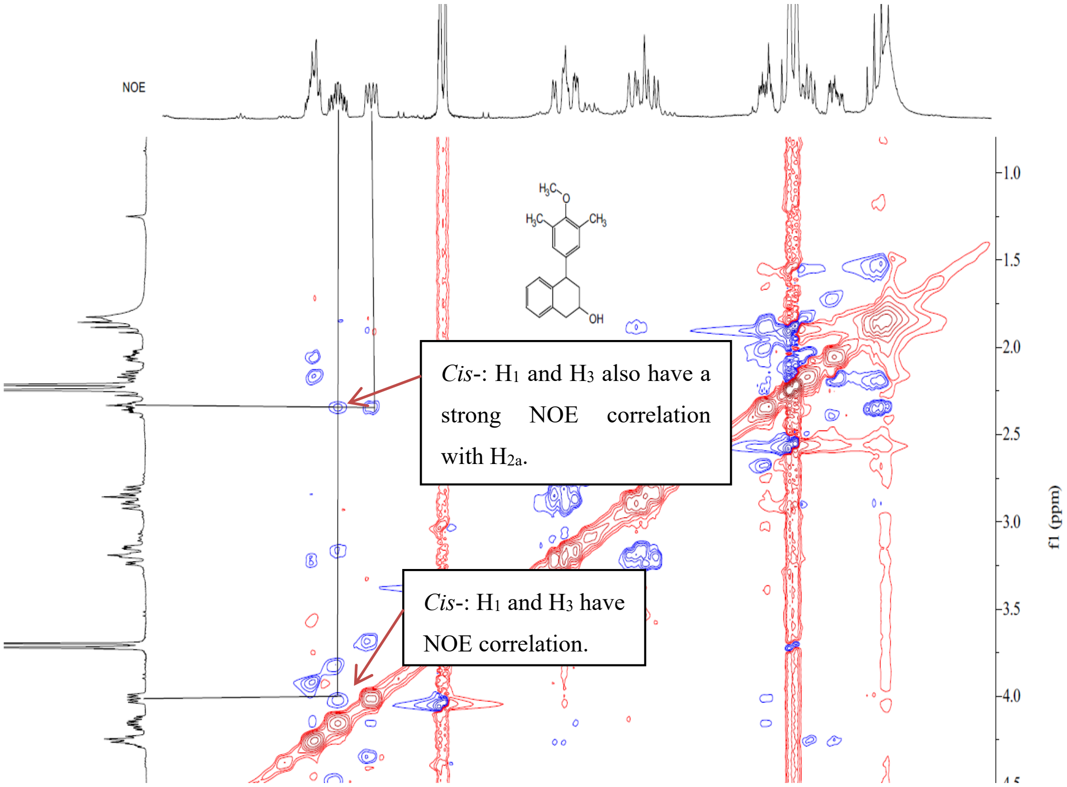

^{1}H NMR ($CDCl_3$, 400 MHz) of 4-(2,5-Dimethoxyphenyl)-1,2,3,4-tetrahydronaphthalen-2-ol ***(14ad)***

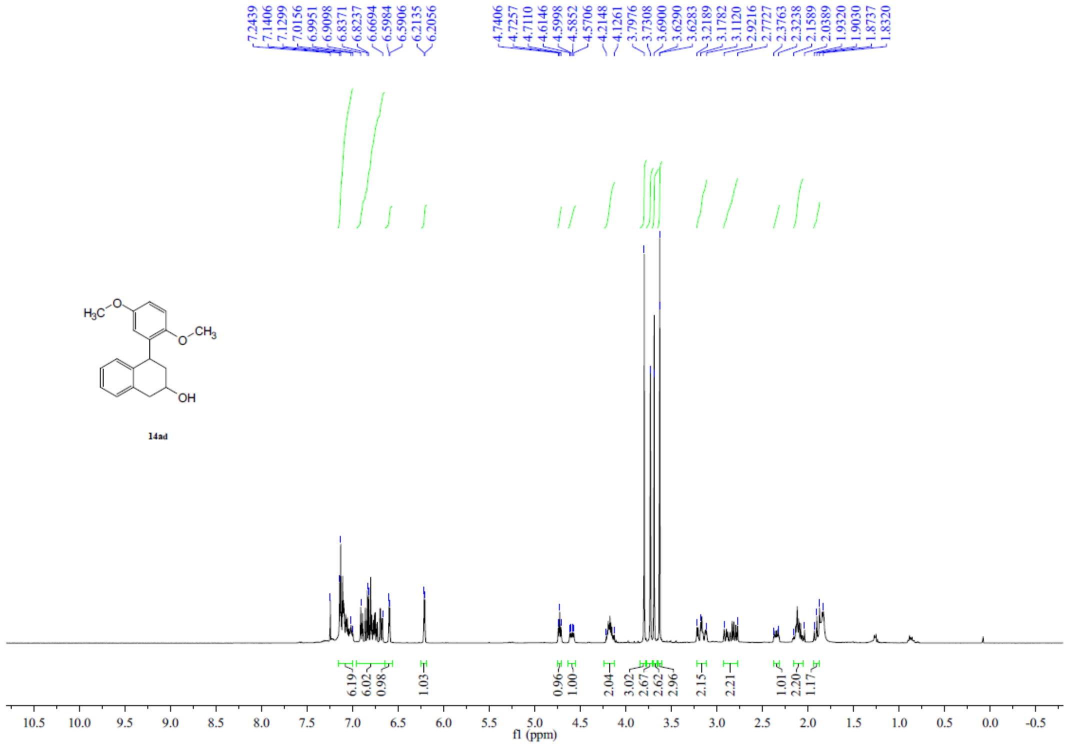

^{13}C NMR ($CDCl_3$, 101 MHz) of 4-(2,5-Dimethoxyphenyl)-1,2,3,4-tetrahydronaphthalen-2-ol ***(14ad)***

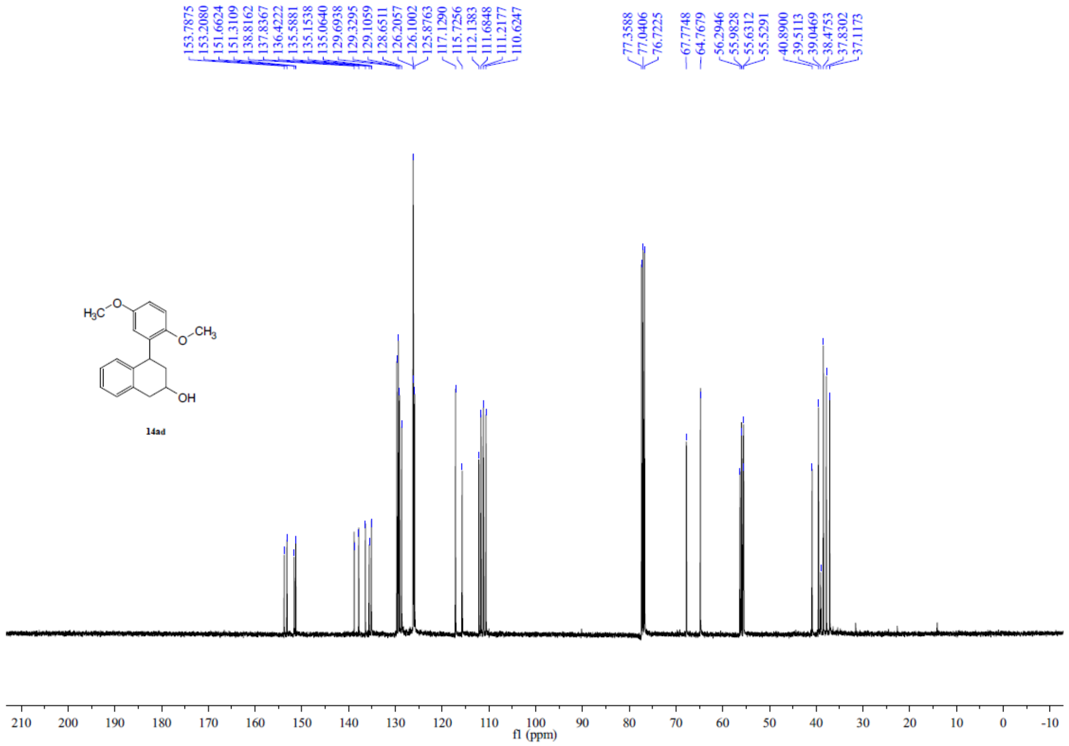

HSQC of 4-(2,5-Dimethoxyphenyl)-1,2,3,4-tetrahydronaphthalen-2-ol ***(14ad)***

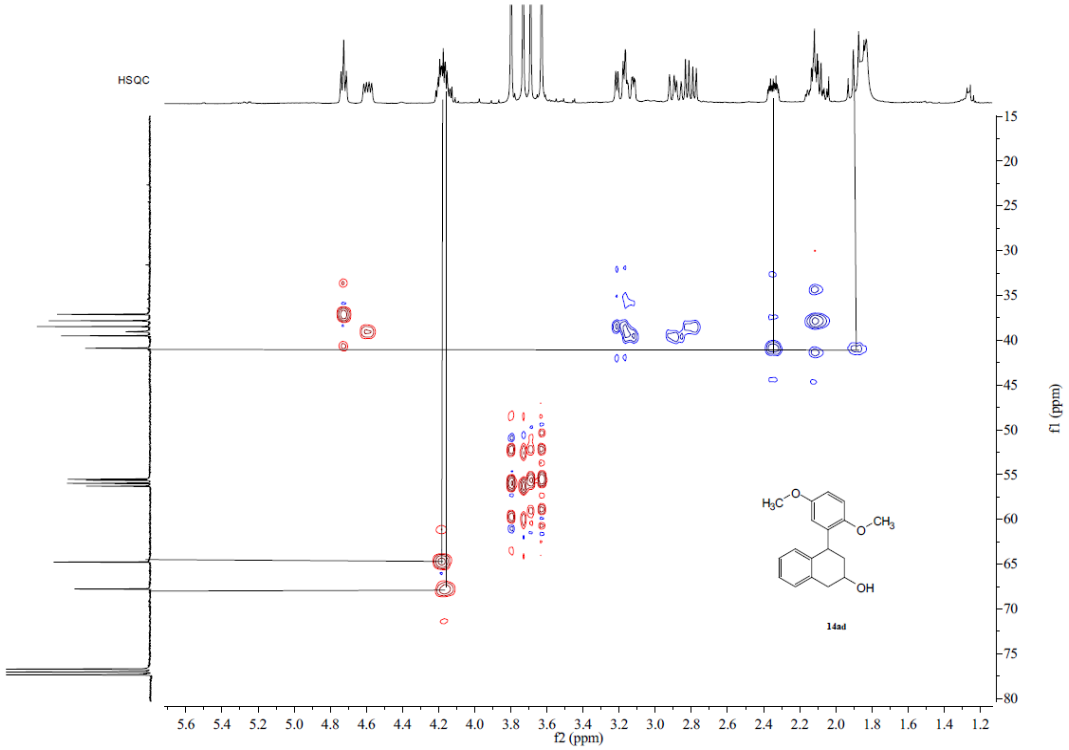

NOE of 4-(2,5-Dimethoxyphenyl)-1,2,3,4-tetrahydronaphthalen-2-ol ***(14ad)***

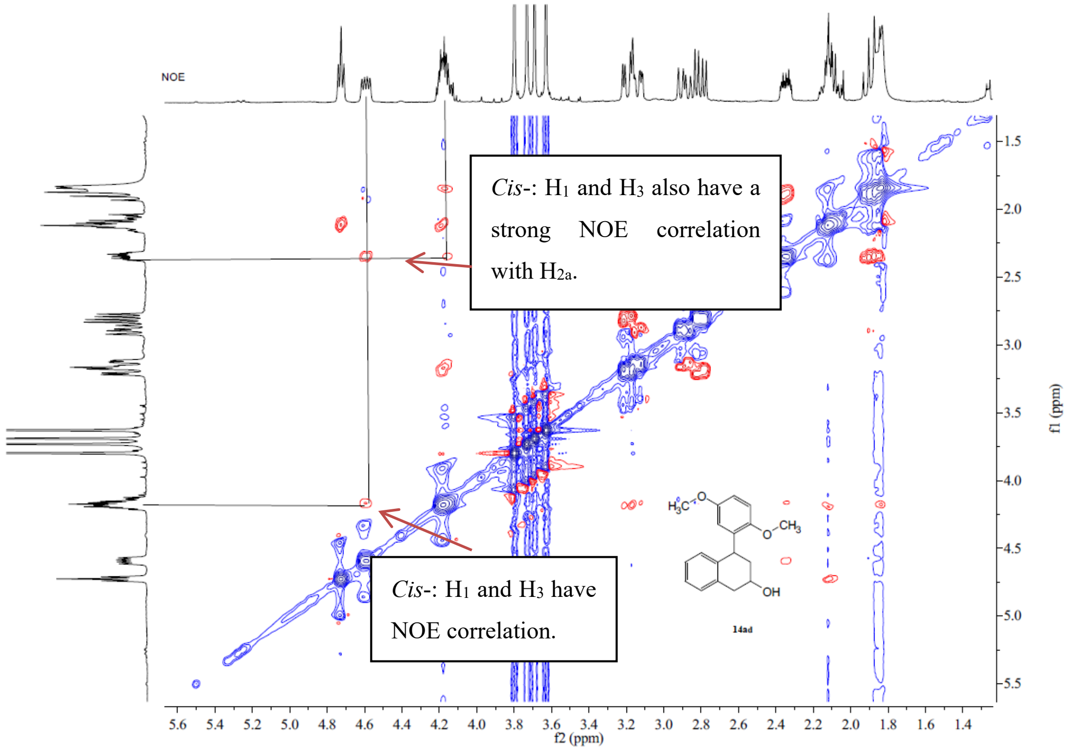

^1H NMR ($CDCl_3$, 400 MHz) of 4-(2-Methoxy-5-methylphenyl)-1,2,3,4-tetrahydronaphthalen-2-ol **(14ae)**

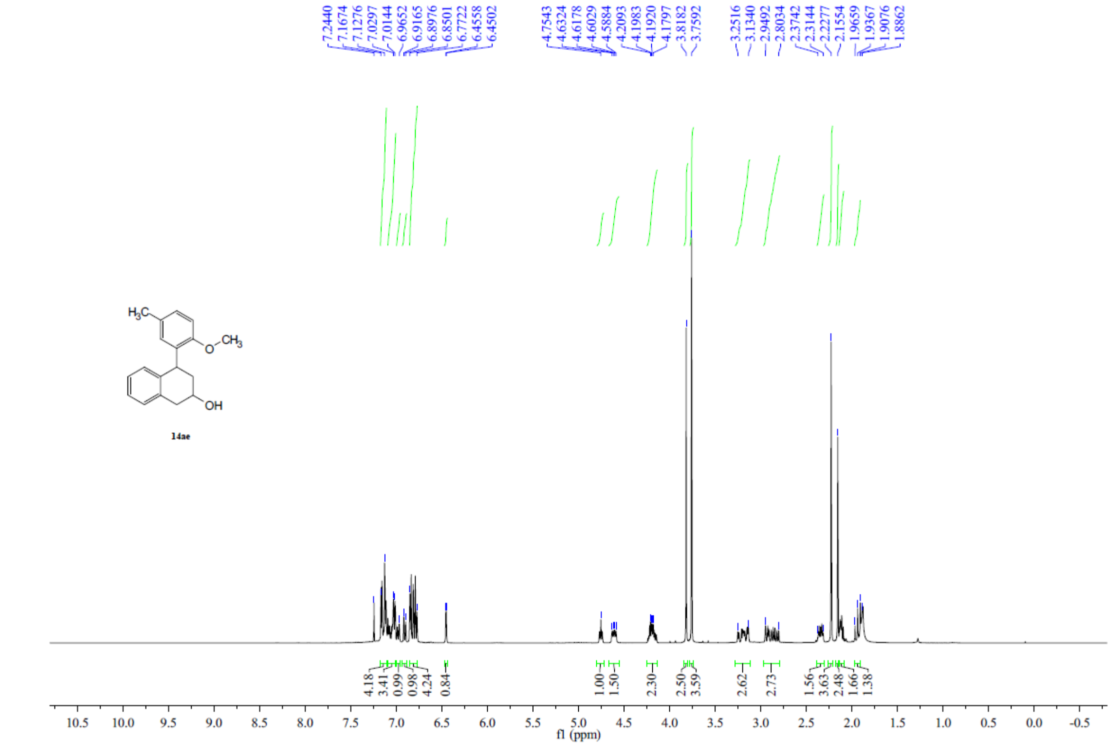

^{13}C NMR ($CDCl_3$, 101 MHz) of 4-(2-Methoxy-5-methylphenyl)-1,2,3,4-tetrahydronaphthalen-2-ol **(14ae)**

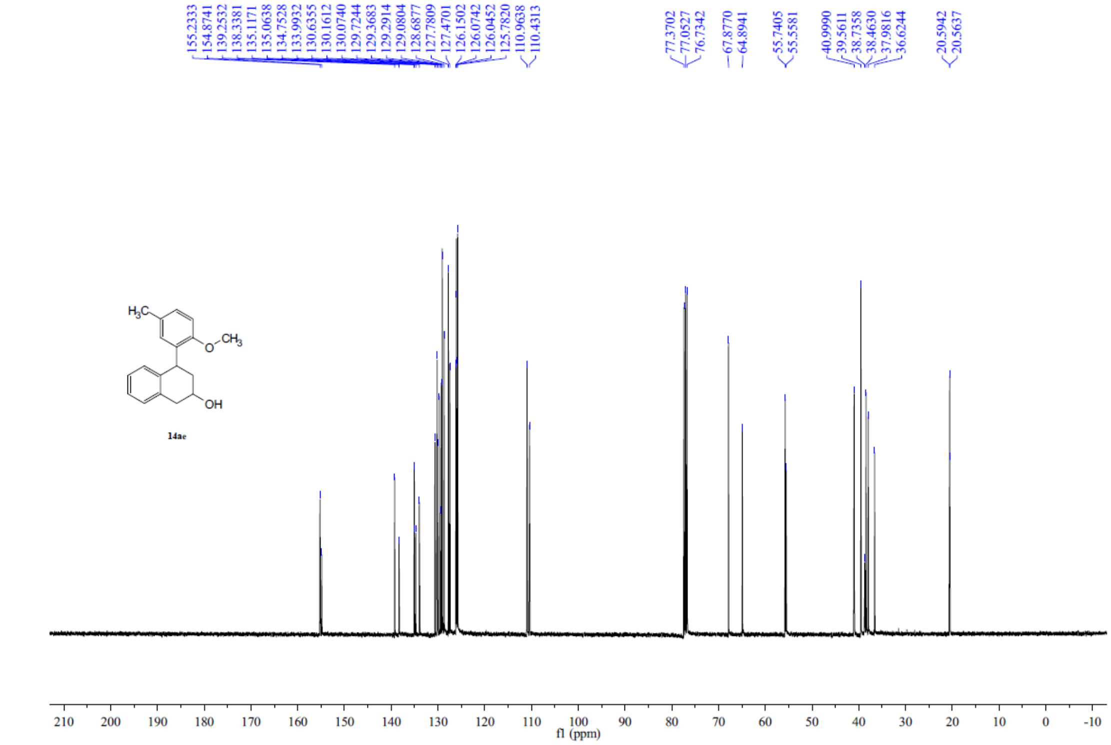

HSQC of 4-(2-Methoxy-5-methylphenyl)-1,2,3,4-tetrahydronaphthalen-2-ol ***(14ae)***

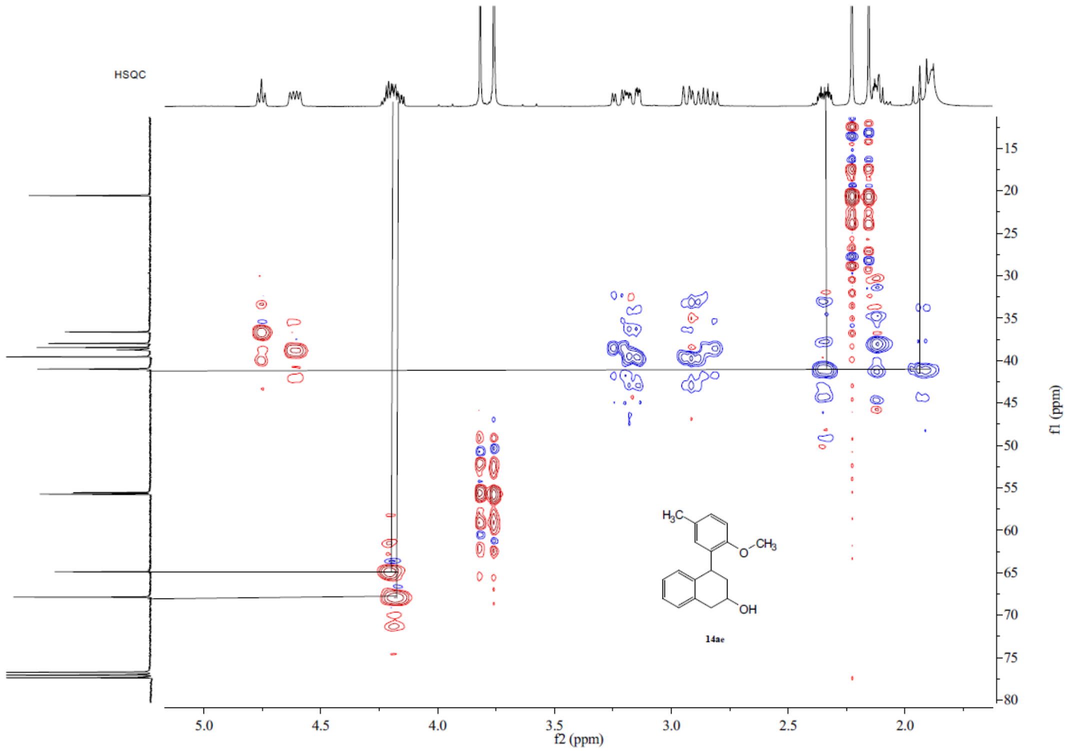

NOE of 4-(2-Methoxy-5-methylphenyl)-1,2,3,4-tetrahydronaphthalen-2-ol ***(14ae)***

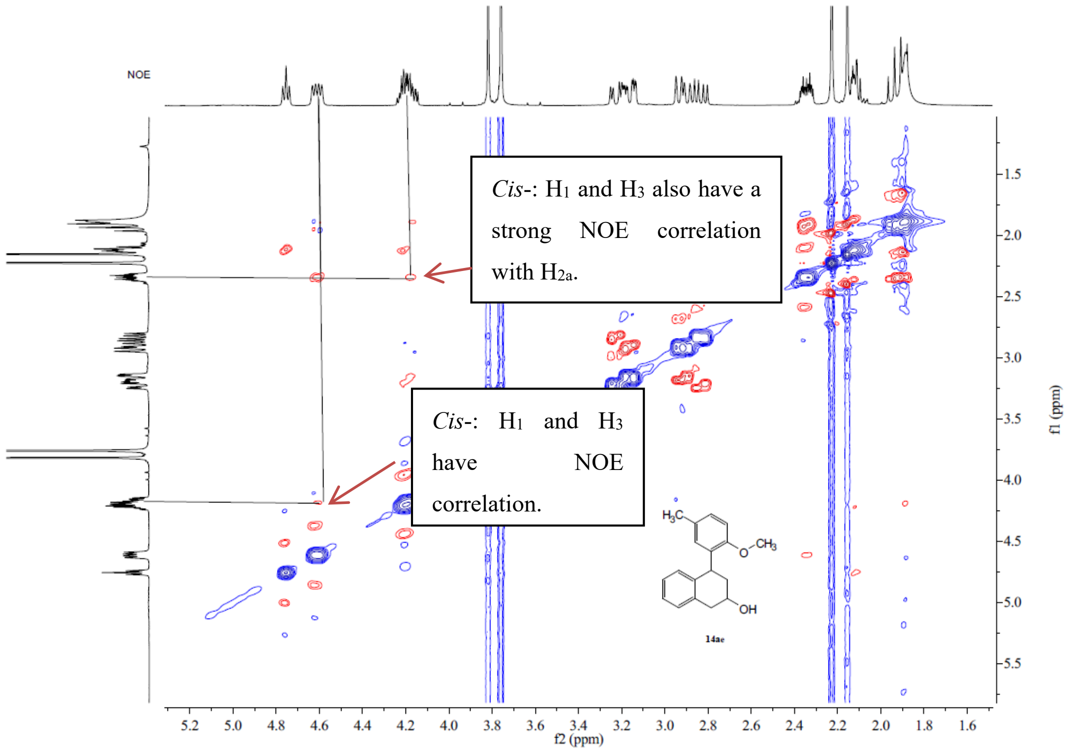

^{1}H NMR ($CDCl_3$, 400 MHz) of 4-(Furan-2-yl)-1,2,3,4-tetrahydronaphthalen-2-ol ***(14af)***

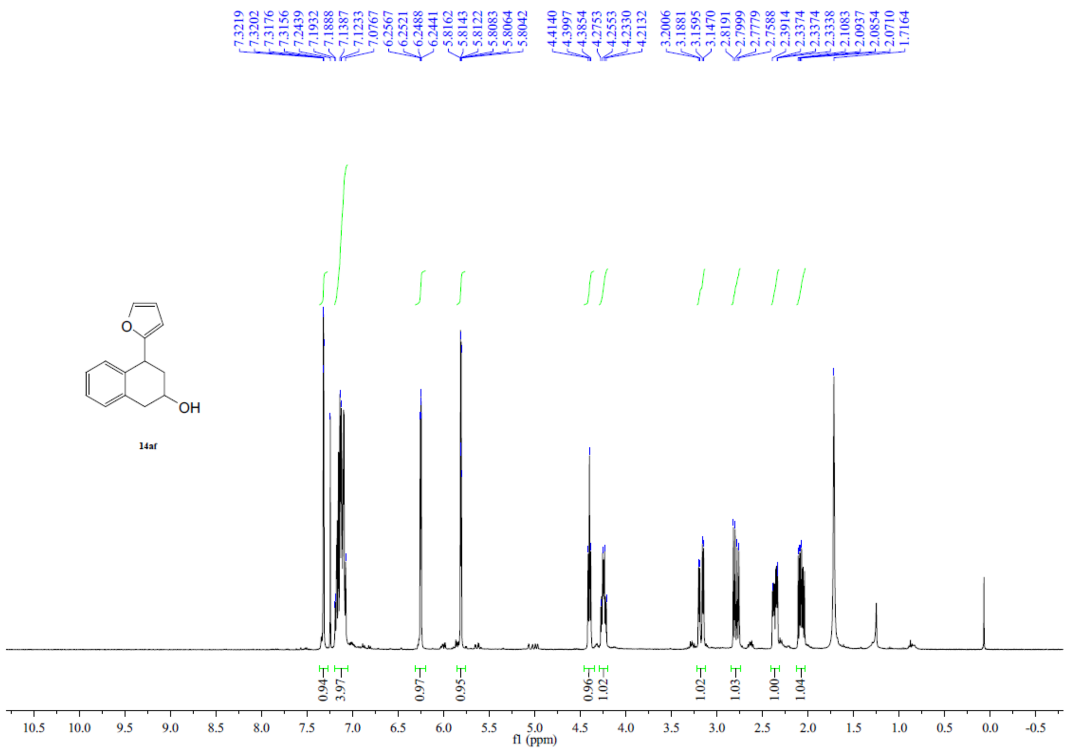

^{13}C NMR ($CDCl_3$, 101 MHz) of 4-(Furan-2-yl)-1,2,3,4-tetrahydronaphthalen-2-ol ***(14af)***

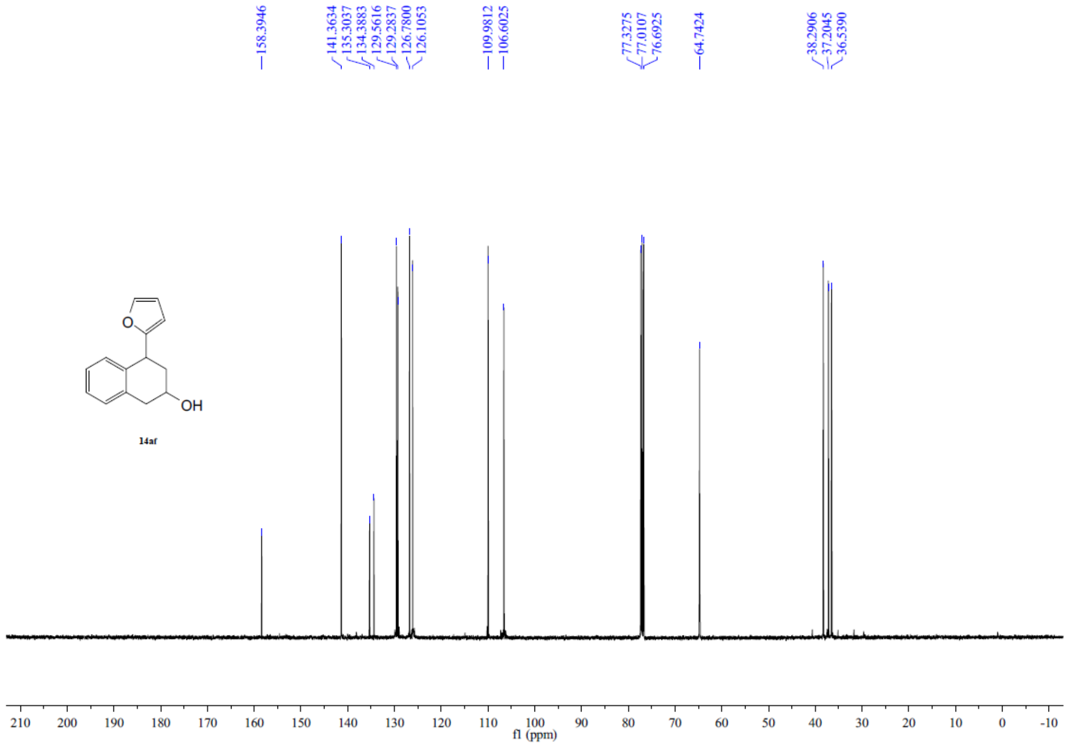

*HSQC of 4-(Furan-2-yl)-1,2,3,4-tetrahydronaphthalen-2-ol **(14af)***

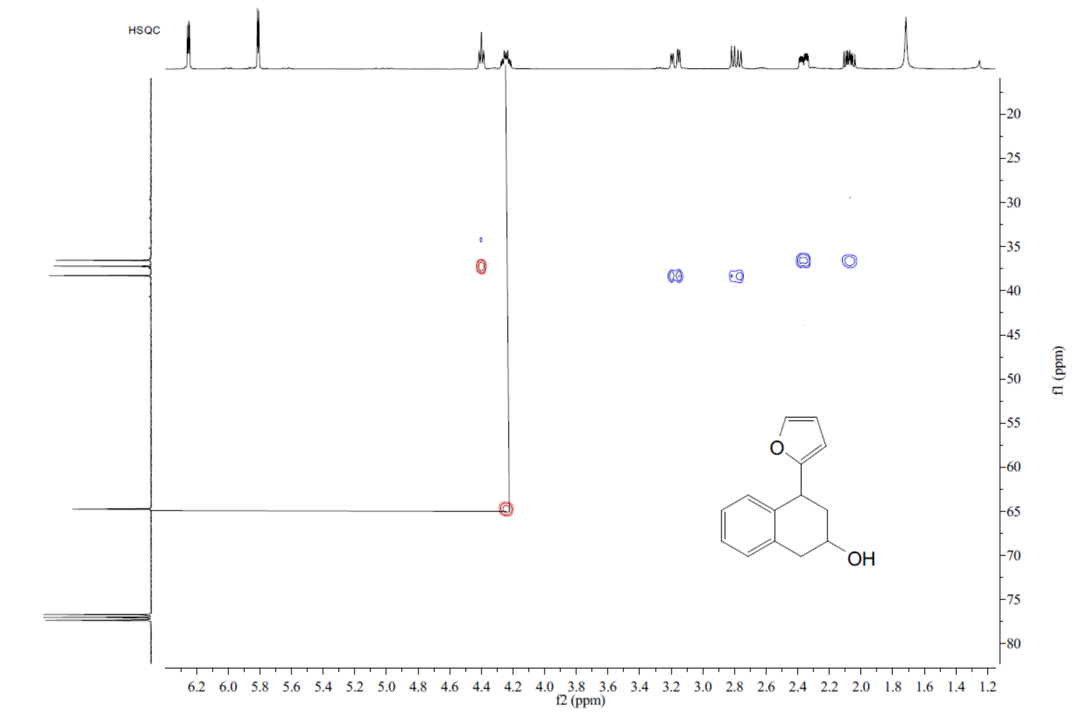

*NOE of 4-(Furan-2-yl)-1,2,3,4-tetrahydronaphthalen-2-ol **(14af)***

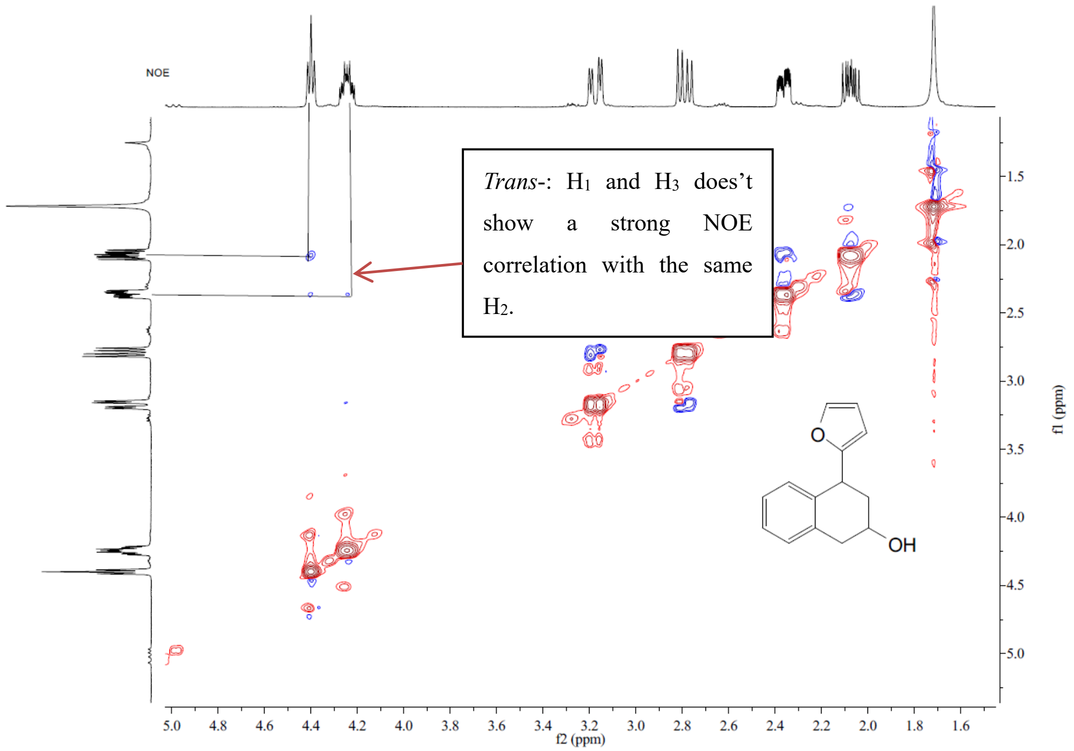

^{1}H NMR ($CDCl_3$, 400 MHz) of 4-(5-Methylfuran-2-yl)-1,2,3,4-tetrahydronaphthalen-2-ol **(14ag)**

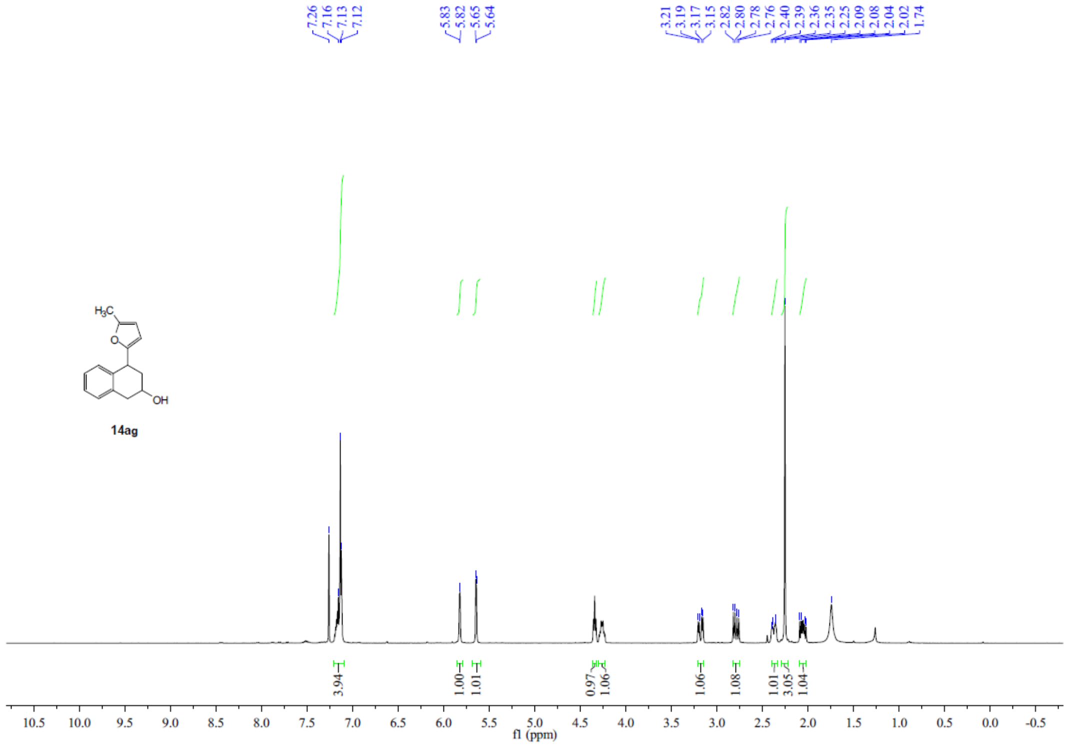

^{13}C NMR ($CDCl_3$, 101 MHz) of 4-(5-Methylfuran-2-yl)-1,2,3,4-tetrahydronaphthalen-2-ol **(14ag)**

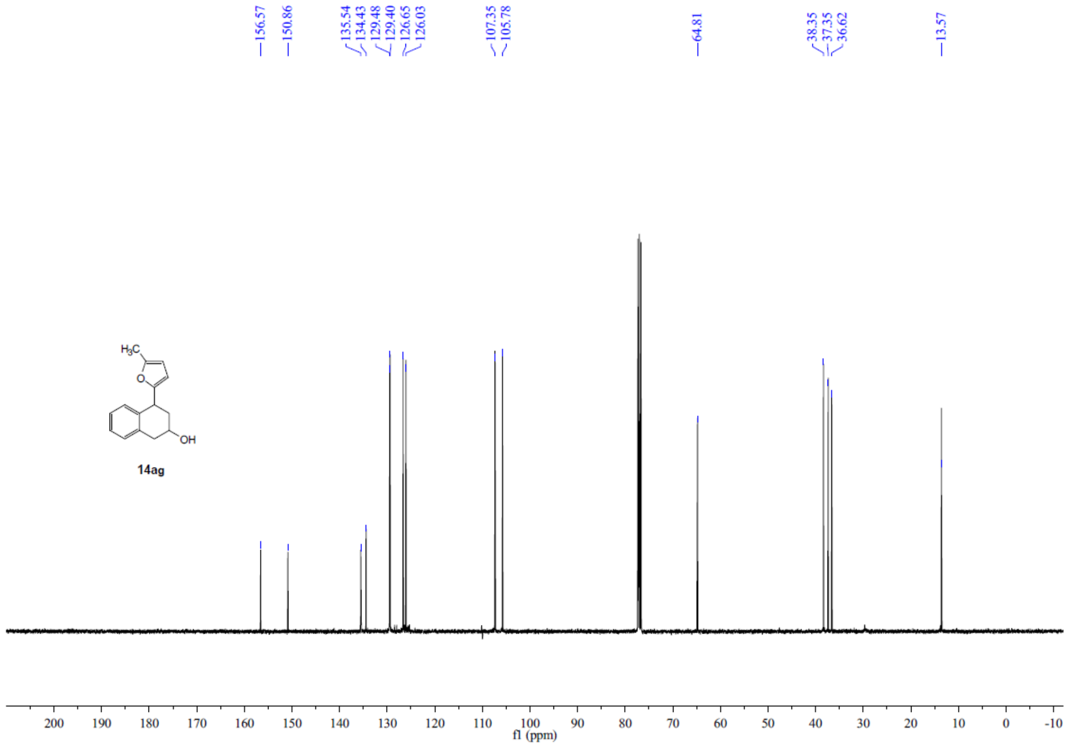

*HSQC of 4-(5-Methylfuran-2-yl)-1,2,3,4-tetrahydronaphthalen-2-ol **(14ag)***

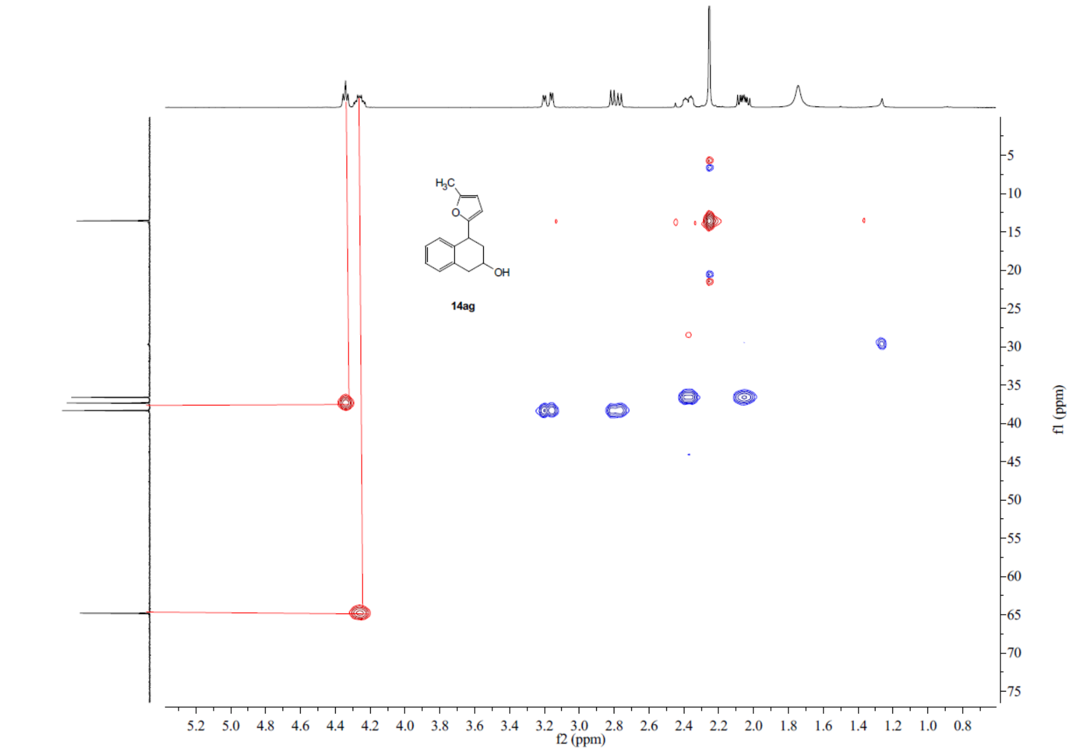

*NOE of 4-(5-Methylfuran-2-yl)-1,2,3,4-tetrahydronaphthalen-2-ol **(14ag)***

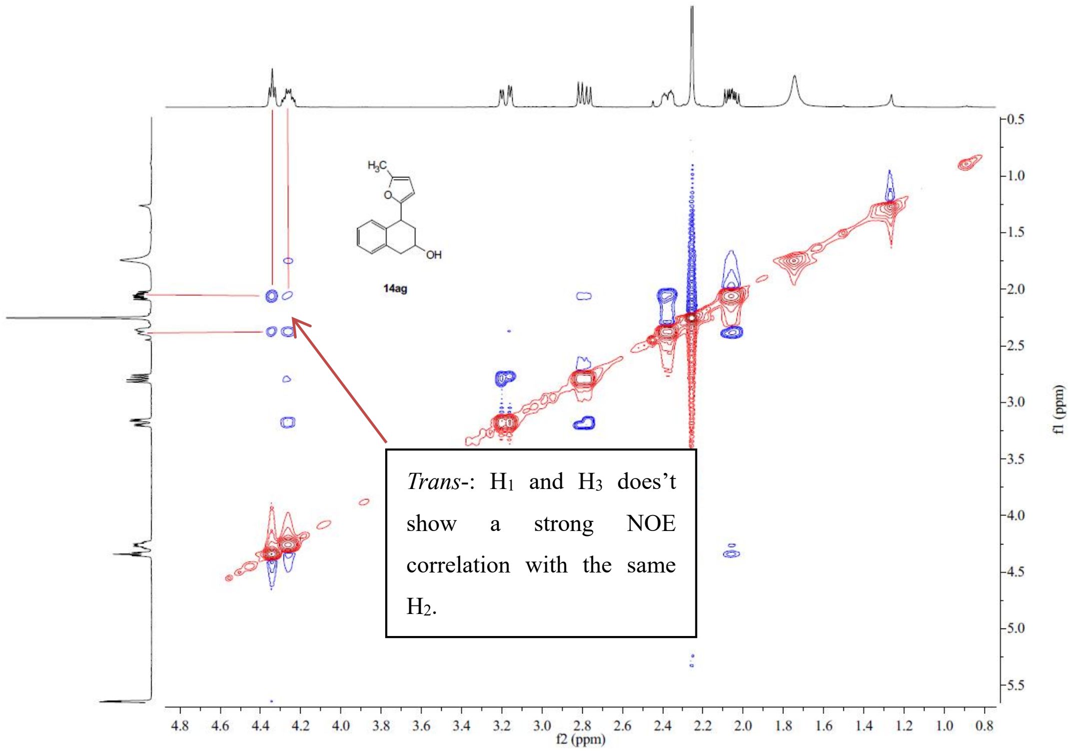

^{1}H NMR ($CDCl_3$, 400 MHz) of 4-(Thiophen-2-yl)-1,2,3,4-tetrahydronaphthalen-2-ol ***(14ah)***

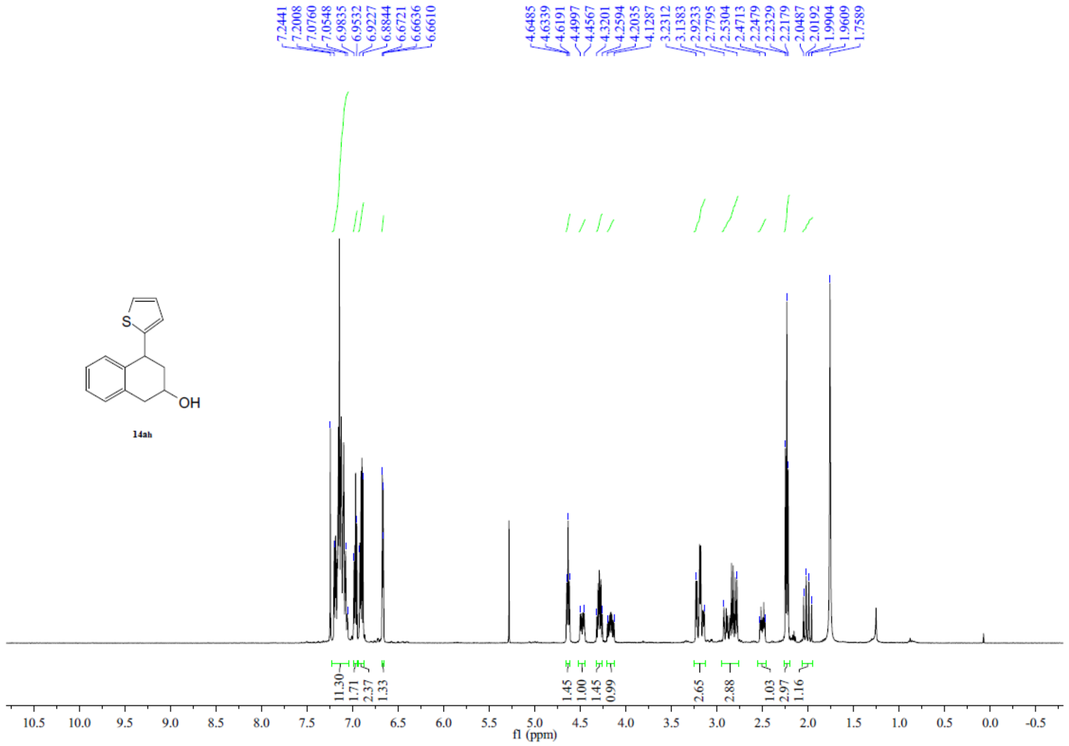

^{13}C NMR ($CDCl_3$, 101 MHz) of 4-(Thiophen-2-yl)-1,2,3,4-tetrahydronaphthalen-2-ol ***(14ah)***

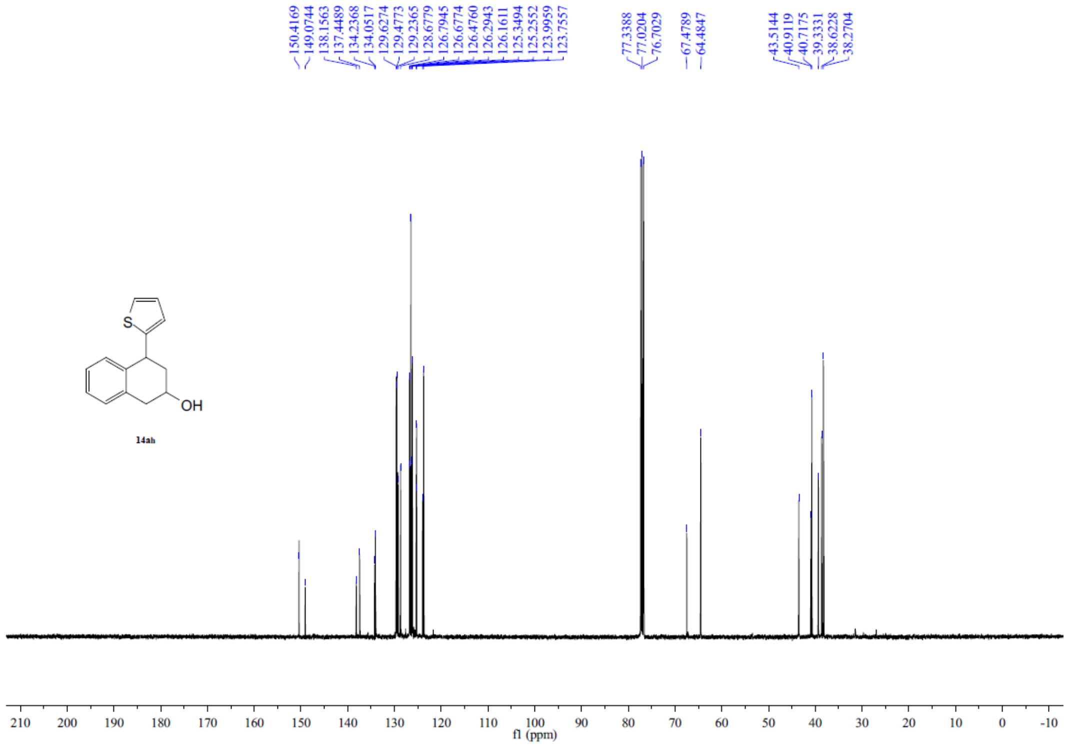

*HSQC of 4-(Thiophen-2-yl)-1,2,3,4-tetrahydronaphthalen-2-ol **(14ah)***

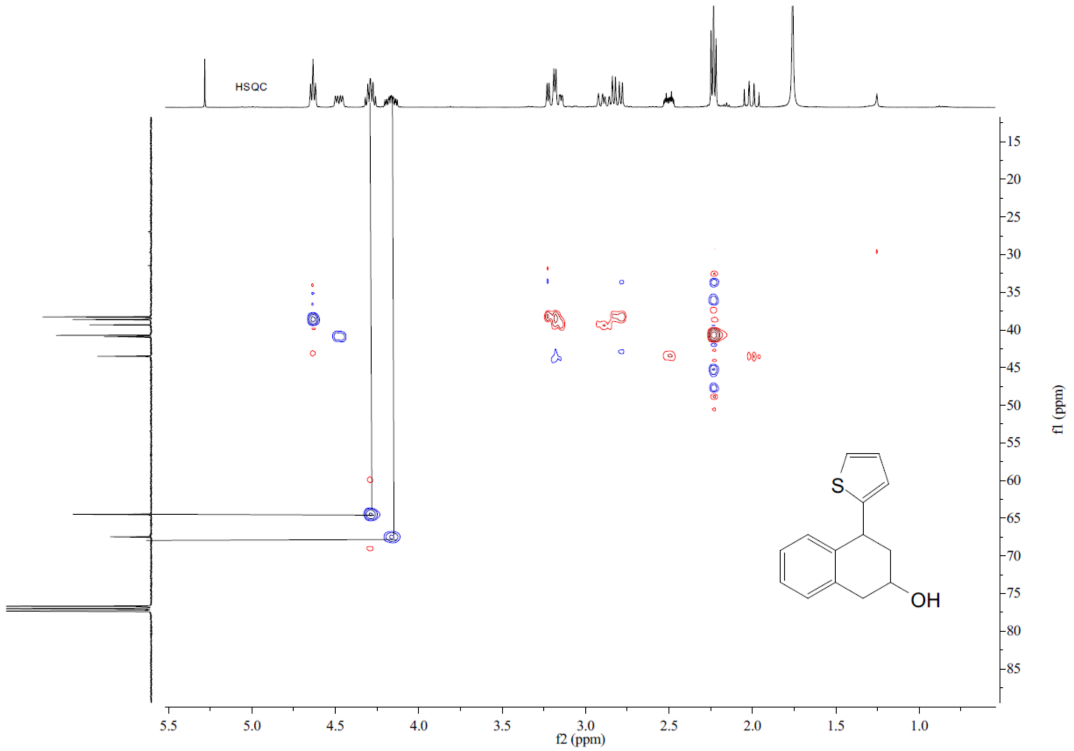

*NOE of 4-(Thiophen-2-yl)-1,2,3,4-tetrahydronaphthalen-2-ol **(14ah)***

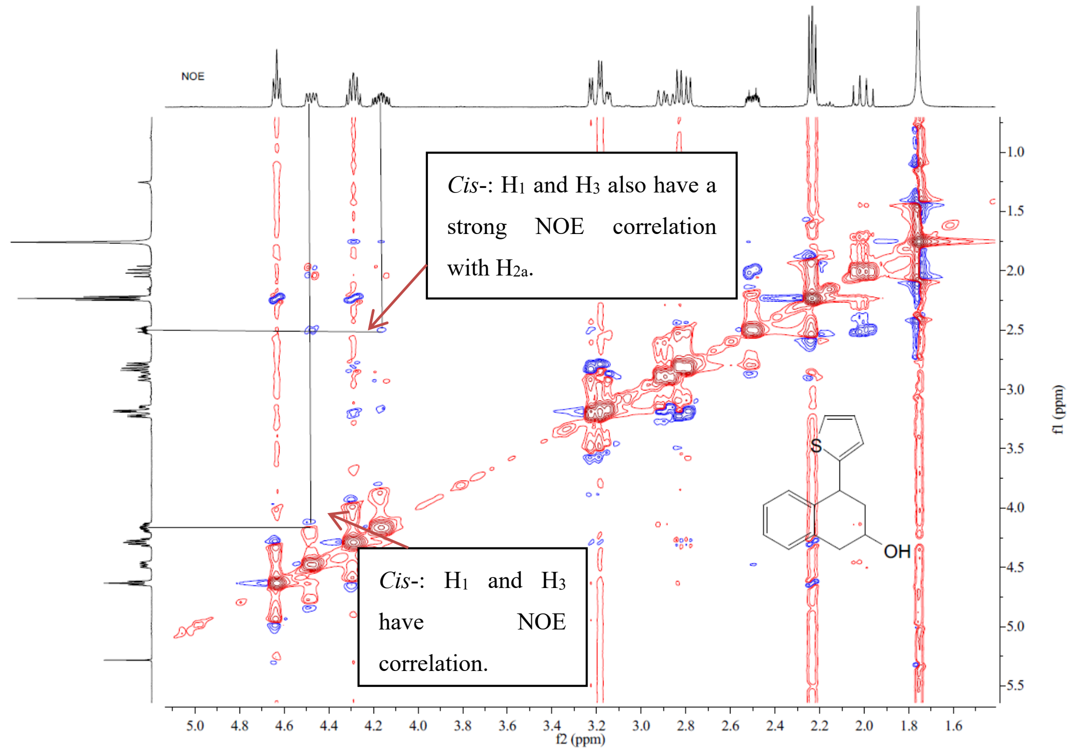

^{1}H NMR ($CDCl_3$, 400 MHz) of 4-Allyl-1,2,3,4-tetrahydronaphthalen-2-ol ***(14ai)***

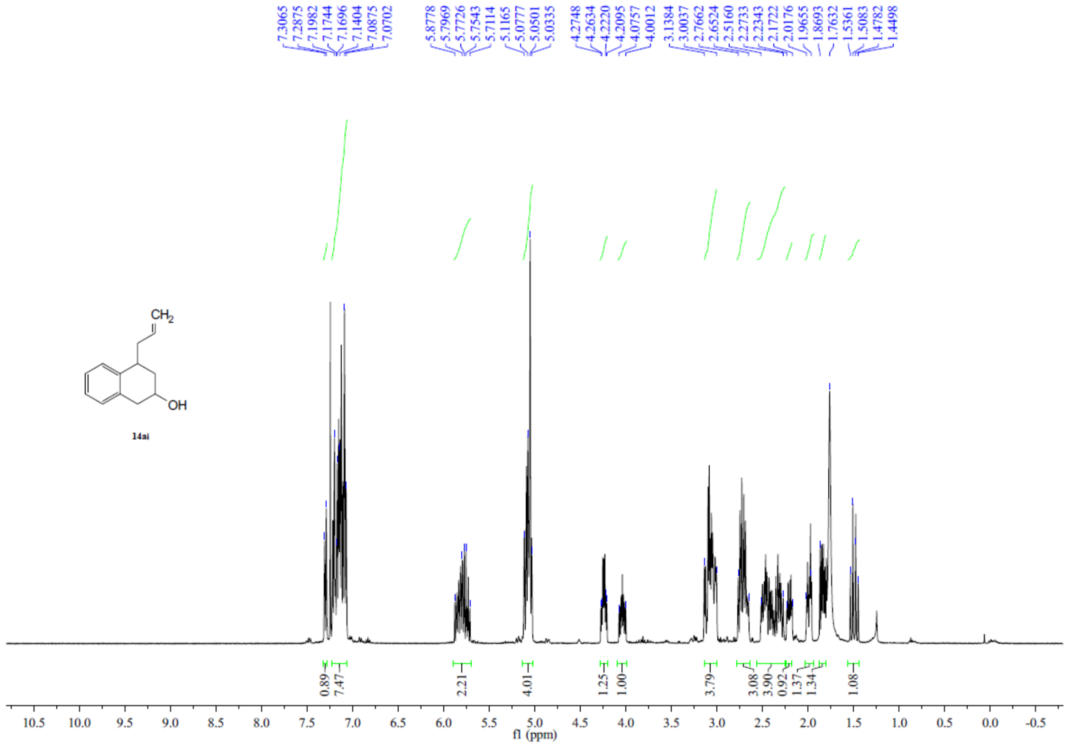

^{13}C NMR ($CDCl_3$, 101 MHz) and 4-Allyl-1,2,3,4-tetrahydronaphthalen-2-ol ***(14ai)***

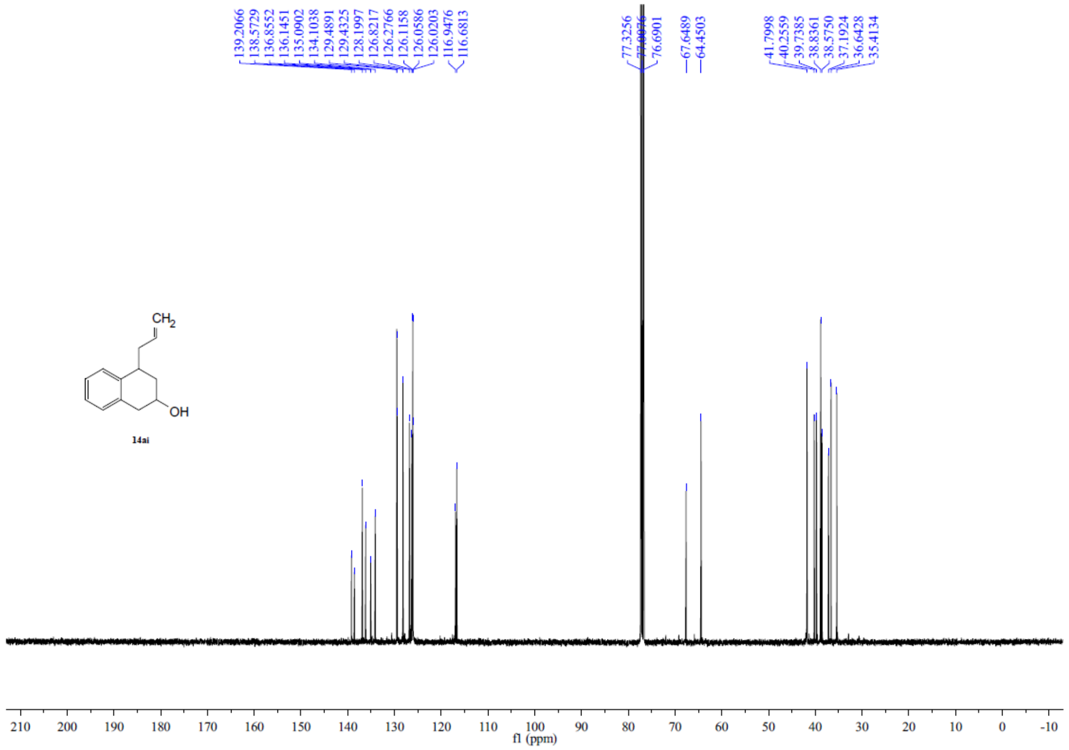

*HSQC of 4-Allyl-1,2,3,4-tetrahydronaphthalen-2-ol **(14ai)***

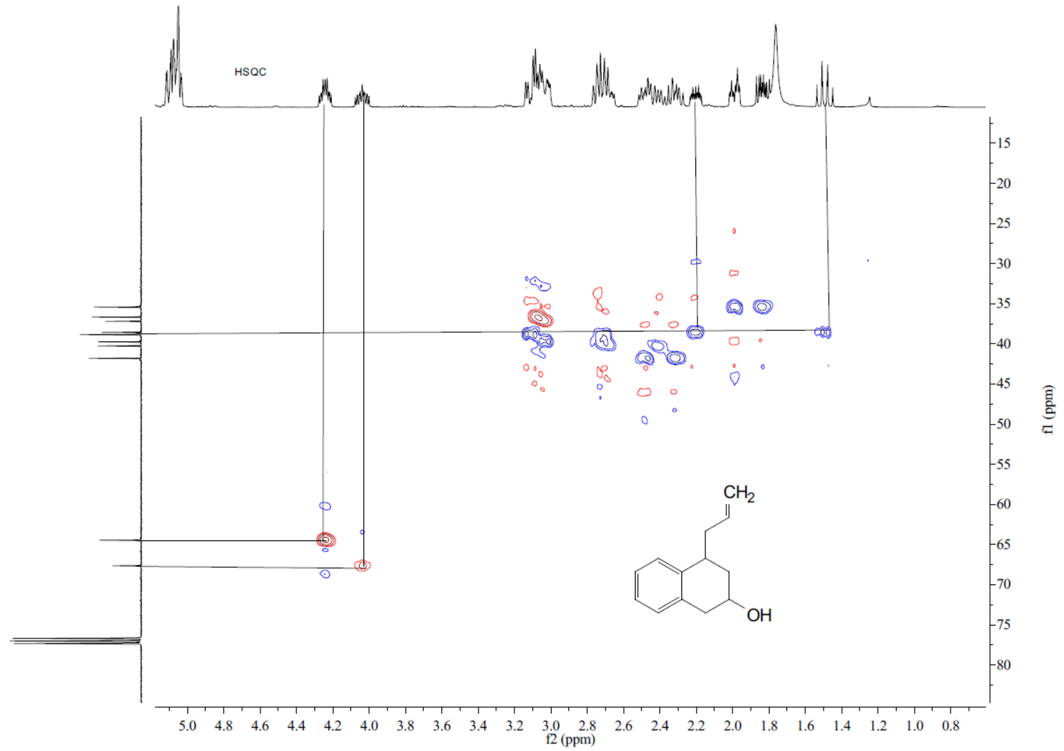

*NOE of 4-Allyl-1,2,3,4-tetrahydronaphthalen-2-ol **(14ai)***

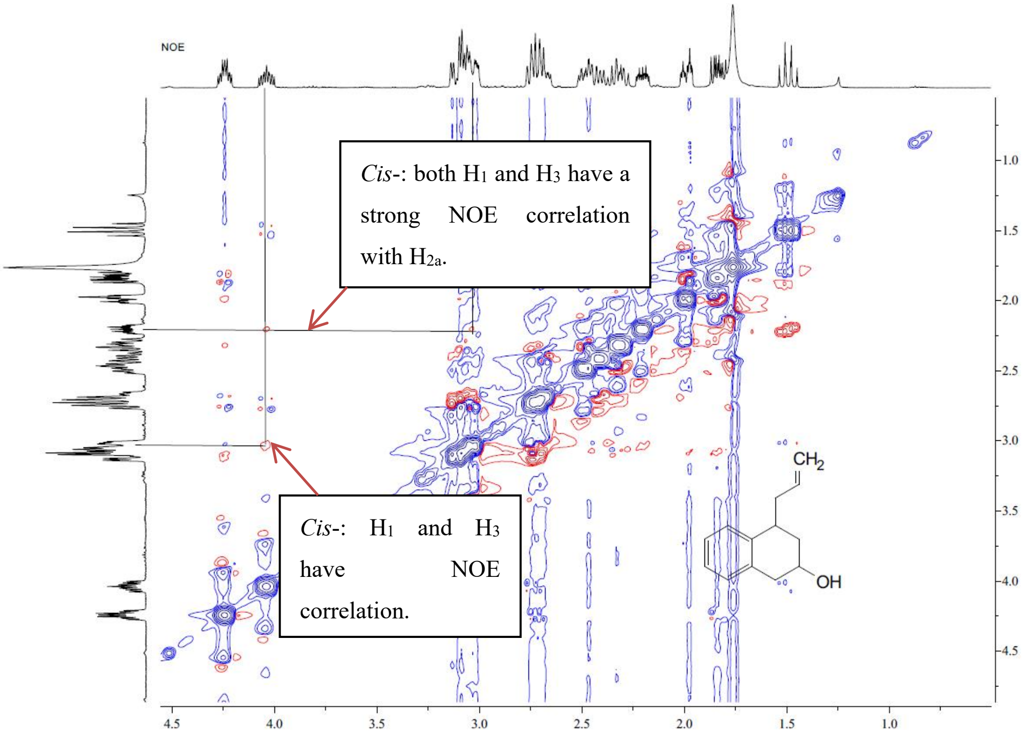

^{1}H NMR ($CDCl_3$, 400 MHz) of 1,2,3,4-Tetrahydro-[1,1'-binaphthalene]-3,4'-diol **(14aj)**

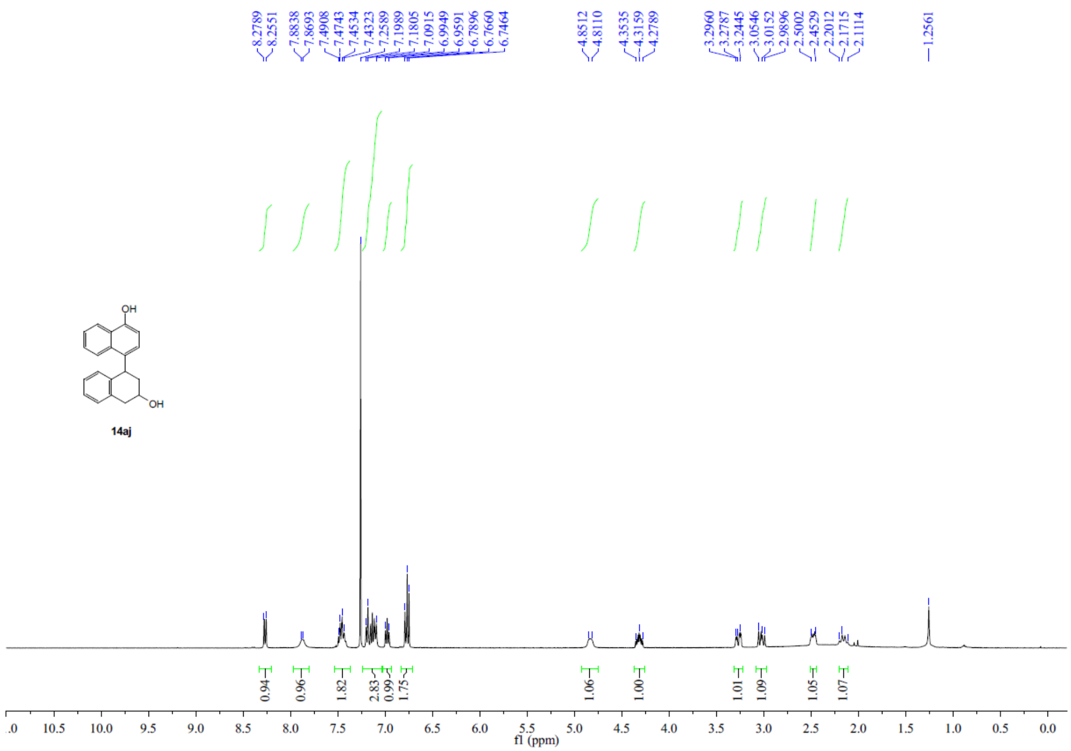

^{13}C NMR ($CDCl_3$, 101 MHz) of 1,2,3,4-Tetrahydro-[1,1'-binaphthalene]-3,4'-diol **(14aj)**

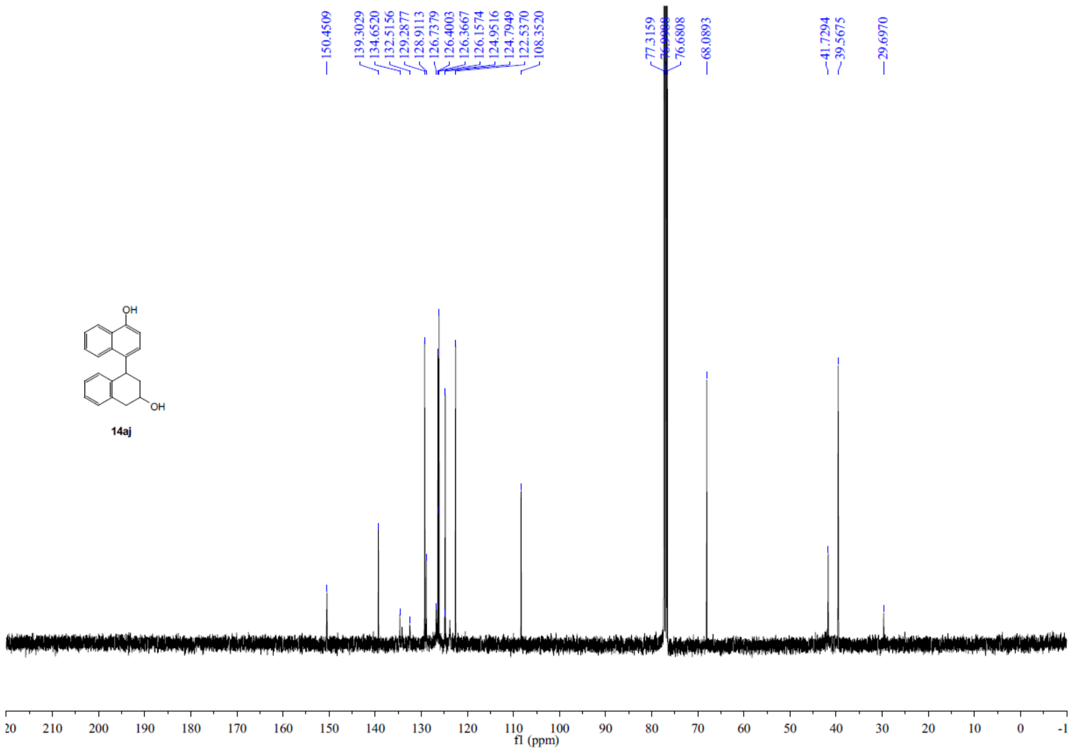

HSQC of 1,2,3,4-Tetrahydro-[1,1'-binaphthalene]-3,4'-diol ***(14aj)***

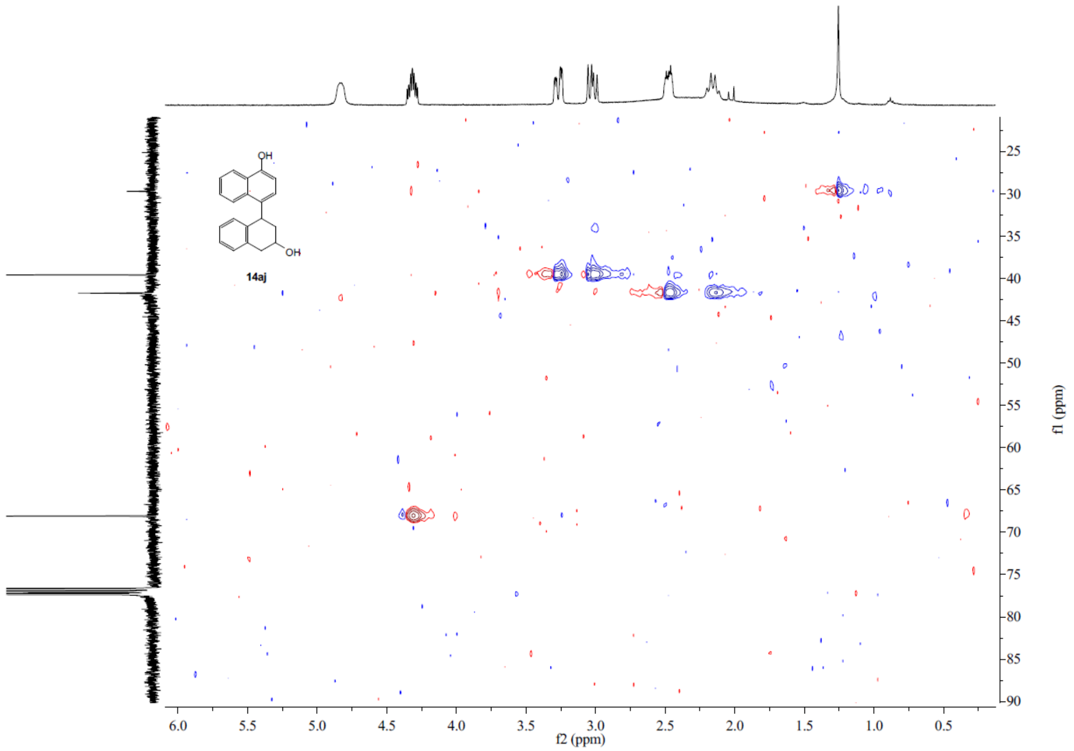

NOE of 1,2,3,4-Tetrahydro-[1,1'-binaphthalene]-3,4'-diol ***(14aj)***

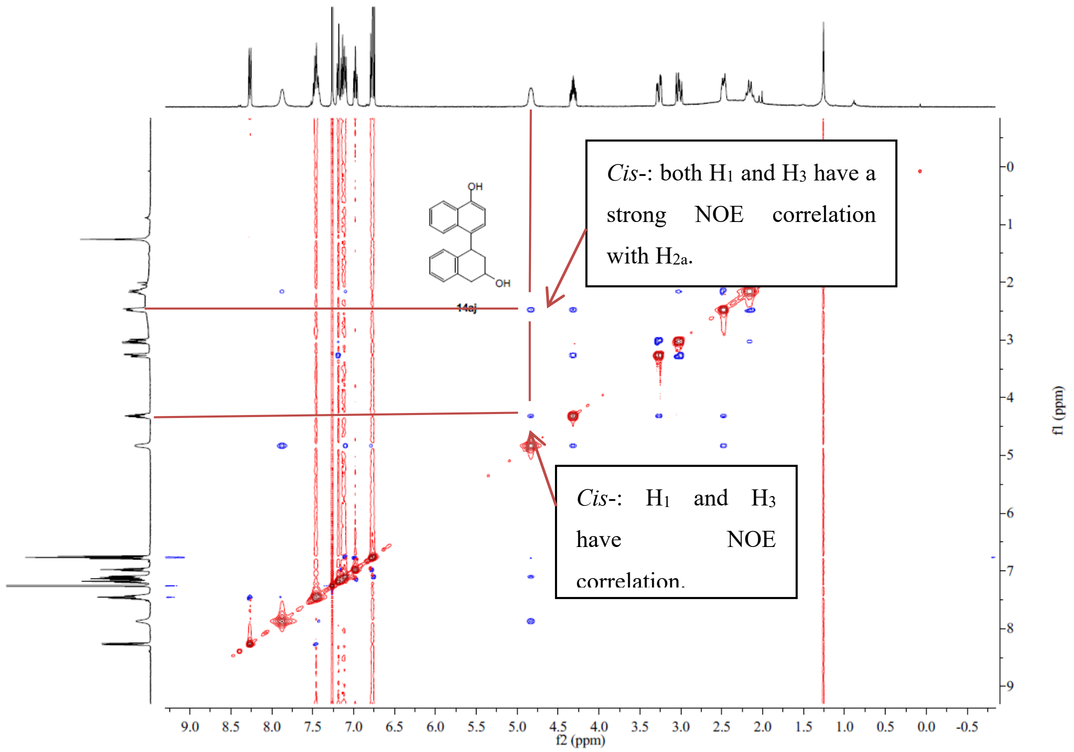

^{1}H NMR ($CDCl_3$, 400 MHz) of 4-(3,4-Dimethoxyphenyl)-6-methoxy-1,2,3,4-tetrahydronaphthalen-2-ol ***(14ba)***

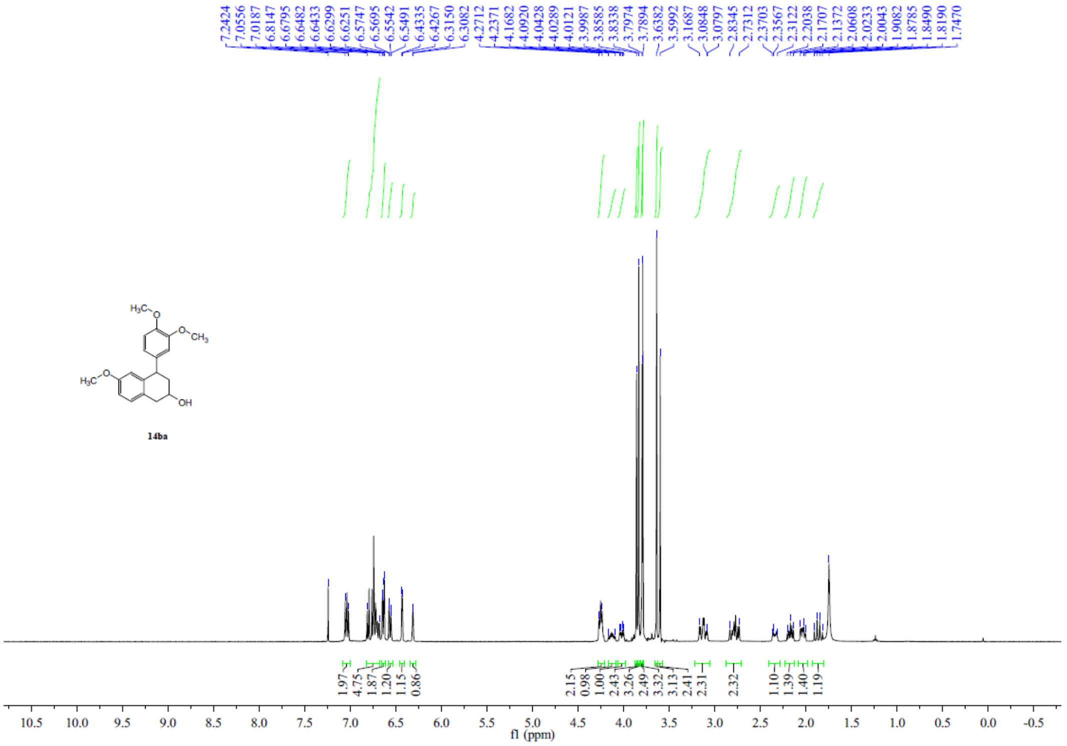

^{13}C NMR ($CDCl_3$, 101 MHz) of 4-(3,4-Dimethoxyphenyl)-6-methoxy-1,2,3,4-tetrahydronaphthalen-2-ol ***(14ba)***

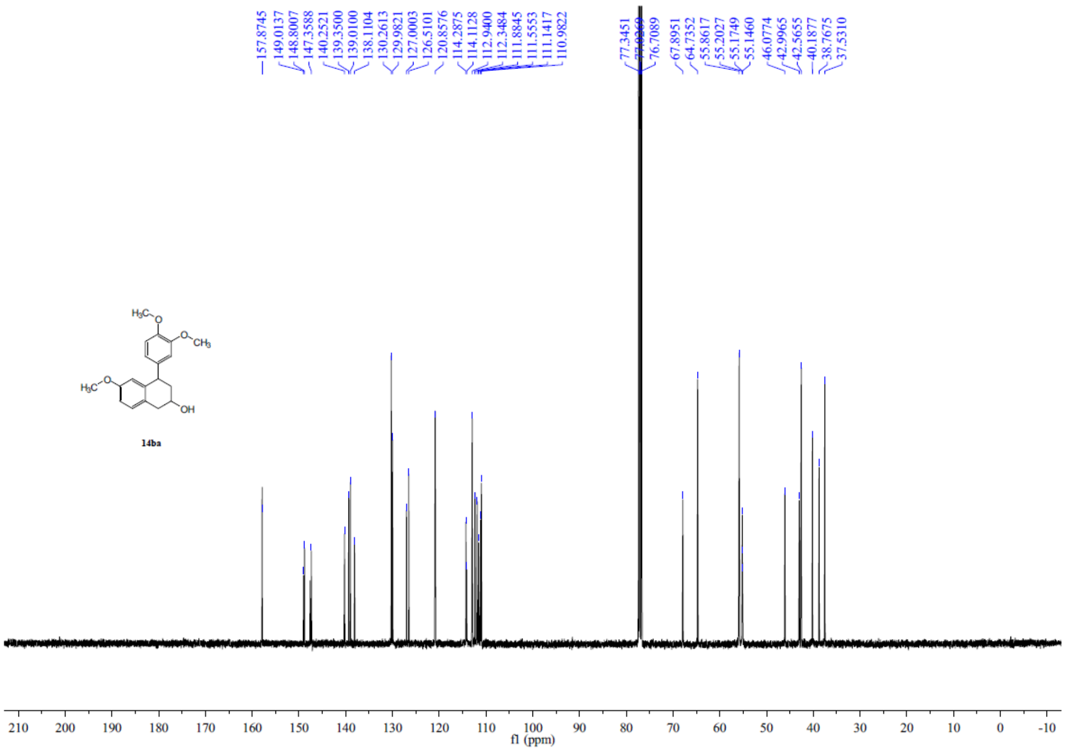

*HSQC of 4-(3,4-Dimethoxyphenyl)-6-methoxy-1,2,3,4-tetrahydronaphthalen-2-ol **(14ba)***

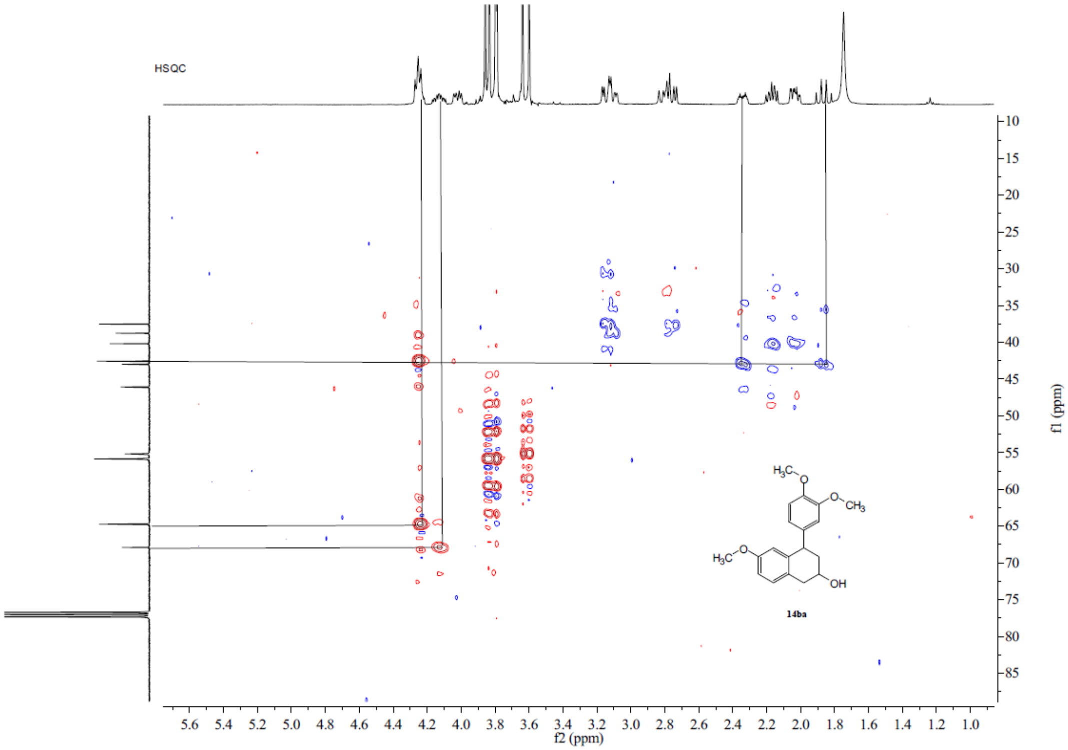

*NOE of 4-(3,4-Dimethoxyphenyl)-6-methoxy-1,2,3,4-tetrahydronaphthalen-2-ol **(14ba)***

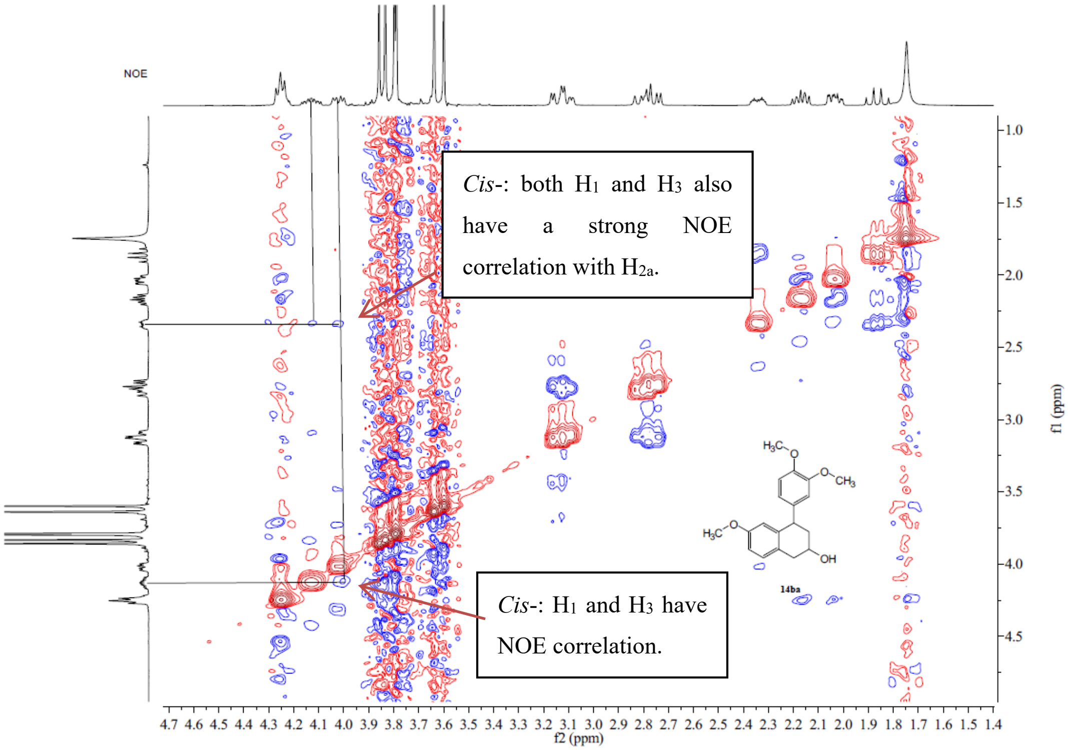

^{1}H NMR ($CDCl_3$, 400 MHz)of 4-(Furan-2-yl)-6-methoxy-1,2,3,4-tetrahydronaphthalen-2-ol ***(14bb)***

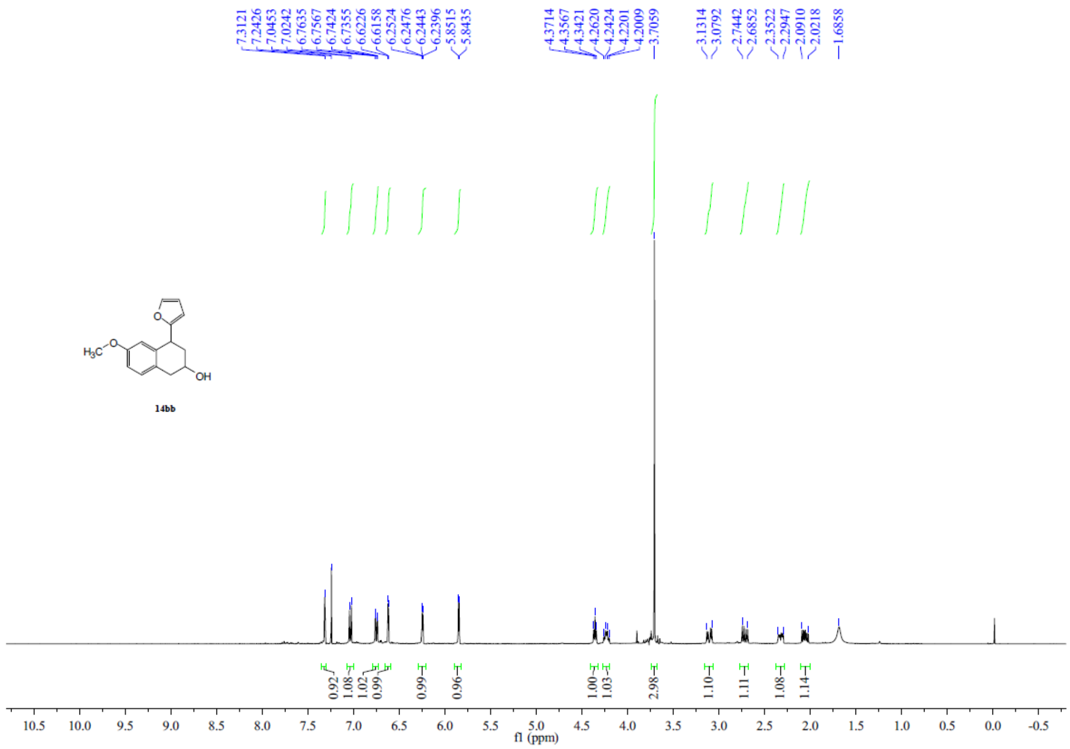

^{13}C NMR ($CDCl_3$, 101 MHz) of 4-(Furan-2-yl)-6-methoxy-1,2,3,4-tetrahydronaphthalen-2-ol ***(14bb)***

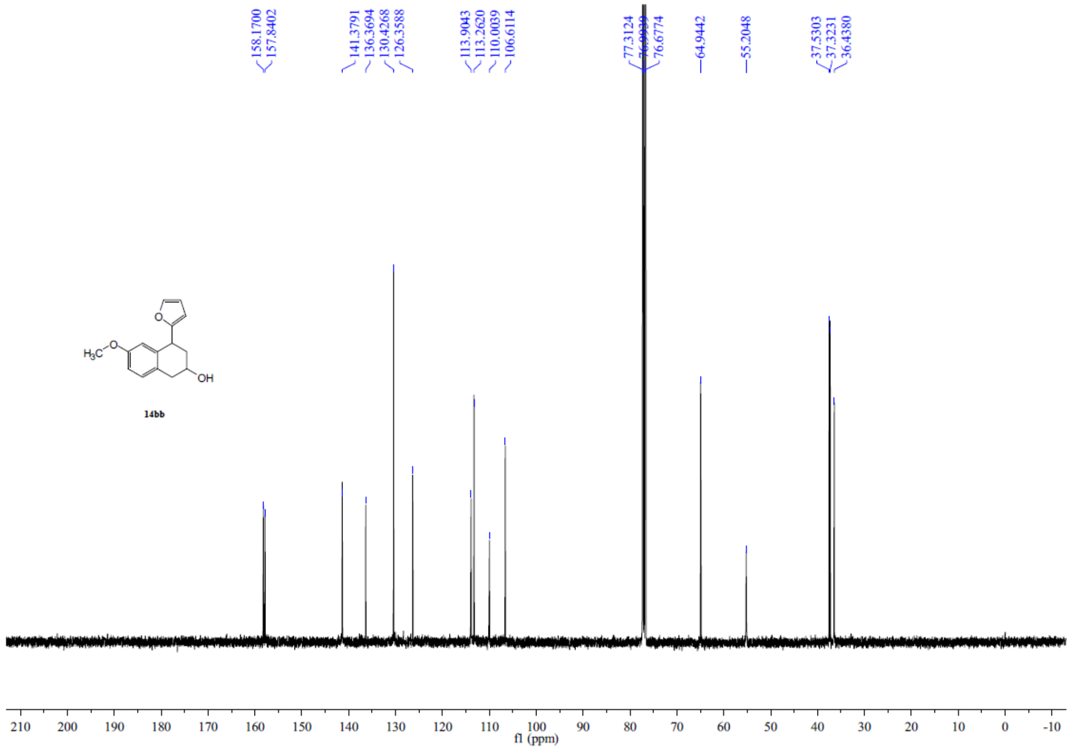

HSQC of 4-(Furan-2-yl)-6-methoxy-1,2,3,4-tetrahydronaphthalen-2-ol ***(14bb)***

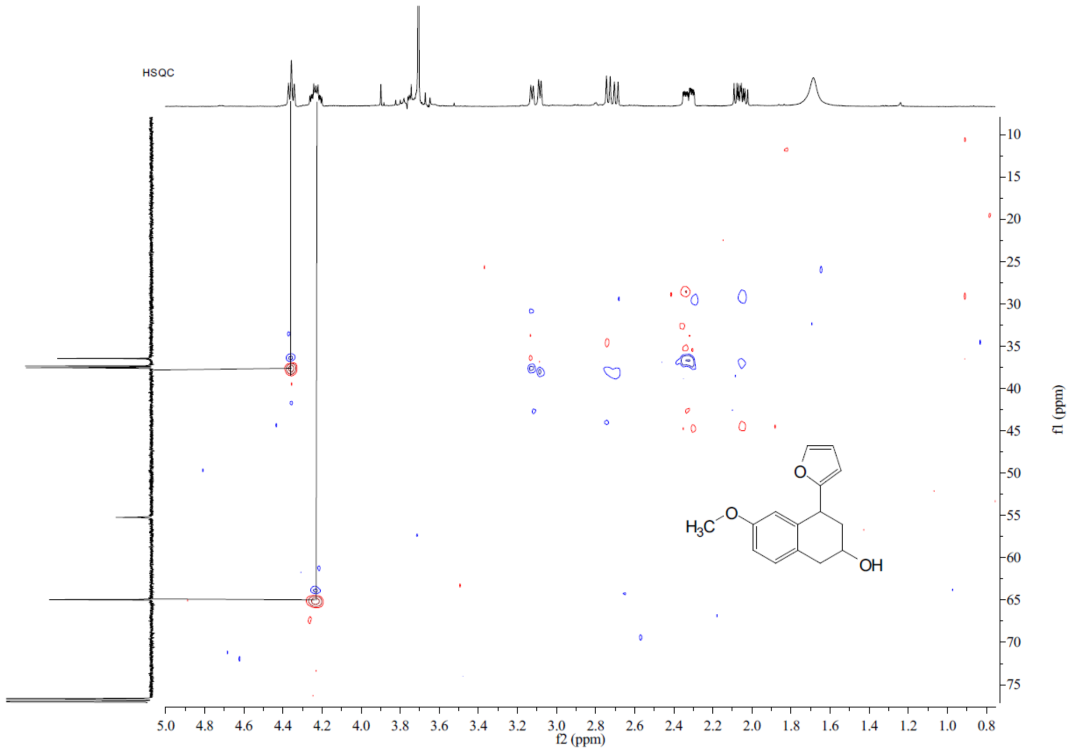

NOE of 4-(Furan-2-yl)-6-methoxy-1,2,3,4-tetrahydronaphthalen-2-ol ***(14bb)***

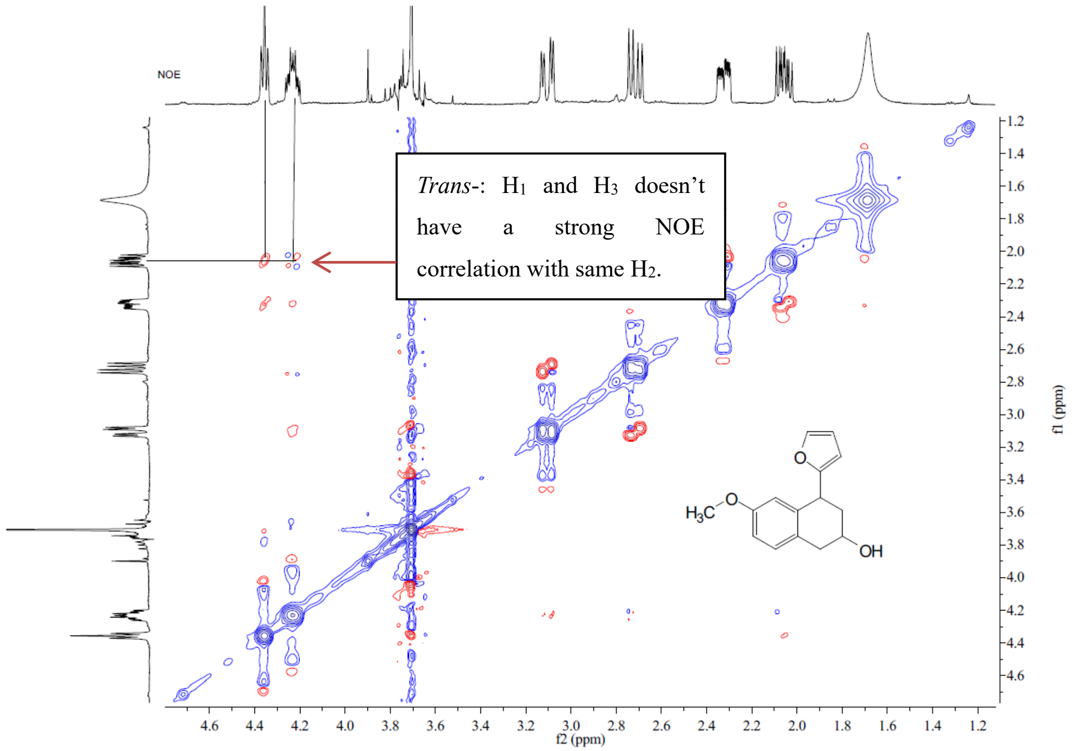

^{1}H NMR ($CDCl_3$, 400 MHz) of *6-Chloro-4-(3,4-dimethoxyphenyl)-1,2,3,4-tetrahydronaphthalen-2-ol* ***(14ca)***

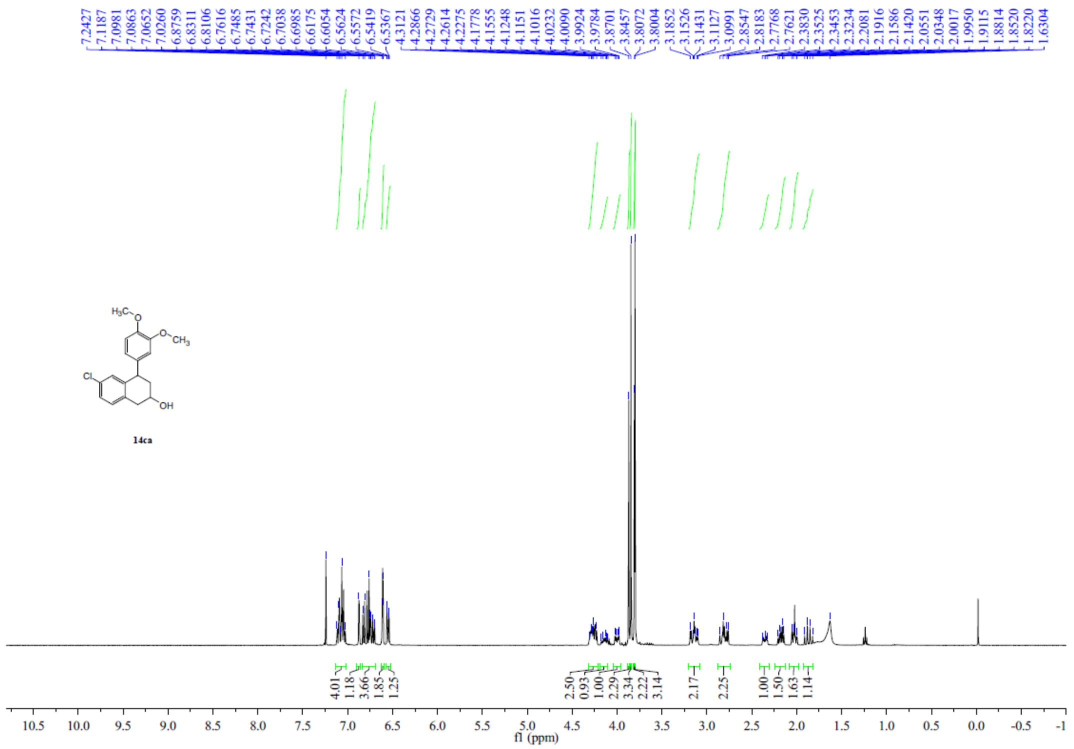

^{13}C NMR ($CDCl_3$, 101 MHz) of *6-Chloro-4-(3,4-dimethoxyphenyl)-1,2,3,4-tetrahydronaphthalen-2-ol* ***(14ca)***

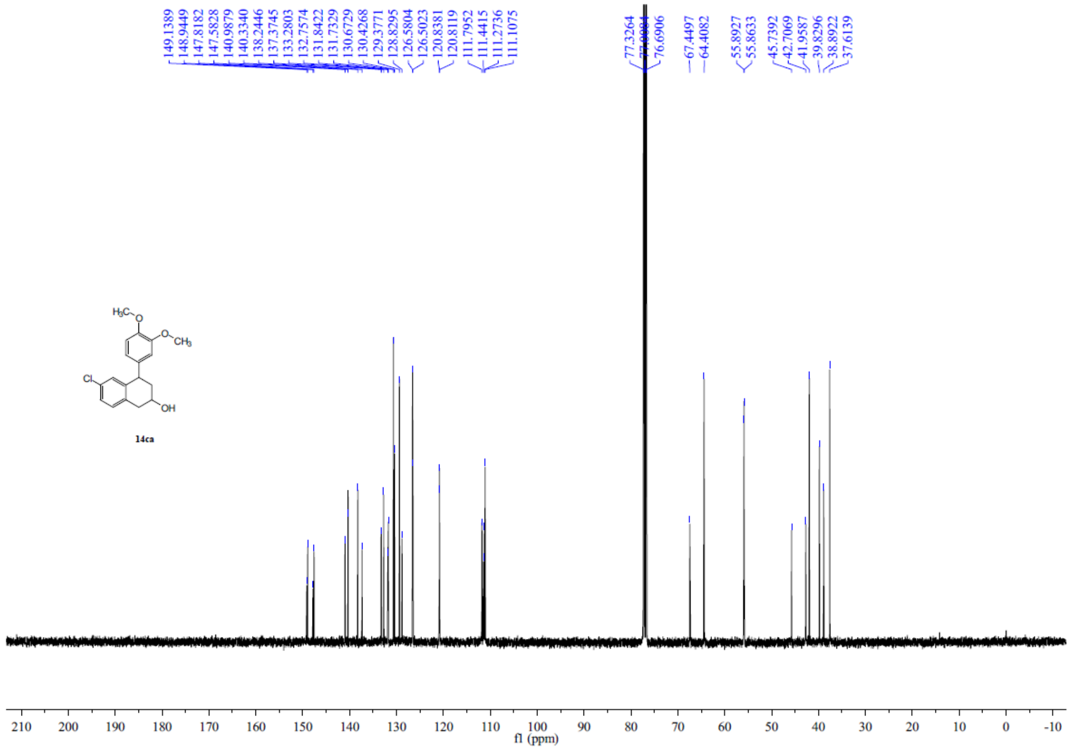

*HSQC of 6-Chloro-4-(3,4-dimethoxyphenyl)-1,2,3,4-tetrahydronaphthalen-2-ol **(14ca)***

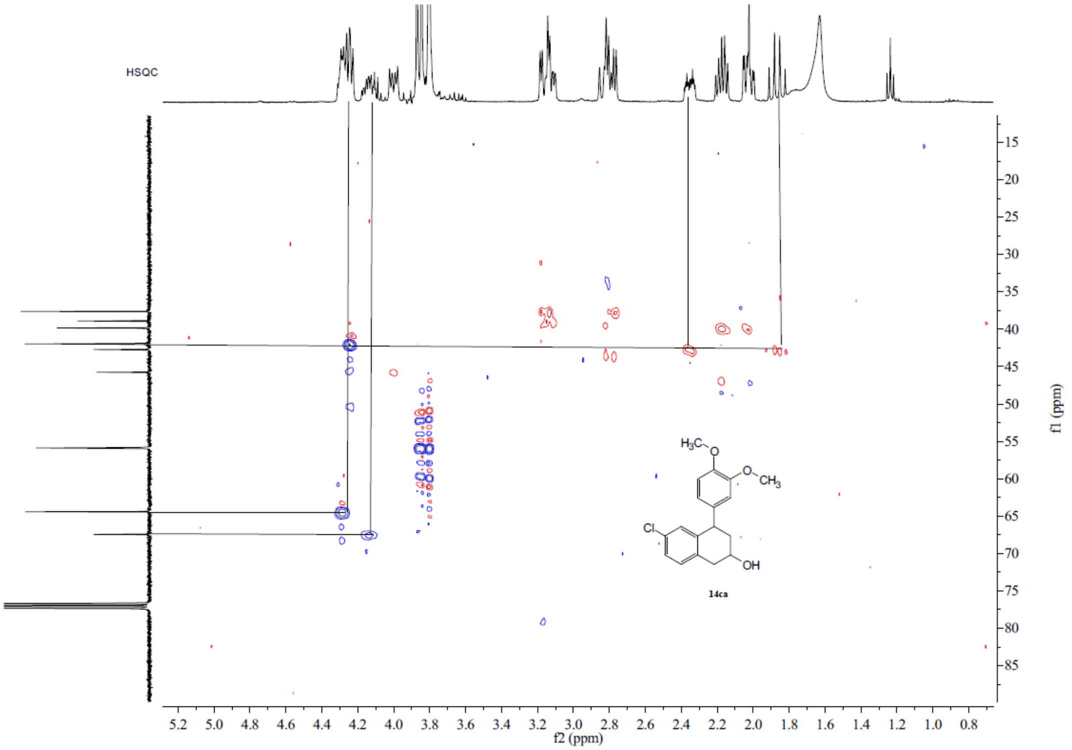

*NOE of 6-Chloro-4-(3,4-dimethoxyphenyl)-1,2,3,4-tetrahydronaphthalen-2-ol **(14ca)***

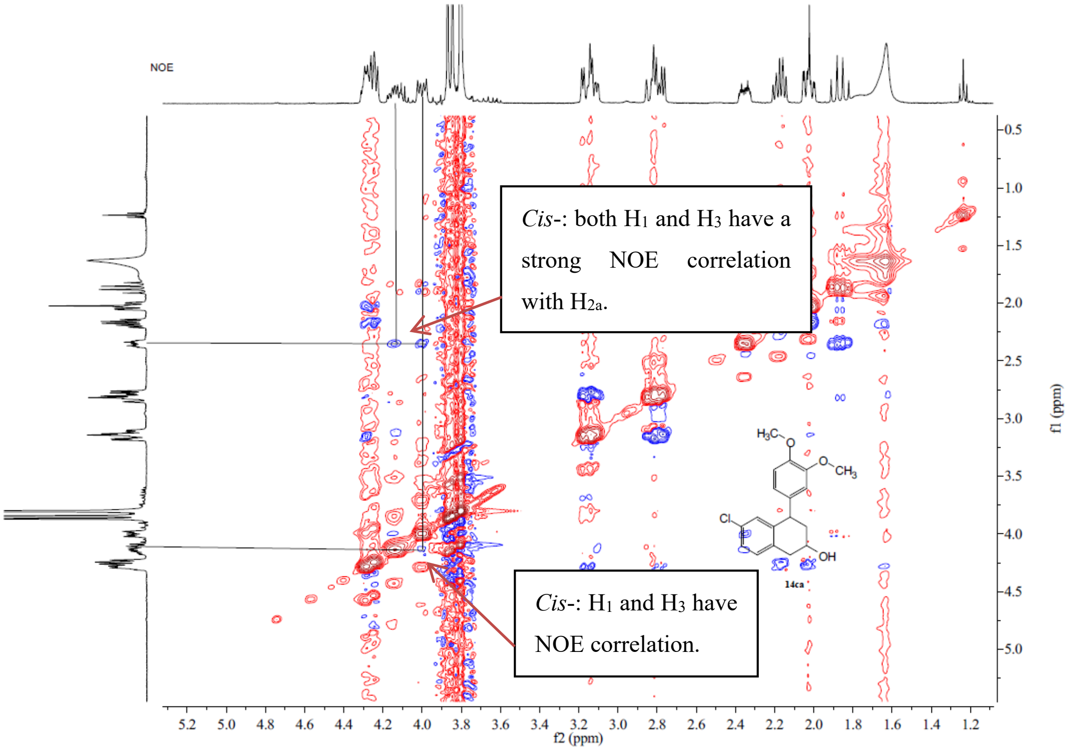

^{1}H NMR (CDCl$_3$, 400 MHz) of 6-Chloro-4-(furan-2-yl)-1,2,3,4-tetrahydronaphthalen-2-ol ***(14cb)***

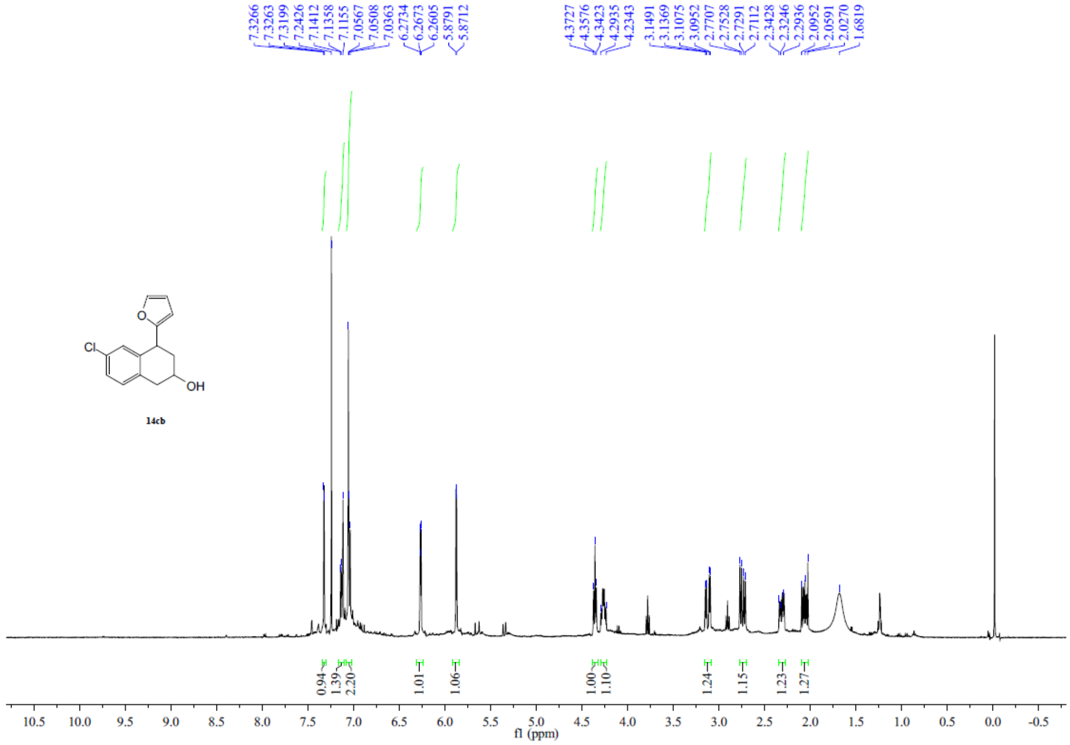

^{13}C NMR (CDCl$_3$, 101 MHz) of 6-Chloro-4-(furan-2-yl)-1,2,3,4-tetrahydronaphthalen-2-ol ***(14cb)***

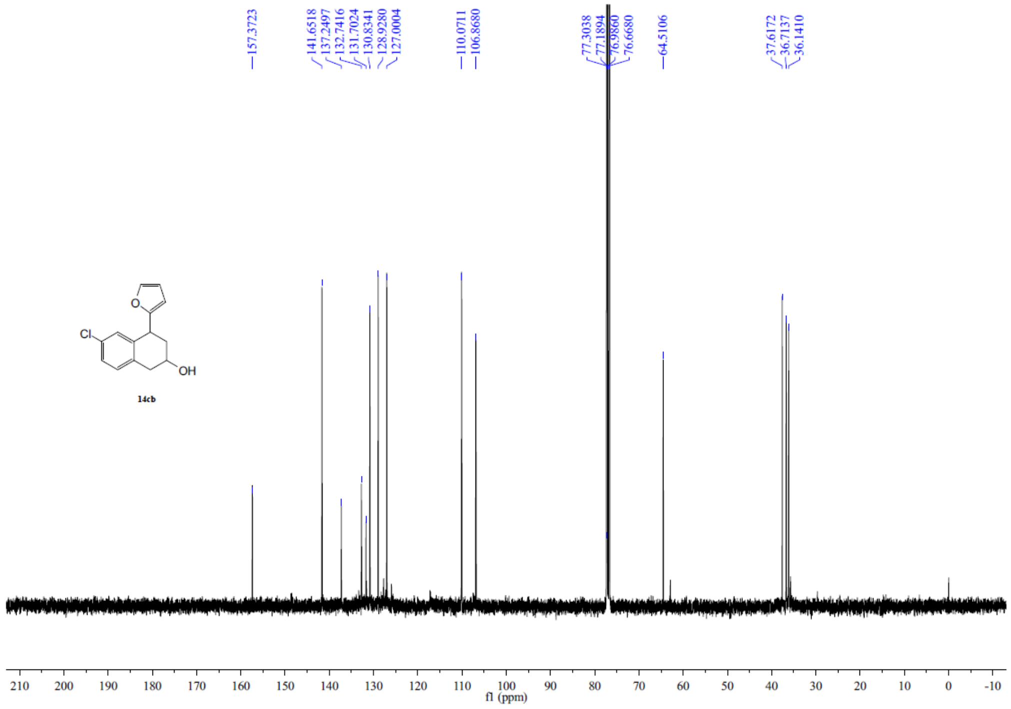

*HSQC of 6-Chloro-4-(furan-2-yl)-1,2,3,4-tetrahydronaphthalen-2-ol **(14cb)***

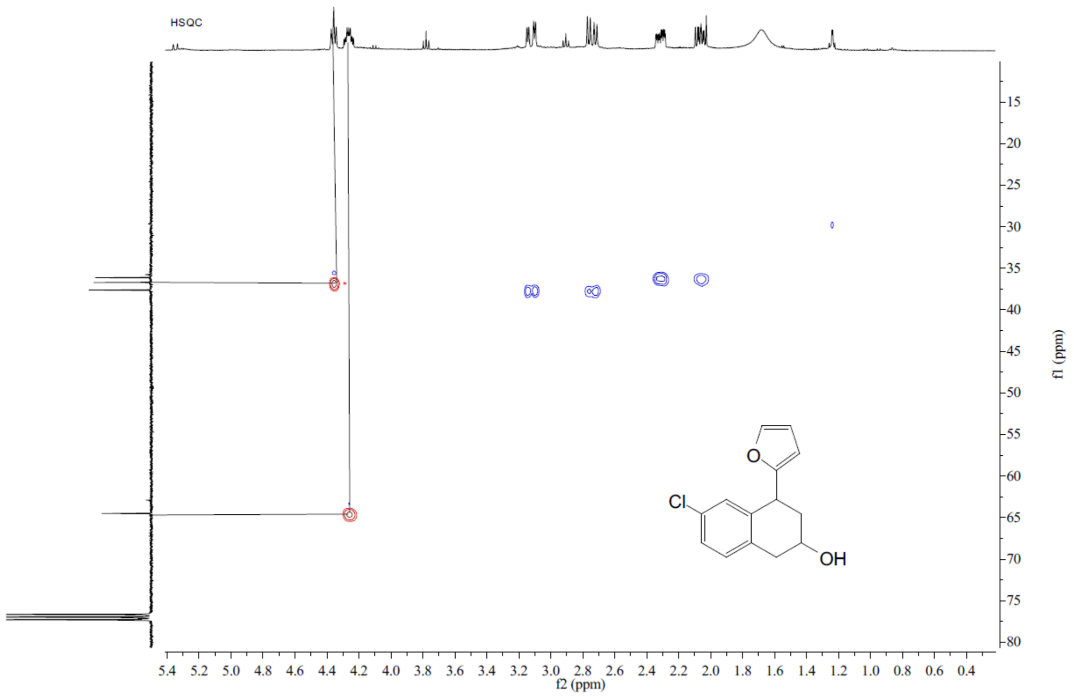

*NOE of 6-Chloro-4-(furan-2-yl)-1,2,3,4-tetrahydronaphthalen-2-ol **(14cb)***

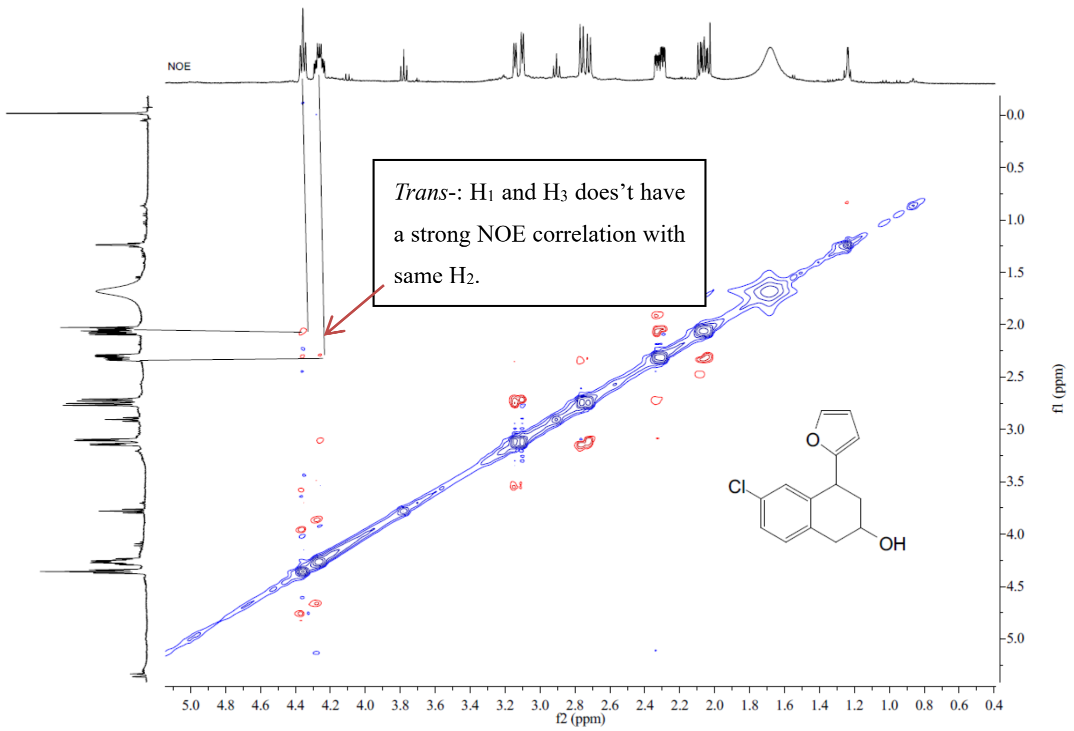

^{1}H NMR ($CDCl_3$, 400 MHz) of 4-(3,4-Dimethoxyphenyl)-6-nitro-1,2,3,4-tetrahydronaphthalen-2-ol ***(14da)***

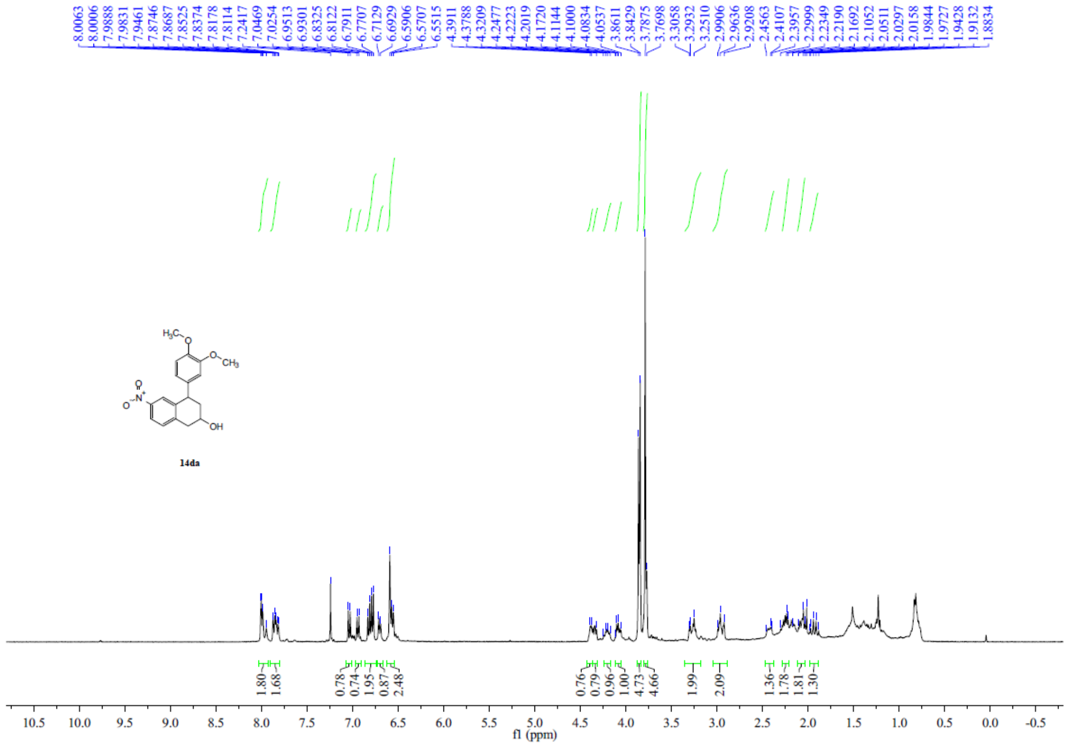

^{13}C NMR ($CDCl_3$, 101 MHz) of 4-(3,4-Dimethoxyphenyl)-6-nitro-1,2,3,4-tetrahydronaphthalen-2-ol ***(14da)***

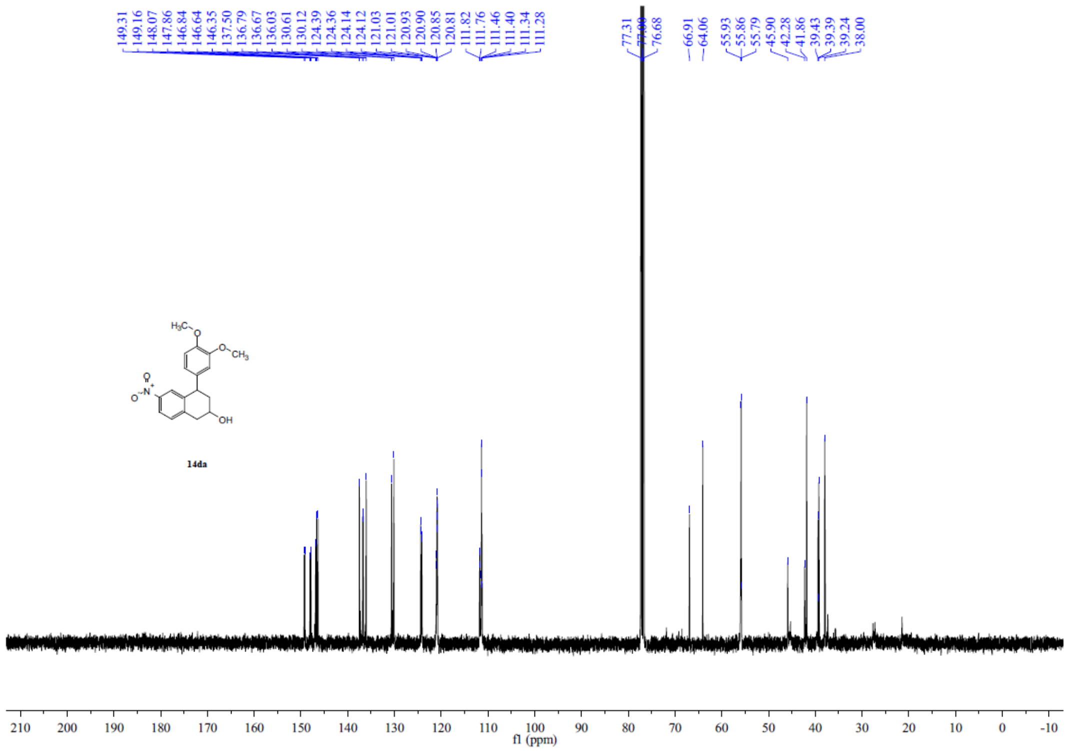

*HSQC of 4-(3,4-Dimethoxyphenyl)-6-nitro-1,2,3,4-tetrahydronaphthalen-2-ol **(14da)***

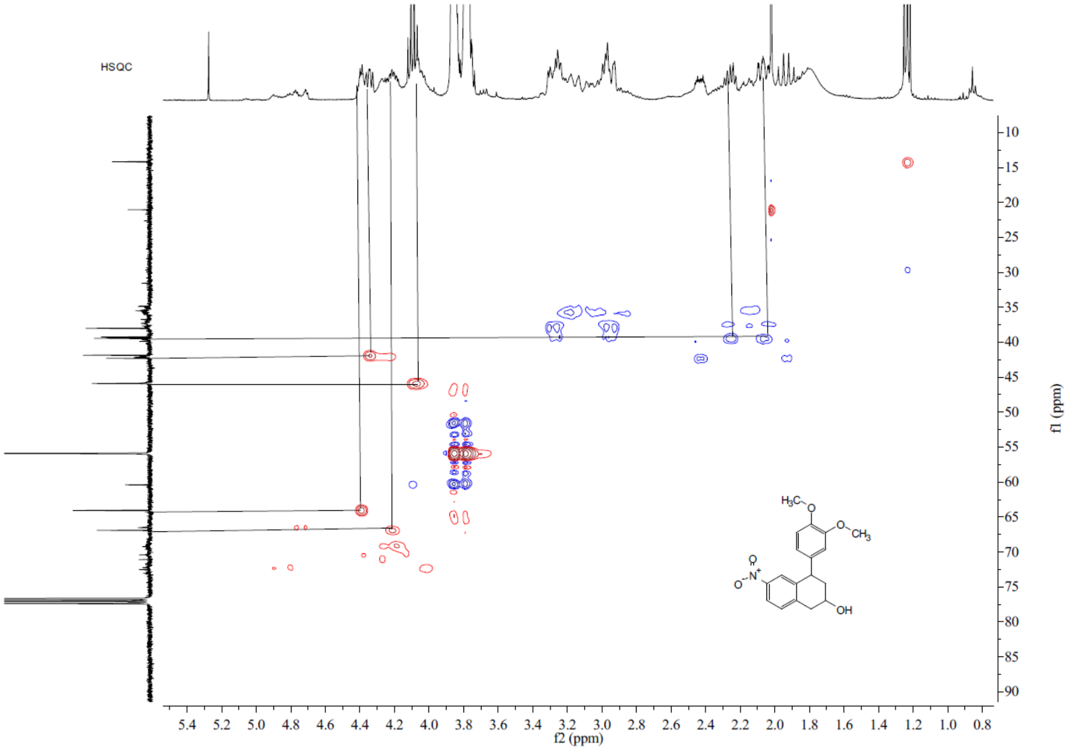

*NOE of 4-(3,4-Dimethoxyphenyl)-6-nitro-1,2,3,4-tetrahydronaphthalen-2-ol **(14da)***

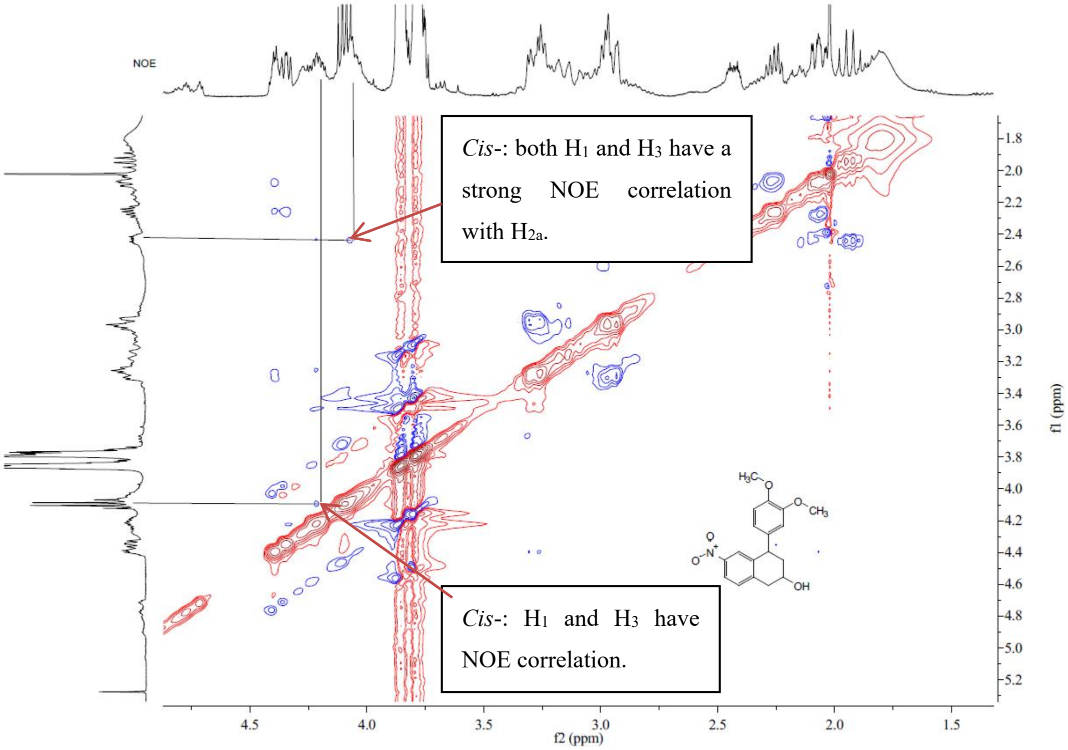

^{1}H NMR ($CDCl_3$, 400 MHz) of 4-(3,4-Dimethoxyphenyl)-1-methyl-1,2,3,4-tetrahydronaphthalen-2-ol ***(14ea)***

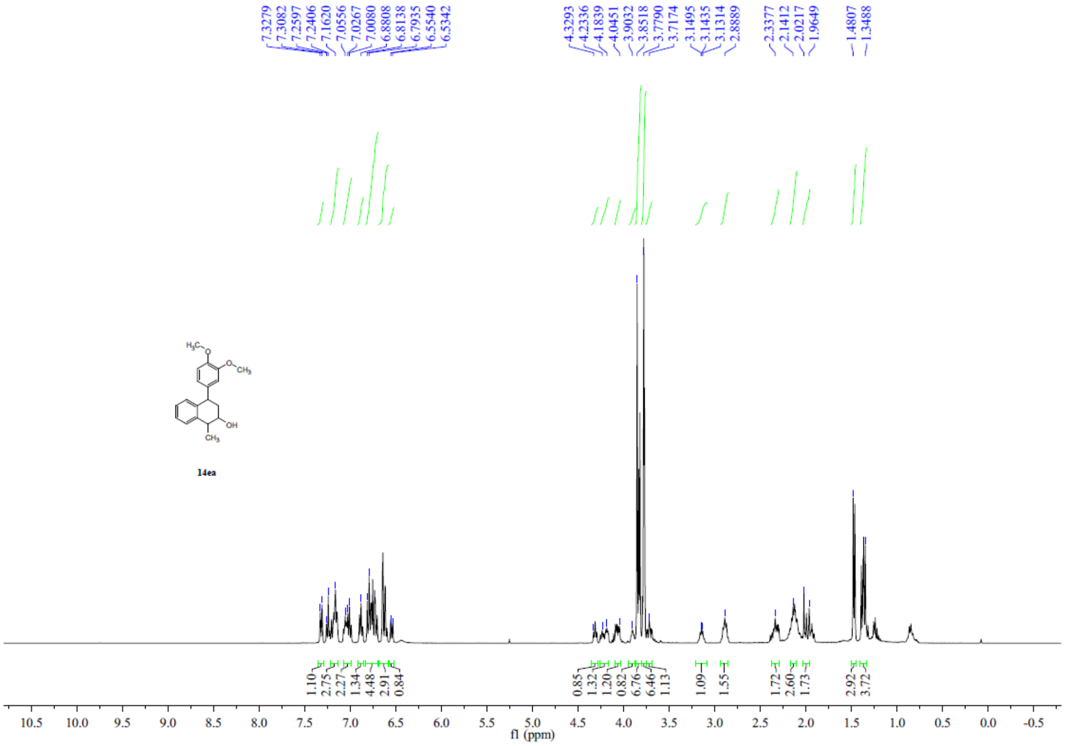

^{13}C NMR ($CDCl_3$, 101 MHz) of 4-(3,4-Dimethoxyphenyl)-1-methyl-1,2,3,4-tetrahydronaphthalen-2-ol ***(14ea)***

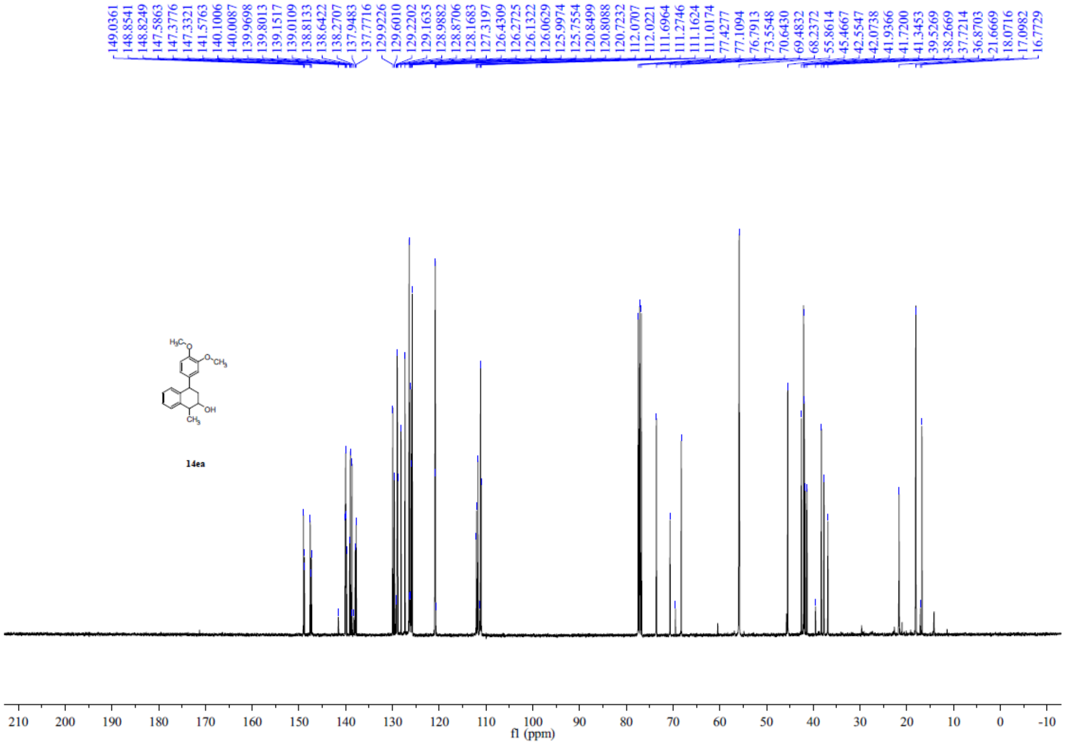

^{1}H NMR ($CDCl_3$, 400 MHz) of 4-(Furan-2-yl)-1-methyl-1,2,3,4-tetrahydronaphthalen-2-ol ***(14eb)***

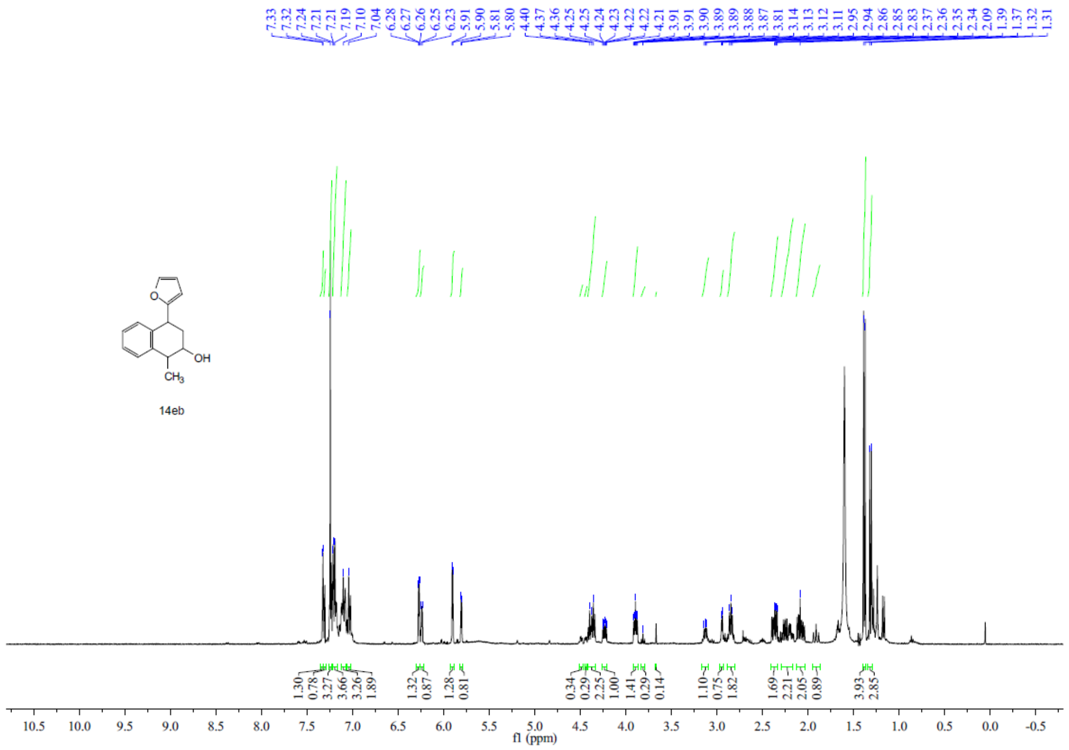

^{13}C NMR ($CDCl_3$, 101 MHz) of 4-(Furan-2-yl)-1-methyl-1,2,3,4-tetrahydronaphthalen-2-ol ***(14eb)***

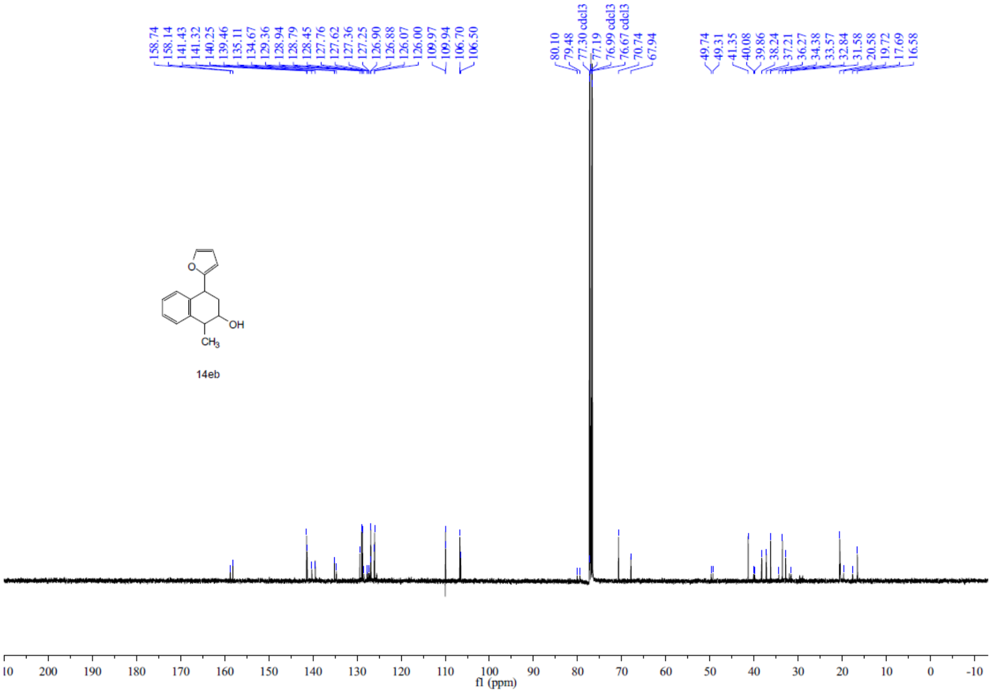

^{1}H NMR ($CDCl_3$, 400 MHz) of 4-(3,4-Dimethoxyphenyl)-4-methyl-1,2,3,4-tetrahydronaphthalen-2-ol ***(14fa)***

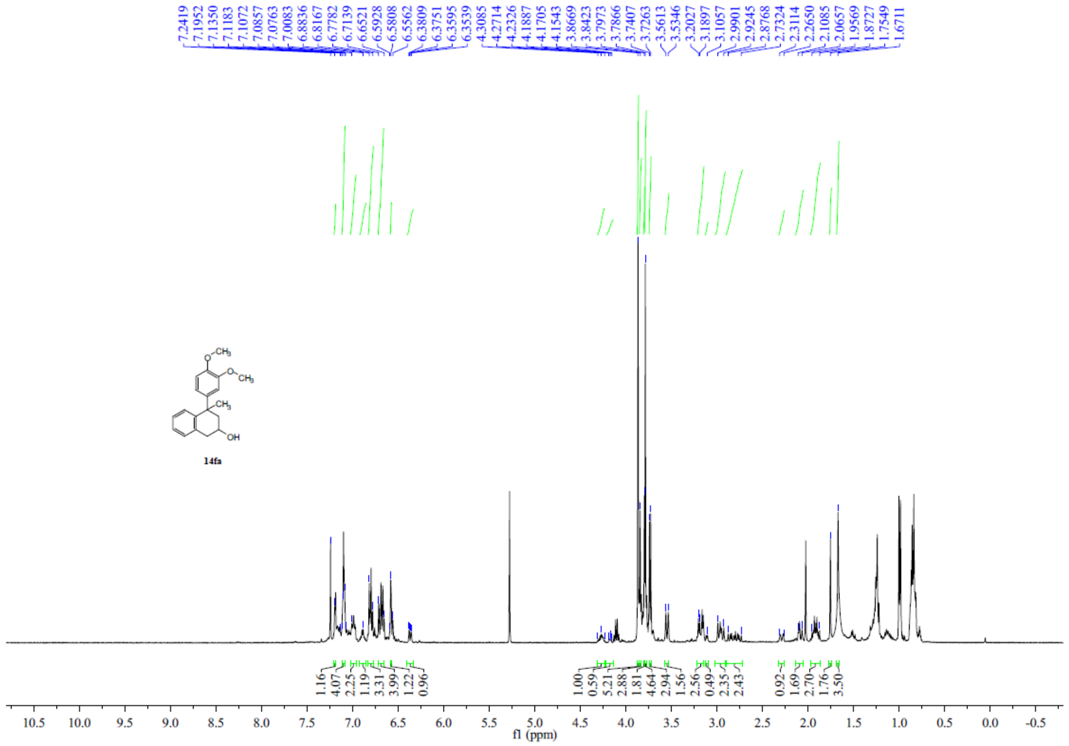

^{13}C NMR ($CDCl_3$, 101 MHz) of 4-(3,4-Dimethoxyphenyl)-4-methyl-1,2,3,4-tetrahydronaphthalen-2-ol ***(14fa)***

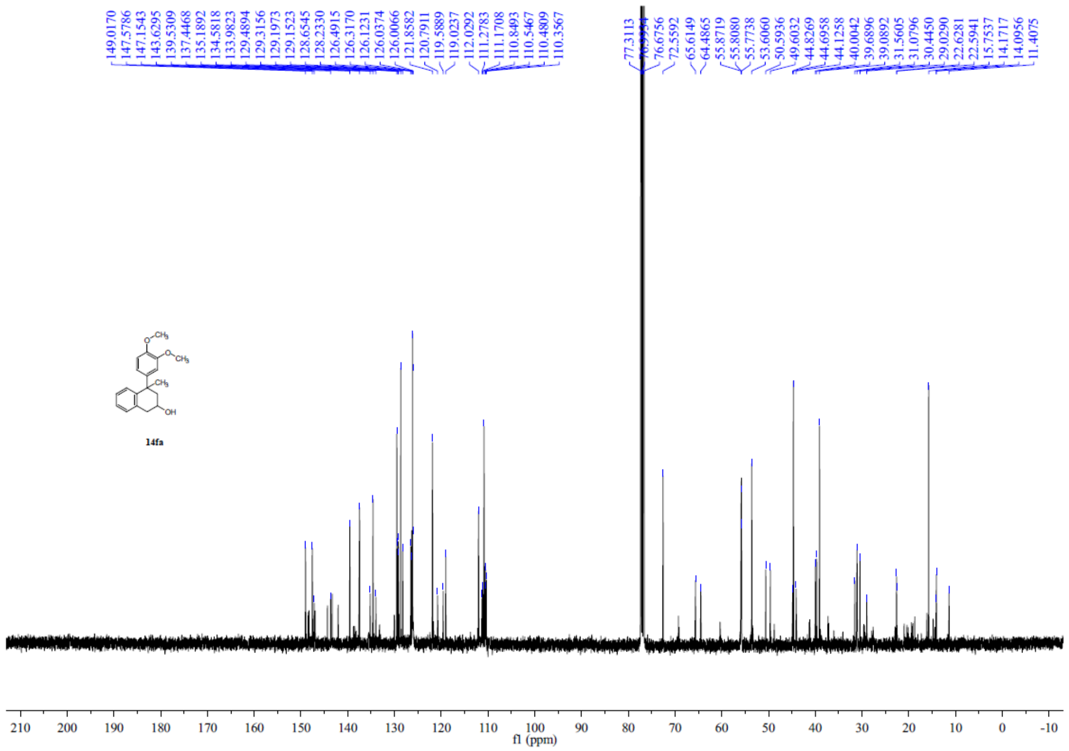

^{1}H NMR ($CDCl_3$, 400 MHz) of 4-(Furan-2-yl)-4-methyl-1,2,3,4-tetrahydronaphthalen-2-ol ***(14fb)***

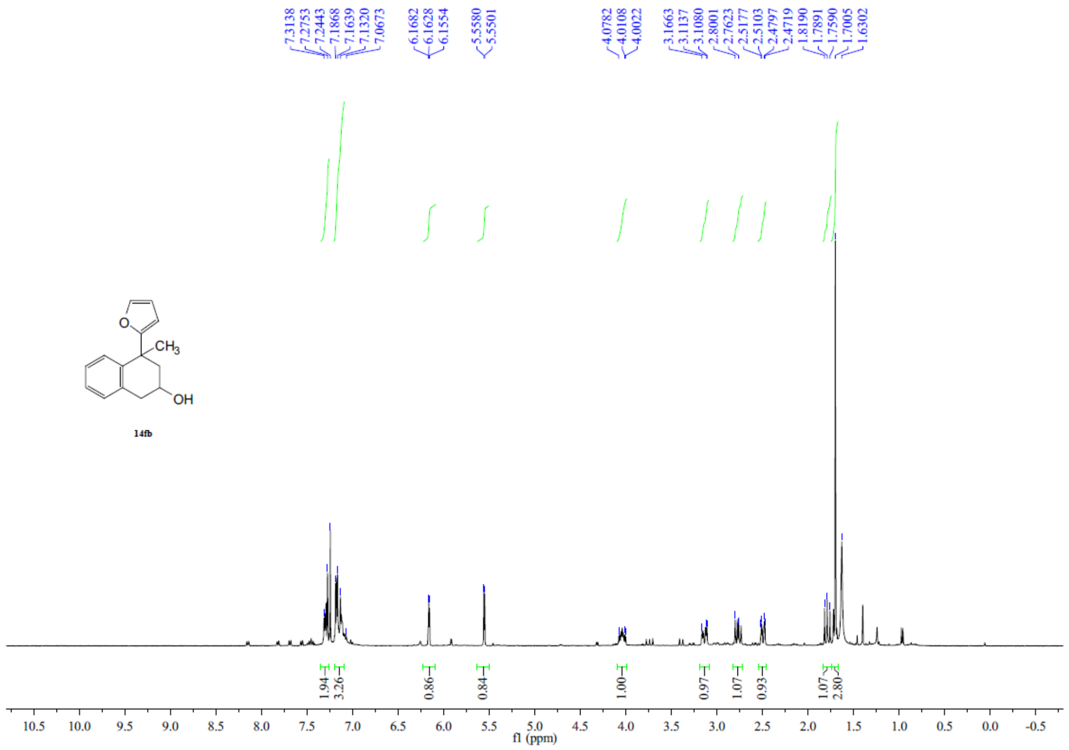

^{13}C NMR ($CDCl_3$, 101 MHz) of 4-(Furan-2-yl)-4-methyl-1,2,3,4-tetrahydronaphthalen-2-ol ***(14fb)***

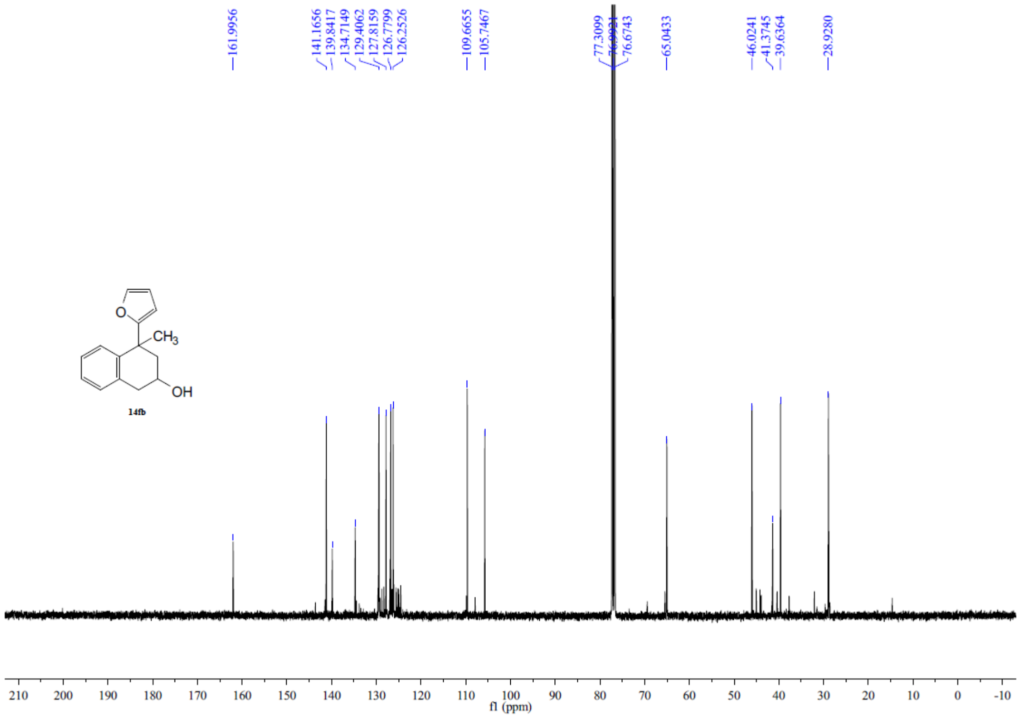

*HSQC of 4-(Furan-2-yl)-4-methyl-1,2,3,4-tetrahydronaphthalen-2-ol **(14fb)***

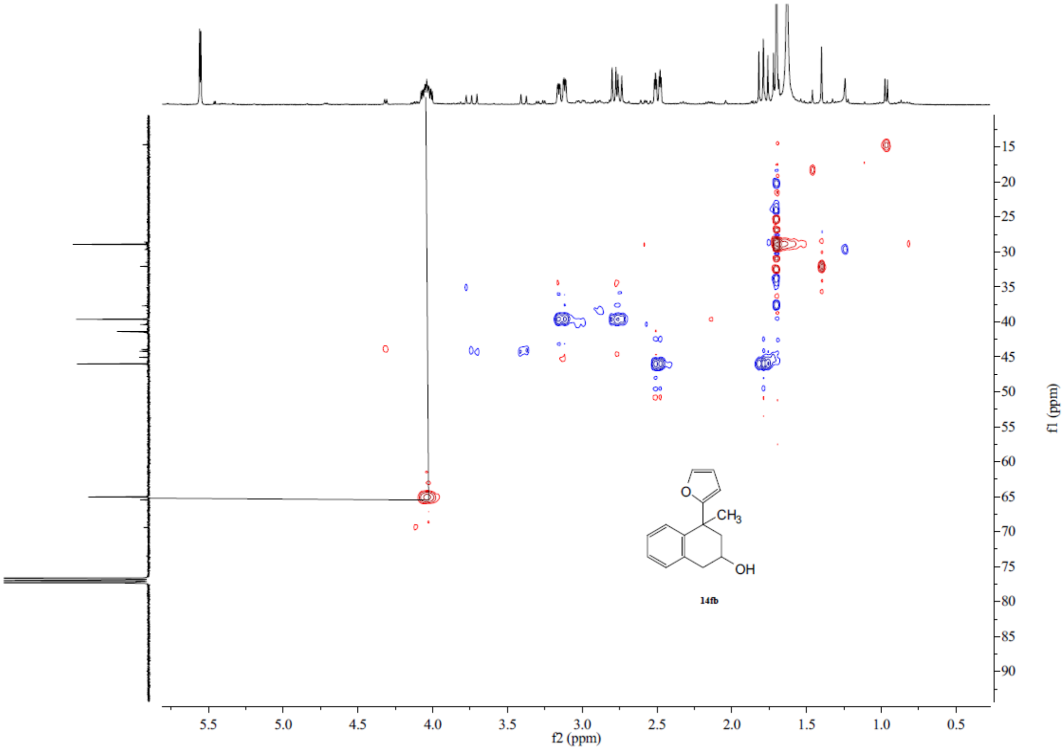

*NOE of 4-(Furan-2-yl)-4-methyl-1,2,3,4-tetrahydronaphthalen-2-ol **(14fb)***

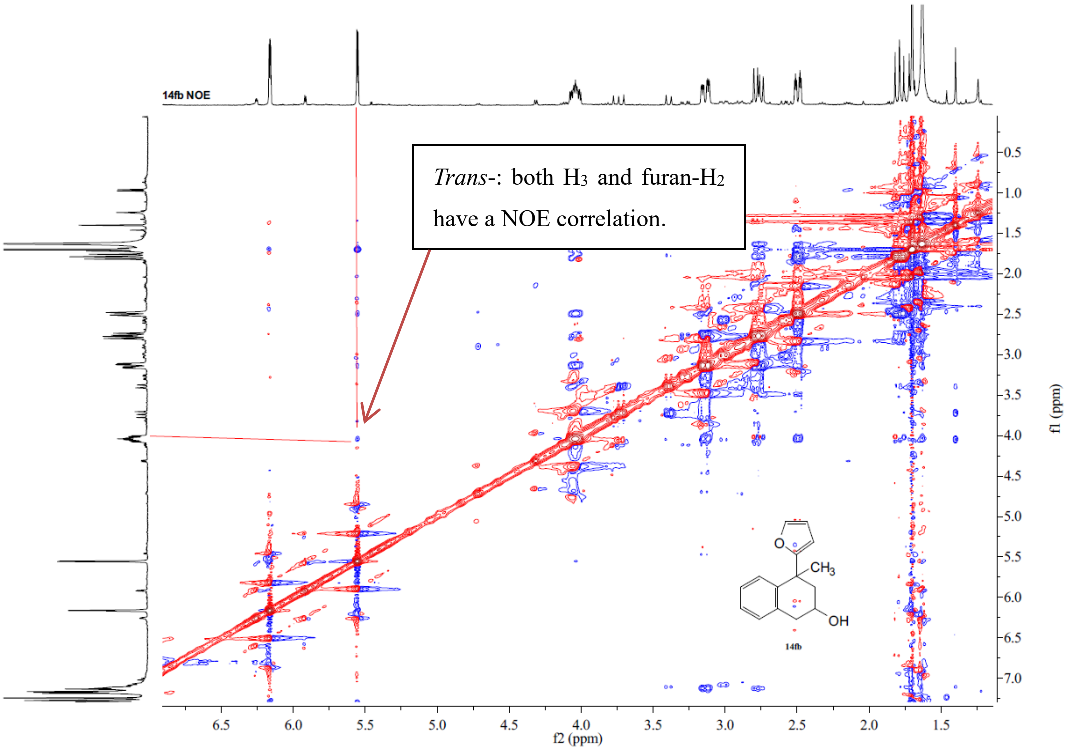

^{1}H NMR ($CDCl_3$, 400 MHz) of 4-(Furan-2-yl)-4-phenyl-1,2,3,4-tetrahydronaphthalen-2-ol **(14ga)**

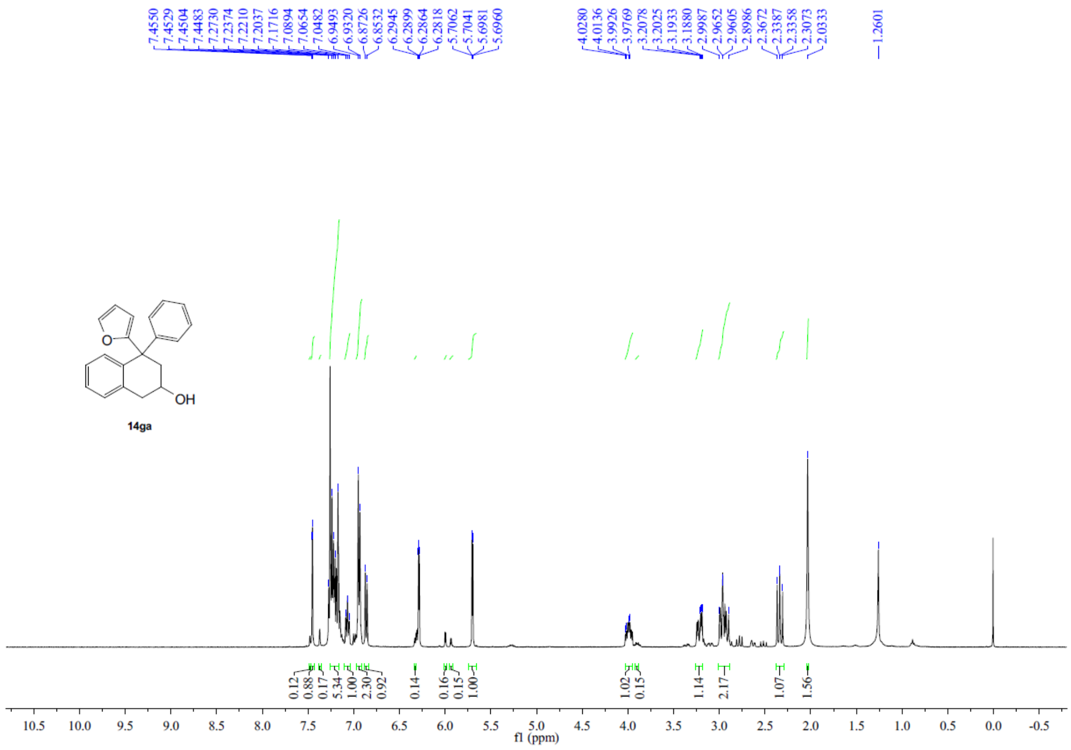

^{13}C NMR ($CDCl_3$, 101 MHz) of 4-(Furan-2-yl)-4-phenyl-1,2,3,4-tetrahydronaphthalen-2-ol **(14ga)**

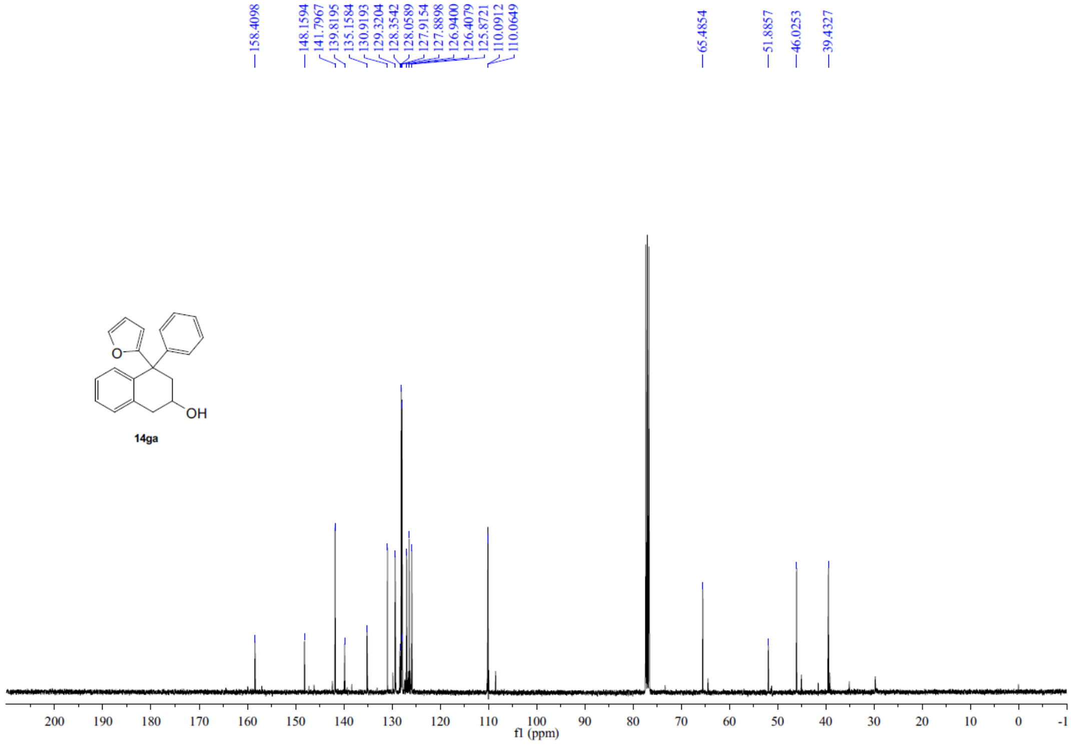

*HSQC of 4-(Furan-2-yl)-4-phenyl-1,2,3,4-tetrahydronaphthalen-2-ol **(14ga)***

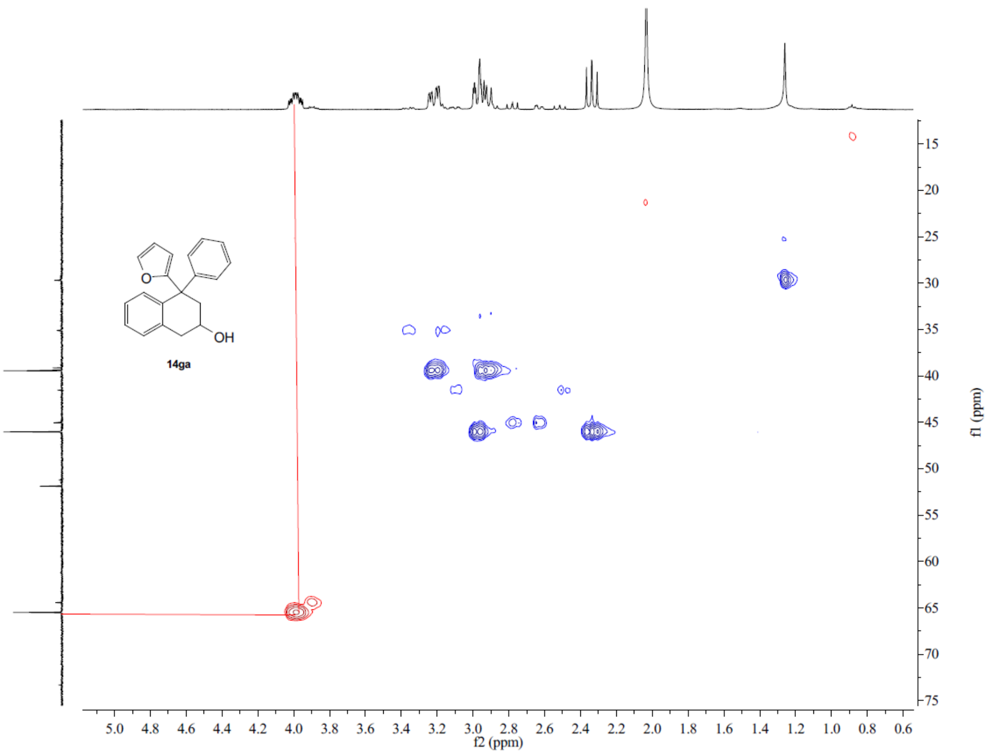

*NOE of 4-(Furan-2-yl)-4-phenyl-1,2,3,4-tetrahydronaphthalen-2-ol **(14ga)***

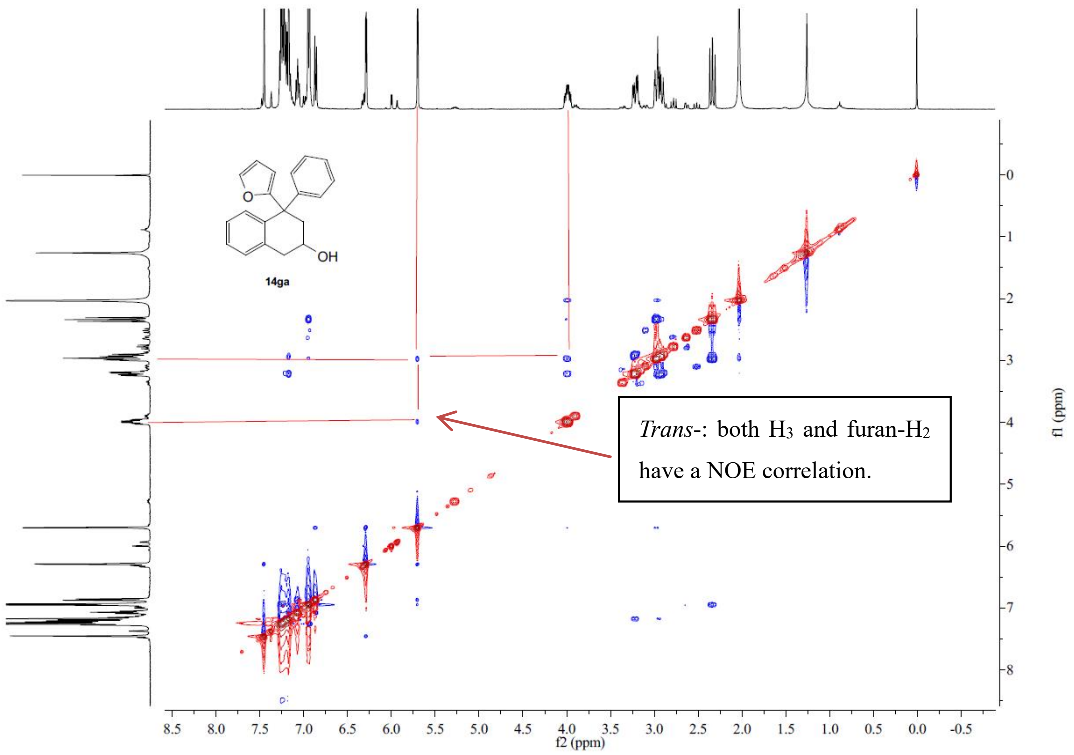

^{1}H NMR (CDCl$_3$, 400 MHz) of 4-(3,4-Dimethoxyphenyl)-1,2,3,4-tetrahydrophenanthren-2-ol ***(14ha)***

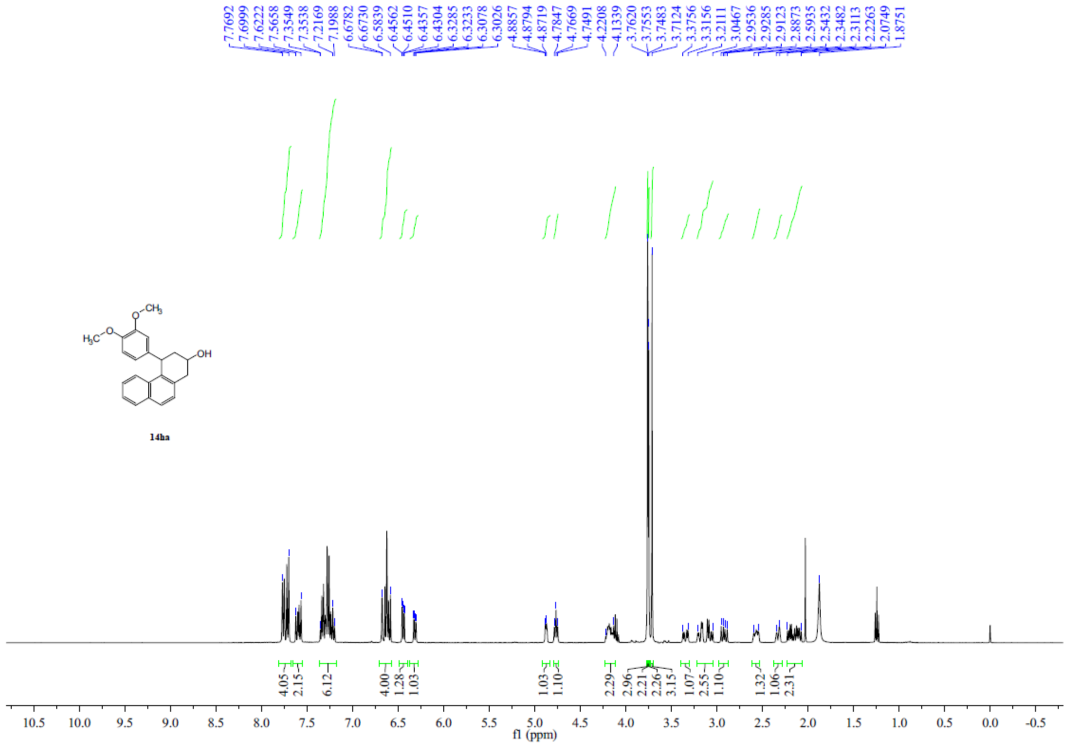

^{13}C NMR (CDCl$_3$, 101 MHz) of 4-(3,4-Dimethoxyphenyl)-1,2,3,4-tetrahydrophenanthren-2-ol ***(14ha)***

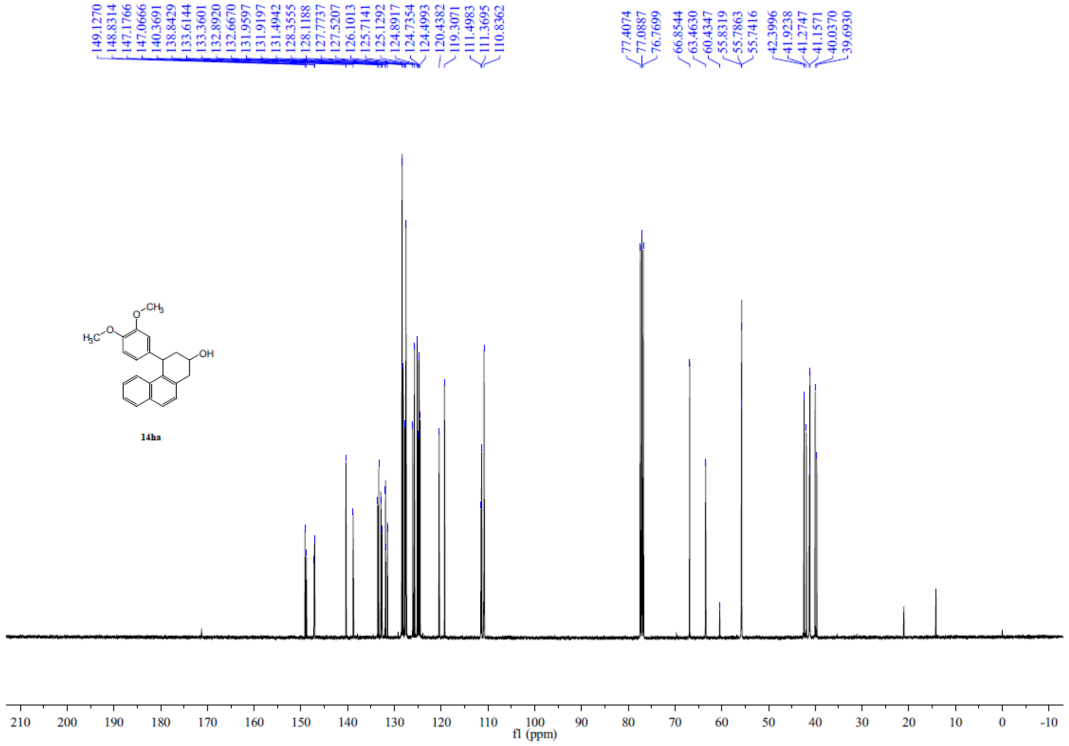

*HSQC of 4-(3,4-Dimethoxyphenyl)-1,2,3,4-tetrahydrophenanthren-2-ol **(14ha)***

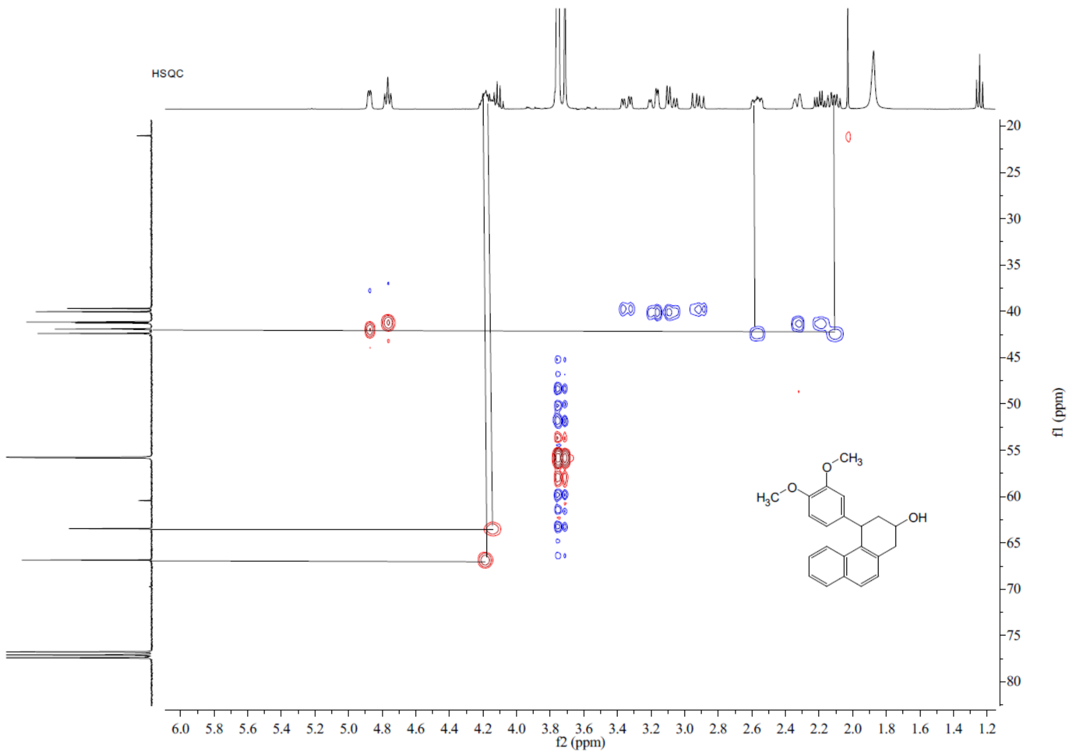

*NOE of 4-(3,4-Dimethoxyphenyl)-1,2,3,4-tetrahydrophenanthren-2-ol **(14ha)***

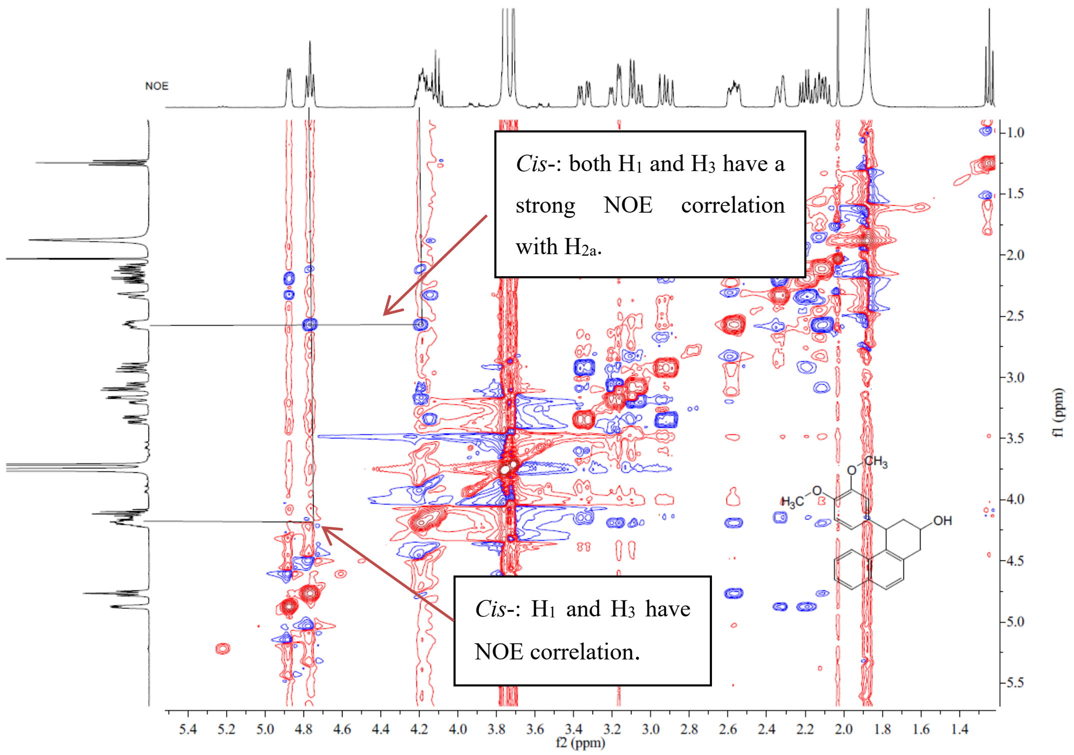

^{1}H NMR ($CDCl_3$, 400 MHz) of 4-(Furan-2-yl)-1,2,3,4-tetrahydrophenanthren-2-ol **(14hb)**

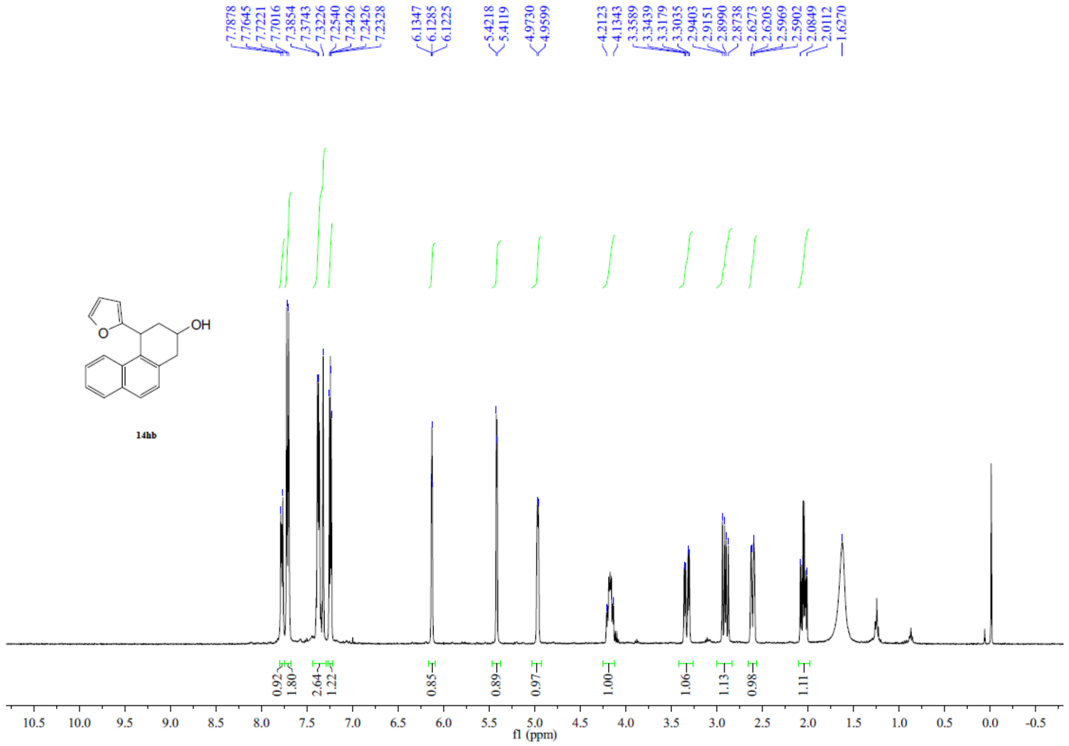

^{13}C NMR ($CDCl_3$, 101 MHz) of 4-(Furan-2-yl)-1,2,3,4-tetrahydrophenanthren-2-ol **(14hb)**

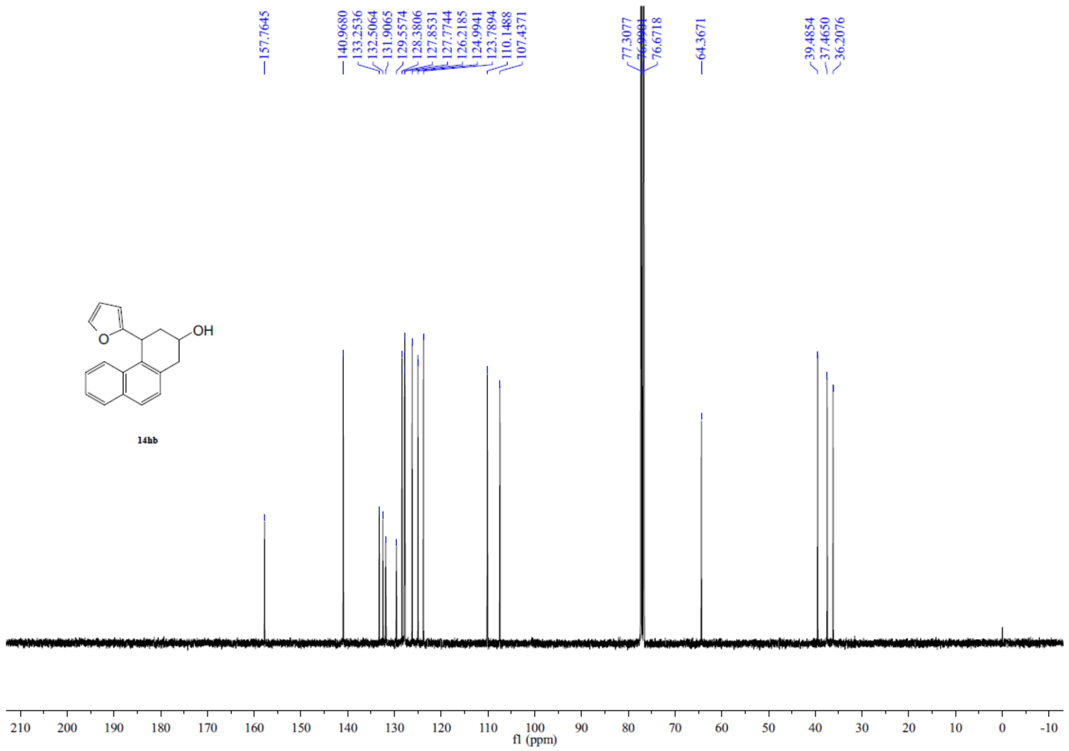

HSQC of 4-(Furan-2-yl)-1,2,3,4-tetrahydrophenanthren-2-ol ***(14hb)***

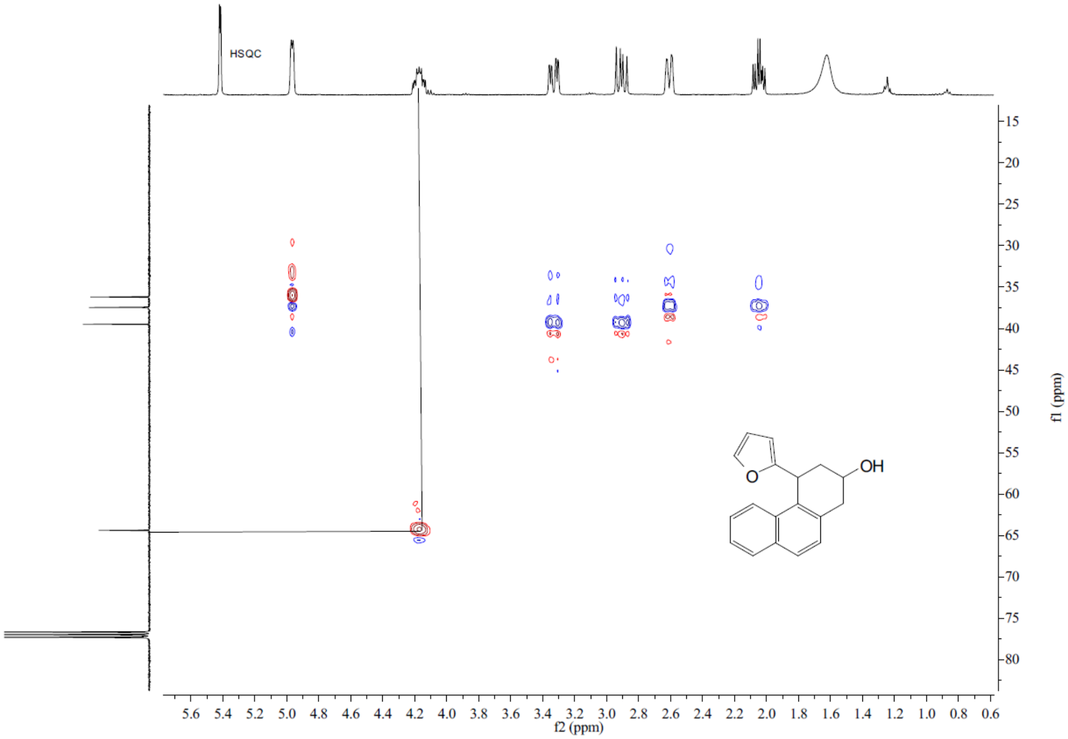

NOE of 4-(Furan-2-yl)-1,2,3,4-tetrahydrophenanthren-2-ol ***(14hb)***

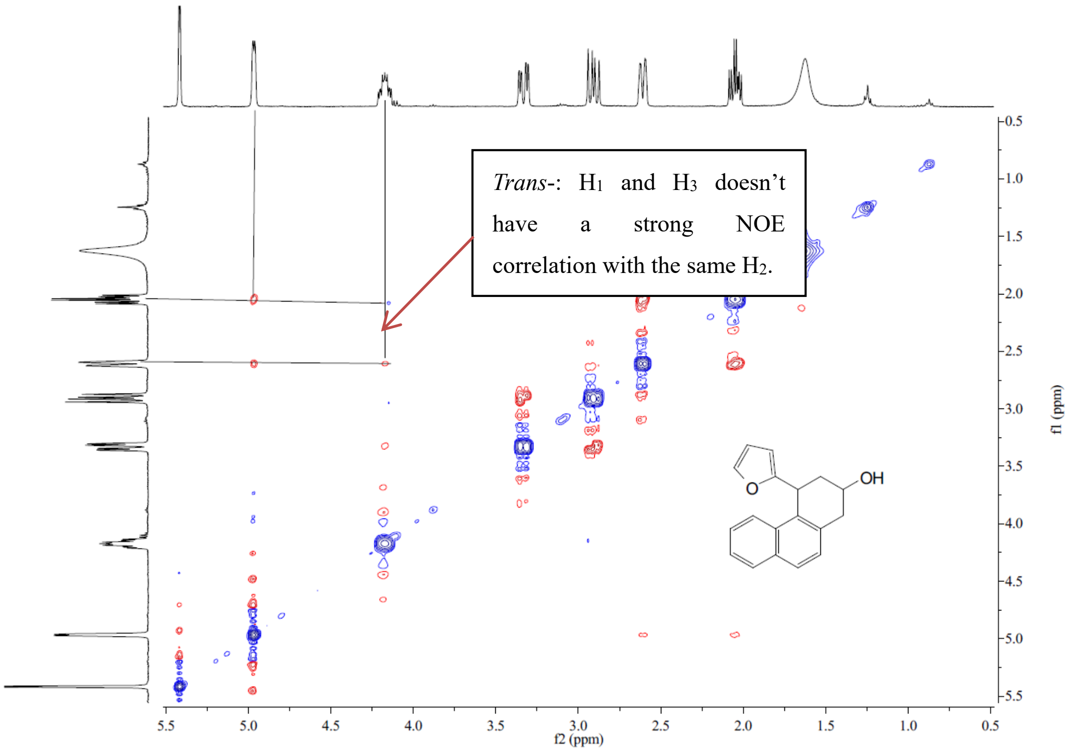

^{1}H NMR ($CDCl_3$, 400 MHz) of 2,2'-(2-(4-Nitro-2-vinylphenyl)ethane-1,1-diyl)difuran **(15)**

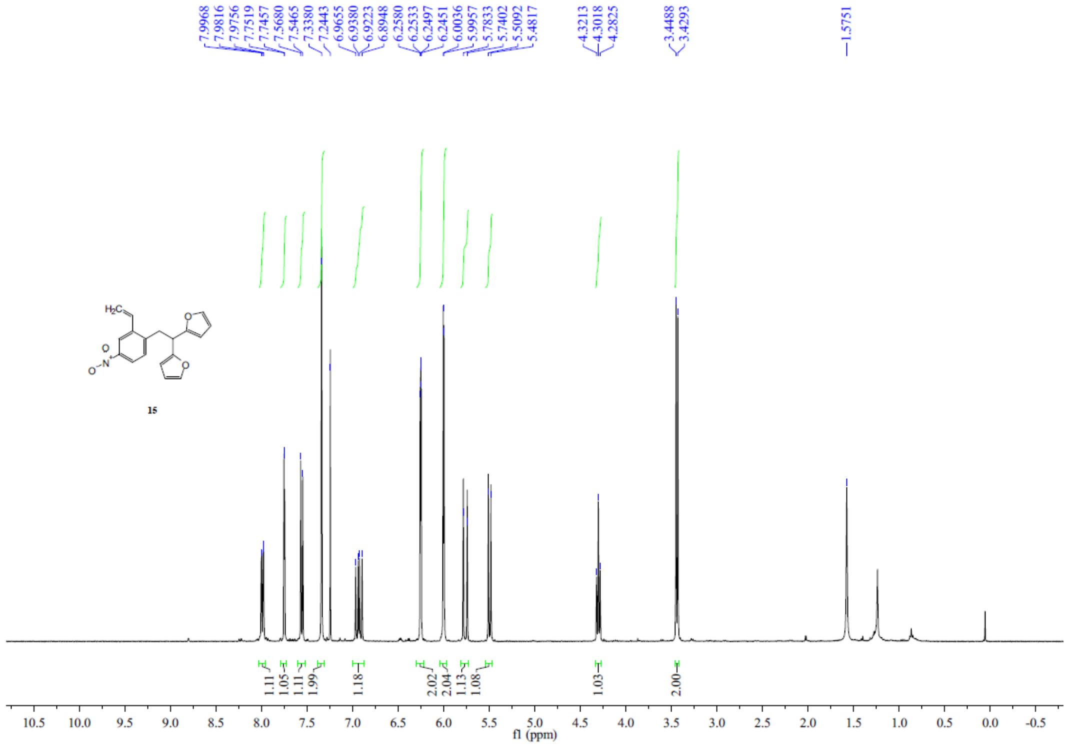

^{13}C NMR ($CDCl_3$, 101 MHz) of 2,2'-(2-(4-Nitro-2-vinylphenyl)ethane-1,1-diyl)difuran **(15)**

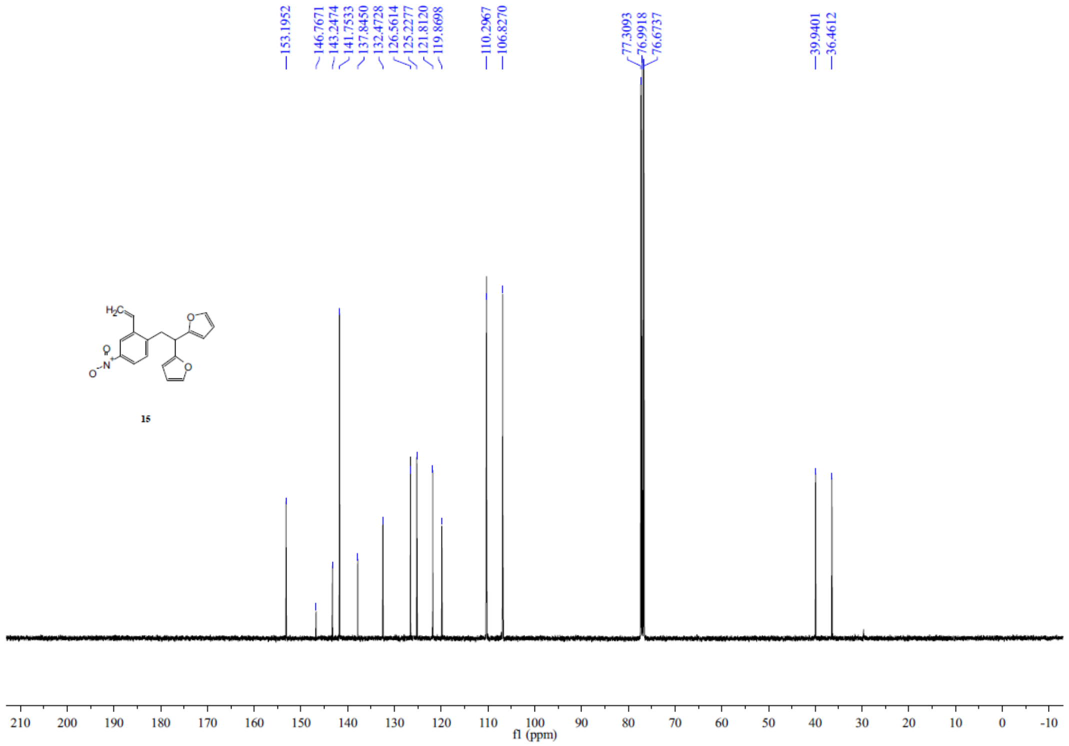

^{1}H NMR ($CDCl_3$, 400 MHz) of 3-(2-Bromophenyl)propan-1-ol ***(17)***

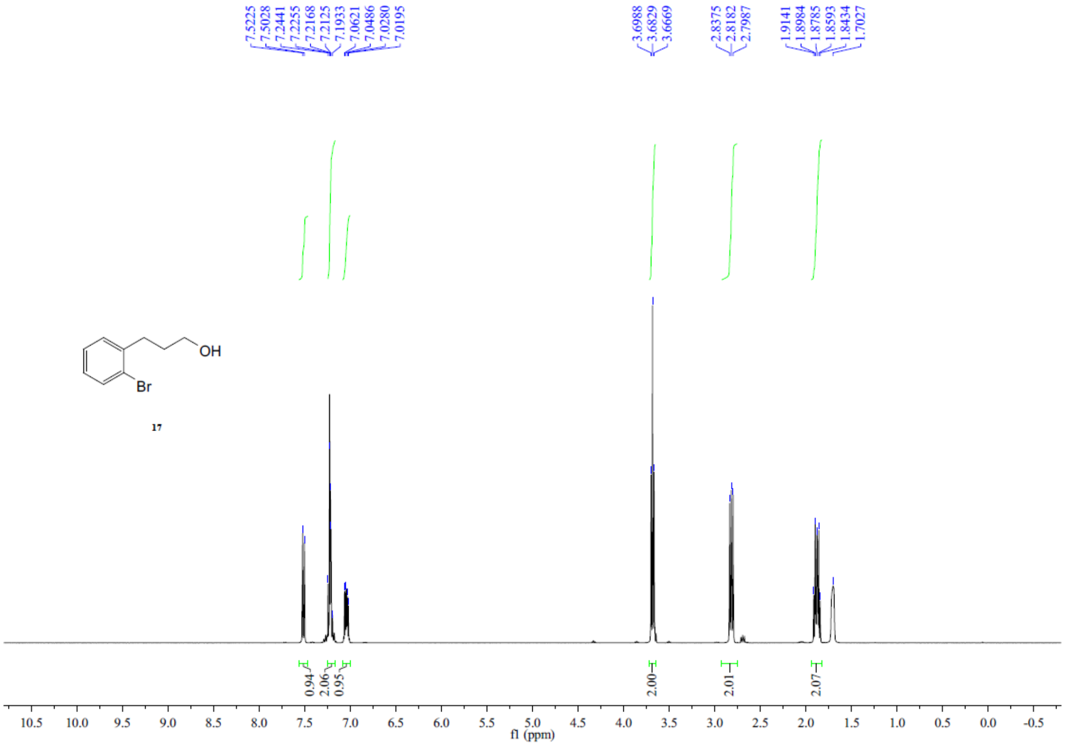

^{13}C NMR ($CDCl_3$, 101 MHz) of 3-(2-Bromophenyl)propan-1-ol ***(17)***

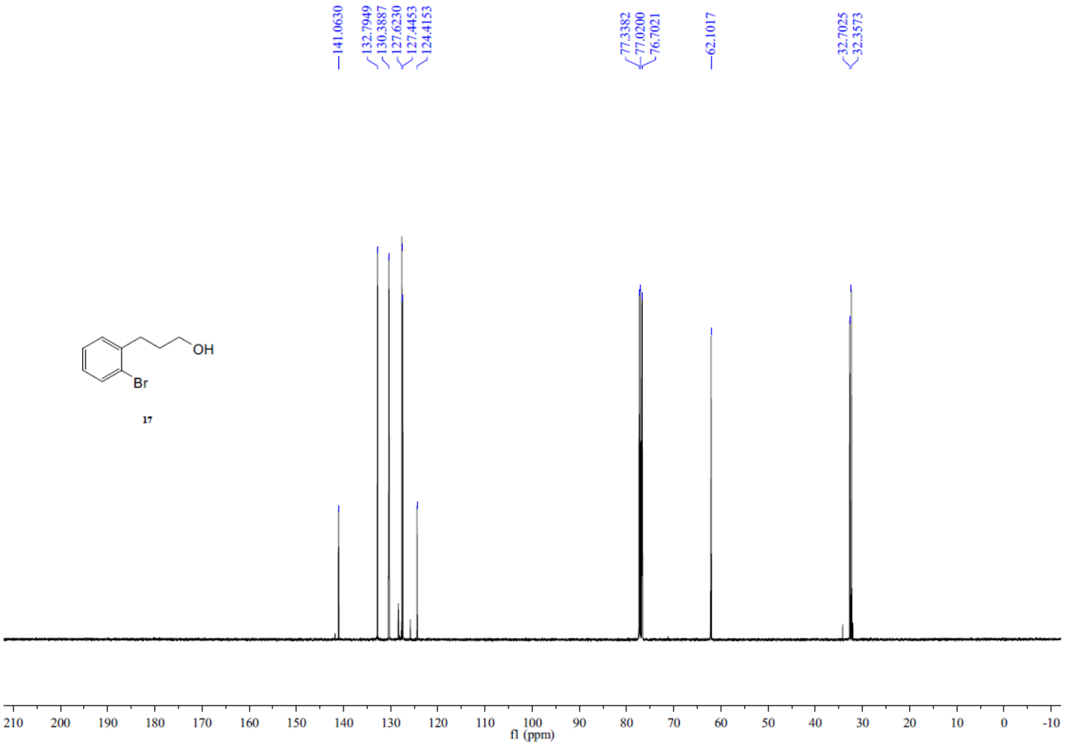

^{1}H NMR ($CDCl_3$, 400 MHz) of 3-(2-Vinylphenyl)propan-1-ol ***(18)***

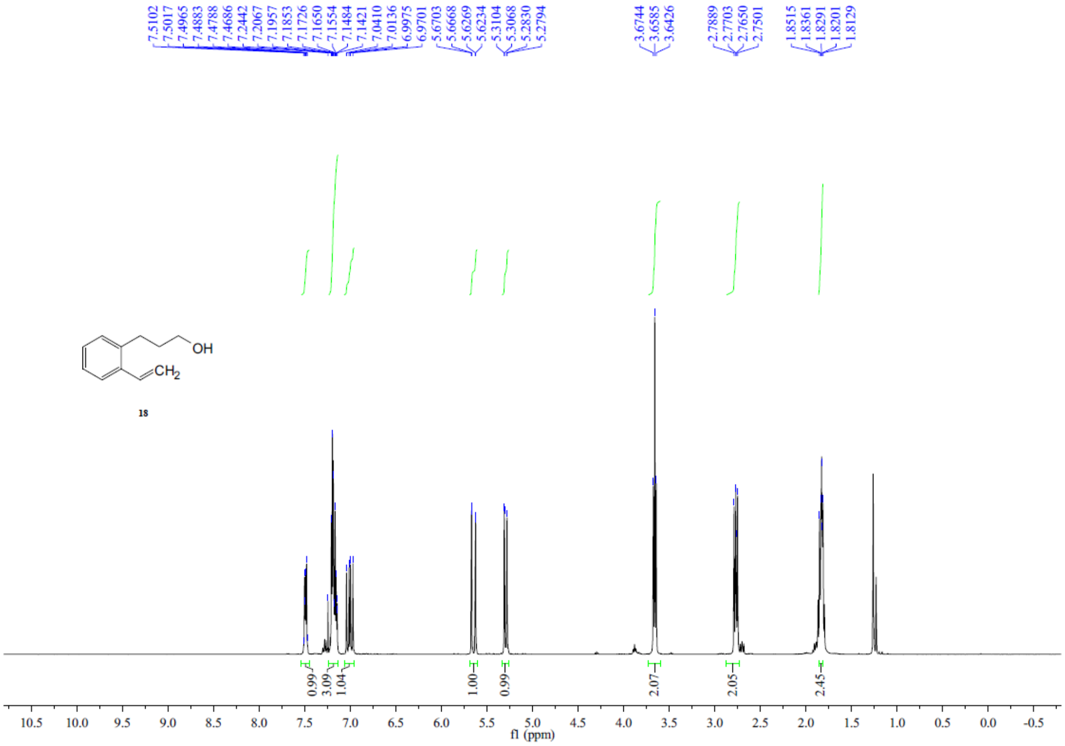

^{13}C NMR ($CDCl_3$, 101 MHz) of 3-(2-Vinylphenyl)propan-1-ol ***(18)***

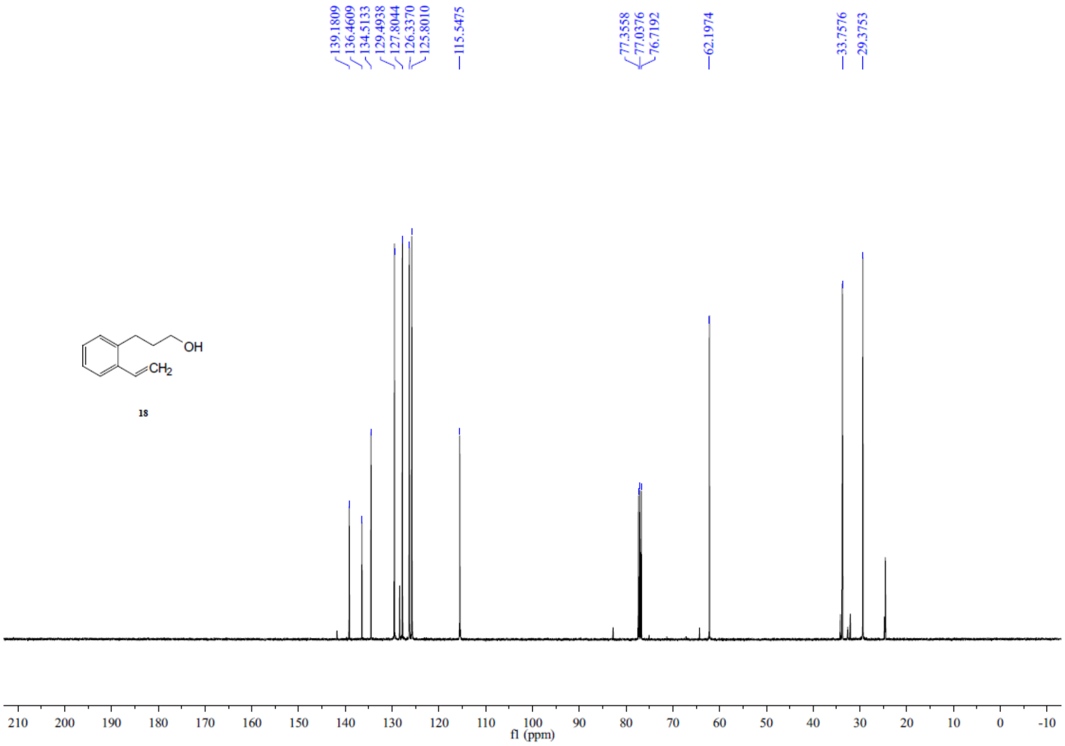

^{1}H NMR ($CDCl_3$, 400 MHz) of 3-(2-Vinylphenyl)propanal ***(19)***

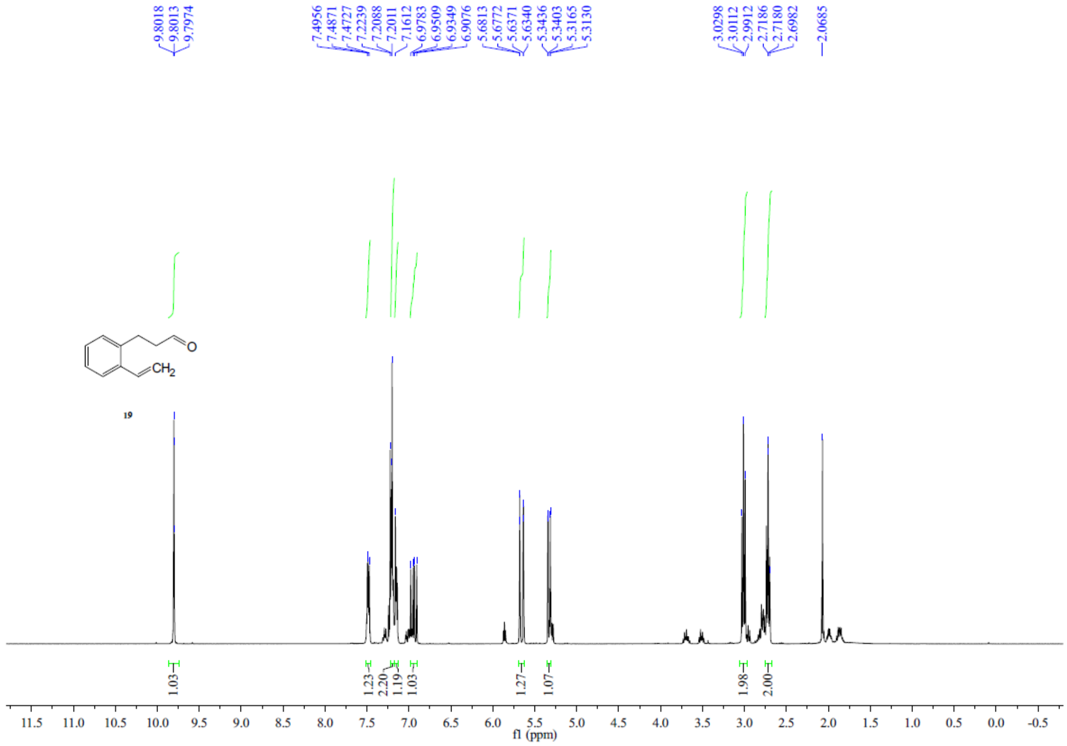

^{13}C NMR ($CDCl_3$, 101 MHz) of 3-(2-Vinylphenyl)propanal ***(19)***

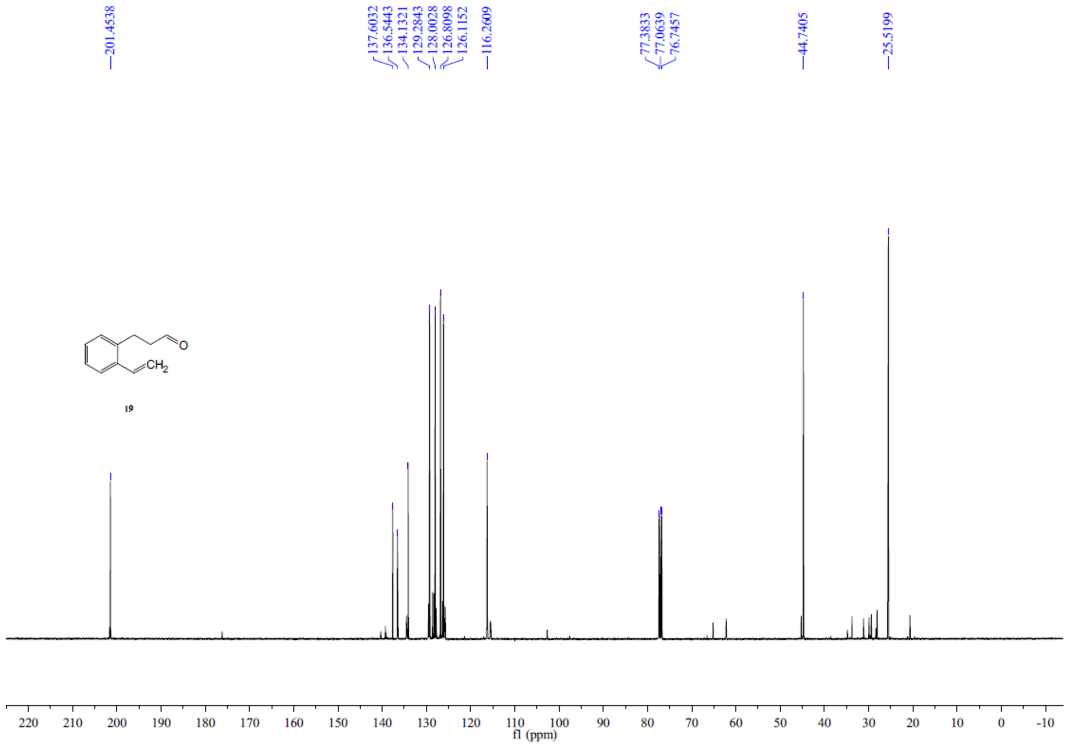

^{1}H NMR ($CDCl_3$, 400 MHz) of 5-(3,4-Dimethoxyphenyl)-6,7,8,9-tetrahydro-5H-benzo[7]annulen-7-ol ***(20a)***

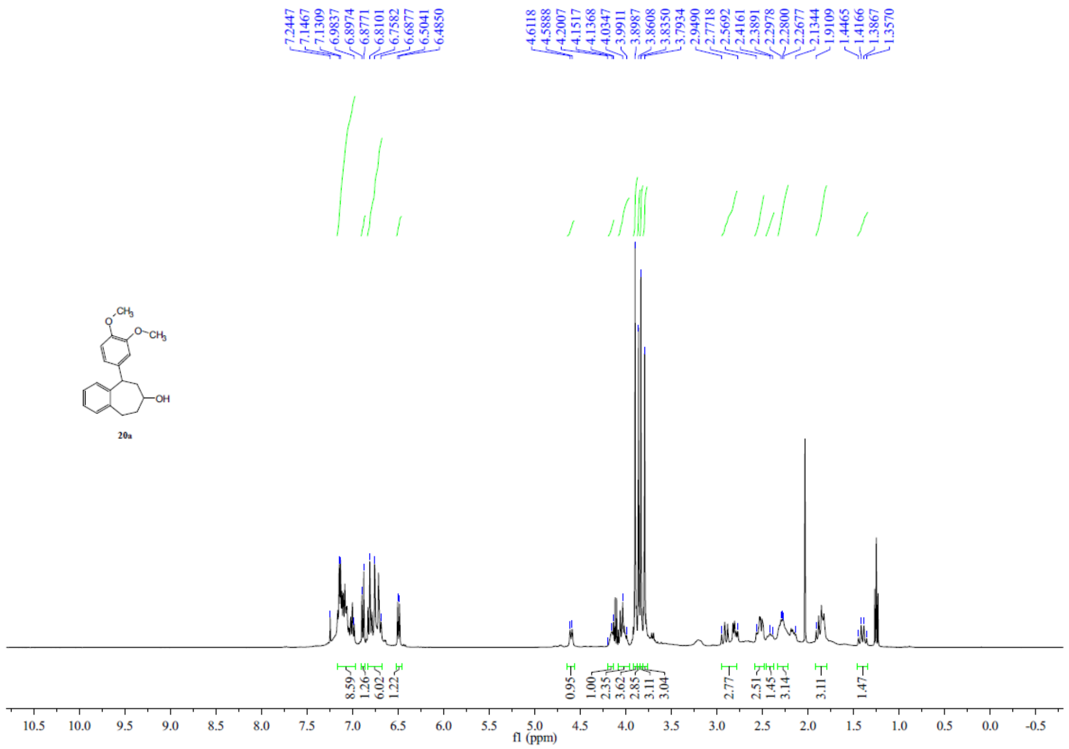

^{13}C NMR ($CDCl_3$, 101 MHz) of 5-(3,4-Dimethoxyphenyl)-6,7,8,9-tetrahydro-5H-benzo[7]annulen-7-ol ***(20a)***

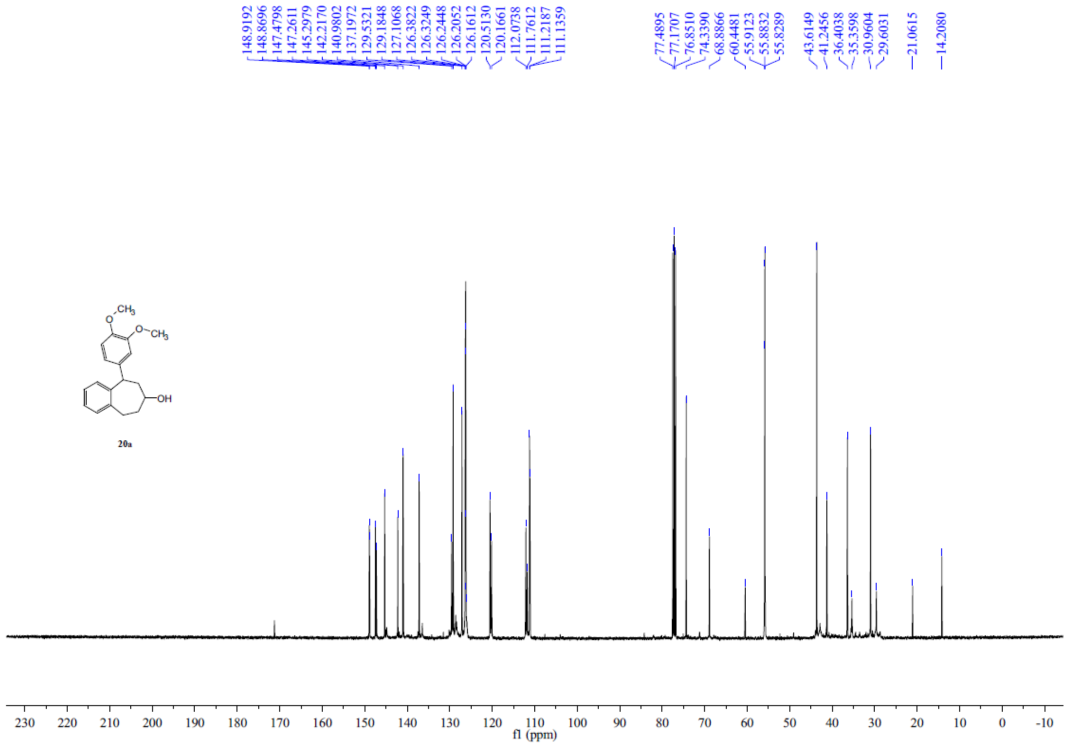

HSQC of 5-(3,4-Dimethoxyphenyl)-6,7,8,9-tetrahydro-5H-benzo[7]annulen-7-ol ***(20a)***

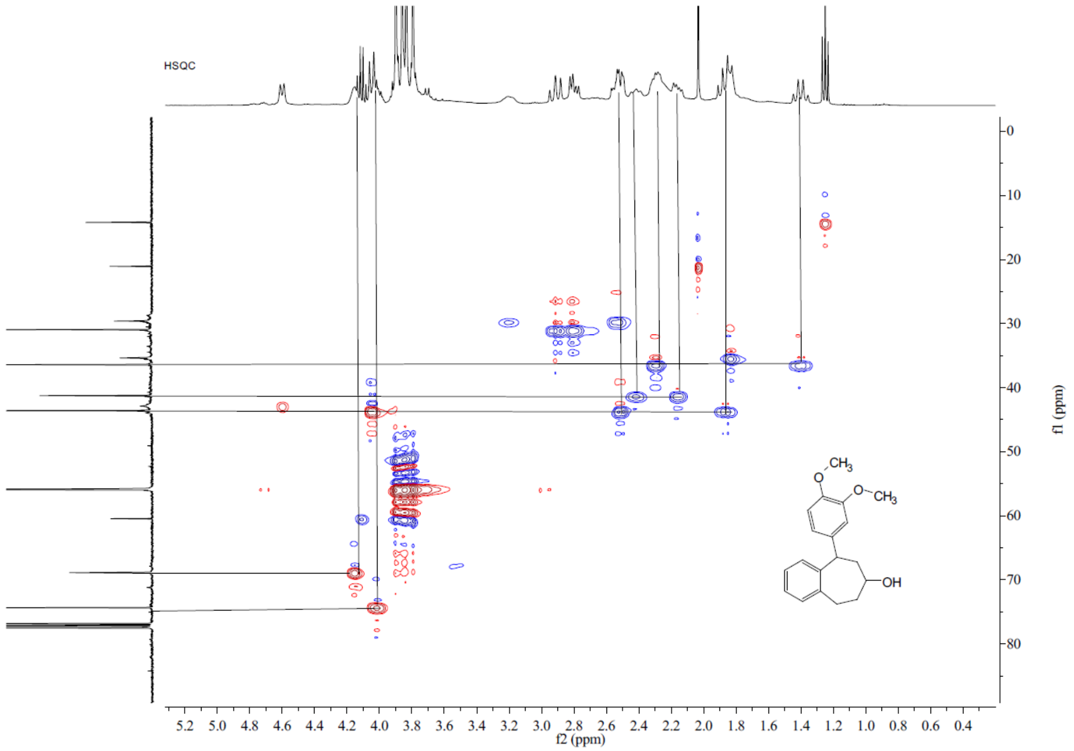

NOE of 5-(3,4-Dimethoxyphenyl)-6,7,8,9-tetrahydro-5H-benzo[7]annulen-7-ol ***(20a)***

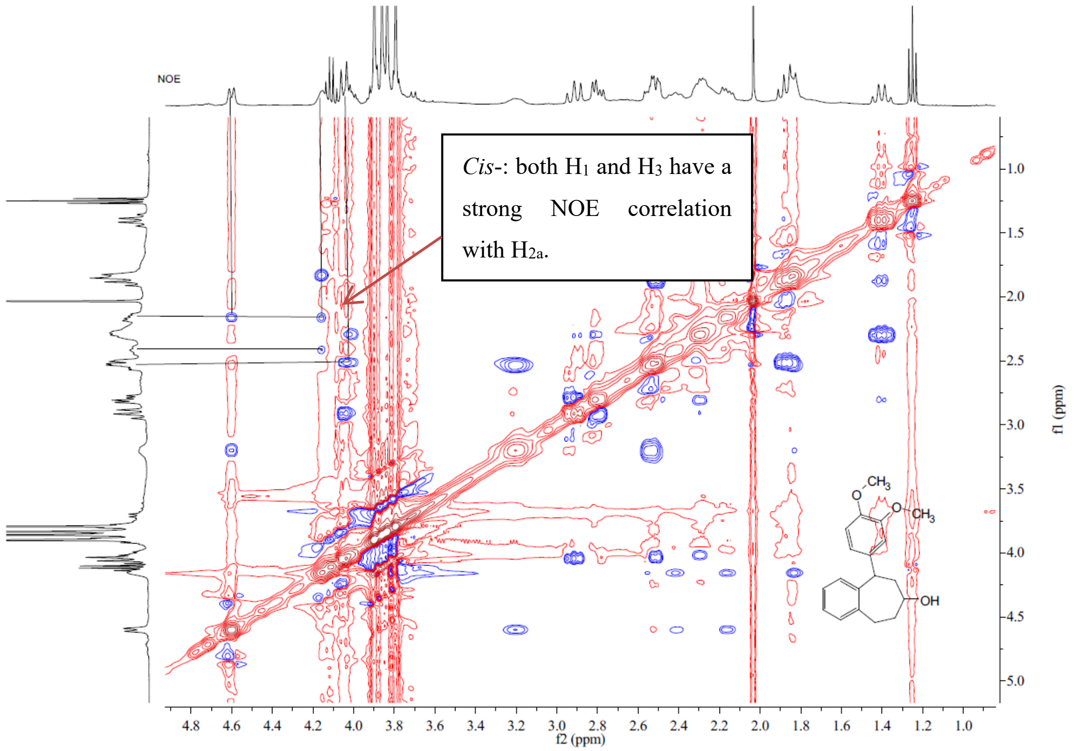

^{1}H NMR ($CDCl_3$, 400 MHz) of 5-(Furan-2-yl)-6,7,8,9-tetrahydro-5H-benzo[7]annulen-7-ol ***(20b)***

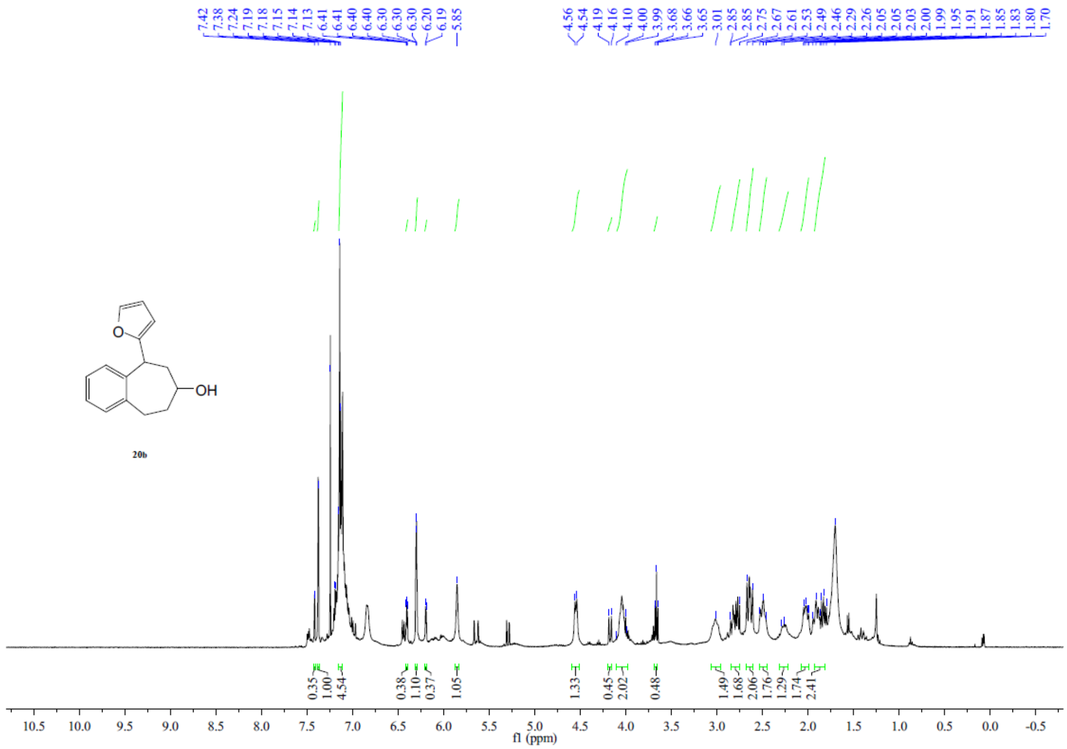

^{13}C NMR ($CDCl_3$, 101 MHz)of 5-(Furan-2-yl)-6,7,8,9-tetrahydro-5H-benzo[7]annulen-7-ol ***(20b)***

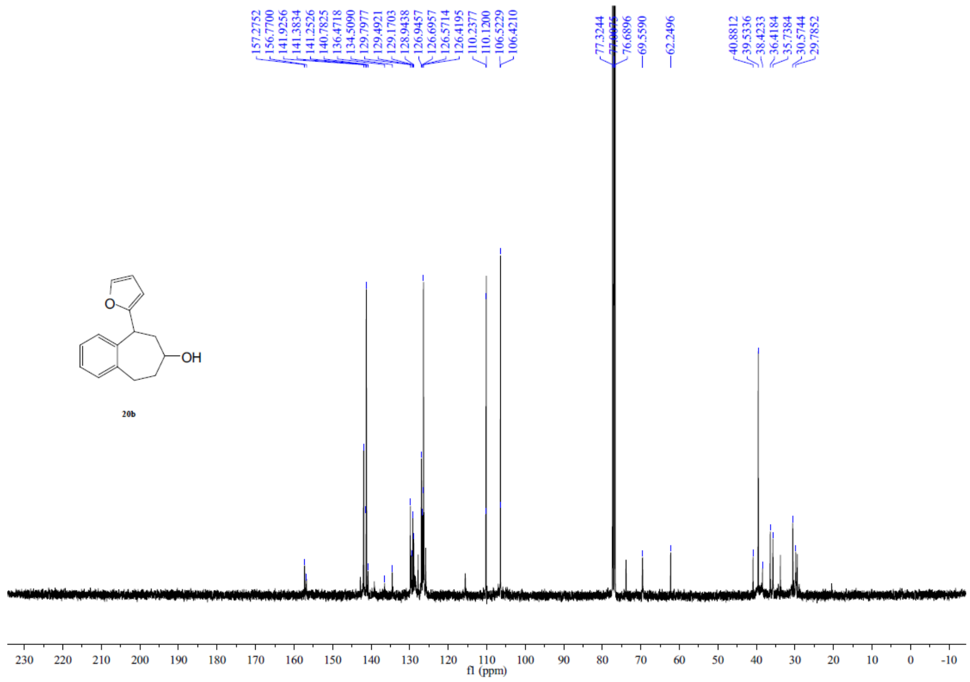

*HSQC of 5-(Furan-2-yl)-6,7,8,9-tetrahydro-5H-benzo[7]annulen-7-ol **(20b)***

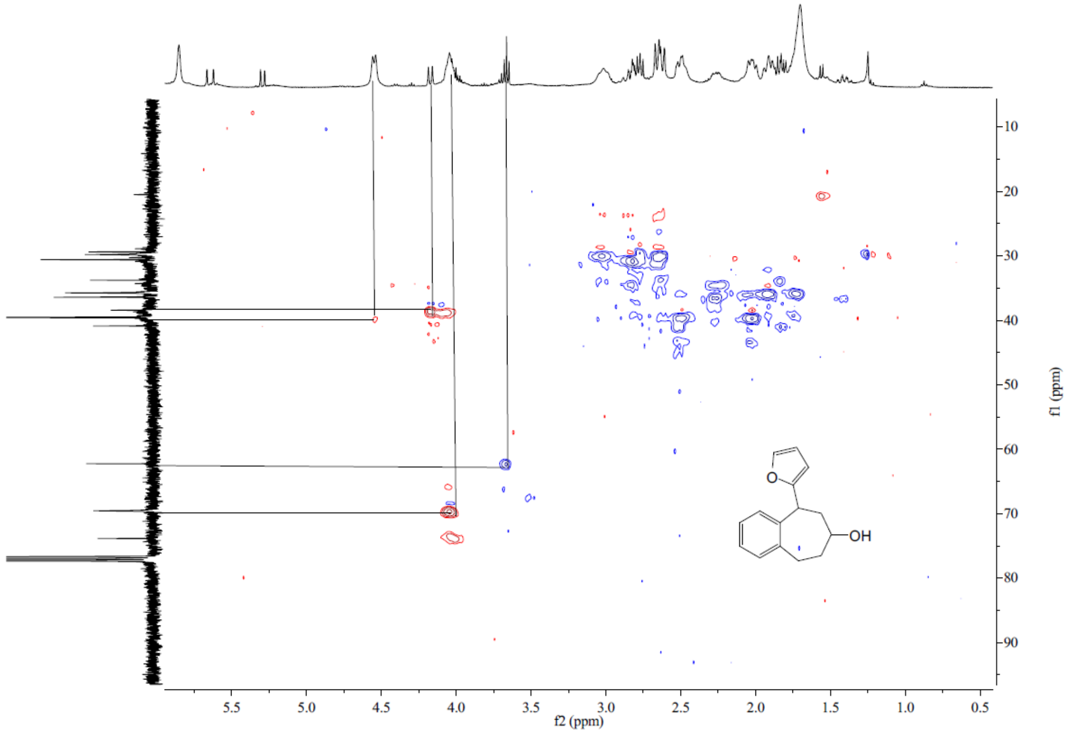

*NOE of 5-(Furan-2-yl)-6,7,8,9-tetrahydro-5H-benzo[7]annulen-7-ol **(20b)***

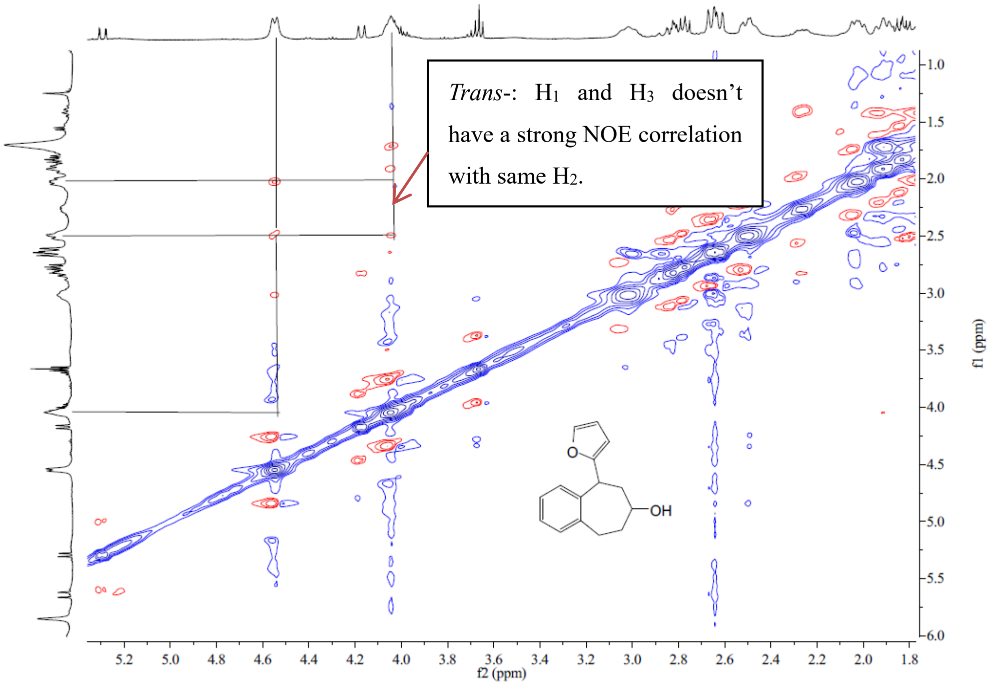

^{1}H NMR ($CDCl_3$, 400 MHz) of 4-(Furan-2-yl)-1,2,3,4-tetrahydronaphthalen-2-yl-4-methylbenzenesulfonate ***(21)***

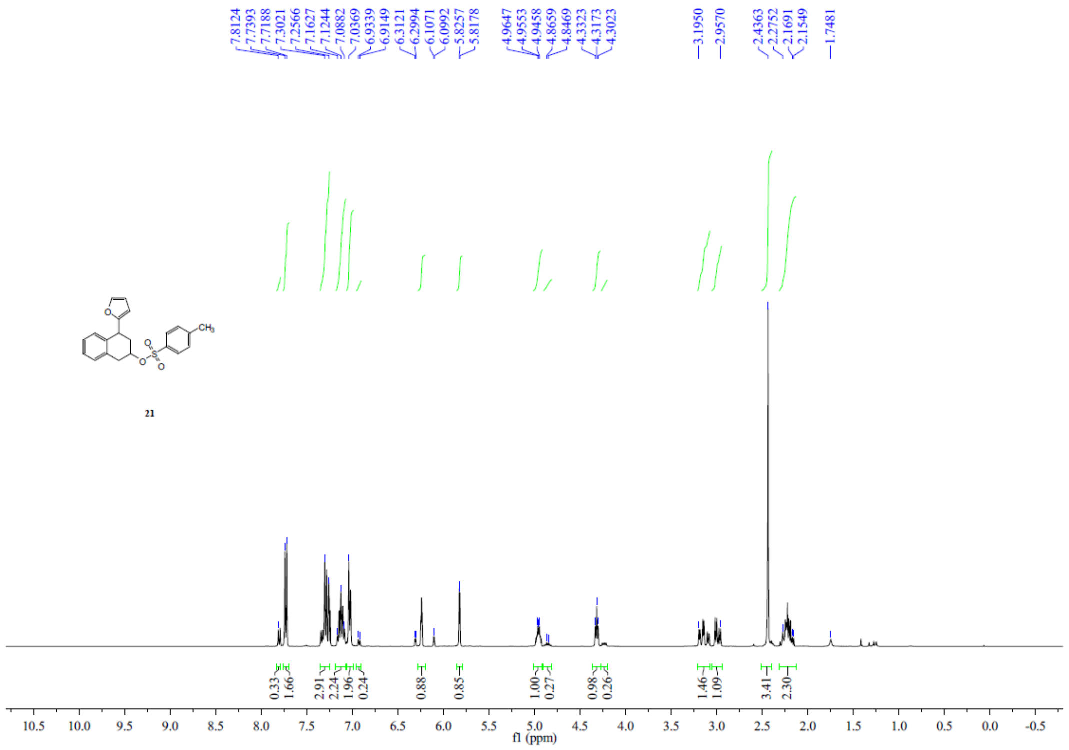

^{13}C NMR ($CDCl_3$, 101 MHz) of 4-(Furan-2-yl)-1,2,3,4-tetrahydronaphthalen-2-yl-4-methylbenzenesulfonate ***(21)***

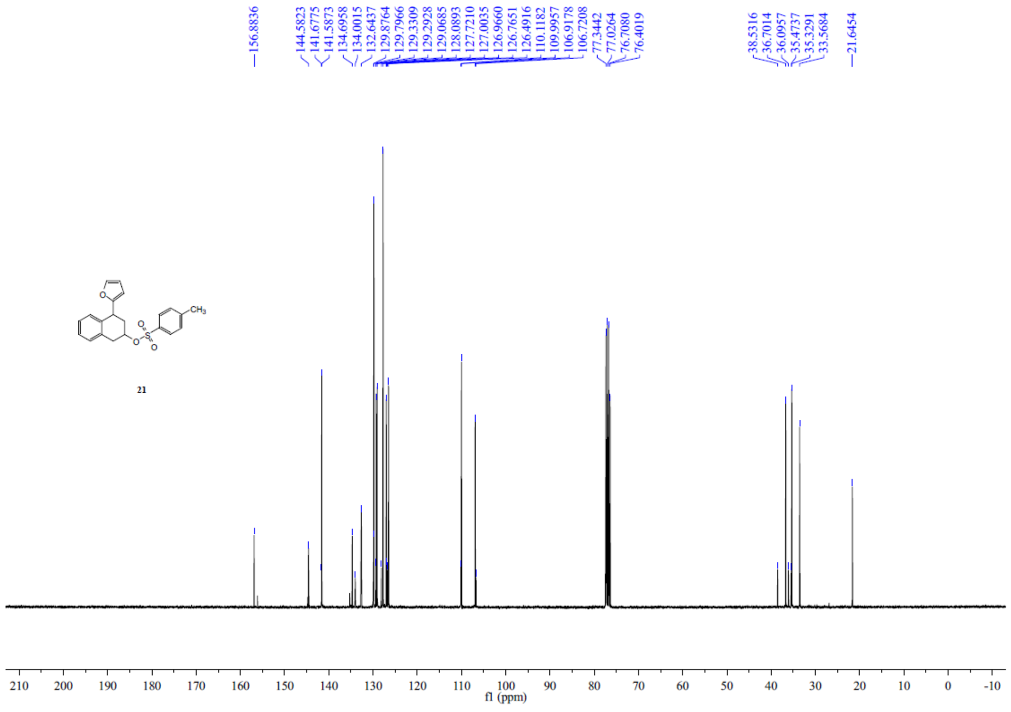

*HSQC of 4-(Furan-2-yl)-1,2,3,4-tetrahydronaphthalen-2-yl-4-methylbenzenesulfonate **(21)***

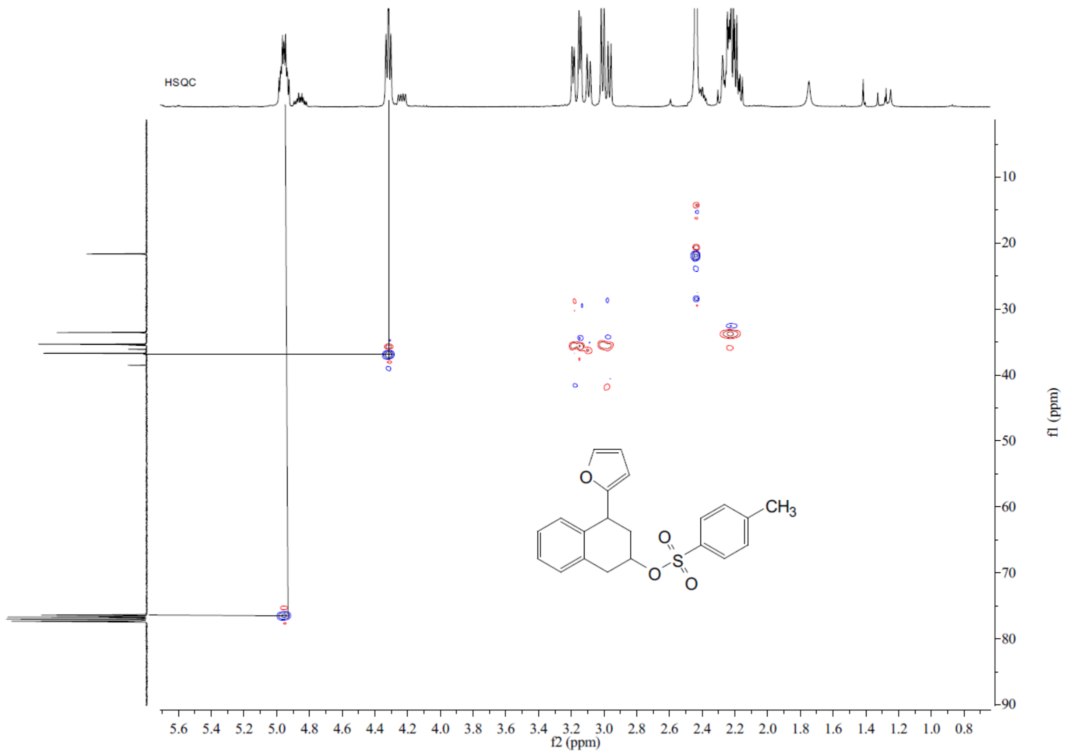

*NOE of 4-(Furan-2-yl)-1,2,3,4-tetrahydronaphthalen-2-yl-4-methylbenzenesulfonate **(21)***

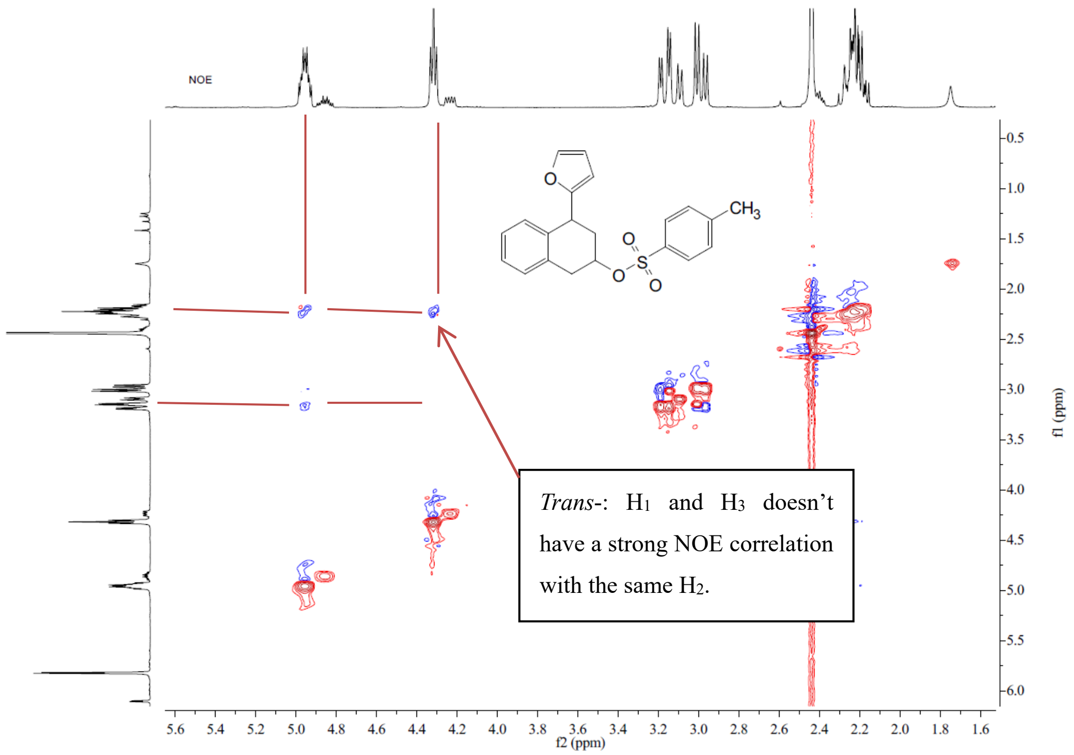

^{1}H NMR ($CDCl_3$, 400 MHz) of *4-(Furan-2-yl)-N,N-dimethyl-1,2,3,4-tetrahydronaphthalen-2-amine hydrochloride* ***(22)***

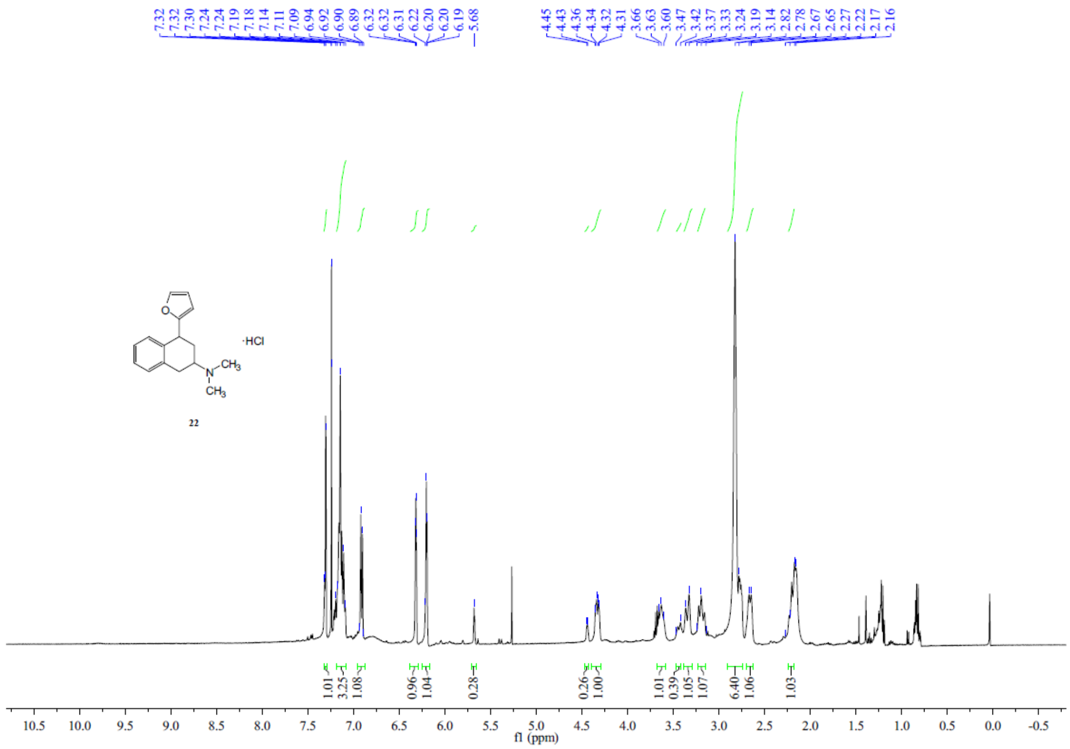

^{13}C NMR ($CDCl_3$, 101 MHz) of *4-(Furan-2-yl)-N,N-dimethyl-1,2,3,4-tetrahydronaphthalen-2-amine hydrochloride* ***(22)***

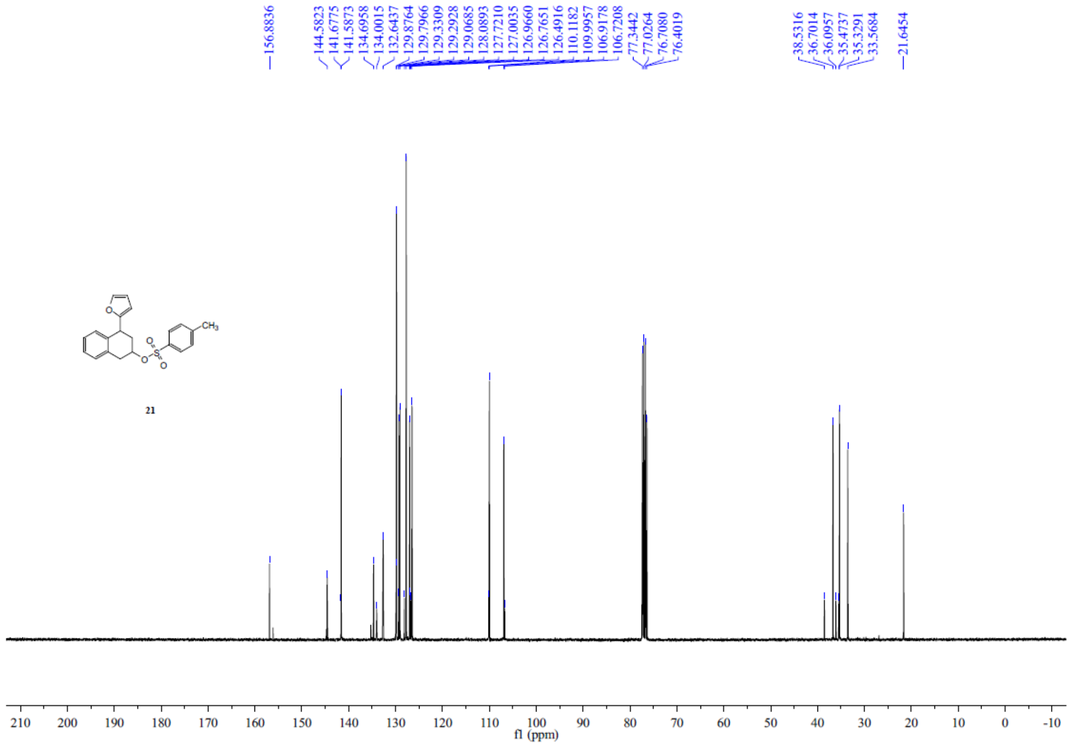

7. X-ray Crystal for compound 21

(Anisotropic displacement parameters for ellipsoid contours are shown at 50% probability for all atoms except H).

The crystallographic data have been deposited with the Cambridge Crystallographic Data Centre (CCDC) as CCDC-2060394. CCDC information can be obtained free of charge from www.ccdc.cam.ac.uk. The detailed crystallographic data are summarized in Table S4 and Table S5.

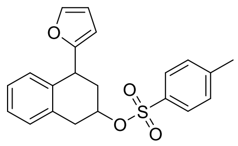

21

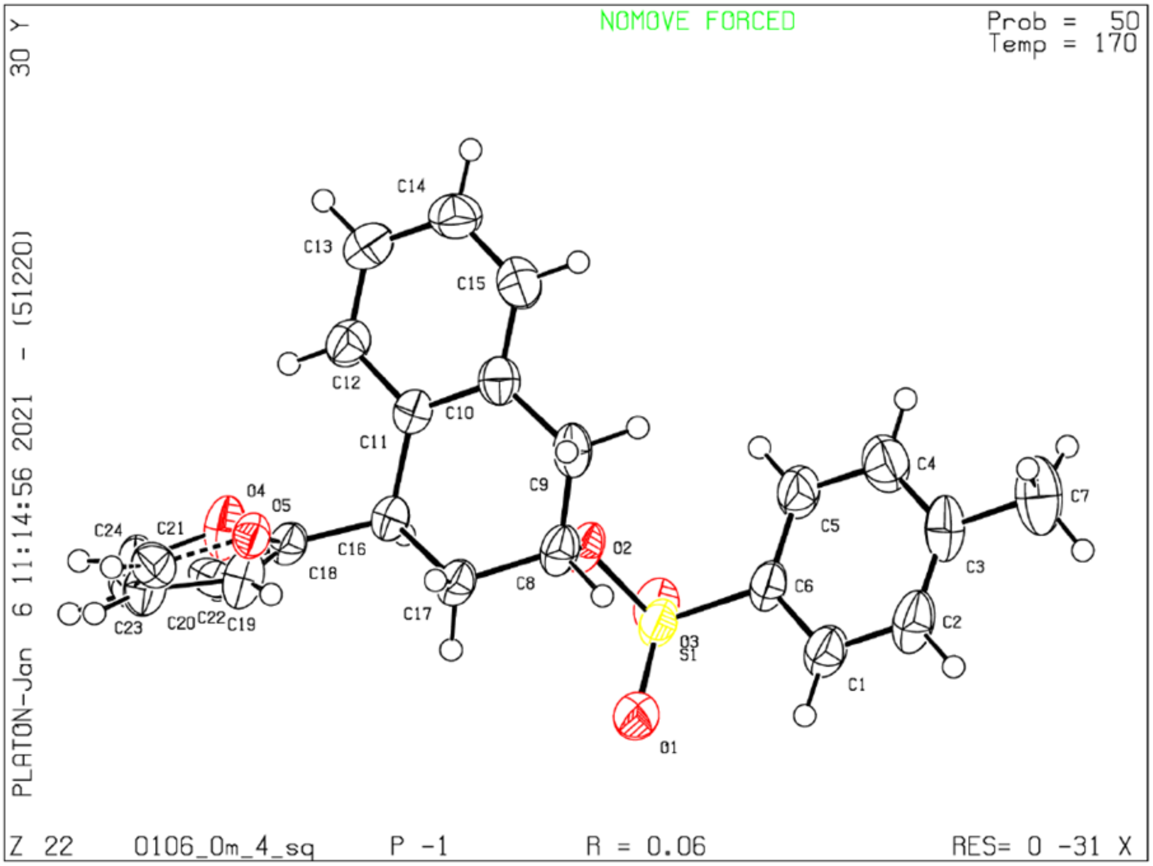

**Table S4:** Crystal data and structure refinement for **21.**	
Identification code	21
Empirical formula	$C_{21}H_{20}O_4S$
Formula weight	368.43
Temperature	170 K

Wavelength	0.71073 Å
Crystal system	triclinic
Space group	P-1
Unit cell dimension	a = 7.8180(6) Å<br>b = 11.0040(9) Å<br>c = 14.0257(12) Å<br>α = 106.509(3)°<br>β = 100.628(2)°<br>γ = 104.333(3)°
Volume	1077.88(15) $Å^3$
Z	2
Density (calculated)	1.135 Mg/m^3
Absorption coefficient	0.170 mm^{-1}
F(000)	388.0
Crystal size	0.15 × 0.08 × 0.05 mm^3
Radiation Theta range for data collection	4.198 to 52.826°
Index ranges	-26 ≤ h ≤ 13<br>-14 ≤ k ≤ 14<br>-22 ≤ l ≤ 23
Reflections collected	4285
Independent reflections	4285 [Rsigma = 0.0534]
Goodness-of-fit on F^2	1.086
Final R indexes [I>=2σ (I)]	R_1 = 0.0574, wR_2 = 0.1352
Final R indexes [all data]	R_1 = 0.0882, wR_2 = 0.1516
Data/restraints/parameters	4285/377/263
Largest diff. peak/hole	0.29/-0.28 e. $Å^{-3}$

Table S5: Data block: **21**

Bond precision:	C-C = 0.0040 A Wavelength=0.71073
Cell:	a=7.8180(6) b=11.0040(9) c=14.0257(12)<br>alpha=106.509(3) beta=100.628(2) gamma=104.333(3)
Temperature:	170 K
Calculated Reported	
Volume	1077.89(16) 1077.88(15)
Space group	P -1 P -1
Hall group	-P 1 -P 1
Moiety formula	C21 H20 O4 S [+ solvent] C21 H20 O4 S
Sum formula	C21 H20 O4 S [+ solvent] C21 H20 O4 S
Mr	368.43 368.43
Dx,g cm-3	1.135 1.135
Z	2 2

Mu (mm^{-1})	0.170	0.170
F000	388.0	388.0
F000’	388.43	
h,k,lmax	9,13,17	0,0,0
Nref	4419	4285
Tmin,Tmax	0.984,0.992	0.668,0.745
Tmin’	0.975	
Correction method=	MULTI-SCAN	

Data completeness= 0.970 Theta(max)= 26.413

R(reflections)= 0.0574 (2988) wR2(reflections)= 0.1516 (4285)

S = 1.086 Npar= 263

8. References

1. Atienza, B. J. P.; Truong, N.; Williams, F. J. *Org. Lett.* **2018**, *20*, 6332–6335. doi:10.1021/acs.orglett.8b02356
2. Yang, X.-Y.; Lin, H.-S.; Matsuo, Y. *J. Org.Chem.* **2019**, *84*, 16314–16322. doi:10.1021/acs.joc.9b02618
3. Dai, X.-J.; Engl, O. D.; León, T.; Buchwald, S. L. *Angew. Chem. Int. Ed.* **2019**, *58*, 3407–3411. doi:10.1002/anie.201814331
4. Kita, Y.; Yata, T.; Nishimoto, Y.; Yasuda, M. *J. Org. Chem.* **2018**, *83*, 740–753. doi:10.1021/acs.joc.7b02739
5. Fustero, S.; Rodríguez, E.; Lázaro, R.; Herrera, L.; Catalán, S.; Barrio, P. *Adv. Synth. Catal.* **2013**, *355*, 1058–1064. doi:10.1002/adsc.201201095
6. Rabasa-Alcañiz, F.; Asensio, A.; Sánchez-Roselló, M.; Escolano, M.; del Pozo, C.; Fustero, S. *J. Org. Chem.* **2017**, *82*, 2505–2514. doi:10.1021/acs.joc.6b02880
7. Métay, E.; Léonel, E.; Sulpice-Gaillet, C.; Nédélec, J. Y. *Synthesis* **2005**, 1682–1688. doi:10.1055/s-2005-865364
8. Cesati, R. R.; de Armas, J.; Hoveyda, A. H. *Org. Lett.* **2002**, *4*, 395–398. doi:10.1021/ol017090c
